# Distal colonocytes targeted by *C. rodentium* recruit T-cell help for barrier defence

**DOI:** 10.1038/s41586-024-07288-1

**Published:** 2024-04-10

**Authors:** Carlene L. Zindl, C. Garrett Wilson, Awalpreet S. Chadha, Lennard W. Duck, Baiyi Cai, Stacey N. Harbour, Yoshiko Nagaoka-Kamata, Robin D. Hatton, Min Gao, David A. Figge, Casey T. Weaver

**Affiliations:** 1 Department of Pathology, Heersink School of Medicine, University of Alabama at Birmingham, Birmingham, AL USA; 2 Department of Medicine, Heersink School of Medicine, University of Alabama at Birmingham, Birmingham, AL USA

**Keywords:** Adaptive immunity, Bacteria

## Abstract

Interleukin 22 (IL-22) has a non-redundant role in immune defence of the intestinal barrier^1–3^. T cells, but not innate lymphoid cells, have an indispensable role in sustaining the IL-22 signalling that is required for the protection of colonic crypts against invasion during infection by the enteropathogen *Citrobacter rodentium*^4^ (*Cr*). However, the intestinal epithelial cell (IEC) subsets targeted by T cell-derived IL-22, and how T cell-derived IL-22 sustains activation in IECs, remain undefined. Here we identify a subset of absorptive IECs in the mid–distal colon that are specifically targeted by *Cr* and are differentially responsive to IL-22 signalling. Major histocompatibility complex class II (MHCII) expression by these colonocytes was required to elicit sustained IL-22 signalling from *Cr*-specific T cells, which was required to restrain *Cr* invasion. Our findings explain the basis for the regionalization of the host response to *Cr* and demonstrate that epithelial cells must elicit MHCII-dependent help from IL-22–producing T cells to orchestrate immune protection in the intestine.

## Abstract (web summary)

The murine enteropathogen *Citrobacter rodentium* targets a specific subset of absorptive intestinal epithelial cells in the mid–distal colon, which stimulate T cells to produce sustained IL-22 signals to mitigate further spread of the pathogen.

## Main

The arc of an infectious response is framed by the tissue site targeted by a pathogen and the host defences that are mounted at that site. The interplay of innate and adaptive immune systems is coordinated with tissue-specific, non-immune cell populations to resist pathogen incursion, with specialization of immune response types determined by the class of pathogen. Immune defence against phagocyte-resistant intracellular pathogens or extracellular parasites and helminths depends on type 1 or type 2 responses, respectively, whereas defence against extracellular bacteria is orchestrated by type 3 immunity, which utilizes immune cells that produce IL-17 family cytokines and the IL-10 family cytokine IL-22^1,2,5^. These cytokines act on non-immune cells, including barrier epithelial cells, to bolster anti-microbial responses.

In some infections, a lack of one of these cytokines can be fatal^1,5^. *Cr* is a Gram-negative murine enteropathogen that is related to enterohaemorrhagic and enteropathogenic *Escherichia coli*, which form attaching and effacing (A/E) lesions on apical surfaces of IECs using type III secretion systems to deliver virulence proteins^6,7^. IL-22 produced by innate and adaptive immune cells is indispensable for host protection against *Cr*^1–4^, and acts on IECs to protect the epithelial barrier. Its production is spatially and temporally constrained^1,3,4^. Resident innate cells, particularly type 3 innate lymphoid cells^2,8^ (ILC3), produce IL-22 early in *Cr* infection, whereas CD4 T cells^3,9^ (T helper 17 (T_H_17) and T helper 22 (T_H_22)) become the dominant source later during the infection^4,10^. Accordingly, deficiency of IL-22 targeted to innate immune cells is fatal early in *Cr* infection^2,4^, whereas IL-22 deficiency targeted to T cells is fatal later^4^.

A basis for subdivision of labour among IL-22–producing immune cells has recently been defined^4^. Innate immune cells, although effective at restraining early *Cr* colonization, are sequestered in isolated mucosal lymphoid tissues and primarily act at a distance^4,10,11^, becoming ineffective as infection progresses. After a delay for their antigen-driven differentiation, T_H_17 and T_H_22 cells that migrate into the distal colon are required to deliver the IL-22 that is necessary to maintain the epithelial barrier and protect crypts from pathogen encroachment^4^. The ability of IL-22–producing T cells, but not innate immune cells, to induce robust, sustained activation of IECs may reflect their deployment in immediate proximity to IECs. Yet, how this T cell ‘help’ for IECs is delivered, and the identities of the epithelial cell populations affected, remain unknown. It has also been unclear why *Cr* infection is limited to the mid–distal large intestine^3,6,12^.

Here we identify a sublineage of absorptive IECs that is regionally restricted to the mid–distal colon and is targeted by *Cr*, explaining the tropism of *Cr* for this tissue niche. We find that IL-22 signalling activates a gene-expression programme in this population that is distinct from that of conventional absorptive IECs, which populate the proximal large and small intestines. We further identify a key role for antigen presentation by IECs in eliciting optimal IL-22 signalling from T cells. Thus, the host response to *Cr* infection reflects macro- and micro-geographical constraints on both pathogen and host that are contingent on MHC-restricted T cell help to IECs.

## Colonocyte subsets have distinct responses to *Cr*

**Fig. 1:**
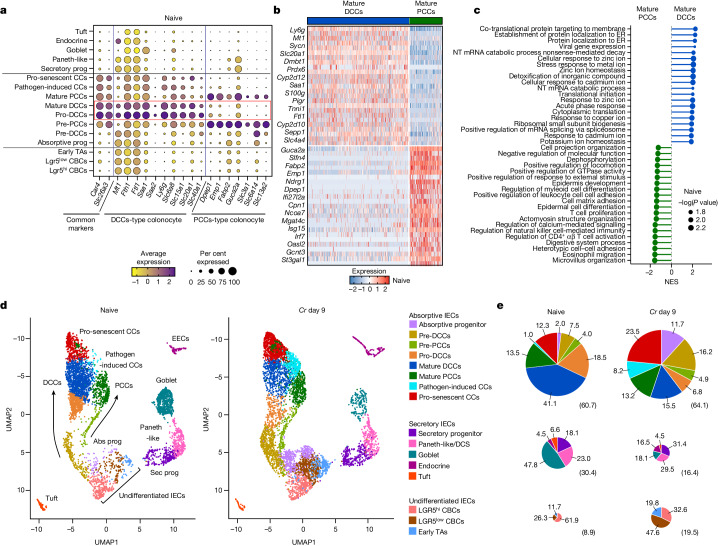
A distinct subset of colonocytes undergoes accelerated maturation in response to *Cr.* scRNA-seq was performed on epithelial cells from mid–distal colons of C57BL/6 mice without infection (naive) and on day 9 of *Cr* infection. **a**, Dot plot of differentially expressed genes in naive mice. Prog, progenitor; TA, transit-amplifying cell. **b**, Heat map of the top 50 differentially expressed genes between mature DCCs and PCCs from naive mice. **c**, Gene set enrichment analysis of Gene Ontology Biological Process pathways, comparing mature DCCs and PCCs from naive mice. ER, endoplasmic reticulum; NT, nuclear-transcribed. **d**, Uniform manifold approximation and projection (UMAP) analysis of integrated biological replicates identified eight unique clusters for absorptive IECs—including two major developmental arms (DCC and PCC), five clusters for secretory IECs and three clusters for undifferentiated IECs. Abs, absorptive; CC, colonocyte; DCS, deep crypt secretory cell; Sec, secretory, EEC, enteroendocrine cell. **e**, Pie charts of percentages of cells within each IEC subset. Numbers in parentheses show the percentages of absorptive (top row), secretory (middle row) and undifferentiated (bottom row) cells in each pool. Two mice pooled per sample, *n* = 2 biological replicates per group.

**Extended Data Fig. 1:**
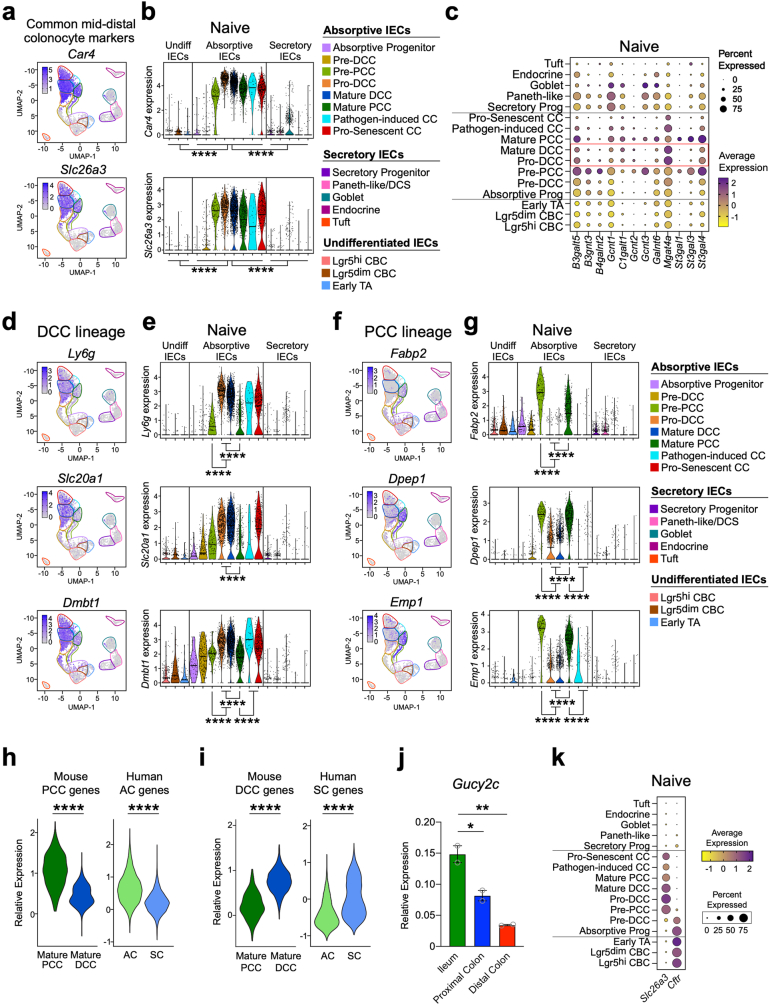
Characterization of colonocytes from naïve BL/6 mice. scRNA-seq was performed on epithelial cells from mid-distal colon of naïve BL/6 mice (n = 2). **a**, UMAPs and **b**, Violin plots of common colonocyte markers. One-way ANOVA; ****p≤0.0001 comparing absorptive IECs to undifferentiated or secretory IECs. **c**, Dot plot of glycoproteins expressed by IECs from naïve mice. **d**, UMAPs and **e**, Violin plots of DCC lineage markers. **f**, UMAPs and **g**, Violin plots of PCC lineage markers. One-way ANOVA; ****p≤0.0001 comparing gene expression in different cell clusters. See Supplementary Tables 3 and 4 for additional statistical analyses. **h**-**i**, Violin plots of manually curated genes expressed by (**h**) mouse mature PCCs (dark green) and human ascending colonic IECs (AC; light green) or (**i**) mouse Mature DCCs (dark blue) and human sigmoid colonic IECs (SC; light blue). Wilcoxon rank sum test; ****p≤0.0001. See Supplementary Table 1 for genes shared by mouse and human IECs. **j**, IECs from distal ileum (green), proximal colon (blue) and distal colon (red) of naïve BL/6 mice were sorted on EpCAM1^+^CD45^–^L/D dye^–^ IECs and *Gucy2c* mRNA expression analyzed by RT-PCR. One-way ANOVA; *p≤0.05 and **p≤0.01. **k**, Dot plot of *Slc26a3* and *Cftr* expression in IEC subsets from naïve BL/6 mice. 2 mice pooled per sample, *n* = 2 biological replicates per group. IEC=intestinal epithelial cell; PCC=proximal colonocyte; DCC=distal colonocyte; DCS=deep crypt secretory cell; TA=transit-amplifying cell; CBC=crypt base columnar cell; Undiff=undifferentiated cell; CFTR, cystic fibrosis transmembrane regulator. Note that *Car4* (carbonic anhydrase 4) and *Slc26a3* (chloride/bicarbonate transporter) are expressed by both PCC and DCC lineage colonocytes but not CBC cells, progenitor cells, or secretory IECs. In contrast, the chloride channel, CFTR is predominantly expressed by undifferentiated IECs and early colonocyte progenitors. Top genes expressed by naïve mature DCCs include *Ly6g*, *Slc20a1*, and *Dmbt1*. The top genes expressed by naïve pre- and mature PCCs include *Fabp2*, *Dpep1*, and *Emp1*. Although the top genes found in mouse mature PCCs and mature DCCs are not expressed by human ascending and sigmoid colonocytes, respectively, there are several genes that are shared between mouse and human colonocytes and differentially expressed in the two sides of the colon (see Supplementary Table 1). Moreover, *Gucy2c*, a transmembrane receptor important for regulating intestinal fluid secretion and a receptor for heat-stable enterotoxins from pathogenic *E.coli*^38^, is highly expressed in the ileum compared to the colon, suggesting that *E. coli* may utilize *Gucy2c* to efficiently target human ileal enterocytes as opposed to distal colonocytes. Interestingly, PCC and DCC lineage colonocytes express different glycoproteins which may dictate differences in cellular communication and interaction with specific molecules or neighboring cells.

Previous studies of the developmental and functional diversity of IECs have largely focused on the small intestine and have defined two major branches, absorptive and secretory^13,14^. To better characterize the diversity of IEC types in the large intestine as a prelude to exploring IEC responses in *Cr* infection, we performed single-cell RNA-sequencing (scRNA-seq) analysis on IECs from the mid–distal colon, the region colonized by this pathogen (Fig. 1). We identified a bifurcation of the absorptive colonocyte lineage, which we designated distal colonocytes (DCCs) and small intestine enterocyte-like proximal colonocytes (PCCs) (Fig. 1a–d and Extended Data Figs. 1a–g and 5a), as discussed below. Although these subsets derive from distinct early progenitors, their gene-expression profiles identified them as members of the absorptive lineage, as both subsets express *Car4*, *Slc26a3*^15,16^ and the cell-surface mucin genes *Muc3* and *Muc13*, which are main components of the protective glycocalyx^17^ (Fig. 1a and Extended Data Figs. 1a,b and 5a). However, mature DCCs differentially expressed *Ly6g*, which encodes a GPI-linked marker of mature neutrophils^18^, as well as genes encoding acute phase proteins (for example, *Saa1* and *Saa2*) that possess antimicrobial activity and can recruit and activate phagocytes^19,20^, and several solute carriers, such as *Slc20a1*, which encodes an inorganic sodium–phosphate cotransporter that can also regulate TNF-induced apoptosis^21^ (Fig. 1a,b and Extended Data Fig. 1d,e). DCCs also expressed higher levels of genes involved in metal ion storage^22,23^ (for example, *Mt1*, *Fth1* and *Slc40a1*), suggesting that they may sequester metal ions to reduce local metal toxicity and/or limit metal nutrient availability to commensal microbes.

By contrast, developing and mature PCCs expressed *Fabp2*, which encodes an intracellular protein involved in lipid metabolism^24^, *Guca2a*, which encodes a polypeptide that activates guanylate cyclase receptors to control water transport, and several glycosyltransferase genes that may contribute to mucosal barrier maintenance (Fig. 1a–c and Extended Data Fig. 1c,f,g). Mature DCCs and PCCs also differed in their expression of solute carrier transporters (for example, DCCs expressed *Slc20a1* and *Slc40a1* whereas PCCs expressed *Slc6a14* and *Slc13a2*). Thus, *Ly6g*^+^*Slc20a1*^+^*Saa1*^+^ DCCs and *Fabp2*^+^*Guca2a*^+^*Dpep1*^+^ PCCs represent distinct sublineages of the colonocyte absorptive lineage with gene-expression profiles suggestive of fundamental functional specialization. Genes that were differentially expressed between human ascending and sigmoid colon (manually curated from Gut Cell Atlas (https://www.gutcellatlas.org/)) were also found to be disparate in mouse proximal and distal colon, respectively (Extended Data Fig. 1h,i and Supplementary Table 1). Of note, *Gucy2c*, the receptor for the heat-stable *E. coli* enterotoxin ST^25^, was most highly expressed in the ileum (Extended Data Fig. 1j)—the main site of *E. coli* infection, suggesting that *E. coli* and *Cr* may be specialized for colonization of different regional IECs.

**Extended Data Fig. 2:**
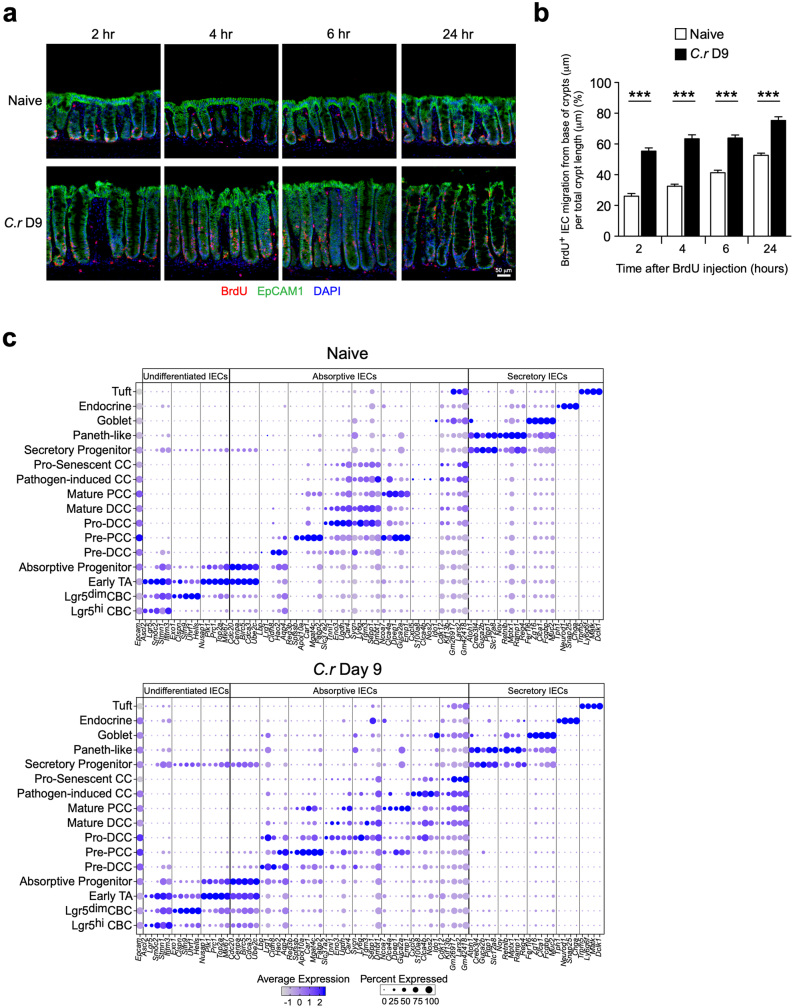
*C.r* infection causes enhanced IEC proliferation in the crypts. **a**, Colon tissue was collected at various times after d9 *C.r*-infected BL/6 mice were treated with BrdU and stained for BrdU (red), EpCAM1 (green) and DAPI (blue). Scale bar, 50 μm. **b**, Percent migration of BrdU^+^ IECs from the crypt base to the luminal surface per total length of the crypt. 4 mice per time point per group, 20–30 crypts per group, *n* = 1. One-way ANOVA; ***p≤0.001. Data are represented as mean ± SEM. **c**, Dot plots of top markers for all populations from scRNA-seq analysis of IECs from naïve and d9 *C.r*. 2 mice pooled per sample, *n* = 2 biological replicates per group. IEC=intestinal epithelial cell; PCC=proximal colonocyte; DCC=distal colonocyte; DCS=deep crypt secretory cell; TA=transit-amplifying cell; CBC=crypt base columnar cell.

**Extended Data Fig. 3:**
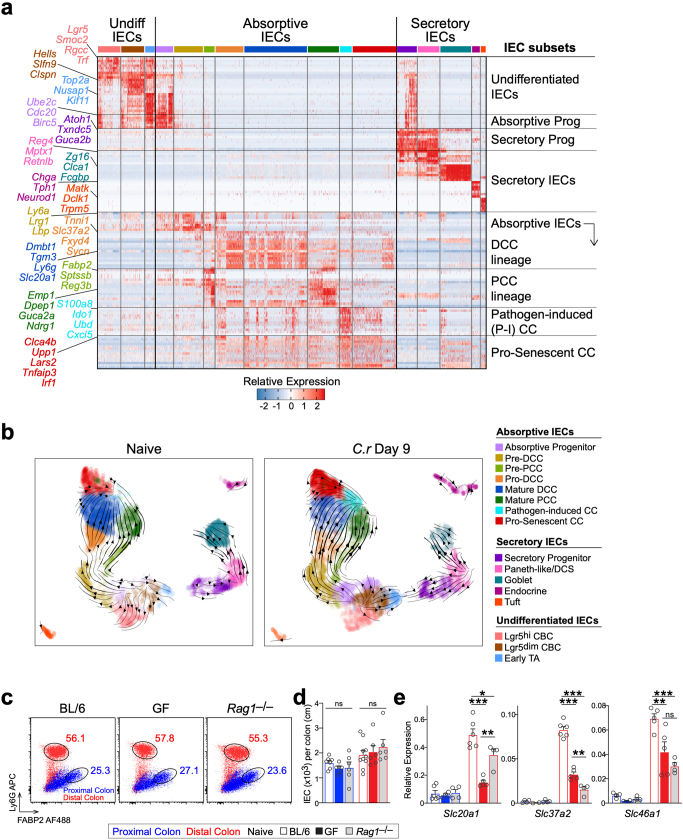
DCC lineage cells expand in response to *C.r* infection and respond to microbes and localized T cells. scRNA-seq was performed on epithelial cells from mid-distal colon of naïve BL/6 mice. **a**, Heatmap of top genes from combined naïve and infected BL/6 mice defining each major IEC subset. **b**, scVelocity plots show transcriptional relationships between the major IEC subsets. Arrowheads denote directionality and lines represent kinetics of differentiation. 2 mice pooled per sample, *n* = 2 biological replicates per group. Wilcoxon rank sum test, *p*-val<0.05 was used for differential gene expression analyses. See Supplementary Table 2 for top expressed genes per cluster. **c**-**e**, IECs from proximal colon (blue) and distal colon (red) of naïve BL/6 (white), GF (color) and *Rag1*^–/–^ (grey) mice were stained for Ly6G, Fabp2, EpCAM1, CD45 and L/D dye, and analyzed by flow cytometry (**c**) and enumerated per cm of tissue (**d**) or sorted on EpCAM1^+^CD45^–^L/D dye^–^ IECs and mRNA expression analyzed by RT-PCR (**e**). 3-4 mice per group; *n* = 2 independent experiments. Two-way ANOVA; *p≤0.05, **p≤0.01 and ***p≤0.001. IEC=intestinal epithelial cell; PCC=proximal colonocyte; DCC=distal colonocyte; DCS=deep crypt secretory cell; TA=transit-amplifying cell; CBC=crypt base columnar cell; Abs= absorptive; Sec=secretory; Undiff=undifferentiated; Prog=progenitor; ns=not significant.

Colonic hyperplasia—elongation of the crypts associated with expansion of undifferentiated crypt base columnar (CBC) stem cells, transit-amplifying cells and progenitor IECs—is a hallmark of *Cr* infection^6,12,15^. It is thought to protect stem cells at the base of crypts by both distancing these cells from the pathogen and accelerating removal of *Cr*-laden IECs^14^ (Extended Data Fig. 2a,b). To characterize this response in the region colonized by *Cr*, we extended the scRNA-seq analysis to compare IECs from the mid–distal colon of naive versus *Cr*-infected mice (Fig. 1d,e). DCCs and PCCs differed in their response to *Cr* infection (Fig. 1d,e and Extended Data Figs. 2c and 3a,b). Progenitors of DCCs underwent considerable expansion, whereas PCCs showed modest changes in frequency, abundance or developmental programming (Fig. 1d,e and Extended Data Figs. 2c and 3a,b). Differentiated DCCs (pro-DCC and mature DCC) were reduced, with a concomitant increase in a population of ‘pathogen-induced colonocytes’ (P-ICCs) that expressed IL-22- and IFNγ-inducible host defence genes^1,26–28^ (for example, *S100a8*, *Cxcl5* and *Ido1*) and colonocytes with a gene-expression profile indicating pro-senescence^29–31^ (for example, *Upp1*, *Lars2* and *Tnfaip3*) (Fig. 1d,e, Extended Data Fig. 3a and Supplementary Table 2). scRNA-seq velocity analysis indicated that DCC sublineage cells underwent rapid maturation and an altered cell fate to become ‘hyperactive’ P-ICCs that contribute to host defence (Extended Data Fig. 3b).

The abundance of undifferentiated IECs (Lgr5^+^ CBC cells^14^ and early transit-amplifying cells) increased, whereas that of goblet cells was reduced in response to *Cr* infection (Fig. 1d,e), consistent with previous reports implicating IFNγ-producing CD4 T cells in control of stem cell proliferation and goblet cell reprogramming and hypoplasia during *Cr* infection^32^. In addition, some colonic *Reg4*^+^*Mptx1*^+^ Paneth-like cells^33^ (or deep crypt secretory cells) dedifferentiated during *Cr* infection, paralleling plasticity in Paneth cells of small intestine that transition into CBC-like stem cells in response to inflammation and injury^34^. These findings identify unanticipated heterogeneity within the colonocyte absorptive lineage and implicate the newly identified DCC subset as the dominant responder to *Cr* infection. They further indicate that a developmental shift in colonic Paneth-like cells may contribute to the reduction in goblet cells (hypoplasia) and expansion of DCC progenitors during *Cr* infection. Together, these results establish that colonic hyperplasia in response to *Cr* infection predominantly comprises accelerated expansion of DCCs to generate P-ICCs, which show increased expression of cytokine-induced host defence molecules, and pro-senescent colonocytes, which appear to be programmed for accelerated cell death.

## DCCs are the principal targets of *Cr* infection

**Fig. 2:**
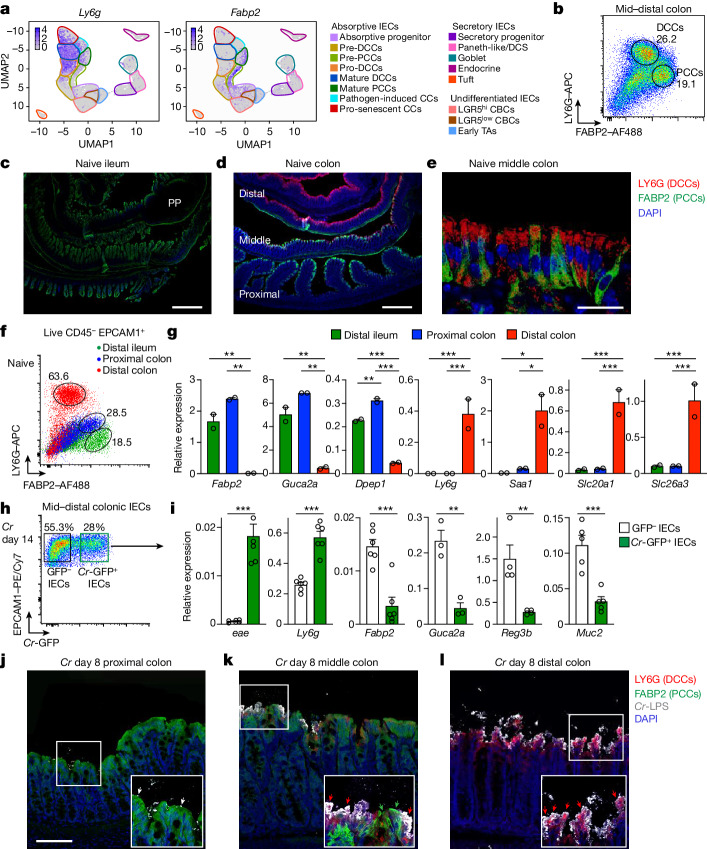
*Cr* predominantly attaches to DCCs. scRNA-seq was performed on epithelial cells from mid–distal colons of C57BL/6 mice without infection (naive) and on day 9 of *Cr* infection. (*n* = 2). **a**, UMAP analysis of *Ly6g* and *Fabp2* expression. **b**, IECs from mid–distal colons of naive mice were stained for LY6G, FABP2, EPCAM1, CD45 and LIVE/DEAD (L/D) dye, and analysed by flow cytometry. *n* = 3 mice and *n* = 2 independent experiments. **c**–**e**, Tissue from distal ileum (**c**; scale bar, 1,000 μm), colon (**d**; scale bar, 1,000 μm) and middle colon region (**e**; scale bar, 50 μm) from naive mice were stained with LY6G and FABP2 antibodies and DAPI (3–4 mice per region, *n* = 2 independent experiments). **f**,**g**, IECs from distal ileum (green), proximal colon (blue) and distal colon (red) of naive mice were stained as in **b** and analysed by flow cytometry (**f**) or sorted as EPCAM1^+^CD45^−^L/D^−^ IECs, and mRNA expression was analysed by PCR with reverse transcription (RT–PCR) (**g**) (2 or 3 mice pooled per sample; *n* = 2 independent experiments). One-way ANOVA. **h**, Mid–distal colon IECs mice on day 8 of *Cr*-GFP infection were sorted for EPCAM1^+^CD45^–^GFP^–^ or EPCAM1^+^CD45^–^*Cr*-GFP^+^ cells. **i**, mRNA expression of indicated genes in sorted *Cr*-GFP^–^ and *Cr*-GFP^+^ IECs was analysed by RT–PCR (2 or 3 mice pooled per sample; *n* = 2 independent experiments). Two-tailed unpaired *t*-test. **j**–**l**, Proximal (**j**), middle (**k**) and distal (**l**) colon tissue from mice on day 8 of *Cr* infection was stained for LY6G, FABP2, *Cr*-LPS and DAPI. White arrows identify rare *Cr* in proximal colon, green arrows identify FABP2^+^ PCCs, and red arrows identify *Cr*-laden LY6G^+^ DCCs. Scale bar, 100 μm. 3 or 4 mice per experiment; *n* = 2 independent experiments. Data are mean ± s.e.m. *P ≤ 0.05, **P ≤ 0.01 and ***P ≤ 0.001.

The studies described above identified differential responses of DCCs and PCCs to *Cr* but did not identify the basis of the difference. We considered that this might reflect geographic segregation of these two subsets, differential interactions with *Cr*, or both. To explore the first possibility, we evaluated the distribution of DCCs and PCCs along the intestinal tracts of naive mice, using subset-specific markers identified by scRNA-seq studies. We used LY6G as a DCC-specific marker and FABP2 as a PCC-specific marker (Fig. 2a,b and Extended Data Fig. 1d–g). LY6G^+^ cells (DCCs) were found exclusively in the distal colon and were interspersed with FABP2^+^ cells (PCCs) in the middle colon, whereas FABP2^+^ cells dominated proximally in the colon and in the terminal ileum of the small intestine (Fig. 2c–e), hence the designations ‘distal’ and enterocyte-like ‘proximal’ for DCCs and PCCs, respectively.

To confirm these findings, we validated gene-expression profiles by RT–PCR. Epithelial cells from the ileum and proximal colon expressed *Fabp2*, *Guca2a* and *Dpep1*—signature PCC-type genes—whereas cells from the distal colon expressed *Ly6g*, *Saa1* and *Slc20a1*—signature DCC genes (Fig. 2f,g, Extended Data Figs. 1d–g, 2c and Supplementary Tables 3 and 4). Notably, *Slc26a3*, which encodes a chloride/bicarbonate transporter associated with congenital chloride diarrheal disease^35^ was primarily expressed by pro- and mature DCCs (Fig. 2g and Extended Data Fig. 1a,b,k), whereas undifferentiated and immature DCCs utilized a different chloride channel, cystic fibrosis transmembrane conductance regulator^36^ (CFTR) (Extended Data Fig. 1k).

To determine whether development of the colonocyte subsets might be driven by interactions with the commensal microbiota or adaptive immune cells, we assessed relative abundance in specific pathogen-free (SPF), germ-free (GF) and *Rag1*^–/–^ mice (Extended Data Fig. 3c–e). There was no significant difference in the distribution or number of DCCs or PCCs among these mice, indicating that development of both cell types is largely independent of signals from the commensal flora or T and B cells (Extended Data Fig. 3c,d), although several DCC-specific genes were differentially expressed, including the SLC family members *Slc20a1*, *Slc37a2* and *Slc46a1* (Extended Data Fig. 3e). Collectively, these data indicate that DCCs represent a newly identified sublineage of absorptive IECs found only in the mid–distal colon. Moreover, DCCs appear to be constitutively responsive to commensals or microbial activation of the adaptive immune system and may sequester metal nutrients to regulate the barrier in this region.

**Extended Data Fig. 4:**
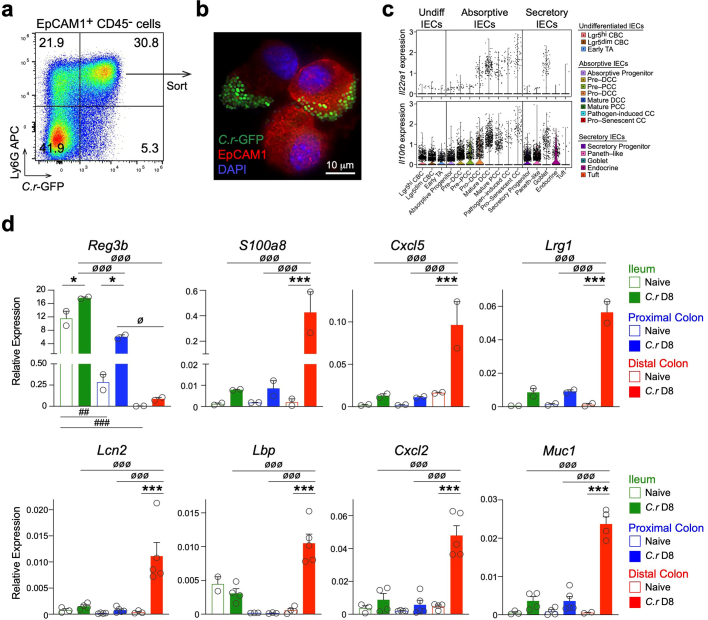
IL-22–inducible genes are differentially upregulated in different regions of the intestines. **a**-**b**, Mid-distal colon IECs from d8 *C.r*-GFP-infected mice were stained for EpCAM1, Ly6G, and CD45 and either **a**) analyzed by flow cytometry or **b**) sorted on EpCAM1^+^ (red) *C.r*-GFP^+^ (green) cells, cytospun, and stained with DAPI (blue). Scale bar = 10 μm. 3 mice per group and *n* = 2 independent experiments. **c**, scRNA-seq was performed on epithelial cells from mid-distal colon of naïve BL/6 mice (n = 2). Violin plots of *Il22ra1* and *Il10rb* genes. Dots denote gene expression per individual cell. **d**, IECs from naïve (open symbol) and d8 *C*.r mice (closed symbol) were sorted from distal ileum (green), proximal colon (blue) and distal colon (red) and mRNA expression was analyzed by RT-PCR. One-way ANOVA with Tukey’s multiple comparison test; *p≤0.05, and ***p≤0.001 comparing naïve and infected mice; and ^øøø^p≤0.001 comparing infected samples from different tissue regions; and ^##^p≤0.01, and ^###^p≤0.001 comparing naïve samples from different tissue regions. 2-3 mice pooled per sample; *n* = 2 independent experiments. Data are represented as mean ± SEM. IEC=intestinal epithelial cell; PCC=proximal colonocyte; DCC=distal colonocyte; DCS=deep crypt secretory cell; TA=transit-amplifying cell; CBC=crypt base columnar cell. The components of the IL-22R (IL-22Ra1 and IL-10Rβ) were found to be highly expressed by mature colonocytes, with no significant difference in IL-22R expression between mature PCCs and mature DCCs. *S100a8/9* antimicrobial peptide, and the *Cxcl2* and *Cxcl5* chemokines are highly expressed by mature colonocytes of the DCC lineage and are heightened in the presence of IL-22–producing CD4 T cells suggesting they may play important roles in bacterial clearance during the late phase of *C.r* infection.

In view of the marked regionalization of DCCs to the same area colonized by *Cr*^3,4,6^ and their preferential infection-induced response (Fig. 1), we speculated that mature DCCs may be targets of *Cr* colonization. To test this, we used a GFP-expressing *Cr* variant to sort uninfected and infected IECs from mid–distal colons of wild-type mice and screened for DCC- and PCC-specific genes (Fig. 2h,i and Extended Data Fig. 4a,b). As a control and internal validation, the *Cr*-specific gene *eae*, which encodes intimin, a molecule crucial for *Cr* attachment to IECs, was strictly limited to *Cr*-GFP-laden IECs. Expression of *Ly6g*, which encodes a marker for DCCs, was significantly enriched in infected *Cr*-GFP^+^ IECs but was also found in uninfected *Cr*-GFP^–^ IECs, establishing that *Cr* binds directly to DCCs, albeit non-uniformly. The non-uniformity is likely to reflect the lack of *Cr* attachment to some mature DCCs, pro-DCCs and P-ICCs that express LY6G but are sequestered within crypts, whereas *Cr*-GFP^+^ IECs appear to be infected mature DCCs and pro-senescent DCCs that are fated to be shed and removed from the colon. Notably, genes that define PCCs (for example, *Fabp2*, *Guca2a* and *Reg3b*) or goblet cells (for example, *Muc2*) were largely restricted to uninfected IECs, indicating that most PCCs are not bound by *Cr*.

To extend these findings, we examined *Cr* attachment to either LY6G^+^ DCCs or FABP2^+^ PCCs in *Cr*-GFP-infected mice *in situ* (Fig. 2j–l). There was minimal epithelial attachment of *Cr* in the proximal colon. In the middle colon, which is populated by both DCCs and PCCs, *Cr* attached to luminal LY6G^+^ DCCs but to very few FABP2^+^ PCCs. In the distal colon, where the absorptive epithelium is comprised almost exclusively of DCCs, the majority of superficial LY6G^+^ DCCs were bound by *Cr*, demonstrating that *Cr* preferentially colonizes DCCs found in the mid–distal colon. Thus, *Cr* shows strong tropism for DCCs at the luminal surface, explaining the restriction of colonization by this pathogen to the mid–distal colon.

## PCCs and DCCs respond differentially to IL-22

**Fig. 3:**
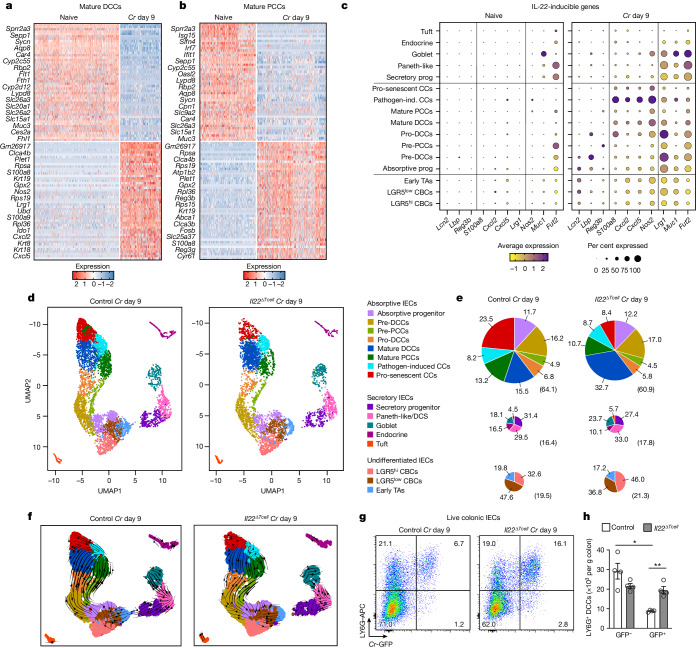
IL-22^+^ T cells accelerate removal of *Cr*-laden mature DCCs. scRNA-seq was performed on epithelial cells from mid–distal colons of C57BL/6 mice without infection (naive) and on day 9 of *Cr* infection of *Il22*^*hCD4*^ (control)^4^ and *Il22*^*∆Tcell*^ conditional knockout (cKO) mice (*n* = 2). **a**,**b**, Heat map of top 50 differentially expressed genes in mature DCCs (**a**) and mature PCCs (**b**), comparing naive and infected mice. **c**, Dot plot of IL-22–inducible genes from naive and infected mice. **d**, UMAP analysis of integrated biological replicates from day 9 of *Cr* infection of control and *Il22*^*∆Tcell*^ cKO mice. **e**, Pie charts show the percentage of cells within each IEC subset. Numbers in parentheses show percentages of absorptive (top row), secretory (middle row) and undifferentiated (bottom row) cells in each pool. **f**, scRNA-seq velocity plots highlight transcriptional relationships between major IEC subsets. Arrowheads denote directionality and lines represent kinetics of differentiation. **g**, IECs from colons on day 9 of *Cr*-GFP infection of *Il22*^*hCD4*^ and *Il22*^*∆Tcell*^ mice were stained for LY6G, CD45, L/D dye and EPCAM1 and analysed by flow cytometry. **h**, Number of *Cr*-GFP^–^LY6G^+^ IECs and *Cr*-GFP^+^LY6G^+^ IECs from *Il22*^hCD4^ (white) and *Il22*^*∆Tcell*^ (grey) mice on day 9 of infection with *Cr*-GFP. Two-tailed unpaired *t*-test; 3 or 4 mice per group; *n* = 2 independent experiments. Data are mean ± s.e.m. *P ≤ 0.05.

**Extended Data Fig. 5:**
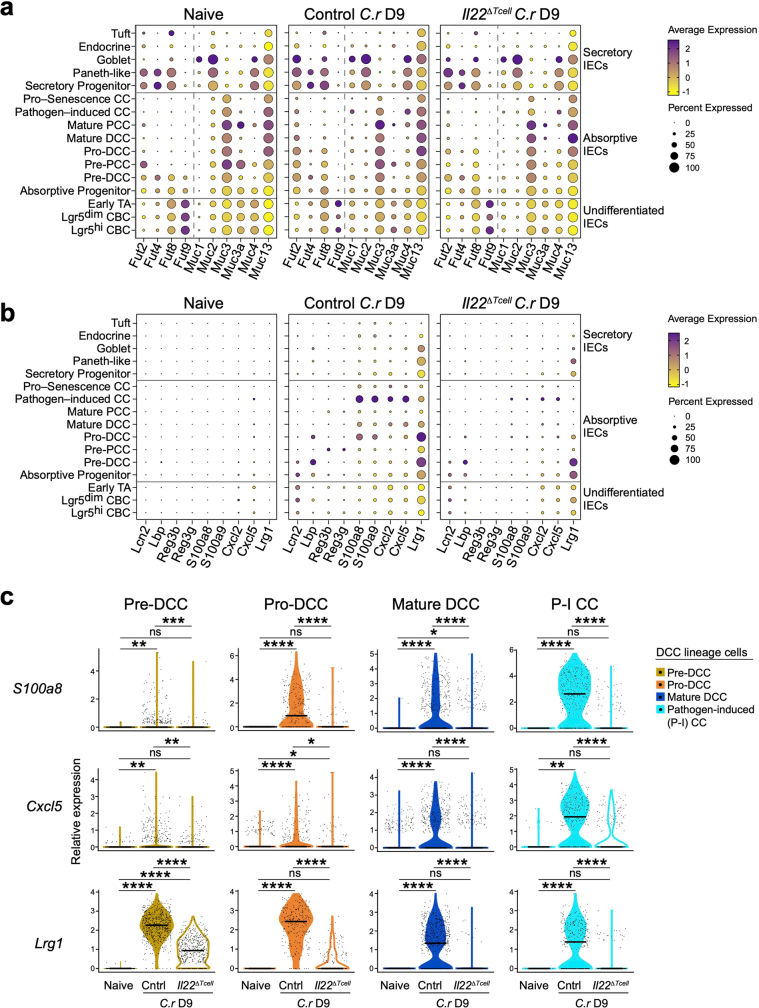
CD4 T cells upregulate IL-22–inducible genes on DCC lineage cells and goblet cells for host defense. scRNA-seq was performed on epithelial cells from mid-distal colon of naïve BL/6, and d9 *C.r Il22*^hCD4^ (Control) and *Il22*^∆Tcell^ cKO mice. 2-3 mice pooled per sample; *n* = 2 independent experiments. **a**-**b**, Dot plots of fucosyl transferases and mucins (**a**) and IL-22–inducible genes (**b**) from naïve, and d9 *C.r Il22*^hCD4^ and *Il22*^∆Tcell^ mice. **c**, Violin plots of *S100a8*, *Cxcl5* and *Lrg1* expression in pre-DCC (beige), pro-DCC (orange), mature DCC (royal blue) and pathogen-induced (P-I) CC from naïve, and *C.r* d9 *Il22*^fl/fl^ and *Il22*^∆Tcell^ mice. Bars represent mean gene expression and dots denote gene expression per individual cell. One-way ANOVA; *p≤0.0332, **p≤0.0021, ***p≤0.0002 and ****p≤0.0001 comparing naïve, and d9 *C.r* Control and *Il22*^∆Tcell^. ns=not significant. DCS=deep crypt secretory cell; TA=transit-amplifying cell; CBC=crypt base columnar cell. As expected in naïve mice, goblet cells predominantly express *Muc2* and *Muc4*; whereas colonocytes express *Muc3* and *Muc13*. *Fut2* gene which encodes for the fucosyl transferase 2 enzyme is upregulated on both goblet cells and colonocytes; whereas *Muc1*, a membrane-bound mucin is predominantly upregulated on stem cells and colonocytes of the DCC lineage during *C.r* infection. *S100a8*, *Cxcl5* and *Lrg1* are significantly upregulated on multiple DCC lineage subsets in response to T cell-derived IL-22.

**Extended Data Fig. 6:**
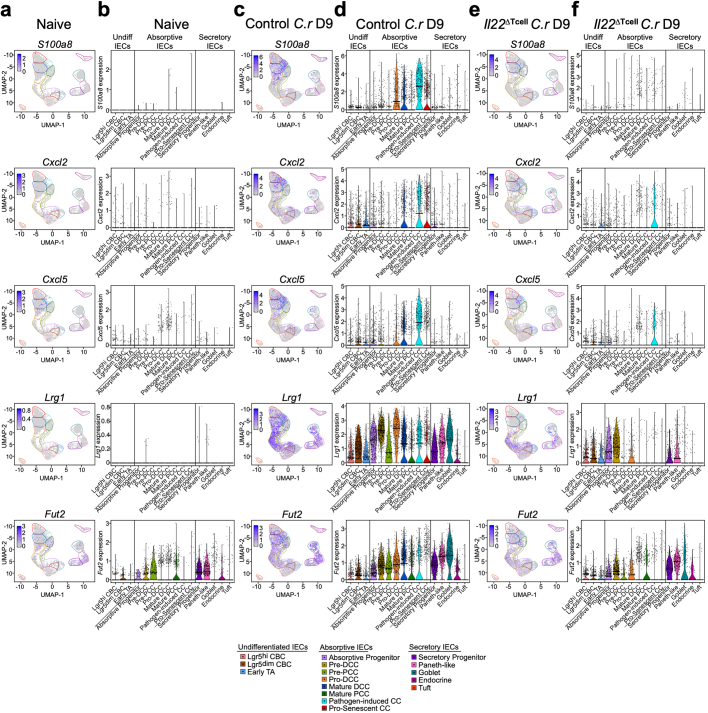
CD4 T cells upregulate IL-22–inducible genes on multiple colonic IEC subsets. scRNA-seq was performed on epithelial cells from mid-distal colon of naïve BL/6, and d9 *C.r Il22*^hCD4^ (Control) and *Il22*^∆Tcell^ cKO mice (n = 2). **a**, UMAPs and **b**, Violin plots from naïve BL/6 mice. **c**, UMAPs and **d**, Violin plots from *C.r* d9 Control mice. **e**, UMAPs and **f**, Violin plots from *C.r* d9 *Il22*^∆Tcell^ mice. See Supplementary Tables 5–7 for statistical analyses. Bars represent mean gene expression and dots denote gene expression per individual cell. *S100a* family of AMPs and neutrophil-recruiting chemokines (e.g., *Cxcl2* and *Cxcl5*) are predominantly upregulated on IECs of the mature DCC lineage in infected control mice and expression of these genes is severely blunted in the absence of IL-22–producing T cells. *Lrg1*, a leucine-rich α-2-glycoprotein that plays a role in cell migration and wound healing^42^ is upregulated on all IECs during *C.r* infection. IL-22–producing T cells augment Lrg1 expression on most IECs with the greatest expression observed on pre-DCCs and pro-DCCs suggesting that T cell-driven upregulation of *Lrg1* may contribute to the expansion of distal colonocytes and ultimate restitution of the damaged epithelium during *C.r* infection. *Fut2*, another known IL-22–regulated gene is upregulated on goblet cells and colonocytes of the DCC lineage in response to IL-22^+^ T cells on d9 of *C.r* infection.

Given the differential targeting of absorptive colonocyte subsets by *Cr*, we speculated that there might be stratification of host defence responses between DCCs and PCCs. Comparison of gene-expression profiles of DCCs and PCCs by scRNA-seq indicated that this was indeed the case, and it did not appear to be due to disparate expression of the IL-22 receptor transcripts (*Il22ra1* and *Il10rb*). Whereas both DCCs and PCCs downregulated many genes during *Cr* infection (for example, *Car4*, *Slc26a3* and several differentially expressed solute carrier genes), indicating that both colonocyte subsets sensed the pathogen and altered their response^15,16,37^, many genes that we had previously reported as contingent on T cell-derived IL-22^4^ were upregulated specifically in DCCs (Fig. 3a–c and Extended Data Figs. 4c,d, 5b,c and 6). Notably, the S100a gene family (for example, *S100a8*) encoding antimicrobial peptides (AMPs) and genes encoding neutrophil-recruiting chemokines (for example, *Cxcl2* and *Cxcl5*) were strongly upregulated in DCC lineage cells (pro-DCC, mature DCC and P-ICC) in infected control mice compared with naive mice.

A specific contribution of DCCs to host defence included production of neutrophil-recruiting chemokines that are required for *Cr* eradication^38^ (Fig. 3a–c). This included marked IL-22–induced enrichment of these chemokine transcripts in P-ICCs (Fig. 3c and Extended Data Figs. 5b,c). In addition, pro-DCC, mature DCCs and P-ICCs specifically upregulated *Nos2*, which encodes inducible enzyme nitric oxide synthase; this is important for production of NO, a broad-spectrum antimicrobial molecule that causes nitrosative and oxidative damage^39^. Furthermore, immature subsets of DCCs upregulated the IL-22–inducible genes *Lcn2*, *Lbp* and *Fut2*, indicating that colonocytes in the mid–lower crypts are also capable of sensing and responding to *Cr* infection. By contrast, upregulation of the IL-22–inducible *Reg3* lectin family of AMP genes by *Cr* infection was PCC-specific. Because *Cr*-infected *Reg3b*^−/−^ mice have increased bacterial load^40,41^, this suggests that PCCs may contribute to host defence by reducing luminal bacteria that can attach to DCCs, although other mechanisms, including modulation of the commensal microbiota, cannot be excluded.

Consistent with these findings, IL-22–induced transcripts of IECs isolated from the distal colon, proximal colon or distal ileum of naive and *Cr*-infected wild-type mice correlated well with the distribution of DCCs and PCCs across these intestinal regions (Extended Data Fig. 4d). Consistent with published reports, we found that *Reg3b* was expressed predominantly in the small intestine, with low levels of *Reg3b* expression in the proximal colon at steady state^41^. At the peak of *Cr* infection (day 8–9 after infection), *Reg3b* was upregulated on IECs from the distal ileum and proximal colon but not the distal colon. By contrast, other IL-22–inducible genes such as *S100a8*, *Cxcl5* and *Lrg1* were predominantly upregulated in the distal colon, but not in ileum or proximal colon. Together, these data establish that upregulation of *S100a8* or *S100a9* and neutrophil-attractant chemokine genes reflects direct targeting of DCCs by *Cr*, whereas PCCs upregulate *Reg3* family transcripts, which may promote pathogen clearance in the lumen. DCCs and PCCs thus appear to be programmed for distinct biological activities downstream of IL-22.

This was confirmed by scRNA-seq analysis of mid–distal IECs from *Cr*-infected *Il22*^*fl/fl*^ (control) and *Il22*^*∆Tcell*^ (T cell-specific deletion of IL-22) mice (Fig. 3d,e and Extended Data Figs. 5a,b and 6); the induction of S100 family AMPs or neutrophil-attractant chemokines or REG3 family AMPs was DCC- or PCC-specific, respectively, and contingent on actions of T cell-derived IL-22 (Extended Data Figs. 5a–c and 6 and Supplementary Tables 5–7). By contrast, and similar to the expansion of stem cells and reprogramming of goblet cells, which was comparable between *Cr*-infected control and *Il22*^*∆Tcell*^ mice and consistent with their regulation by IFNγ^32^, but not IL-22, the expansion of absorptive progenitors and immature pre-DCCs was not controlled by T cell-derived IL-22. Moreover, scRNA-seq velocity analysis indicated that infection-driven transition from immature to mature colonocyte subsets in control mice progressed with a pattern of constant acceleration (Fig. 3f). However, we found a critical role for T cell-derived IL-22 in programming DCCs to accelerate their transition to pro-senescence during infection (Fig. 3d–f), and the migration of maturing DCC subsets in infected *Il22*^*∆Tcell*^ mice was irregular (Fig. 3f), suggesting discontinuous terminal differentiation. The aberrant transition of DCCs to pro-senescent colonocytes in *Cr*-infected *Il22*^*∆Tcell*^ mice suggested that IL-22–producing T cells act to promote the more rapid removal of *Cr*-laden colonocytes.

Supporting this idea, we found a significant increase in live *Cr*-GFP^+^ DCCs in *Cr-*GFP-infected *Il22*^*∆Tcell*^ mice compared with controls (Fig. 3g,h). In addition, compared with *Cr*-GFP-infected *Il22*^*∆Tcell*^ mice, control mice had significantly higher numbers of GFP^–^ DCCs compared with GFP^+^ DCCs, further implicating IL-22 produced by T cells in promoting replacement of *Cr*-laden DCCs with newly generated DCCs. In this regard, we found it notable that T cell-derived IL-22 augmented *Lrg1* expression at multiple stages of DCC development. *Lrg1*, which encodes a leucine-rich α-2-glycoprotein involved in IEC migration and wound healing^42^, was upregulated by most IECs during *Cr* infection but was most highly expressed in immature DCCs (pre-DCC and pro-DCC), suggesting that *Lrg1* may have a role in the movement of DCCs up the crypts during *Cr* infection (Fig. 3c and Extended Data Figs. 2c, 5b,c and 6). Together, these findings indicate that the delivery of IL-22 to DCCs by CD4 T cells is required both to activate these cells for enhanced anti-bacterial defence and to accelerate the removal and replacement of *Cr*-laden colonocytes at the luminal surface to expedite pathogen clearance, representing a novel mechanism by which IL-22 signalling into DCCs may counter *Cr*-mediated effector mechanisms to promote the retention of infected DCCs to which they anchor^43,44^.

## Epithelial MHCII is required to recruit T-cell help

**Fig. 4:**
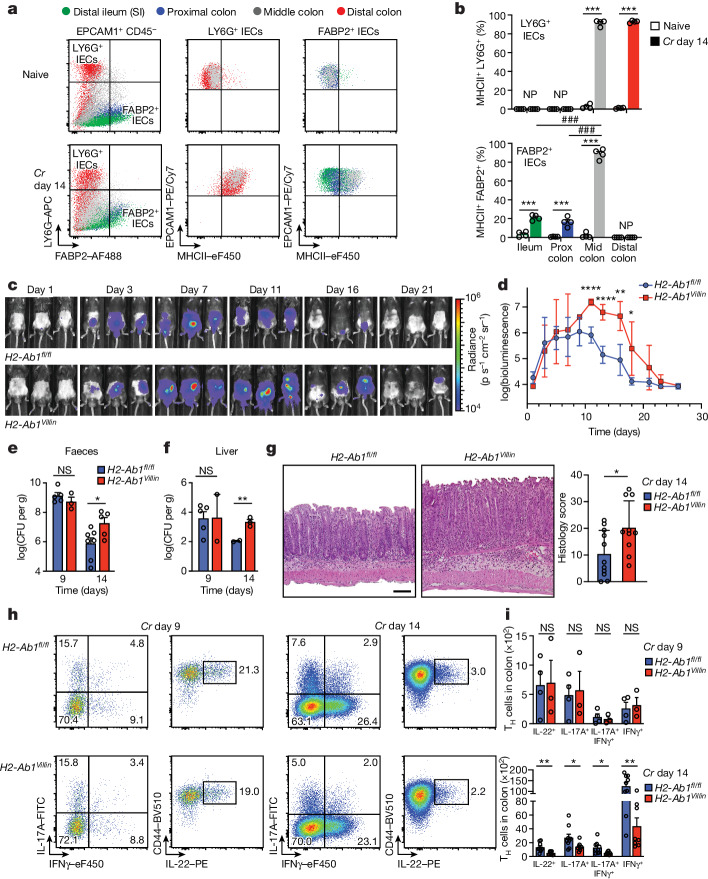
Epithelial MHCII is required to limit bacterial overgrowth and intestinal pathology. **a**, IECs from distal ileum (green), and proximal (blue), middle (grey) and distal (red) colon of C57BL/6 mice without infection (naive) and on day 14 of *Cr* infection were stained for EPCAM1, CD45, LY6G, FABP2 and MHCII and L/D dye, and analysed by flow cytometry. SI, small intestine. **b**, Percentage of MHCII^+^LY6G^+^ DCCs and MHCII^+^ FABP2^+^ IECs from C57BL/6 mice without infection (naive) and on day 14 of *Cr* infection. Three mice pooled per region; *n* = 2 independent experiments. Two-way ANOVA. Asterisks indicate *P* values for naive versus infected mice. ^###^*P* ≤ 0.001 (comparing colonic regions). Mid, middle; NP, not present; Prox, proximal. **c**,**d**, Serial whole-body imaging (**c**) and colonization kinetics (**d**) of *Cr*-infected *H2-Ab1*^*fl/fl*^ (blue) and *H2-Ab1*^*Villin*^ (red) mice. Five mice per group; *n* = 2 independent experiments. **e**,**f**, Number of colony-forming units (CFU) from faeces (**e**) and liver (**f**) of day 9 and day 14 *Cr*-GFP *H2-Ab1*^*fl/fl*^ and *H2-Ab1*^*Villin*^ mice (4 or 5 mice per group, *n* = 2 independent experiments). **g**, Colons on day 14 of *Cr* infection of *H2-Ab1*^*fl/fl*^ and *H2-Ab1*^*Villin*^ mice, stained with haematoxylin and eosin. Scale bar, 100 μm. **h**, Colon cells from day 9 and day 14 *Cr H2-Ab1*^*fl/fl*^ and *H2-Ab1*^*Villin*^ mice were stimulated with phorbol 12-myristate 13-acetate (PMA) and ionomycin and stained for surface CD4, TCRβ, CD44 and L/D dye, then stained for intracellular IL-17, IL-22 and IFNγ and analysed by flow cytometry. **i**, Number of colonic IL-22^+^, IL-17A^+^, IL-17A^+^IFNγ^+^ and IFNγ^+^ T cells from day 9 and day 14 *Cr H2-Ab1*^*fl/fl*^ and *H2-Ab1*^*Villin*^ mice. Two-tailed unpaired *t*-test comparing *H2-Ab1*^*fl/fl*^ and *H2-Ab1*^*Villin*^ mice (**d**–**g**,**i**); 3 or 4 mice per group; *n* = 2 independent experiments. Data are mean ± s.e.m. NS, not significant.

The injection of bacterial effector proteins into host IECs is required for the attachment of *Cr* to superficial DCCs that line the luminal surface of the distal colon. This also represents a potential vulnerability for *Cr*, as these effectors may be sensed by intracellular pattern-recognition receptors and provide antigenic peptides recognized by T cells. Although antigenic priming of naive CD4 T cells to *Cr* is thought to occur in lymphoid tissues by type 2 conventional dendritic cells^45^, it is unclear whether *Cr* antigens can be presented by IECs to directly recruit CD4 T cell help^46^. Previous studies have found that IECs begin to upregulate MHCII and other components of the class II antigen processing and presentation (APP) system coincident with the influx of effector CD4 T cells into the infected mid–distal colon, leading to MHCII expression by the majority of IECs later in infection^4,16,46,47^. This coincided with sustained STAT3 activation in IECs driven by T cell-derived IL-22 signalling^4,16^. In the extension of these studies (Fig. 4a,b), we found that by day 14 of *Cr* infection, 90% of LY6G^+^ DCC and FABP2^+^ PCC cells from the mid–distal colon upregulate MHCII, compared with 20% of FABP2^+^ PCCs and distal illeal enterocytes. The coordination of IEC upregulation of the MHCII APP system with the recruitment of robust, T cell-dependent IL-22 signalling into IECs led us to posit a role for direct, antigen-dependent release of IL-22 by *Cr-*specific T_H_17 or T_H_22 cells.

**Extended Data Fig. 7:**
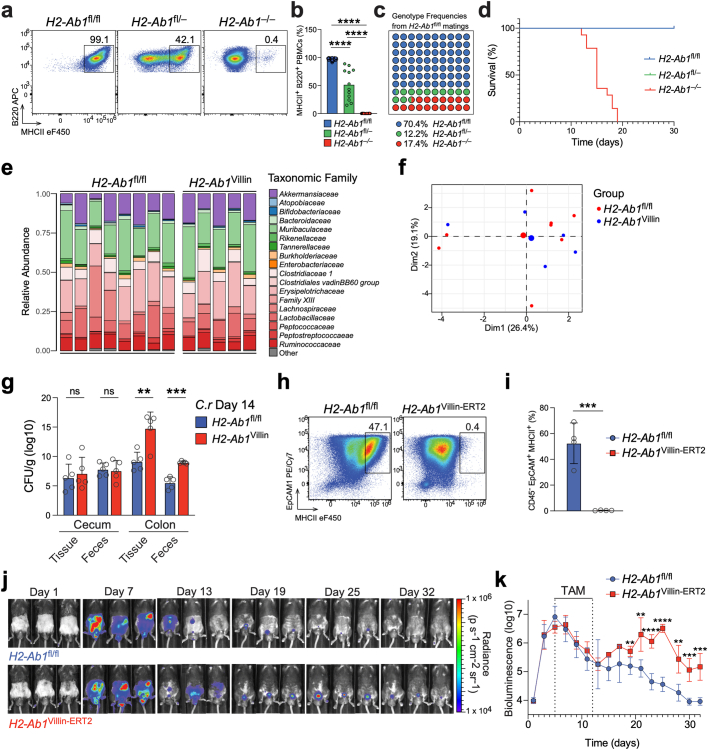
IEC-derived MHCII augments local T cell-driven protection of the colon during the late phase of *C.r* infection. Notably, we found that >20% of progeny from crosses of *H2-Ab1*^fl/fl^ x Villin-cre mice were either heterozygous or homozygous complete knockouts for *H2-Ab1* (**a**-**c**), presumably due to spontaneous recombination of the targeted allele. All mice with spontaneous global deficiency of *H2-Ab1* completely succumbed to *C.r* infection before d20, consistent with their inability to recruit adaptive immunity (**d**). However, properly targeted *H2-Ab1*^Villin^ mice that survived *C.r* infection had equivalent diversity of colonic commensals and displayed normal MHCII expression on PBMCs (**e**-**f**). **a**, Peripheral blood cells were isolated from naïve *H2-Ab1*^fl/fl^ and *H2-Ab1*^Villin^ littermates, stained for B220, MHCII and L/D dye and analyzed by flow cytometry to exclude mice with germline deletion of MHCII. **b**, Percent MHCII^+^ B220^+^ PBMCs from naïve *H2-Ab1*^fl/fl^ (blue), *H2-Ab1*^fl/–^ (green) and *H2-Ab1*^–/–^ (red) mice. **c**, Dot plot of the genotype frequencies of 100 offspring from *H2-Ab1*^fl/fl^ matings. n=at least 12 mice per group pooled from multiple litters. One-way ANOVA; ****p≤0.0001. **d**, Survival kinetics of *C.r*-infected control *H2-Ab1*^fl/fl^ (blue), *H2-Ab1*^fl/–^ (green) and *H2-Ab1*^–/–^ (red) mice. 5-6 mice per group, n = 2 independent experiments **e**, Fecal samples were collected from *H2-Ab1*^fl/fl^ and *H2-Ab1*^Villin^ littermates for 16 S rRNA-seq. **f**, PCA plots of individual samples used for 16 S rRNA-seq. 5–8 mice per group, n = 1 experiment **g**, Colon and cecum tissue and feces from d14 *C.r*-GFP *H2-Ab1*^fl/fl^ (blue) and *H2-Ab1*^Villin^ (red) mice was analyzed for *C.r* colony forming units (cfu) per gram of tissue or feces. 5 mice per group, *n* = 1 experiment. Two-tailed unpaired t-test; **p≤0.01 and ***p≤0.001. **h**, *C.r*-infected *H2-Ab1*^fl/fl^ and *H2-Ab1*^Villin-ERT2^ mice were administered tamoxifen on d5-d12 and IECs were isolated on d14 for flow cytometry to enumerate MHCII^+^ EpCAM1^+^ IECs. **i**, Percent MHCII^+^ EpCAM1^+^ IECs from d14 *C.r H2-Ab1*^fl/fl^ (blue) and *H2-Ab1*^Villin-ERT2^ (red) mice. 4 mice per group, n = 1 experiment. Two-tailed unpaired t-test; ***p≤0.001. **j**, Serial whole-body imaging and **k**, colonization kinetics of *C.r*-infected *H2-Ab1*^fl/fl^ (blue) and *H2-Ab1*^Villin-ERT2^ (red) mice that received tamoxifen on d5-d12. 5 mice per group, n = 2 independent experiments. Two-tailed unpaired t-test; *p≤0.0332, **p≤0.0021, ***p≤0.0002 and ***p≤0.0001 comparing *H2-Ab1*^fl/fl^ and *H2-Ab1*^Villin-ERT2^ mice. Data are represented as mean ± SEM. ns=not significant. PBMC=peripheral blood mononuclear cells.

To test this, we explored T cell and IEC responses to *Cr* infection in mice with specific deficiency of MHCII targeted to IECs (*H2-Ab1*^*Villin*^). Properly targeted *H2-Ab1*^*Villin*^ mice (Extended Data Fig. 7a–f) exhibited similar bacterial loads to *H2-Ab1*^*fl/fl*^ controls over the early course of infection (days 0–8; Fig. 4c,d) but had significantly increased bacterial burden over the late course (days 12–18). This correlated with similar *Cr* burdens in fecal contents and liver on day 9 but significantly increased burdens on day 14 after infection in *H2-Ab1*^*Villin*^ mice (Fig. 4e,f). Moreover, *Cr* burden was increased on day 14 in the colons of *H2-Ab1*^*Villin*^ mice compared with controls, but not in the caecum (Extended Data Fig. 7g). This correlated with significantly greater colonic inflammation and disease scores in *H2-Ab1*^*Villin*^ mice compared with *H2-Ab1*^*fl/fl*^ controls (Fig. 4g). To exclude aberrant effects potentially conferred by IEC MHCII deficiency from birth, we also analysed *H2-Ab1*^*Villin-ERT2*^ mice, which enabled us to delete MHCII after initiation of infection. Similar to *Cr*-infected *H2-Ab1*^*Villin*^ mice, we found significantly increased bacterial burden in *H2-Ab1*^*Villin-ERT2*^ mice late in infection (Extended Data Fig. 7h–k). On day 9 of infection, when MHCII expression is normally upregulated on a small fraction of IECs^4^, there was no difference in T_H_17 and T_H_22 cell numbers in colons of infected *H2-Ab1*^*Villin*^ mice compared with controls, consistent with a dispensable epithelial MHCII effect on effector T cells early in infection (Fig. 4h,i). In contrast, by day 14 of infection, when the majority of mid–distal colonocytes normally express MHCII, there was a more than 50% reduction in the numbers of T_H_17 and T_H_22 cells in the colons of infected *H2-Ab1*^*Villin*^ mice compared with controls.

**Fig. 5:**
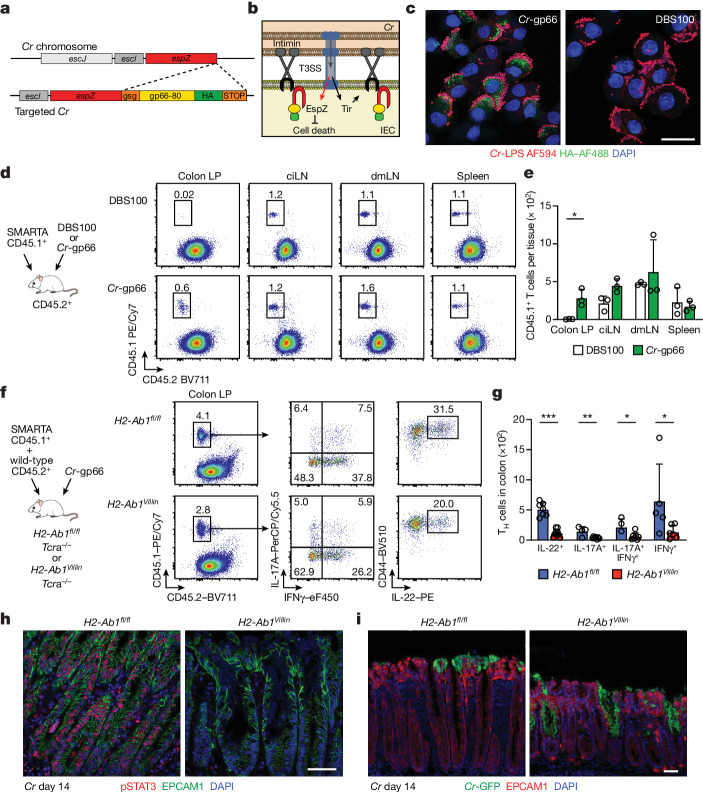
Epithelial MHCII is required for mucosal retention of *Cr*-specific T_H_ cells, prolonged colonocyte STAT3 activation and crypt protection. **a**, Design of the *Cr*-*espZ*-gp66-HA (*Cr*-gp66) construct. **b**, Schematic of *Cr*–host IEC interaction, highlighting injection of the *Cr* effector proteins Tir (targeted to IEC apical membrane) and EspZ (gp66–HA-tagged and associated with Tir cytosolic domain). **c**, Distal colon EPCAM1^+^LY6G^+^L/D^–^CD45^–^ cells from *Cr*-gp66 or *Cr* (DBS100) infected mice (day 8 of infection) were sorted and stained for HA and LPS and with DAPI. Scale bar, 20 μm. 2 or 3 mice per group; *n* = 2 experiments. **d**, CD45.1^+^ SMARTA T cells were transferred into CD45.2^+^ C57BL/6 recipients infected with *Cr* DBS100 (black) or *Cr*-gp66 (green). Colonic lamina propria (LP), pooled caudal-iliac lymph node (ciLN), distal mesenteric lymph node (dmLN) and spleen cells (day 14) were stained for CD45.1, CD45.2, CD4 and TCRβ and with L/D dye and analysed by flow cytometry. **e**, Number of CD45.1^+^ CD4 T cells from adoptively transferred mice on day 14 of infection with either *Cr* (DBS100; white) or *Cr*-gp66 (green) (3–5 mice per group; *n* = 2 experiments). **f**, SMARTA CD45.1^+^ and wild-type CD45.2^+^ CD4 T cells (1:1 ratio) transferred into *H2-Ab1*^*fl/fl*^ *Tcra*^–/–^ (blue) or *H2-Ab1*^*Villin*^*Tcra*^–/–^ (red) recipients infected with *Cr*-gp66. Colonic lamina propria cells from day 14 *Cr*-gp66 were stimulated with PMA and ionomycin, stained for surface markers as in **d**, then stained for intracellular IL-17A, IL-22 and IFNγ, and analysed by flow cytometry. **g**, Number of colonic IL-22^+^, IL-17A^+^, IL-17A^+^IFNγ^+^ and IFNγ^+^CD45.1^+^ CD4 T cells from day 14 *Cr*-gp66-infected mice (4–7 mice per group, *n* = 3 experiments). **h**,**i**, Colons from *H2-Ab1*^*fl/fl*^ and *H2-Ab1*^*Villin*^ mice on day 14 of infection with *Cr*-GFP were stained for pSTAT3, EPCAM1 and DAPI (**h**) or for *Cr*-GFP, EPCAM1 and DAPI (**i**) (3–5 mice per group; *n* = 2 experiments). Scale bars, 50 μm. Data are mean ± s.e.m. **e**,**g**, Two-tailed unpaired *t*-test. *P ≤ 0.05, **P ≤ 0.01 and ***P ≤ 0.001.

**Extended Data Fig. 8:**
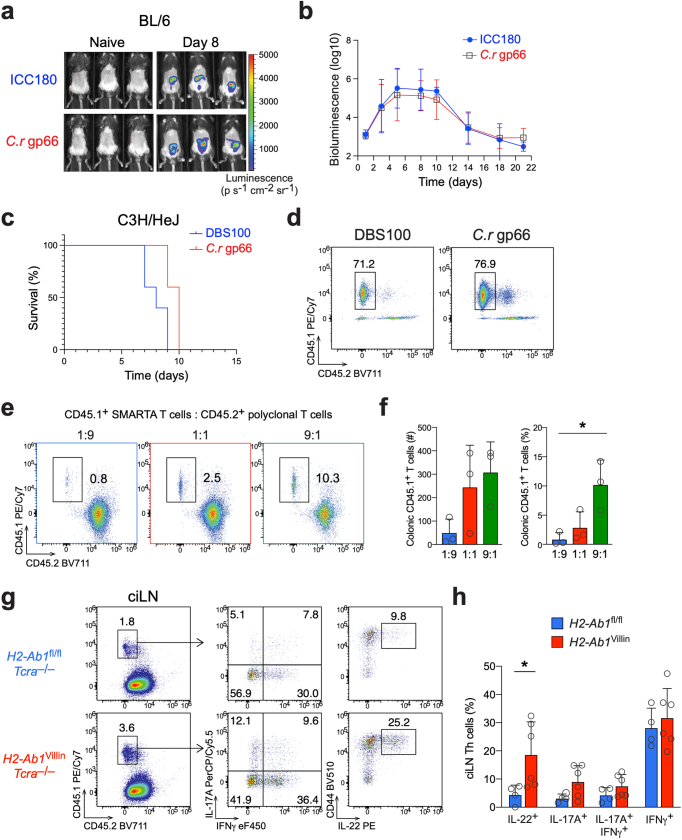
Characterization of antigen-specific T cell responses during *C.r* infection. **a**, Serial whole-body imaging and **b**, colonization kinetics of BL/6 mice infected with *C.r* (DBS100; blue) or *C.r* gp66 (red). 3–5 mice per group, n = 2 independent experiments. **c**, Survival kinetics of susceptible C3H/HeJ mice infected with either DBS100 (blue) or *C.r* gp66 (red). 5 mice per group, n = 1 experiment. **d**, Colon cells from *Tcra*^*–/–*^ mice infected with either *C.r* (DBS100) or *C.r* gp66 and adoptively transferred with CD45.1^+^ SMARTA CD4 T cells were stained for surface CD4, TCRβ, L/D dye, CD45.1 and CD45.2 and analyzed by flow cytometry. 4–6 mice per group, *n* = 1 experiment. **e**, Colon cells from *Tcra*^*–/–*^ mice infected with *C.r*-gp66 and adoptively transferred with different ratios of CD45.1^+^ CD4 T cells to CD45.2^+^ polyclonal CD4 T cells (1:9; blue, 1:1; red, 9:1; green) were stained for surface markers same as (d) and analyzed by flow cytometry. 3–5 mice per group, *n* = 2 independent experiments. **f**, Number and percent of CD45.1^+^ SMARTA CD4 T cells recovered after *C.r*-gp66 infection of mice receiving either 1:9 (blue), 1:1 (red) or 9:1 (green) ratio of CD45.1^+^ SMARTA to CD45.2^+^ polyclonal CD4 T cells. 3–5 mice per group, *n* = 2 independent experiments. One-way ANOVA; *p≤0.02. **g**, ciLN cells from d14 *C.r H2-Ab1*^fl/fl^.*Tcra*^–/–^ (blue) and *H2-Ab1*^Villin^.*Tcra*^–/–^ (red) mice adoptively transferred with CD45.1^+^ SMARTA and CD45.2^+^ polyclonal CD4 T cells (1:1) were stimulated for 4hrs with PMA/Ion and GolgiPlug, stained for surface markers same as (d), and then stained for intracellular IL-17, IL-22 and IFNγ and analyzed by flow cytometry. **h**, Frequency of ciLN IL-22^+^, IL-17A^+^, IL-17A^+^IFNγ^+^ and IFNγ^+^ T cells from d14 *C.r H2-Ab1*^fl/fl^.*Tcra*^–/–^ (blue) and *H2-Ab1*^Villin^.*Tcra*^–/–^ (red) mice transferred with CD45.1^+^ SMARTA cells and CD45.2^+^ polyclonal CD4 T cells (1:1). Two-way ANOVA; *p≤0.03. 4–6 mice per group, *n* = 2 independent experiments. Data are represented as mean ± SEM. ns=not significant. ciLN=pooled caudal and iliac lymph nodes.

In view of these results, we explored the possibility that non-classical antigen presentation by IECs may contribute to interactions with *Cr*-specific CD4 T cells. We engineered a novel *Cr* strain (*Cr*-gp66) expressing the lymphocytic choriomeningitis virus (LCMV) gp_66–80_ epitope recognized by the SMARTA T cell receptor (TCR) to enable T cell adoptive transfer studies. A minigene encoding the LCMV gp_66–80_ peptide and a haemagglutinin (HA) tag was introduced in-frame to the 3′ terminus of the *espZ* gene, which encodes a *Cr* effector protein that is injected into IECs to inhibit their death and prolong *Cr* colonization^48^ (Fig. 5a,b). Immunostaining of the HA tag in IECs infected with *Cr*-gp66, but not the wild-type *Cr* control strain (DBS100), identified punctate, cytosolic distribution of EspZ at sites of *Cr* attachment (Fig. 5c)—consistent with association of at least a fraction of EspZ with translocated intimin receptor^48^ (Tir). C57BL/6 mice infected with luminescent *Cr*-gp66 displayed similar colonization kinetics to a control luminescent *Cr* strain (ICC180) (Extended Data Fig. 8a,b), whereas susceptible C3H/HeJ mice succumbed similarly to infection with both engineered and control wild-type strains (Extended Data Fig. 8c), establishing that virulence was not attenuated by the introduction of LCMV gp_66–80_ and the HA tag.

SMARTA T cells transferred into C57BL/6 mice were recruited to the colon only in mice infected with *Cr*-gp66, and not the wild-type strain, despite similar population of lymphoid tissues in mice infected with either strain (Fig. 5d,e). To increase the number of *Cr*-specific T cells for analysis, we transferred increasing ratios of CD45.1^+^ SMARTA:CD45.2^+^ polyclonal T cells into T cell-deficient (*Tcra*^*–/–*^) mice^49^, resulting in cell dose-dependent recovery of colonic SMARTA T cells (Extended Data Fig. 8d–f). T cell-deficient mice with (*H2-Ab1*^*Villin*^*Tcra*^–/–^) or without (*H2-Ab1*^*fl/fl*^*Tcra*^–/–^) IEC MHCII deficiency were therefore populated with co-transfers of SMARTA:polyclonal T cells (1:1 ratio) to assess *Cr*-specific responses (Fig. 5f,g). In agreement with previous results (Fig. 4i), colons of mice lacking IEC-derived MHCII had significantly decreased SMARTA T cell subsets compared with wild-type littermates, including an approximately fivefold decrease in IL-22^+^ T cells (Fig. 5f,g). By contrast, lymph nodes draining distal colons of these mice showed a reciprocal increase of IL-22^+^ SMARTA T cells (Extended Data Fig. 8g,h). Thus, local retention and/or activation of *Cr*-specific T cell effectors in the infected colon is contingent on recognition of bacterial antigen injected into MHCII-expressing DCCs.

These findings suggested that without the ability to present *Cr* antigen to effector T cells, IECs are unable to recruit sufficient IL-22 to restrain *Cr* infection. Indeed, compared to controls, mice deficient for MHCII on IECs (*H2-Ab1*^*Villin*^) showed a substantial loss of STAT3 activation in both crypt-lining and superficial IECs during the late phase of *Cr* infection (Fig. 5h). This was accompanied by *Cr* invasion of colonic crypts in *H2-Ab1*^*Villin*^ mice but not in *H2-Ab1*^*fl/fl*^ controls (Fig. 5i), phenocopying the defects in STAT3 activation and crypt anti-bacterial defence reported in mice with T cell-specific IL-22 deficiency^4^. Collectively, these findings establish a requirement for MHCII-dependent antigen presentation of *Cr* antigen by colonic IECs to retain T_H_17 and T_H_22 cells and elicit IL-22-dependent help in defence against *Cr*.

## Discussion

A fundamental property of effector CD4 T cells is their provision of help to cells that express cognate antigen. Here we identify a subset of absorptive colonocytes that are the cellular hosts of *Cr* and show that these cells obtain epithelium-protective IL-22 via MHCII-restricted interactions with effector T cells of the T_H_17 pathway. Our findings address the longstanding conundrum of why *Cr* colonization is regionally restricted^6^: *Cr* attachment is primarily limited to the middle and distal colon owing to its tropism for DCCs. Our findings also reveal a basis for the indispensable role of T cells in protection of the intestinal barrier: T cells activated locally via non-classical antigen presentation by the intestinal epithelium are required to sustain high amplitude IL-22 signalling to IECs as they undergo developmental shifts in response to infection. Finally, our findings provide a basis for a requirement of coordinated signalling of IL-22 and IFNγ into IECs under pathogen threat. Because IFNγ is required to induce the MHCII APP pathway in IECs, it is prerequisite for protective actions of IL-22—or other signals that may be delivered directly to IECs by T cells. In essence, IFNγ signalling ‘licenses’ IECs for recruitment of enhanced IL-22 signalling from T_H_17 and T_H_22 cells, which is essential for barrier protection.

In contrast to *Cr*, which targets DCCs, ileal enterocytes are the infectious niche for enteropathogenic *E. coli* in humans^50^. The specific host molecules required for initial attachment of *Cr* or enteropathogenic *E. coli* to IECs remain unknown. Although firm attachment to IECs by both pathogens relies on intimin–Tir interactions, fimbriae and pili are thought to mediate the initial attachment to IECs via exposed d-mannose residues on glycoproteins^51^. However, intimin may also contribute to bacterial tropism. Whereas most enteropathogenic *E. coli* strains express intimin-α, *Cr* uses intimin-β^6,52^ Thus, in addition to species-specific differences in fimbriae and pili, sequence variation between bacterial intimin subtypes may contribute to host and tissue specificity. Our identification of an absorptive cell type that is targeted by *Cr* should facilitate comparative studies to define the basis for species- and cell-specific tropism of A/E enteropathogens.

Identification of subset-specific programming of colonocytes by IL-22 has implications for anti-bacterial defence strategies. Reg3 family defensins—originally implicated in antibacterial activity against *Cr*^1^—were restricted to PCCs, which are not targeted by *Cr*, suggesting indirect actions of these AMPs in pathogen resistance^40,53^. By contrast, DCCs upregulated genes such as *Lcn2*^54^, whose product binds bacterial siderophores to thwart iron sequestration from the host, and S100a8/9^55^ (calprotectin), a calcium/zinc-binding metal chelator that prevents bacterial uptake of metal ions during *Cr* infection. In addition, IL-22 stimulated DCCs to produce CXCL2 and CXCL5 to recruit neutrophils that themselves produce LCN2 and S100a8/9^56^, thereby amplifying local antimicrobial activity. Several DCC-specific genes involved in metal ion homeostasis (for example, *Ftl1* and *Slc40a1*) are downregulated during *Cr* infection^16^. The acquisition of metal ions by *Cr* (and *E. coli*) is a double-edged sword—although they benefit bacterial growth, their dysregulation can also generate toxic reactive oxygen species. Insights into how these mechanisms operate to favour host or pathogen should be facilitated by our findings.

*Cr* and other A/E enteropathogens must attach to survive. In addition to induction of AMPs and neutrophil-attractant chemokines, we identified programming of DCCs for accelerated shedding as an additional mechanism of host resistance, presumably to counter the actions of bacterial effector proteins injected into *Cr*-bound DCCs that would otherwise inhibit senescence and prolong survival of the cellular host of *Cr*^43^. The retention of pro-senescent DCCs in the absence of T cell-derived IL-22 is particularly striking, given that decreased shedding of colonized DCC cells increased bacterial load. Our findings indicate that IFNγ and IL-22 work in concert to accelerate production and removal of DCCs, respectively, thereby denying *Cr* a stable cellular platform for colonization. Because *Cr* lacks flagella and is therefore immotile^57^, its lateral spread to adjacent IECs from pioneering microcolonies requires host cell-to-cell migration, making accelerated clearance of infected colonocytes an effective host strategy to deny *Cr* purchase. In essence, by speeding up the ‘escalator’ of maturing DCCs as they move up crypts and off the luminal surface, coordinated actions of IFNγ and IL-22 restrain the lateral spread of *Cr* to prevent crypt invasion. Notably, however, deficiency of IL-22 alone results in the invasion of crypts^4^, indicating that, without the actions of IL-22 to promote accelerated shedding of *Cr*-laden DCCs—or other mechanisms driven by IL-22—IFNγ-driven hyperplasia is ineffectual at blunting crypt invasion. The ability to isolate *Cr*-DCC conjugates holds promise for elucidating competing strategies of pathogen and host to control survival of infected colonocytes.

The model system for tracking *Cr*-specific T cells introduced here will facilitate studies to address new questions raised by antigen recognition at the intestinal epithelium. For example, it will be of interest to determine whether bacterial peptides presented by IECs are derived exclusively from injected effector proteins, and by what pathway(s) *Cr* antigens are processed by IECs to load MHCII for T cell recognition. Further, it will be of interest to determine whether coordinated IL-22 and IFNγ signalling into IECs is supported by transdifferentiation of T_H_17 cells into T_H_1-like cells in the inflamed intestines—that is, whether the original speculation of a ‘two-for-one’ effector lineage ascribed to T_H_17^58^ reflects a mechanism for delivery of both IL-22 (or IL-17) and IFNγ to barrier epithelia by T cells bearing the same antigenic receptor. In addition to providing new insights into the importance of epithelial cell expression of MHCII APP machinery to host defence, our findings open a window into coordination of immune interactions by which A/E enteropathogens are resisted and identify a new absorptive enterocyte subset with which T cells can dialogue.

## Methods

### Mice

*Il22*^*hCD4.fl*^ reporter/floxed and *Il22*^*∆Tcell*^ cKO mice were previously generated within our laboratory^4^. C57BL/6 (wild type), C3H/HeJ, *H2-Ab1* floxed, *Cd4-*cre, SMARTA-1^59^ CD45.1, *Tcra*^–/–^, *Villin*-cre and *Villin*-cre/ERT2 mice were purchased from Jackson Laboratory. *H2-Ab1*^*Villin*^ (*H2-Ab1* floxed × *Villin*-cre) cKO mice were screened by PCR and flow cytometry to exclude mice with spontaneous, Cre-independent germline deletion of MHCII^60^. In most experiments, littermates were used as controls and experimental adult animals (8–12 wk old) were co-caged in groups of 2–7 mice. Male and female mice were used whenever possible. All mouse strains were bred and maintained at University of Alabama at Birmingham (UAB) in accordance with IACUC guidelines.

### *Cr* strains and infections

*Cr* strain, DBS100 (ATCC) was used for scRNA-seq experiments. For flow cytometry analysis and to track *Cr* *in situ*, we used a strain of *Cr* expressing GFP^61^ (derived from DBS100; provided by B. A. Vallance). For whole-body imaging experiments, we used the bioluminescent *Cr* strain ICC180^62^ (derived from DBS100; generously provided by G. Frankel and S. Wiles). A fresh, single colony was grown in 10 ml LB at 37 °C with rotation at 225 rpm for 12–14 h. Next day, 1 ml of overnight culture was added to 250 ml LB broth, incubated at 37 °C with rotation (225 rpm) for 3–3.5 h until OD_600_ reached 1.0 on spectrophotometer. Bacteria were pelleted at 25 °C, 3,000 rpm for 15 min and then resuspended in 5 ml sterile PBS. Mice were inoculated with 2 × 10^9^ CFU in a total volume of 100 µl of PBS by gastric gavage.

### Generation of *Cr*-*espZ*-gsg-gp66-HA strain by allelic exchange

DNA sequence containing a Gly-Ser-Gly (GSG) linker, LCMV glycoprotein residues 66–80 (DIYKGVYQFKSVEFD), and HA tag flanked by upstream and downstream regions of ~500 bp homologous to *Cr* (DBS100) *espZ* containing BamHI and SphI sites was synthesized by GeneArt (ThermoFisher) and then cloned into the pCAL52 shuttle vector containing *CmR*, *phoN* and *sacB* selection (a gift from A. Baumler) by T4 ligation. Ligation products were transformed into One Shot PIR2 *E. coli* (ThermoFisher) for propagation of the π-dependent R6Kγ origin of replication in pCAL52. Successfully ligated pCAL52 clones were sequence verified and grown overnight in LB broth at 37 °C. pCAL52 *Cr*-*espZ*-gsg-gp66-HA plasmid was purified from PIR2 *E. coli* using the QIAprep Spin Miniprep Kit (Qiagen). Purified plasmid was transformed into the EcNR1Δasd *E. coli* strain (a gift from M. Gray) that is auxotrophic for diaminopimelic acid (DAP) and selected for by chloramphenicol resistance on LB agar plates containing 25 μg ml^−1^ chloramphenicol and 50 μg ml^−1^ DAP for mating. Transformed EcNR1Δasd *E. coli* containing the pCAL52 *Cr*-*espZ*-gsg-gp66-HA plasmid was grown overnight in LB broth with 25 μg ml^−1^ chloramphenicol and 50 μg ml^−1^ DAP and *Cr* (DBS100 or ICC180) was grown overnight in LB broth. Puddle mating was performed by mixing 1.3 ml of overnight EcNR1Δasd *E. coli* pCAL52 *Cr*-*espZ*-gsg-gp66-HA culture and 200 μl of *Cr* (DBS100 or ICC180) and centrifugation followed by resuspension in 50 μl LB broth. This mixture was then spotted onto an LB agar plate containing 50 μg ml^−1^ DAP and incubated at 30 °C overnight to promote conjugation. The puddle mating was then collected into 1 ml of LB broth and 2 × 10^−6^ dilutions were plated on LB agar plates containing 25 μg ml^−1^ chloramphenicol (without DAP) and incubated at 37 °C overnight. Single-crossover merodiploid clones were confirmed by PCR and then selected in 25% sucrose LB broth for 3–4 days with serial passages of 5 × 10^−3^ dilutions into fresh 25% sucrose LB broth every 24 h. A volume of 50–100 μl of sucrose culture was plated every 24 h on LB agar containing 5-bromo-4-chloro-3-indolyl phosphate p-toluidine salt (BCIP) for blue-white detection of clones that had successfully undergone a double-crossover event to remove the *phoN*-containing pCAL52 backbone. White colonies were confirmed as either wild-type revertants or *espZ*-gsg-gp66-HA insertion mutants by PCR using the following forward and reverse primers: EspZ_F 5′-CGGGAATTGCAGCAATGTGT-3′ and EspZ_R 5′-GGTTGGGGCTAACGGAGTAT-3′.

### Isolation of IECs

Intestinal tissue was flushed with PBS, cut into regions (5 cm ileum, 4 cm mid–distal colon or 2 cm each for proximal, middle, or distal colon), opened longitudinally and then cut into strips of 1 cm length. Tissue pieces were incubated for 20 min at 37 °C with 1 mM DTT (Sigma), followed by 2 mM EDTA (Invitrogen) in H5H medium (1× HBSS, 5% FBS, 20 mM Hepes, and 2.5 mM β-mercaptoethanol). Tissue pieces were vortexed briefly after each 20 min incubation, followed by washing with H5H prior to centrifugation at 1,800 rpm for 10 min at 4 °C. IECs were then purified on a 40%/75% Percoll gradient by centrifugation for 20 min at 25 °C and 2,000 rpm with no brake. For analysis of *Cr*-GFP attached to IECs, tissue pieces from 4 cm of mid–distal colon were incubated for 20 min at 37 °C with 1 mM EDTA in H5H medium, followed by gentle mixing and washing with H5H.

### Colonic lamina propria CD4 T cell isolation

Colons were flushed with PBS, opened longitudinally then cut into small pieces and placed in H5H medium. Tissue was then minced in 1.5 ml microcentrifuge tubes for 2–3 min before being transferred into scintillation vials with 10 ml of complete R10 medium (1× RPMI 1640, 10% FBS, 1× penicillin/streptomycin, 1× nonessential amino acids, 1 mM sodium pyruvate, 2 mM l-glutamine, and 2.5 mM β-mercaptoethanol) with collagenase IV (Sigma, 100 U ml^−1^) and DNase I (Sigma, 20 mg ml^−1^). Tissue was digested at 37 °C for 40 min with stirring followed by filtering over a 70 μm filter and washing with complete R10 medium. Cells were then centrifuged at 1,750 rpm at 4 °C for 10 min and then purified on a 40%/75% Percoll as described above. Where indicated, cells were stimulated with PMA (50 ng ml^−1^) and ionomycin (750 ng ml^−1^) at 37 °C for 4 h in the presence of GolgiPlug (BD Biosciences).

### scRNA-seq and data analysis

Cells were isolated with DTT/EDTA (as described above) from mid–distal colons of naive (C57BL/6), and day 9 *Cr*-infected control (*Il22*^*hCD4*^) and T cell-specific IL-22 cKO (*Il22*^*∆Tcell*^) mice. Cells were pooled from 2–3 mice per condition. Live cells were sorted into 5 μl PBS with 5% FBS and 0.1 mM EDTA in an Eppendorf tube using BD Aria II. Prior to loading on the 10x Genomics Chromium instrument, cells were counted using a haemocytometer and viability of at least 90% for all samples were confirmed by trypan blue staining for a targeted number of 10,000 live cells. scRNA-seq libraries were prepared using the Chromium Single Cell 3′ Library Kit (10x Genomics) at the FCSC Core Facility at UAB. Libraries were sequenced on an Illumina NovaSeq 6000 at the Sequencing Core Facility at La Jolla Institute, CA. Cell counts were produced by Cell Ranger pipelines and transformed into Seurat objects (see ‘Extended scRNA-seq and data analysis’). Data are publicly available at the Gene Expression Omnibus GEO under accession GSE227331.

### Extended scRNA-seq and data analysis

#### Processing of raw scRNA-seq data

Raw sequencing data were processed with the Cell Ranger pipeline software (v.3.0.2; 10x Genomics). Raw base call files generated by Novaseq 6000 (Illumina) were converted to FASTQ files using cellranger mkfastq with default parameters. The Cell Ranger count pipeline was used to perform quality control, sample demultiplexing, barcode processing, alignment and single-cell 5′ gene counting. Cell ranger count was used to align raw reads against the GRCm38 mouse reference transcriptome. Subsequently, cell barcodes and unique molecular identifiers underwent filtering and correction using default parameters in Cell Ranger. Reads with the retained barcodes were quantified and used to build the gene-expression matrix.

#### Classification of IECs using single-cell transcriptome data

Seurat^63^ (v.3.0.0), implemented using the R package, was applied to exclude low-quality cells. Cells that expressed fewer than 200 genes were filtered out. Genes that were not detected in at least three single cells were excluded. EPCAM1^+^ cells were selected and based on these criteria, we retained the total numbers of IECs per genotype: 5,064 cells from naive C57BL/6, 6,081 cells from day 9 *Cr*-infected *Il22*^*hCD4*^ (control) and 4,416 cells from day 9 *Cr*-infected *Il22*^*∆Tcell*^ pooled samples. The processed data was normalized using Seurat’s NormalizeData function, which used a global scaling normalization method, LogNormalize, to normalize the gene-expression measurements for each cell to the total gene expression. Highly variable genes were then identified using the function FindVariableGenes in Seurat. The anchors were identified using the FindIntegrationAnchors function, and thus the matrices from different samples were integrated with the IntegrateData function. The variation arising from library size and percentage of mitochondrial genes was regressed out using the function ScaleData in Seurat. Principal component analysis was performed using the Seurat function RunPCA, and a *k*-nearest neighbour graph was constructed using the FindNeighbors function in Seurat with the number of significant principal components identified from principal component analysis. Clusters were identified using FindClusters function with resolution of 0.8. The clusters were visualized in two dimensions with UMAP. The normalization, integration, and clustering were performed under standard Seurat workflow.

#### Identification of differentially expressed genes

Differential gene-expression analyses were carried out using the Seurat function FindMarkers. In brief, we performed the Wilcoxon rank-sum test with the default threshold of 0.25 for log_2_ fold change and a filter for the minimum percent of cells in a cluster greater than 25%. The differentially expressed genes were isolated by comparing significantly upregulated genes and downregulated genes defined as adjusted *P* value (*P*_adj_) <0.05. Top differentially expressed genes were visualized by heat map via ComplexHeatmap (v2.11.1) package in R. Expression of individual differentially expressed genes were represented as violin plots. Violin plots were rendered using the function VlnPlot in Seurat. The non-parametric Wilcoxon rank-sum test with adjusted *P* value < 0.05, based on Bonferroni correction method was used for differential gene-expression analyses.

#### Gene set enrichment analysis

The fgsea R package^64^ (v1.4.0) was used for gene set enrichment. Gene sets used were Gene Ontology Biological Process gene sets. The input for gene set enrichment analysis was a set of gene signatures of a cluster or cell type obtained from Seurat. A *P* value quantifying the likelihood that a given gene set displays the observed level of enrichment for genes was calculated using fast gene set enrichment analysis (fgsea, v1.4.0) with 1 million permutations. Gene set enrichment *P* values of normalized enrichment scores were corrected with the Benjamini-Hochberg procedure^65^. The top enriched terms were visualized with dot plots using R package ggplot2 (v3.3.5).

#### Trajectory analysis

RNA velocity analysis was conducted using the scVelo package (v0.2.2) with Scanpy (v1.6.1) on Python (v3.8.5). To perform trajectory analysis, the un-spliced and spliced variant count matrix that was calculated using 10× pipeline in velocyto (v0.17.16) was fused with an anndata object containing the UMAP information and cluster identities defined in Seurat analysis. The combined dataset was then processed using the scVelo pipeline: The ratio of un-spliced:spliced RNA for each gene was filtered and normalized using the default settings. Afterward, the first and second moments were calculated for velocity estimation. Following moment calculation, the dynamic model was used to calculate the RNA velocities. The dynamic model iteratively estimates the parameters that best model the phase trajectory of each gene, therefore capturing the most accurate, albeit more computationally intensive, estimate of the dynamics for each gene. These approaches were used to graphically model the RNA velocity for each condition.

### Tissue and cell preparation

Tissues were fixed in cold 4% paraformaldehyde overnight at 4 °C. The next day, tissue was rinsed in cold 1× PBS for several washes including an overnight incubation at 4 °C. Third day, tissue was placed in cold 30% sucrose in 1× PBS overnight at 4 °C. Tissue was embedded in OCT (Tissue-Tek) and frozen with 2-methyl butane chilled with liquid nitrogen. For pSTAT3 staining, tissue sections were permeabilized in cold methanol (Fisher) for 10–15 min at −20 °C. Tissue sections were blocked at room temperature for 30 min with 10% mouse serum in 1× PBS and 0.05% Tween-20. Antibodies were diluted in 2% BSA/PBS/Tween-20 and incubated for 20–30 min at room temperature or overnight at 4 °C (pSTAT3 stain). For BrdU experiments, mice were given 100 mg BrdU per kg intraperitoneally on day 9 of *Cr* infection, colon tissue was collected and fixed at various time points after BrdU administration. Tissue was incubated with 0.025 M HCl followed by 0.1 M borate buffer, pH 8.5 to permeabilize and neutralize the tissue prior to staining as described above. Sorted epithelial cells (2–5 × 10^5^ cells/ml) were cytospun onto glass slides at 1,500 rpm for 2 min at room temperature. Cells were then dried for 10 min, fixed with 4% paraformaldehyde for 10 min and then permeabilized with cold methanol for 10 minutes prior to staining. The following antibodies and reagents were used: anti-BrdU (BU1/75; Abcam), anti-EPCAM1 (G8.8; ThermoFisher), anti-FABP2 (polyclonal; R&D/Fisher), anti-GFP (A-11122; ThermoFisher), anti-goat IgG (ThermoFisher), anti-HA tag (polyclonal; Fisher/Novus Biologicals), anti-LY6G (1A8; Biolegend or ThermoFisher), anti-rabbit IgG (ThermoFisher), Streptavidin-Alexa Fluor 594, UEA-1 (Vector Labs) and Prolong Diamond antifade mountant with DAPI (ThermoFisher).

### Flow cytometry

Colon cells were stained with Fc Block (Clone 2.4G2) followed by staining with fluorescently labelled antibodies in IEC buffer (PBS with 5% FBS and 2 mM EDTA to reduce cell clumping) for IECs or 2% FBS in PBS for T cells on ice in 1.5 ml microcentrifuge tubes. For intracellular staining, cells were fixed and permeabilized using BD Cytofix/Cytoperm kit (BD Bioscience). Samples were acquired on an Attune NxT flow cytometer (Life Technologies) and analysed with FlowJo software. Cells were sorted on either a BD ARIA II or S6 Enceladus (BD Biosciences). The following antibodies/reagents were used: anti-CD45 (30-F11; Biolegend), anti-EPCAM1 (G8.8; ThermoFisher), anti-FABP2 (polyclonal; R&D/Fisher), anti-goat IgG (ThermoFisher), anti-LY6G (1A8; BioLegend), anti-MHCII (M5/114.15.2; Fisher), Live/Dead Fixable Near-IR dead cell dye (ThermoFisher), anti-CD4 (RM4-5; BioLegend), anti-TCRβ (Η57−597; ThermoFisher), anti-CD44 (IM7; BioLegend), anti-CD45.1 (A20; ThermoFisher), anti-CD45.2 (104; BD Biosciences), anti-IL-17A (TC11-18H10; BD Biosciences), anti-IFNγ (XMG1.2; ThermoFisher), and anti-IL-22 (1H8PWSR; ThermoFisher).

### Real Time PCR

cDNA synthesis was performed with iScript Reverse Transcription Supermix (Bio-Rad) according to manufacturer’s instructions. cDNA amplification was analysed with SsoAdvanced Universal SYBR Green Supermix (Bio-Rad) in a Biorad CFX qPCR instrument. See Supplementary Table 8 for the list of primer sequences.

### Colony counts of *Cr*-GFP

Faeces were collected, weighed, and dispersed for 30 s in PBS using a PowerGen500 homogenizer (Fisher). Liver was removed under sterile conditions, placed in 2–3 ml H5H in Miltenyi M tubes, weighed, and homogenized using Miltenyi GentleMACS Dissociator using Program RNA_01. Homogenate was filtered through a 70-μm cell strainer and then spun at 8,000 rpm for 15 min to pellet cells. Cell pellet was resuspended in PBS and serially diluted and plated in duplicate on LB plates containing 30 μg ml^−1^ chloramphenicol. Colonies were counted after 12 h incubation at 37 °C to determine the log_10_ CFU per gram of tissue.

#### Bioluminescence Imaging

Mice were anaesthetized with a VetFlo isoflurane vapourizer and then shaved, chemically depilated, and placed in a supine position in a custom-built chamber for imaging with IVIS Lumina III system and analysis with Living Image Software (PerkinElmer) in the UAB Small Animal Imaging Shared Facility (supported by S10 instrumentation grant 1S10OD021697). Baseline images were collected prior to gavage with 2 × 10^9^ CFU *Cr* ICC180 strain. Images were collected from mice every 2 days beginning day 1 post-infection through day 34 post-infection. Where indicated, mice were administered 100 μl tamoxifen (20 mg ml^−1^ in corn oil) intraperitoneally on days 5–12 post-infection.

#### Adoptive transfer experiments

Spleens and lymph nodes from SMARTA-1 CD45.1^+^ donors were collected and dissociated in complete R10 medium prior to centrifugation at 1,500 rpm for 5 min at 4 C. Red blood cells were removed from the cell suspension by ACK lysis for 2 min prior to quenching with complete R10 medium. Naive CD4^+^ T cells from pooled spleens and lymph nodes were purified using the naive CD4^+^ mouse T cell Isolation kit per manufacturer’s instructions (Miltenyi Biotec). Purified T cells were transferred by intravenous injection (1 × 10^6^ total cells) into recipients, followed by *Cr* infection on the same day. Where indicated, 5 × 10^5^ SMARTA^+^CD45.1^+^ and 5 × 10^5^ wild-type CD45.2^+^ CD4 T cells (1 × 10^6^ total cells; 1:1 ratio) were transferred into recipient mice.

#### 16 S rRNA microbiota sequencing and analysis

Fecal samples from individual mice were collected and frozen at −80 °C. Fecal DNA isolation, 16 S rRNA V4 region PCR amplification, and sequencing on Illumina MiSeq was performed following previously established methods at the UAB Microbiome Institutional Research Core^66,67^. Sequencing data quality control, read mapping, and amplicon sequence variant (ASV) generation was completed using QIIME^67–69^. Taxonomy classification was completed using the SILVA database^70^.

### Reporting summary

Further information on research design is available in the Nature Portfolio Reporting Summary linked to this article.

## Online content

Any methods, additional references, Nature Portfolio reporting summaries, source data, extended data, supplementary information, acknowledgements, peer review information; details of author contributions and competing interests; and statements of data and code availability are available at 10.1038/s41586-024-07288-1.

### Supplementary information

Supplementary Tables Supplementary Tables 1–8.

Reporting Summary

## Data Availability

The scRNA-seq data used in this study have been deposited in the Gene Expression Omnibus database under the accession number GSE227331.

## Code availability

The shell, R and Python scripts that enabled the main steps of the analyses performed in this project are available on request.
