# Supplementary material for: Distal colonocytes targeted by C. rodentium recruit T-cell help for barrier defence
Source: Nature. 2024 Apr 10;629(8012):669–78. doi: 10.1038/s41586-024-07288-1 (PMC11096101; doi:10.1038/s41586-024-07288-1)
Supplement: Supplementary file 1 — Supplementary Tables 1–8. [file 41586_2024_7288_MOESM1_ESM.pdf]

nature portfolio

https://doi.org/10.1038/s41586-024-07288-1

Supplementary information

Distal colonocytes targeted by *C. rodentium* recruit T-cell help for barrier defence

In the format provided by the authors and unedited

Supplementary Table 1. Genes expressed by human ascending colon epithelial cells and mouse mature PCCs compared to human sigmoid colon epithelial cells and mouse mature DCCs.

**Human ascending colon (AC) ECs**	**p_val**	**avg_log2FC**	**pct.1 (AC)**	**pct.2 (SC)**	**p_val_adj**
FABP5	0	1.241903	0.743	0.573	0
ACTB	3.27E-271	0.596254	0.991	0.981	6.02E-267
SSFA2	4.10E-67	0.662749	0.51	0.453	7.55E-63
SLC9a3	7.69E-44	0.762939	0.213	0.136	1.42E-39

**Mouse Mature PCCs**	**p_val**	**avg_log2FC**	**pct.1 (PCC)**	**pct.2 (DCC)**	**p_val_adj**
Fabp2	3.61E-179	2.750772	0.657	0.036	6.68E-175
Actb	5.24E-39	0.631615	1	0.998	9.69E-35
Ssfa2	5.30E-43	0.972053	0.768	0.431	9.80E-39
Slc9a3	2.19E-23	0.41261	0.126	0.013	4.06E-19

**Human sigmoid colon (SC) ECs**	**p_val**	**avg_log2FC**	**pct.1 (AC)**	**pct.2 (SC)**	**p_val_adj**
SELENOP	5.11E-153	-0.919802	0.232	0.443	9.41E-149
HOXB13	0	-0.552783	0	0.289	0
CA4	3.26E-107	-1.051605	0.265	0.436	6.00E-103
SLC26A2	4.36E-10	-0.526863	0.609	0.652	8.03E-06
AOC1	0	-1.001982	0.305	0.646	0
WFDC2	1.45E-240	-0.80081	0.202	0.486	2.67E-236
NDUFA13	0	-0.98495	0.364	0.761	0
EFNA1	8.78E-165	-0.557076	0.261	0.506	1.62E-160
LSR	0	-0.773105	0.378	0.701	0
GSN	5.07E-226	-1.408427	0.323	0.594	9.34E-222

**Mouse Mature DCCs**	**p_val**	**avg_log2FC**	**pct.1 (PCC)**	**pct.2 (DCC)**	**p_val_adj**
Sepp1	3.74E-100	-1.121497	0.94	0.998	6.92E-96
Hoxb13	3.07E-66	-1.255158	0.437	0.796	5.67E-62
Car4	6.28E-53	-0.680782	1	1	1.16E-48
Slc26a2	3.53E-42	-0.762159	0.836	0.956	6.53E-38
Slc26a3	9.65E-22	-0.625526	0.78	0.906	1.78E-17
Aoc1	3.82E-20	-0.794967	0.213	0.429	7.06E-16
Wfdc2	3.82E-17	-0.689517	0.437	0.599	7.06E-13
Ndufa13	3.67E-14	-0.551289	0.691	0.775	6.80E-10
Efna1	2.83E-10	-0.543387	0.657	0.705	5.23E-06
Lsr	1.94E-08	-0.474636	0.123	0.236	0.00036
Gsn	2.23E-06	-0.400776	0.454	0.515	0.041229

Supplementary Table 2. Top expressed genes per cluster.

Cluster	Genes >											
Lgr5-hi	*Lgr5*	*Smoc2*	*Rgcc*	*Trf*	*Acot1*	*Ascl2*	*Cdca7*	*Stmn1*	*Ifitm3*			
Lgr5-dim	*Hells*	*Slfn9*	*Uhrf1*	*Clspn*	*Exo1*	*Tcf19*	*Top2a*					
Early TA cells	*Nusap1*	*Kif11*	*Kif20b*	*Prc1*	*Cdk1*	*Plk1*	*Mki67*					
Absorptive Progenitor	*Ube2c*	*Cdc20*	*Birc5*	*Tpx2*	*Cenpa*	*Cdkn3*	*Cdca3*					
Pre-DCC	*Ly6a*	*Lrg1*	*Ly6c1*	*Lbp*	*Cldn8*	*Hao2*	*Gsdmc4*	*Aqp4*				
Pre-MCC	*Aqp8*	*Fabp2*	*Sptssb*	*Reg3b*	*Reg3g*	*Apol10a*	*Higd1a*	*Mgat4c*	*Car1*			
Pro-DCC	*Tnni1*	*Slc37a2*	*Fxyd4*	*Sycn*	*Saa1*	*Ugdh*	*Eno3*					
Mature DCC	*Car4*	*Dmbt1*	*Tgm3*	*Ly6g*	*Slc20a1*	*Slc26a3*	*Cyp2d9*	*Sepp1*				
Mature MCC	*Emp1*	*Dpep1*	*Guca2a*	*Ndrg1*	*Ncoa7*	*Cyp2c55*	*Clca4a*	*Muc3*				
Pathogen-induced CC	*S100a8*	*Ido1*	*Ubd*	*Cxcl2*	*Cxcl5*	*Nos2*	*Xdh*	*Gbp2*	*Tgm2*	*Tnf*	*Cd274*	*Adrb2*
Pro-Senescent CC	*Lars2*	*Atp12a*	*Tnfaip3*	*Irf1*	*Kif13b*	*Tle4*	*Mprip*	*Arap2*	*Cdk12*	*Myo15b*	*Gm26917*	*Gm42418*
Secretory Progenitor	*Atoh1*	*Txndc5*	*Guca2b*	*Dll1*	*Cbfa2t3*	*Plpp1*	*Slc12a8*	*Creb3l4*				
Paneth-like cells	*Agr2*	*Spink4*	*Reg4*	*Mptx1*	*Retnlb*	*Ramp1*	*Nov*					
Goblet cells	*Zg16*	*Clca1*	*Fcgbp*	*Tff3*	*Muc2*	*Spink1*	*Fer1l6*					
EEC	*Chga*	*Tph1*	*Neurod1*	*Snap25*	*Pyy*	*Sct*	*Pcsk1n*					
Tuft	*Matk*	*Dclk1*	*Trpm5*	*Ly6g6f*	*Dnah5*	*Alox5*						

Supplementary Table 3. One-way ANOVA of DCC lineage genes shown in Figure ED1e.

Gene	Tukey's multiple comparisons test	Mean Diff.	95.00% CI of diff.	Below threshold?	Summary	Adjusted P Value
Ly6g	Lgr5-hi vs. Lgr5-dim	-0.1729	-1.463 to 1.117	No	ns	>0.9999
Ly6g	Lgr5-hi vs. Early TA	0.04906	-1.712 to 1.810	No	ns	>0.9999
Ly6g	Lgr5-hi vs. Sec Prog	-0.1058	-1.101 to 0.8895	No	ns	>0.9999
Ly6g	Lgr5-hi vs. Paneth-like	0.06723	-0.8729 to 1.007	No	ns	>0.9999
Ly6g	Lgr5-hi vs. Goblet	-0.07483	-0.9004 to 0.7507	No	ns	>0.9999
Ly6g	Lgr5-hi vs. Endocrine	0.1420	-1.438 to 1.722	No	ns	>0.9999
Ly6g	Lgr5-hi vs. Tuft	0.09216	-1.267 to 1.452	No	ns	>0.9999
Ly6g	Lgr5-hi vs. Abs Prog	-0.4349	-2.074 to 1.204	No	ns	>0.9999
Ly6g	Lgr5-hi vs. Pre-DCC	-0.7913	-1.836 to 0.2535	No	ns	0.4013
Ly6g	Lgr5-hi vs. Pre-PCC	-2.832	-4.100 to -1.564	Yes	****	<0.0001
Ly6g	Lgr5-hi vs. Pro-DCC	-11.09	-11.95 to -10.23	Yes	****	<0.0001
Ly6g	Lgr5-hi vs. Mature DCC	-5.890	-6.667 to -5.114	Yes	****	<0.0001
Ly6g	Lgr5-hi vs. Mature PCC	-1.299	-2.208 to -0.3897	Yes	***	0.0001
Ly6g	Lgr5-hi vs. P-I CC	-3.769	-5.962 to -1.575	Yes	****	<0.0001
Ly6g	Lgr5-hi vs. Pro-Senescent CC	-1.317	-2.243 to -0.3908	Yes	***	0.0001
Ly6g	Lgr5-dim vs. Early TA	0.2219	-1.722 to 2.166	No	ns	>0.9999
Ly6g	Lgr5-dim vs. Sec Prog	0.06713	-1.224 to 1.359	No	ns	>0.9999
Ly6g	Lgr5-dim vs. Paneth-like	0.2401	-1.009 to 1.490	No	ns	>0.9999
Ly6g	Lgr5-dim vs. Goblet	0.09805	-1.068 to 1.264	No	ns	>0.9999
Ly6g	Lgr5-dim vs. Endocrine	0.3149	-1.467 to 2.096	No	ns	>0.9999
Ly6g	Lgr5-dim vs. Tuft	0.2650	-1.324 to 1.854	No	ns	>0.9999
Ly6g	Lgr5-dim vs. Abs Prog	-0.2620	-2.096 to 1.572	No	ns	>0.9999
Ly6g	Lgr5-dim vs. Pre-DCC	-0.6185	-1.949 to 0.7117	No	ns	0.9697
Ly6g	Lgr5-dim vs. Pre-PCC	-2.659	-4.171 to -1.148	Yes	****	<0.0001
Ly6g	Lgr5-dim vs. Pro-DCC	-10.92	-12.11 to -9.727	Yes	****	<0.0001
Ly6g	Lgr5-dim vs. Mature DCC	-5.717	-6.849 to -4.586	Yes	****	<0.0001
Ly6g	Lgr5-dim vs. Mature PCC	-1.126	-2.352 to 0.1005	No	ns	0.1150
Ly6g	Lgr5-dim vs. P-I CC	-3.596	-5.939 to -1.253	Yes	****	<0.0001
Ly6g	Lgr5-dim vs. Pro-Senescent CC	-1.144	-2.383 to 0.09495	No	ns	0.1093
Ly6g	Early TA vs. Sec Prog	-0.1548	-1.917 to 1.607	No	ns	>0.9999
Ly6g	Early TA vs. Paneth-like	0.01818	-1.713 to 1.750	No	ns	>0.9999
Ly6g	Early TA vs. Goblet	-0.1239	-1.796 to 1.548	No	ns	>0.9999
Ly6g	Early TA vs. Endocrine	0.09297	-2.054 to 2.240	No	ns	>0.9999
Ly6g	Early TA vs. Tuft	0.04310	-1.947 to 2.034	No	ns	>0.9999
Ly6g	Early TA vs. Abs Prog	-0.4840	-2.675 to 1.707	No	ns	>0.9999
Ly6g	Early TA vs. Pre-DCC	-0.8404	-2.631 to 0.9500	No	ns	0.9671
Ly6g	Early TA vs. Pre-PCC	-2.881	-4.810 to -0.9522	Yes	****	<0.0001
Ly6g	Early TA vs. Pro-DCC	-11.14	-12.83 to -9.449	Yes	****	<0.0001
Ly6g	Early TA vs. Mature DCC	-5.939	-7.587 to -4.291	Yes	****	<0.0001
Ly6g	Early TA vs. Mature PCC	-1.348	-3.063 to 0.3669	No	ns	0.3342
Ly6g	Early TA vs. P-I CC	-3.818	-6.449 to -1.186	Yes	****	<0.0001
Ly6g	Early TA vs. Pro-Senescent CC	-1.366	-3.090 to 0.3577	No	ns	0.3199
Ly6g	Abs Prog vs. Pre-DCC	-0.3564	-2.027 to 1.314	No	ns	>0.9999
Ly6g	Abs Prog vs. Pre-PCC	-2.397	-4.216 to -0.5786	Yes	***	0.0007
Ly6g	Abs Prog vs. Pro-DCC	-10.65	-12.21 to -9.093	Yes	****	<0.0001
Ly6g	Abs Prog vs. Mature DCC	-5.455	-6.973 to -3.938	Yes	****	<0.0001
Ly6g	Abs Prog vs. Mature PCC	-0.8639	-2.453 to 0.7256	No	ns	0.8925
Ly6g	Abs Prog vs. P-I CC	-3.334	-5.886 to -0.7820	Yes	***	0.0008
Ly6g	Abs Prog vs. Pro-Senescent CC	-0.8822	-2.482 to 0.7171	No	ns	0.8805
Ly6g	Pre-DCC vs. Pre-PCC	-2.041	-3.350 to -0.7323	Yes	****	<0.0001
Ly6g	Pre-DCC vs. Pro-DCC	-10.30	-11.21 to -9.380	Yes	****	<0.0001
Ly6g	Pre-DCC vs. Mature DCC	-5.099	-5.940 to -4.258	Yes	****	<0.0001
Ly6g	Pre-DCC vs. Mature PCC	-0.5075	-1.472 to 0.4575	No	ns	0.9159
Ly6g	Pre-DCC vs. P-I CC	-2.977	-5.195 to -0.7601	Yes	***	0.0005
Ly6g	Pre-DCC vs. Pro-Senescent CC	-0.5258	-1.507 to 0.4554	No	ns	0.9031
Ly6g	Pre-PCC vs. Pro-DCC	-8.256	-9.422 to -7.091	Yes	****	<0.0001
Ly6g	Pre-PCC vs. Mature DCC	-3.058	-4.164 to -1.952	Yes	****	<0.0001
Ly6g	Pre-PCC vs. Mature PCC	1.533	0.3304 to 2.737	Yes	**	0.0014
Ly6g	Pre-PCC vs. P-I CC	-0.9365	-3.267 to 1.394	No	ns	0.9926

Ly6g	Pre-PCC vs. Pro-Senescent CC	1.515	0.2990 to 2.731	Yes	**	0.0021
Ly6g	Pro-DCC vs. Mature DCC	5.199	4.605 to 5.792	Yes	****	<0.0001
Ly6g	Pro-DCC vs. Mature PCC	9.790	9.031 to 10.55	Yes	****	<0.0001
Ly6g	Pro-DCC vs. P-I CC	7.320	5.184 to 9.456	Yes	****	<0.0001
Ly6g	Pro-DCC vs. Pro-Senescent CC	9.772	8.992 to 10.55	Yes	****	<0.0001
Ly6g	Mature DCC vs. Mature PCC	4.591	3.926 to 5.256	Yes	****	<0.0001
Ly6g	Mature DCC vs. P-I CC	2.121	0.01726 to 4.226	Yes	*	0.0457
Ly6g	Mature DCC vs. Pro-Senescent CC	4.573	3.885 to 5.261	Yes	****	<0.0001
Ly6g	Mature PCC vs. P-I CC	-2.470	-4.627 to -0.3133	Yes	**	0.0086
Ly6g	Mature PCC vs. Pro-Senescent CC	-0.01836	-0.8536 to 0.8169	No	ns	>0.9999
Ly6g	P-I CC vs. Pro-Senescent CC	2.452	0.2876 to 4.616	Yes	*	0.0101
Ly6g	Sec Prog vs. Paneth-like	0.1730	-0.7690 to 1.115	No	ns	>0.9999
Ly6g	Sec Prog vs. Goblet	0.03093	-0.7968 to 0.8586	No	ns	>0.9999
Ly6g	Sec Prog vs. Endocrine	0.2478	-1.333 to 1.829	No	ns	>0.9999
Ly6g	Sec Prog vs. Tuft	0.1979	-1.163 to 1.559	No	ns	>0.9999
Ly6g	Sec Prog vs. Abs Prog	-0.3292	-1.969 to 1.311	No	ns	>0.9999
Ly6g	Sec Prog vs. Pre-DCC	-0.6856	-1.732 to 0.3610	No	ns	0.6644
Ly6g	Sec Prog vs. Pre-PCC	-2.727	-3.996 to -1.457	Yes	****	<0.0001
Ly6g	Sec Prog vs. Pro-DCC	-10.98	-11.84 to -10.12	Yes	****	<0.0001
Ly6g	Sec Prog vs. Mature DCC	-5.784	-6.563 to -5.006	Yes	****	<0.0001
Ly6g	Sec Prog vs. Mature PCC	-1.193	-2.104 to -0.2820	Yes	***	0.0008
Ly6g	Sec Prog vs. P-I CC	-3.663	-5.857 to -1.469	Yes	****	<0.0001
Ly6g	Sec Prog vs. Pro-Senescent CC	-1.211	-2.140 to -0.2831	Yes	***	0.0009
Ly6g	Paneth-like vs. Goblet	-0.1421	-0.9026 to 0.6184	No	ns	>0.9999
Ly6g	Paneth-like vs. Endocrine	0.07480	-1.472 to 1.622	No	ns	>0.9999
Ly6g	Paneth-like vs. Tuft	0.02493	-1.296 to 1.346	No	ns	>0.9999
Ly6g	Paneth-like vs. Abs Prog	-0.5022	-2.110 to 1.105	No	ns	0.9996
Ly6g	Paneth-like vs. Pre-DCC	-0.8586	-1.853 to 0.1357	No	ns	0.1865
Ly6g	Paneth-like vs. Pre-PCC	-2.899	-4.126 to -1.673	Yes	****	<0.0001
Ly6g	Paneth-like vs. Pro-DCC	-11.16	-11.95 to -10.36	Yes	****	<0.0001
Ly6g	Paneth-like vs. Mature DCC	-5.957	-6.664 to -5.251	Yes	****	<0.0001
Ly6g	Paneth-like vs. Mature PCC	-1.366	-2.217 to -0.5155	Yes	****	<0.0001
Ly6g	Paneth-like vs. P-I CC	-3.836	-6.006 to -1.666	Yes	****	<0.0001
Ly6g	Paneth-like vs. Pro-Senescent CC	-1.384	-2.253 to -0.5155	Yes	****	<0.0001
Ly6g	Goblet vs. Endocrine	0.2169	-1.263 to 1.697	No	ns	>0.9999
Ly6g	Goblet vs. Tuft	0.1670	-1.075 to 1.409	No	ns	>0.9999
Ly6g	Goblet vs. Abs Prog	-0.3601	-1.903 to 1.183	No	ns	>0.9999
Ly6g	Goblet vs. Pre-DCC	-0.7165	-1.603 to 0.1702	No	ns	0.2868
Ly6g	Goblet vs. Pre-PCC	-2.757	-3.899 to -1.616	Yes	****	<0.0001
Ly6g	Goblet vs. Pro-DCC	-11.01	-11.67 to -10.36	Yes	****	<0.0001
Ly6g	Goblet vs. Mature DCC	-5.815	-6.361 to -5.270	Yes	****	<0.0001
Ly6g	Goblet vs. Mature PCC	-1.224	-1.946 to -0.5022	Yes	****	<0.0001
Ly6g	Goblet vs. P-I CC	-3.694	-5.817 to -1.571	Yes	****	<0.0001
Ly6g	Goblet vs. Pro-Senescent CC	-1.242	-1.986 to -0.4989	Yes	****	<0.0001
Ly6g	Endocrine vs. Tuft	-0.04987	-1.882 to 1.782	No	ns	>0.9999
Ly6g	Endocrine vs. Abs Prog	-0.5769	-2.625 to 1.471	No	ns	0.9999
Ly6g	Endocrine vs. Pre-DCC	-0.9334	-2.546 to 0.6793	No	ns	0.8345
Ly6g	Endocrine vs. Pre-PCC	-2.974	-4.740 to -1.209	Yes	****	<0.0001
Ly6g	Endocrine vs. Pro-DCC	-11.23	-12.73 to -9.732	Yes	****	<0.0001
Ly6g	Endocrine vs. Mature DCC	-6.032	-7.485 to -4.579	Yes	****	<0.0001
Ly6g	Endocrine vs. Mature PCC	-1.441	-2.969 to 0.08746	No	ns	0.0906
Ly6g	Endocrine vs. P-I CC	-3.911	-6.425 to -1.397	Yes	****	<0.0001
Ly6g	Endocrine vs. Pro-Senescent CC	-1.459	-2.998 to 0.07941	No	ns	0.0857
Ly6g	Tuft vs. Abs Prog	-0.5271	-2.411 to 1.357	No	ns	0.9999
Ly6g	Tuft vs. Pre-DCC	-0.8835	-2.281 to 0.5140	No	ns	0.7205
Ly6g	Tuft vs. Pre-PCC	-2.924	-4.496 to -1.353	Yes	****	<0.0001
Ly6g	Tuft vs. Pro-DCC	-11.18	-12.45 to -9.917	Yes	****	<0.0001
Ly6g	Tuft vs. Mature DCC	-5.982	-7.192 to -4.772	Yes	****	<0.0001
Ly6g	Tuft vs. Mature PCC	-1.391	-2.690 to -0.09183	Yes	*	0.0222
Ly6g	Tuft vs. P-I CC	-3.861	-6.243 to -1.479	Yes	****	<0.0001

Ly6g	Tuft vs. Pro-Senescent CC	-1.409	-2.721 to -0.09807	Yes	*	0.0211

Gene	Tukey's multiple comparisons test	Mean Diff.	95.00% CI of diff.	Below threshold?	Summary	Adjusted P Value
Slc20a1	Lgr5-hi vs. Lgr5-dim	-0.7097	-2.137 to 0.7176	No	ns	0.9461
Slc20a1	Lgr5-hi vs. Early TA	0.08437	-1.864 to 2.032	No	ns	>0.9999
Slc20a1	Lgr5-hi vs. Sec Prog	0.02709	-1.073 to 1.127	No	ns	>0.9999
Slc20a1	Lgr5-hi vs. Paneth-like	0.2027	-0.8373 to 1.243	No	ns	>0.9999
Slc20a1	Lgr5-hi vs. Goblet	0.1939	-0.7194 to 1.107	No	ns	>0.9999
Slc20a1	Lgr5-hi vs. Endocrine	0.2400	-1.508 to 1.988	No	ns	>0.9999
Slc20a1	Lgr5-hi vs. Tuft	0.2762	-1.228 to 1.780	No	ns	>0.9999
Slc20a1	Lgr5-hi vs. Abs Prog	-0.7952	-2.609 to 1.018	No	ns	0.9824
Slc20a1	Lgr5-hi vs. Pre-DCC	-2.406	-3.561 to -1.250	Yes	****	<0.0001
Slc20a1	Lgr5-hi vs. Pre-PCC	-4.928	-6.331 to -3.525	Yes	****	<0.0001
Slc20a1	Lgr5-hi vs. Pro-DCC	-4.821	-5.771 to -3.872	Yes	****	<0.0001
Slc20a1	Lgr5-hi vs. Mature DCC	-4.049	-4.908 to -3.190	Yes	****	<0.0001
Slc20a1	Lgr5-hi vs. Mature PCC	-1.164	-2.170 to -0.1587	Yes	**	0.0073
Slc20a1	Lgr5-hi vs. P-I CC	-0.7446	-3.171 to 1.682	No	ns	0.9996
Slc20a1	Lgr5-hi vs. Pro-Senescent CC	-1.649	-2.674 to -0.6247	Yes	****	<0.0001
Slc20a1	Lgr5-dim vs. Early TA	0.7941	-1.356 to 2.944	No	ns	0.9970
Slc20a1	Lgr5-dim vs. Sec Prog	0.7368	-0.6912 to 2.165	No	ns	0.9274
Slc20a1	Lgr5-dim vs. Paneth-like	0.9124	-0.4699 to 2.295	No	ns	0.6518
Slc20a1	Lgr5-dim vs. Goblet	0.9036	-0.3861 to 2.193	No	ns	0.5464
Slc20a1	Lgr5-dim vs. Endocrine	0.9496	-1.021 to 2.920	No	ns	0.9586
Slc20a1	Lgr5-dim vs. Tuft	0.9859	-0.7722 to 2.744	No	ns	0.8660
Slc20a1	Lgr5-dim vs. Abs Prog	-0.08555	-2.115 to 1.944	No	ns	>0.9999
Slc20a1	Lgr5-dim vs. Pre-DCC	-1.696	-3.167 to -0.2245	Yes	**	0.0078
Slc20a1	Lgr5-dim vs. Pre-PCC	-4.218	-5.891 to -2.546	Yes	****	<0.0001
Slc20a1	Lgr5-dim vs. Pro-DCC	-4.112	-5.427 to -2.796	Yes	****	<0.0001
Slc20a1	Lgr5-dim vs. Mature DCC	-3.340	-4.591 to -2.088	Yes	****	<0.0001
Slc20a1	Lgr5-dim vs. Mature PCC	-0.4547	-1.811 to 0.9020	No	ns	0.9990
Slc20a1	Lgr5-dim vs. P-I CC	-0.03496	-2.627 to 2.557	No	ns	>0.9999
Slc20a1	Lgr5-dim vs. Pro-Senescent CC	-0.9398	-2.311 to 0.4311	No	ns	0.5859
Slc20a1	Early TA vs. Sec Prog	-0.05728	-2.006 to 1.891	No	ns	>0.9999
Slc20a1	Early TA vs. Paneth-like	0.1184	-1.797 to 2.034	No	ns	>0.9999
Slc20a1	Early TA vs. Goblet	0.1095	-1.740 to 1.959	No	ns	>0.9999
Slc20a1	Early TA vs. Endocrine	0.1556	-2.220 to 2.531	No	ns	>0.9999
Slc20a1	Early TA vs. Tuft	0.1918	-2.010 to 2.394	No	ns	>0.9999
Slc20a1	Early TA vs. Abs Prog	-0.8796	-3.303 to 1.544	No	ns	0.9975
Slc20a1	Early TA vs. Pre-DCC	-2.490	-4.471 to -0.5094	Yes	**	0.0018
Slc20a1	Early TA vs. Pre-PCC	-5.012	-7.147 to -2.878	Yes	****	<0.0001
Slc20a1	Early TA vs. Pro-DCC	-4.906	-6.773 to -3.038	Yes	****	<0.0001
Slc20a1	Early TA vs. Mature DCC	-4.134	-5.957 to -2.310	Yes	****	<0.0001
Slc20a1	Early TA vs. Mature PCC	-1.249	-3.146 to 0.6481	No	ns	0.6562
Slc20a1	Early TA vs. P-I CC	-0.8290	-3.740 to 2.082	No	ns	0.9999
Slc20a1	Early TA vs. Pro-Senescent CC	-1.734	-3.641 to 0.1732	No	ns	0.1249
Slc20a1	Abs Prog vs. Pre-DCC	-1.610	-3.459 to 0.2379	No	ns	0.1748
Slc20a1	Abs Prog vs. Pre-PCC	-4.133	-6.145 to -2.121	Yes	****	<0.0001
Slc20a1	Abs Prog vs. Pro-DCC	-4.026	-5.753 to -2.299	Yes	****	<0.0001
Slc20a1	Abs Prog vs. Mature DCC	-3.254	-4.933 to -1.575	Yes	****	<0.0001
Slc20a1	Abs Prog vs. Mature PCC	-0.3691	-2.127 to 1.389	No	ns	>0.9999
Slc20a1	Abs Prog vs. P-I CC	0.05060	-2.772 to 2.873	No	ns	>0.9999
Slc20a1	Abs Prog vs. Pro-Senescent CC	-0.8543	-2.624 to 0.9150	No	ns	0.9579
Slc20a1	Pre-DCC vs. Pre-PCC	-2.522	-3.970 to -1.075	Yes	****	<0.0001
Slc20a1	Pre-DCC vs. Pro-DCC	-2.416	-3.430 to -1.401	Yes	****	<0.0001
Slc20a1	Pre-DCC vs. Mature DCC	-1.644	-2.574 to -0.7131	Yes	****	<0.0001
Slc20a1	Pre-DCC vs. Mature PCC	1.241	0.1738 to 2.309	Yes	**	0.0068
Slc20a1	Pre-DCC vs. P-I CC	1.661	-0.7919 to 4.114	No	ns	0.6078
Slc20a1	Pre-DCC vs. Pro-Senescent CC	0.7561	-0.3293 to 1.842	No	ns	0.5570
Slc20a1	Pre-PCC vs. Pro-DCC	0.1069	-1.182 to 1.396	No	ns	>0.9999
Slc20a1	Pre-PCC vs. Mature DCC	0.8789	-0.3449 to 2.103	No	ns	0.5005
Slc20a1	Pre-PCC vs. Mature PCC	3.764	2.433 to 5.095	Yes	****	<0.0001
Slc20a1	Pre-PCC vs. P-I CC	4.183	1.605 to 6.762	Yes	****	<0.0001
Slc20a1	Pre-PCC vs. Pro-Senescent CC	3.279	1.933 to 4.624	Yes	****	<0.0001

Slc20a1	Pro-DCC vs. Mature DCC	0.7720	0.1151 to 1.429	Yes	**	0.0058
Slc20a1	Pro-DCC vs. Mature PCC	3.657	2.817 to 4.497	Yes	****	<0.0001
Slc20a1	Pro-DCC vs. P-I CC	4.077	1.714 to 6.439	Yes	****	<0.0001
Slc20a1	Pro-DCC vs. Pro-Senescent CC	3.172	2.309 to 4.034	Yes	****	<0.0001
Slc20a1	Mature DCC vs. Mature PCC	2.885	2.149 to 3.621	Yes	****	<0.0001
Slc20a1	Mature DCC vs. P-I CC	3.305	0.9769 to 5.632	Yes	***	0.0001
Slc20a1	Mature DCC vs. Pro-Senescent CC	2.400	1.638 to 3.161	Yes	****	<0.0001
Slc20a1	Mature PCC vs. P-I CC	0.4197	-1.966 to 2.806	No	ns	>0.9999
Slc20a1	Mature PCC vs. Pro-Senescent CC	-0.4851	-1.409 to 0.4388	No	ns	0.9169
Slc20a1	P-I CC vs. Pro-Senescent CC	-0.9048	-3.299 to 1.489	No	ns	0.9961
Slc20a1	Sec Prog vs. Paneth-like	0.1757	-0.8654 to 1.217	No	ns	>0.9999
Slc20a1	Sec Prog vs. Goblet	0.1668	-0.7476 to 1.081	No	ns	>0.9999
Slc20a1	Sec Prog vs. Endocrine	0.2129	-1.536 to 1.961	No	ns	>0.9999
Slc20a1	Sec Prog vs. Tuft	0.2491	-1.256 to 1.754	No	ns	>0.9999
Slc20a1	Sec Prog vs. Abs Prog	-0.8223	-2.636 to 0.9916	No	ns	0.9759
Slc20a1	Sec Prog vs. Pre-DCC	-2.433	-3.589 to -1.276	Yes	****	<0.0001
Slc20a1	Sec Prog vs. Pre-PCC	-4.955	-6.359 to -3.552	Yes	****	<0.0001
Slc20a1	Sec Prog vs. Pro-DCC	-4.848	-5.799 to -3.898	Yes	****	<0.0001
Slc20a1	Sec Prog vs. Mature DCC	-4.076	-4.936 to -3.216	Yes	****	<0.0001
Slc20a1	Sec Prog vs. Mature PCC	-1.191	-2.198 to -0.1847	Yes	**	0.0051
Slc20a1	Sec Prog vs. P-I CC	-0.7717	-3.199 to 1.655	No	ns	0.9995
Slc20a1	Sec Prog vs. Pro-Senescent CC	-1.677	-2.702 to -0.6508	Yes	****	<0.0001
Slc20a1	Paneth-like vs. Goblet	-0.008867	-0.8502 to 0.8324	No	ns	>0.9999
Slc20a1	Paneth-like vs. Endocrine	0.03721	-1.674 to 1.748	No	ns	>0.9999
Slc20a1	Paneth-like vs. Tuft	0.07345	-1.388 to 1.535	No	ns	>0.9999
Slc20a1	Paneth-like vs. Abs Prog	-0.9980	-2.776 to 0.7802	No	ns	0.8652
Slc20a1	Paneth-like vs. Pre-DCC	-2.608	-3.708 to -1.508	Yes	****	<0.0001
Slc20a1	Paneth-like vs. Pre-PCC	-5.131	-6.488 to -3.774	Yes	****	<0.0001
Slc20a1	Paneth-like vs. Pro-DCC	-5.024	-5.905 to -4.143	Yes	****	<0.0001
Slc20a1	Paneth-like vs. Mature DCC	-4.252	-5.034 to -3.470	Yes	****	<0.0001
Slc20a1	Paneth-like vs. Mature PCC	-1.367	-2.308 to -0.4263	Yes	****	<0.0001
Slc20a1	Paneth-like vs. P-I CC	-0.9474	-3.348 to 1.453	No	ns	0.9938
Slc20a1	Paneth-like vs. Pro-Senescent CC	-1.852	-2.813 to -0.8911	Yes	****	<0.0001
Slc20a1	Goblet vs. Endocrine	0.04608	-1.591 to 1.683	No	ns	>0.9999
Slc20a1	Goblet vs. Tuft	0.08231	-1.292 to 1.456	No	ns	>0.9999
Slc20a1	Goblet vs. Abs Prog	-0.9891	-2.696 to 0.7180	No	ns	0.8333
Slc20a1	Goblet vs. Pre-DCC	-2.600	-3.580 to -1.619	Yes	****	<0.0001
Slc20a1	Goblet vs. Pre-PCC	-5.122	-6.384 to -3.860	Yes	****	<0.0001
Slc20a1	Goblet vs. Pro-DCC	-5.015	-5.742 to -4.289	Yes	****	<0.0001
Slc20a1	Goblet vs. Mature DCC	-4.243	-4.846 to -3.640	Yes	****	<0.0001
Slc20a1	Goblet vs. Mature PCC	-1.358	-2.157 to -0.5598	Yes	****	<0.0001
Slc20a1	Goblet vs. P-I CC	-0.9385	-3.287 to 1.410	No	ns	0.9930
Slc20a1	Goblet vs. Pro-Senescent CC	-1.843	-2.666 to -1.021	Yes	****	<0.0001
Slc20a1	Endocrine vs. Tuft	0.03623	-1.991 to 2.063	No	ns	>0.9999
Slc20a1	Endocrine vs. Abs Prog	-1.035	-3.301 to 1.231	No	ns	0.9742
Slc20a1	Endocrine vs. Pre-DCC	-2.646	-4.430 to -0.8615	Yes	****	<0.0001
Slc20a1	Endocrine vs. Pre-PCC	-5.168	-7.121 to -3.215	Yes	****	<0.0001
Slc20a1	Endocrine vs. Pro-DCC	-5.061	-6.719 to -3.403	Yes	****	<0.0001
Slc20a1	Endocrine vs. Mature DCC	-4.289	-5.897 to -2.682	Yes	****	<0.0001
Slc20a1	Endocrine vs. Mature PCC	-1.404	-3.095 to 0.2863	No	ns	0.2426
Slc20a1	Endocrine vs. P-I CC	-0.9846	-3.766 to 1.797	No	ns	0.9981
Slc20a1	Endocrine vs. Pro-Senescent CC	-1.889	-3.591 to -0.1874	Yes	*	0.0136
Slc20a1	Tuft vs. Abs Prog	-1.071	-3.155 to 1.012	No	ns	0.9293
Slc20a1	Tuft vs. Pre-DCC	-2.682	-4.228 to -1.136	Yes	****	<0.0001
Slc20a1	Tuft vs. Pre-PCC	-5.204	-6.943 to -3.466	Yes	****	<0.0001
Slc20a1	Tuft vs. Pro-DCC	-5.097	-6.496 to -3.699	Yes	****	<0.0001
Slc20a1	Tuft vs. Mature DCC	-4.325	-5.664 to -2.987	Yes	****	<0.0001
Slc20a1	Tuft vs. Mature PCC	-1.441	-2.878 to -0.003432	Yes	*	0.0487
Slc20a1	Tuft vs. P-I CC	-1.021	-3.656 to 1.614	No	ns	0.9949
Slc20a1	Tuft vs. Pro-Senescent CC	-1.926	-3.376 to -0.4751	Yes	***	0.0006

Gene	Tukey's multiple comparisons test	Mean Diff.	95.00% CI of diff.	Below threshold?	Summary	Adjusted P Value
Dmbt1	Lgr5-hi vs. Lgr5-dim	-0.06907	-0.9334 to 0.7952	No	ns	>0.9999
Dmbt1	Lgr5-hi vs. Early TA	-0.05708	-1.237 to 1.123	No	ns	>0.9999
Dmbt1	Lgr5-hi vs. Sec Prog	-0.01102	-0.6772 to 0.6551	No	ns	>0.9999
Dmbt1	Lgr5-hi vs. Paneth-like	-0.03234	-0.6622 to 0.5975	No	ns	>0.9999
Dmbt1	Lgr5-hi vs. Goblet	0.004252	-0.5488 to 0.5573	No	ns	>0.9999
Dmbt1	Lgr5-hi vs. Endocrine	-0.5627	-1.621 to 0.4957	No	ns	0.9087
Dmbt1	Lgr5-hi vs. Tuft	0.05539	-0.8554 to 0.9661	No	ns	>0.9999
Dmbt1	Lgr5-hi vs. Abs Prog	-0.1790	-1.277 to 0.9191	No	ns	>0.9999
Dmbt1	Lgr5-hi vs. Pre-DCC	-0.5657	-1.266 to 0.1343	No	ns	0.2866
Dmbt1	Lgr5-hi vs. Pre-PCC	-19.47	-20.32 to -18.62	Yes	****	<0.0001
Dmbt1	Lgr5-hi vs. Pro-DCC	-0.9725	-1.548 to -0.3975	Yes	****	<0.0001
Dmbt1	Lgr5-hi vs. Mature DCC	-0.5777	-1.098 to -0.05756	Yes	*	0.0135
Dmbt1	Lgr5-hi vs. Mature PCC	-5.156	-5.765 to -4.547	Yes	****	<0.0001
Dmbt1	Lgr5-hi vs. P-I CC	-0.1438	-1.613 to 1.326	No	ns	>0.9999
Dmbt1	Lgr5-hi vs. Pro-Senescent CC	-0.07276	-0.6933 to 0.5478	No	ns	>0.9999
Dmbt1	Lgr5-dim vs. Early TA	0.01199	-1.290 to 1.314	No	ns	>0.9999
Dmbt1	Lgr5-dim vs. Sec Prog	0.05805	-0.8067 to 0.9228	No	ns	>0.9999
Dmbt1	Lgr5-dim vs. Paneth-like	0.03672	-0.8004 to 0.8738	No	ns	>0.9999
Dmbt1	Lgr5-dim vs. Goblet	0.07332	-0.7076 to 0.8543	No	ns	>0.9999
Dmbt1	Lgr5-dim vs. Endocrine	-0.4936	-1.687 to 0.6998	No	ns	0.9900
Dmbt1	Lgr5-dim vs. Tuft	0.1245	-0.9402 to 1.189	No	ns	>0.9999
Dmbt1	Lgr5-dim vs. Abs Prog	-0.1099	-1.339 to 1.119	No	ns	>0.9999
Dmbt1	Lgr5-dim vs. Pre-DCC	-0.4966	-1.388 to 0.3944	No	ns	0.8715
Dmbt1	Lgr5-dim vs. Pre-PCC	-19.40	-20.42 to -18.39	Yes	****	<0.0001
Dmbt1	Lgr5-dim vs. Pro-DCC	-0.9035	-1.700 to -0.1068	Yes	**	0.0100
Dmbt1	Lgr5-dim vs. Mature DCC	-0.5086	-1.267 to 0.2494	No	ns	0.6238
Dmbt1	Lgr5-dim vs. Mature PCC	-5.087	-5.909 to -4.266	Yes	****	<0.0001
Dmbt1	Lgr5-dim vs. P-I CC	-0.07468	-1.644 to 1.495	No	ns	>0.9999
Dmbt1	Lgr5-dim vs. Pro-Senescent CC	-0.003689	-0.8338 to 0.8265	No	ns	>0.9999
Dmbt1	Early TA vs. Sec Prog	0.04605	-1.134 to 1.226	No	ns	>0.9999
Dmbt1	Early TA vs. Paneth-like	0.02473	-1.135 to 1.185	No	ns	>0.9999
Dmbt1	Early TA vs. Goblet	0.06133	-1.059 to 1.181	No	ns	>0.9999
Dmbt1	Early TA vs. Endocrine	-0.5056	-1.944 to 0.9327	No	ns	0.9982
Dmbt1	Early TA vs. Tuft	0.1125	-1.221 to 1.446	No	ns	>0.9999
Dmbt1	Early TA vs. Abs Prog	-0.1219	-1.590 to 1.346	No	ns	>0.9999
Dmbt1	Early TA vs. Pre-DCC	-0.5086	-1.708 to 0.6908	No	ns	0.9872
Dmbt1	Early TA vs. Pre-PCC	-19.42	-20.71 to -18.12	Yes	****	<0.0001
Dmbt1	Early TA vs. Pro-DCC	-0.9155	-2.046 to 0.2156	No	ns	0.2841
Dmbt1	Early TA vs. Mature DCC	-0.5206	-1.625 to 0.5835	No	ns	0.9657
Dmbt1	Early TA vs. Mature PCC	-5.099	-6.248 to -3.951	Yes	****	<0.0001
Dmbt1	Early TA vs. P-I CC	-0.08667	-1.850 to 1.676	No	ns	>0.9999
Dmbt1	Early TA vs. Pro-Senescent CC	-0.01568	-1.171 to 1.139	No	ns	>0.9999
Dmbt1	Abs Prog vs. Pre-DCC	-0.3867	-1.506 to 0.7326	No	ns	0.9986
Dmbt1	Abs Prog vs. Pre-PCC	-19.29	-20.51 to -18.08	Yes	****	<0.0001
Dmbt1	Abs Prog vs. Pro-DCC	-0.7936	-1.839 to 0.2521	No	ns	0.3977
Dmbt1	Abs Prog vs. Mature DCC	-0.3987	-1.415 to 0.6178	No	ns	0.9942
Dmbt1	Abs Prog vs. Mature PCC	-4.977	-6.042 to -3.913	Yes	****	<0.0001
Dmbt1	Abs Prog vs. P-I CC	0.03522	-1.674 to 1.745	No	ns	>0.9999
Dmbt1	Abs Prog vs. Pro-Senescent CC	0.1062	-0.9652 to 1.178	No	ns	>0.9999
Dmbt1	Pre-DCC vs. Pre-PCC	-18.91	-19.78 to -18.03	Yes	****	<0.0001
Dmbt1	Pre-DCC vs. Pro-DCC	-0.4068	-1.021 to 0.2077	No	ns	0.6468
Dmbt1	Pre-DCC vs. Mature DCC	-0.01200	-0.5755 to 0.5515	No	ns	>0.9999
Dmbt1	Pre-DCC vs. Mature PCC	-4.591	-5.237 to -3.944	Yes	****	<0.0001
Dmbt1	Pre-DCC vs. P-I CC	0.4219	-1.063 to 1.907	No	ns	0.9999
Dmbt1	Pre-DCC vs. Pro-Senescent CC	0.4929	-0.1644 to 1.150	No	ns	0.4197
Dmbt1	Pre-PCC vs. Pro-DCC	18.50	17.72 to 19.28	Yes	****	<0.0001
Dmbt1	Pre-PCC vs. Mature DCC	18.90	18.15 to 19.64	Yes	****	<0.0001
Dmbt1	Pre-PCC vs. Mature PCC	14.32	13.51 to 15.12	Yes	****	<0.0001
Dmbt1	Pre-PCC vs. P-I CC	19.33	17.77 to 20.89	Yes	****	<0.0001
Dmbt1	Pre-PCC vs. Pro-Senescent CC	19.40	18.59 to 20.22	Yes	****	<0.0001
Dmbt1	Pro-DCC vs. Mature DCC	0.3948	-0.002949 to 0.7926	No	ns	0.0542
Dmbt1	Pro-DCC vs. Mature PCC	-4.184	-4.692 to -3.675	Yes	****	<0.0001

Dmbt1	Pro-DCC vs. P-I CC	0.8288	-0.6020 to 2.260	No	ns	0.8336
Dmbt1	Pro-DCC vs. Pro-Senescent CC	0.8998	0.3775 to 1.422	Yes	****	<0.0001
Dmbt1	Mature DCC vs. Mature PCC	-4.579	-5.024 to -4.133	Yes	****	<0.0001
Dmbt1	Mature DCC vs. P-I CC	0.4339	-0.9757 to 1.844	No	ns	0.9996
Dmbt1	Mature DCC vs. Pro-Senescent CC	0.5049	0.04375 to 0.9661	Yes	*	0.0165
Dmbt1	Mature PCC vs. P-I CC	5.013	3.568 to 6.457	Yes	****	<0.0001
Dmbt1	Mature PCC vs. Pro-Senescent CC	5.084	4.524 to 5.643	Yes	****	<0.0001
Dmbt1	P-I CC vs. Pro-Senescent CC	0.07099	-1.379 to 1.521	No	ns	>0.9999
Dmbt1	Sec Prog vs. Paneth-like	-0.02132	-0.6518 to 0.6091	No	ns	>0.9999
Dmbt1	Sec Prog vs. Goblet	0.01527	-0.5385 to 0.5690	No	ns	>0.9999
Dmbt1	Sec Prog vs. Endocrine	-0.5517	-1.610 to 0.5071	No	ns	0.9216
Dmbt1	Sec Prog vs. Tuft	0.06641	-0.8448 to 0.9776	No	ns	>0.9999
Dmbt1	Sec Prog vs. Abs Prog	-0.1679	-1.266 to 0.9305	No	ns	>0.9999
Dmbt1	Sec Prog vs. Pre-DCC	-0.5547	-1.255 to 0.1458	No	ns	0.3214
Dmbt1	Sec Prog vs. Pre-PCC	-19.46	-20.31 to -18.61	Yes	****	<0.0001
Dmbt1	Sec Prog vs. Pro-DCC	-0.9615	-1.537 to -0.3858	Yes	****	<0.0001
Dmbt1	Sec Prog vs. Mature DCC	-0.5667	-1.088 to -0.04577	Yes	*	0.0180
Dmbt1	Sec Prog vs. Mature PCC	-5.145	-5.755 to -4.536	Yes	****	<0.0001
Dmbt1	Sec Prog vs. P-I CC	-0.1327	-1.602 to 1.337	No	ns	>0.9999
Dmbt1	Sec Prog vs. Pro-Senescent CC	-0.06174	-0.6829 to 0.5595	No	ns	>0.9999
Dmbt1	Paneth-like vs. Goblet	0.03660	-0.4729 to 0.5461	No	ns	>0.9999
Dmbt1	Paneth-like vs. Endocrine	-0.5303	-1.567 to 0.5060	No	ns	0.9319
Dmbt1	Paneth-like vs. Tuft	0.08774	-0.7972 to 0.9727	No	ns	>0.9999
Dmbt1	Paneth-like vs. Abs Prog	-0.1466	-1.223 to 0.9302	No	ns	>0.9999
Dmbt1	Paneth-like vs. Pre-DCC	-0.5333	-1.199 to 0.1327	No	ns	0.3021
Dmbt1	Paneth-like vs. Pre-PCC	-19.44	-20.26 to -18.62	Yes	****	<0.0001
Dmbt1	Paneth-like vs. Pro-DCC	-0.9402	-1.473 to -0.4069	Yes	****	<0.0001
Dmbt1	Paneth-like vs. Mature DCC	-0.5453	-1.019 to -0.07180	Yes	**	0.0079
Dmbt1	Paneth-like vs. Mature PCC	-5.124	-5.694 to -4.554	Yes	****	<0.0001
Dmbt1	Paneth-like vs. P-I CC	-0.1114	-1.565 to 1.342	No	ns	>0.9999
Dmbt1	Paneth-like vs. Pro-Senescent CC	-0.04041	-0.6225 to 0.5417	No	ns	>0.9999
Dmbt1	Goblet vs. Endocrine	-0.5669	-1.558 to 0.4246	No	ns	0.8472
Dmbt1	Goblet vs. Tuft	0.05114	-0.7809 to 0.8832	No	ns	>0.9999
Dmbt1	Goblet vs. Abs Prog	-0.1832	-1.217 to 0.8506	No	ns	>0.9999
Dmbt1	Goblet vs. Pre-DCC	-0.5699	-1.164 to 0.02405	No	ns	0.0766
Dmbt1	Goblet vs. Pre-PCC	-19.48	-20.24 to -18.71	Yes	****	<0.0001
Dmbt1	Goblet vs. Pro-DCC	-0.9768	-1.417 to -0.5368	Yes	****	<0.0001
Dmbt1	Goblet vs. Mature DCC	-0.5819	-0.9472 to -0.2167	Yes	****	<0.0001
Dmbt1	Goblet vs. Mature PCC	-5.161	-5.644 to -4.677	Yes	****	<0.0001
Dmbt1	Goblet vs. P-I CC	-0.1480	-1.570 to 1.274	No	ns	>0.9999
Dmbt1	Goblet vs. Pro-Senescent CC	-0.07701	-0.5750 to 0.4210	No	ns	>0.9999
Dmbt1	Endocrine vs. Tuft	0.6181	-0.6094 to 1.846	No	ns	0.9402
Dmbt1	Endocrine vs. Abs Prog	0.3837	-0.9885 to 1.756	No	ns	0.9999
Dmbt1	Endocrine vs. Pre-DCC	-0.003011	-1.083 to 1.077	No	ns	>0.9999
Dmbt1	Endocrine vs. Pre-PCC	-18.91	-20.09 to -17.73	Yes	****	<0.0001
Dmbt1	Endocrine vs. Pro-DCC	-0.4099	-1.414 to 0.5941	No	ns	0.9913
Dmbt1	Endocrine vs. Mature DCC	-0.01501	-0.9886 to 0.9585	No	ns	>0.9999
Dmbt1	Endocrine vs. Mature PCC	-4.594	-5.617 to -3.570	Yes	****	<0.0001
Dmbt1	Endocrine vs. P-I CC	0.4189	-1.265 to 2.103	No	ns	>0.9999
Dmbt1	Endocrine vs. Pro-Senescent CC	0.4899	-0.5408 to 1.521	No	ns	0.9632
Dmbt1	Tuft vs. Abs Prog	-0.2344	-1.496 to 1.028	No	ns	>0.9999
Dmbt1	Tuft vs. Pre-DCC	-0.6211	-1.557 to 0.3151	No	ns	0.6432
Dmbt1	Tuft vs. Pre-PCC	-19.53	-20.58 to -18.48	Yes	****	<0.0001
Dmbt1	Tuft vs. Pro-DCC	-1.028	-1.875 to -0.1811	Yes	**	0.0034
Dmbt1	Tuft vs. Mature DCC	-0.6331	-1.444 to 0.1775	No	ns	0.3455
Dmbt1	Tuft vs. Mature PCC	-5.212	-6.082 to -4.341	Yes	****	<0.0001
Dmbt1	Tuft vs. P-I CC	-0.1991	-1.795 to 1.396	No	ns	>0.9999
Dmbt1	Tuft vs. Pro-Senescent CC	-0.1281	-1.007 to 0.7503	No	ns	>0.9999

Supplementary Table 4. One-way ANOVA of PCC lineage genes shown in Figure ED1g.

Gene	Tukey's multiple comparisons test	Mean Diff.	95.00% CI of diff.	Below threshold?	Summary	Adjusted P Value
Fabp2	Lgr5-hi vs. Lgr5-dim	-2.095	-5.504 to 1.314	No	ns	0.7611
Fabp2	Lgr5-hi vs. Early TA	-0.9000	-5.552 to 3.752	No	ns	>0.9999
Fabp2	Lgr5-hi vs. Sec Prog	0.2470	-2.380 to 2.874	No	ns	>0.9999
Fabp2	Lgr5-hi vs. Paneth-like	-0.2220	-2.706 to 2.262	No	ns	>0.9999
Fabp2	Lgr5-hi vs. Goblet	0.9463	-1.235 to 3.127	No	ns	0.9841
Fabp2	Lgr5-hi vs. Endocrine	1.042	-3.132 to 5.216	No	ns	>0.9999
Fabp2	Lgr5-hi vs. Tuft	1.071	-2.521 to 4.662	No	ns	0.9997
Fabp2	Lgr5-hi vs. Abs Prog	-3.519	-7.850 to 0.8117	No	ns	0.2777
Fabp2	Lgr5-hi vs. Pre-DCC	-0.3762	-3.137 to 2.384	No	ns	>0.9999
Fabp2	Lgr5-hi vs. Pre-PCC	-52.50	-55.85 to -49.15	Yes	****	<0.0001
Fabp2	Lgr5-hi vs. Pro-DCC	1.052	-1.215 to 3.320	No	ns	0.9702
Fabp2	Lgr5-hi vs. Mature DCC	1.061	-0.9901 to 3.113	No	ns	0.9259
Fabp2	Lgr5-hi vs. Mature PCC	-2.804	-5.206 to -0.4017	Yes	**	0.0064
Fabp2	Lgr5-hi vs. P-I CC	1.069	-4.727 to 6.864	No	ns	>0.9999
Fabp2	Lgr5-hi vs. Pro-Senescent CC	1.068	-1.379 to 3.516	No	ns	0.9831
Fabp2	Lgr5-dim vs. Early TA	1.195	-3.941 to 6.330	No	ns	>0.9999
Fabp2	Lgr5-dim vs. Sec Prog	2.342	-1.069 to 5.752	No	ns	0.5830
Fabp2	Lgr5-dim vs. Paneth-like	1.873	-1.429 to 5.174	No	ns	0.8550
Fabp2	Lgr5-dim vs. Goblet	3.041	-0.03887 to 6.121	No	ns	0.0573
Fabp2	Lgr5-dim vs. Endocrine	3.137	-1.570 to 7.844	No	ns	0.6354
Fabp2	Lgr5-dim vs. Tuft	3.166	-1.033 to 7.364	No	ns	0.4099
Fabp2	Lgr5-dim vs. Abs Prog	-1.424	-6.270 to 3.422	No	ns	0.9998
Fabp2	Lgr5-dim vs. Pre-DCC	1.719	-1.796 to 5.233	No	ns	0.9530
Fabp2	Lgr5-dim vs. Pre-PCC	-50.40	-54.40 to -46.41	Yes	****	<0.0001
Fabp2	Lgr5-dim vs. Pro-DCC	3.147	0.005320 to 6.289	Yes	*	0.0491
Fabp2	Lgr5-dim vs. Mature DCC	3.156	0.1666 to 6.146	Yes	*	0.0265
Fabp2	Lgr5-dim vs. Mature PCC	-0.7087	-3.949 to 2.531	No	ns	>0.9999
Fabp2	Lgr5-dim vs. P-I CC	3.164	-3.026 to 9.354	No	ns	0.9326
Fabp2	Lgr5-dim vs. Pro-Senescent CC	3.163	-0.1108 to 6.437	No	ns	0.0716
Fabp2	Early TA vs. Sec Prog	1.147	-3.507 to 5.801	No	ns	>0.9999
Fabp2	Early TA vs. Paneth-like	0.6780	-3.896 to 5.252	No	ns	>0.9999
Fabp2	Early TA vs. Goblet	1.846	-2.571 to 6.263	No	ns	0.9889
Fabp2	Early TA vs. Endocrine	1.942	-3.731 to 7.615	No	ns	0.9987
Fabp2	Early TA vs. Tuft	1.971	-3.288 to 7.229	No	ns	0.9964
Fabp2	Early TA vs. Abs Prog	-2.619	-8.408 to 3.170	No	ns	0.9764
Fabp2	Early TA vs. Pre-DCC	0.5238	-4.206 to 5.254	No	ns	>0.9999
Fabp2	Early TA vs. Pre-PCC	-51.60	-56.69 to -46.50	Yes	****	<0.0001
Fabp2	Early TA vs. Pro-DCC	1.952	-2.508 to 6.413	No	ns	0.9827
Fabp2	Early TA vs. Mature DCC	1.961	-2.393 to 6.316	No	ns	0.9773
Fabp2	Early TA vs. Mature PCC	-1.904	-6.434 to 2.627	No	ns	0.9883
Fabp2	Early TA vs. P-I CC	1.969	-4.984 to 8.922	No	ns	0.9999
Fabp2	Early TA vs. Pro-Senescent CC	1.968	-2.586 to 6.523	No	ns	0.9847
Fabp2	Abs Prog vs. Pre-DCC	3.143	-1.271 to 7.557	No	ns	0.5167
Fabp2	Abs Prog vs. Pre-PCC	-48.98	-53.78 to -44.17	Yes	****	<0.0001
Fabp2	Abs Prog vs. Pro-DCC	4.572	0.4473 to 8.696	Yes	*	0.0139
Fabp2	Abs Prog vs. Mature DCC	4.580	0.5711 to 8.589	Yes	**	0.0090
Fabp2	Abs Prog vs. Mature PCC	0.7154	-3.484 to 4.915	No	ns	>0.9999
Fabp2	Abs Prog vs. P-I CC	4.588	-2.154 to 11.33	No	ns	0.5991
Fabp2	Abs Prog vs. Pro-Senescent CC	4.587	0.3618 to 8.813	Yes	*	0.0185
Fabp2	Pre-DCC vs. Pre-PCC	-52.12	-55.58 to -48.66	Yes	****	<0.0001
Fabp2	Pre-DCC vs. Pro-DCC	1.429	-0.9949 to 3.852	No	ns	0.8140
Fabp2	Pre-DCC vs. Mature DCC	1.437	-0.7849 to 3.660	No	ns	0.6851
Fabp2	Pre-DCC vs. Mature PCC	-2.427	-4.977 to 0.1221	No	ns	0.0826
Fabp2	Pre-DCC vs. P-I CC	1.445	-4.413 to 7.303	No	ns	>0.9999
Fabp2	Pre-DCC vs. Pro-Senescent CC	1.445	-1.148 to 4.037	No	ns	0.8717
Fabp2	Pre-PCC vs. Pro-DCC	53.55	50.47 to 56.63	Yes	****	<0.0001
Fabp2	Pre-PCC vs. Mature DCC	53.56	50.64 to 56.48	Yes	****	<0.0001
Fabp2	Pre-PCC vs. Mature PCC	49.69	46.51 to 52.87	Yes	****	<0.0001
Fabp2	Pre-PCC vs. P-I CC	53.57	47.41 to 59.72	Yes	****	<0.0001

Fabp2	Pre-PCC vs. Pro-Senescent CC	53.57	50.35 to 56.78	Yes	****	<0.0001
Fabp2	Pro-DCC vs. Mature DCC	0.008769	-1.560 to 1.578	No	ns	>0.9999
Fabp2	Pro-DCC vs. Mature PCC	-3.856	-5.862 to -1.850	Yes	****	<0.0001
Fabp2	Pro-DCC vs. P-I CC	0.01629	-5.627 to 5.659	No	ns	>0.9999
Fabp2	Pro-DCC vs. Pro-Senescent CC	0.01587	-2.044 to 2.076	No	ns	>0.9999
Fabp2	Mature DCC vs. Mature PCC	-3.865	-5.622 to -2.108	Yes	****	<0.0001
Fabp2	Mature DCC vs. P-I CC	0.007516	-5.552 to 5.567	No	ns	>0.9999
Fabp2	Mature DCC vs. Pro-Senescent CC	0.007104	-1.812 to 1.826	No	ns	>0.9999
Fabp2	Mature PCC vs. P-I CC	3.872	-1.826 to 9.570	No	ns	0.6015
Fabp2	Mature PCC vs. Pro-Senescent CC	3.872	1.665 to 6.079	Yes	****	<0.0001
Fabp2	P-I CC vs. Pro-Senescent CC	-0.0004123	-5.718 to 5.717	No	ns	>0.9999
Fabp2	Sec Prog vs. Paneth-like	-0.4690	-2.955 to 2.017	No	ns	>0.9999
Fabp2	Sec Prog vs. Goblet	0.6993	-1.485 to 2.883	No	ns	0.9994
Fabp2	Sec Prog vs. Endocrine	0.7951	-3.381 to 4.971	No	ns	>0.9999
Fabp2	Sec Prog vs. Tuft	0.8236	-2.770 to 4.417	No	ns	>0.9999
Fabp2	Sec Prog vs. Abs Prog	-3.766	-8.098 to 0.5662	No	ns	0.1777
Fabp2	Sec Prog vs. Pre-DCC	-0.6231	-3.386 to 2.140	No	ns	>0.9999
Fabp2	Sec Prog vs. Pre-PCC	-52.74	-56.10 to -49.39	Yes	****	<0.0001
Fabp2	Sec Prog vs. Pro-DCC	0.8055	-1.465 to 3.076	No	ns	0.9981
Fabp2	Sec Prog vs. Mature DCC	0.8143	-1.240 to 2.869	No	ns	0.9936
Fabp2	Sec Prog vs. Mature PCC	-3.051	-5.455 to -0.6461	Yes	**	0.0015
Fabp2	Sec Prog vs. P-I CC	0.8218	-4.975 to 6.618	No	ns	>0.9999
Fabp2	Sec Prog vs. Pro-Senescent CC	0.8214	-1.629 to 3.271	No	ns	0.9990
Fabp2	Paneth-like vs. Goblet	1.168	-0.8410 to 3.178	No	ns	0.8295
Fabp2	Paneth-like vs. Endocrine	1.264	-2.823 to 5.351	No	ns	0.9996
Fabp2	Paneth-like vs. Tuft	1.293	-2.198 to 4.783	No	ns	0.9969
Fabp2	Paneth-like vs. Abs Prog	-3.297	-7.544 to 0.9498	No	ns	0.3563
Fabp2	Paneth-like vs. Pre-DCC	-0.1542	-2.781 to 2.473	No	ns	>0.9999
Fabp2	Paneth-like vs. Pre-PCC	-52.27	-55.52 to -49.03	Yes	****	<0.0001
Fabp2	Paneth-like vs. Pro-DCC	1.274	-0.8286 to 3.378	No	ns	0.7799
Fabp2	Paneth-like vs. Mature DCC	1.283	-0.5843 to 3.151	No	ns	0.5818
Fabp2	Paneth-like vs. Mature PCC	-2.582	-4.829 to -0.3346	Yes	**	0.0082
Fabp2	Paneth-like vs. P-I CC	1.291	-4.442 to 7.024	No	ns	>0.9999
Fabp2	Paneth-like vs. Pro-Senescent CC	1.290	-1.005 to 3.586	No	ns	0.8638
Fabp2	Goblet vs. Endocrine	0.09577	-3.815 to 4.006	No	ns	>0.9999
Fabp2	Goblet vs. Tuft	0.1243	-3.157 to 3.406	No	ns	>0.9999
Fabp2	Goblet vs. Abs Prog	-4.465	-8.542 to -0.3882	Yes	*	0.0164
Fabp2	Goblet vs. Pre-DCC	-1.322	-3.665 to 1.020	No	ns	0.8597
Fabp2	Goblet vs. Pre-PCC	-53.44	-56.46 to -50.43	Yes	****	<0.0001
Fabp2	Goblet vs. Pro-DCC	0.1062	-1.629 to 1.841	No	ns	>0.9999
Fabp2	Goblet vs. Mature DCC	0.1150	-1.326 to 1.556	No	ns	>0.9999
Fabp2	Goblet vs. Mature PCC	-3.750	-5.657 to -1.843	Yes	****	<0.0001
Fabp2	Goblet vs. P-I CC	0.1225	-5.486 to 5.731	No	ns	>0.9999
Fabp2	Goblet vs. Pro-Senescent CC	0.1221	-1.842 to 2.086	No	ns	>0.9999
Fabp2	Endocrine vs. Tuft	0.02856	-4.813 to 4.870	No	ns	>0.9999
Fabp2	Endocrine vs. Abs Prog	-4.561	-9.973 to 0.8509	No	ns	0.2208
Fabp2	Endocrine vs. Pre-DCC	-1.418	-5.679 to 2.843	No	ns	0.9991
Fabp2	Endocrine vs. Pre-PCC	-53.54	-58.20 to -48.87	Yes	****	<0.0001
Fabp2	Endocrine vs. Pro-DCC	0.01044	-3.949 to 3.970	No	ns	>0.9999
Fabp2	Endocrine vs. Mature DCC	0.01921	-3.820 to 3.859	No	ns	>0.9999
Fabp2	Endocrine vs. Mature PCC	-3.846	-7.883 to 0.1921	No	ns	0.0823
Fabp2	Endocrine vs. P-I CC	0.02672	-6.616 to 6.669	No	ns	>0.9999
Fabp2	Endocrine vs. Pro-Senescent CC	0.02631	-4.039 to 4.091	No	ns	>0.9999
Fabp2	Tuft vs. Abs Prog	-4.590	-9.566 to 0.3870	No	ns	0.1105
Fabp2	Tuft vs. Pre-DCC	-1.447	-5.139 to 2.245	No	ns	0.9943
Fabp2	Tuft vs. Pre-PCC	-53.57	-57.72 to -49.42	Yes	****	<0.0001
Fabp2	Tuft vs. Pro-DCC	-0.01812	-3.358 to 3.322	No	ns	>0.9999
Fabp2	Tuft vs. Mature DCC	-0.009354	-3.206 to 3.187	No	ns	>0.9999
Fabp2	Tuft vs. Mature PCC	-3.874	-7.307 to -0.4419	Yes	*	0.0107
Fabp2	Tuft vs. P-I CC	-0.001838	-6.295 to 6.291	No	ns	>0.9999

Fabp2	Tuft vs. Pro-Senescent CC	-0.002251	-3.467 to 3.462	No	ns	>0.9999

Gene	Tukey's multiple comparisons test	Mean Diff.	95.00% CI of diff.	Below threshold?	Summary	Adjusted P Value
Dpep1	Lgr5-hi vs. Lgr5-dim	-0.06907	-0.9334 to 0.7952	No	ns	>0.9999
Dpep1	Lgr5-hi vs. Early TA	-0.05708	-1.237 to 1.123	No	ns	>0.9999
Dpep1	Lgr5-hi vs. Sec Prog	-0.01102	-0.6772 to 0.6551	No	ns	>0.9999
Dpep1	Lgr5-hi vs. Paneth-like	-0.03234	-0.6622 to 0.5975	No	ns	>0.9999
Dpep1	Lgr5-hi vs. Goblet	0.004252	-0.5488 to 0.5573	No	ns	>0.9999
Dpep1	Lgr5-hi vs. Endocrine	-0.5627	-1.621 to 0.4957	No	ns	0.9087
Dpep1	Lgr5-hi vs. Tuft	0.05539	-0.8554 to 0.9661	No	ns	>0.9999
Dpep1	Lgr5-hi vs. Abs Prog	-0.1790	-1.277 to 0.9191	No	ns	>0.9999
Dpep1	Lgr5-hi vs. Pre-DCC	-0.5657	-1.266 to 0.1343	No	ns	0.2866
Dpep1	Lgr5-hi vs. Pre-PCC	-19.47	-20.32 to -18.62	Yes	****	<0.0001
Dpep1	Lgr5-hi vs. Pro-DCC	-0.9725	-1.548 to -0.3975	Yes	****	<0.0001
Dpep1	Lgr5-hi vs. Mature DCC	-0.5777	-1.098 to -0.05756	Yes	*	0.0135
Dpep1	Lgr5-hi vs. Mature PCC	-5.156	-5.765 to -4.547	Yes	****	<0.0001
Dpep1	Lgr5-hi vs. P-I CC	-0.1438	-1.613 to 1.326	No	ns	>0.9999
Dpep1	Lgr5-hi vs. Pro-Senescent CC	-0.07276	-0.6933 to 0.5478	No	ns	>0.9999
Dpep1	Lgr5-dim vs. Early TA	0.01199	-1.290 to 1.314	No	ns	>0.9999
Dpep1	Lgr5-dim vs. Sec Prog	0.05805	-0.8067 to 0.9228	No	ns	>0.9999
Dpep1	Lgr5-dim vs. Paneth-like	0.03672	-0.8004 to 0.8738	No	ns	>0.9999
Dpep1	Lgr5-dim vs. Goblet	0.07332	-0.7076 to 0.8543	No	ns	>0.9999
Dpep1	Lgr5-dim vs. Endocrine	-0.4936	-1.687 to 0.6998	No	ns	0.9900
Dpep1	Lgr5-dim vs. Tuft	0.1245	-0.9402 to 1.189	No	ns	>0.9999
Dpep1	Lgr5-dim vs. Abs Prog	-0.1099	-1.339 to 1.119	No	ns	>0.9999
Dpep1	Lgr5-dim vs. Pre-DCC	-0.4966	-1.388 to 0.3944	No	ns	0.8715
Dpep1	Lgr5-dim vs. Pre-PCC	-19.40	-20.42 to -18.39	Yes	****	<0.0001
Dpep1	Lgr5-dim vs. Pro-DCC	-0.9035	-1.700 to -0.1068	Yes	**	0.0100
Dpep1	Lgr5-dim vs. Mature DCC	-0.5086	-1.267 to 0.2494	No	ns	0.6238
Dpep1	Lgr5-dim vs. Mature PCC	-5.087	-5.909 to -4.266	Yes	****	<0.0001
Dpep1	Lgr5-dim vs. P-I CC	-0.07468	-1.644 to 1.495	No	ns	>0.9999
Dpep1	Lgr5-dim vs. Pro-Senescent CC	-0.003689	-0.8338 to 0.8265	No	ns	>0.9999
Dpep1	Early TA vs. Sec Prog	0.04605	-1.134 to 1.226	No	ns	>0.9999
Dpep1	Early TA vs. Paneth-like	0.02473	-1.135 to 1.185	No	ns	>0.9999
Dpep1	Early TA vs. Goblet	0.06133	-1.059 to 1.181	No	ns	>0.9999
Dpep1	Early TA vs. Endocrine	-0.5056	-1.944 to 0.9327	No	ns	0.9982
Dpep1	Early TA vs. Tuft	0.1125	-1.221 to 1.446	No	ns	>0.9999
Dpep1	Early TA vs. Abs Prog	-0.1219	-1.590 to 1.346	No	ns	>0.9999
Dpep1	Early TA vs. Pre-DCC	-0.5086	-1.708 to 0.6908	No	ns	0.9872
Dpep1	Early TA vs. Pre-PCC	-19.42	-20.71 to -18.12	Yes	****	<0.0001
Dpep1	Early TA vs. Pro-DCC	-0.9155	-2.046 to 0.2156	No	ns	0.2841
Dpep1	Early TA vs. Mature DCC	-0.5206	-1.625 to 0.5835	No	ns	0.9657
Dpep1	Early TA vs. Mature PCC	-5.099	-6.248 to -3.951	Yes	****	<0.0001
Dpep1	Early TA vs. P-I CC	-0.08667	-1.850 to 1.676	No	ns	>0.9999
Dpep1	Early TA vs. Pro-Senescent CC	-0.01568	-1.171 to 1.139	No	ns	>0.9999
Dpep1	Abs Prog vs. Pre-DCC	-0.3867	-1.506 to 0.7326	No	ns	0.9986
Dpep1	Abs Prog vs. Pre-PCC	-19.29	-20.51 to -18.08	Yes	****	<0.0001
Dpep1	Abs Prog vs. Pro-DCC	-0.7936	-1.839 to 0.2521	No	ns	0.3977
Dpep1	Abs Prog vs. Mature DCC	-0.3987	-1.415 to 0.6178	No	ns	0.9942
Dpep1	Abs Prog vs. Mature PCC	-4.977	-6.042 to -3.913	Yes	****	<0.0001
Dpep1	Abs Prog vs. P-I CC	0.03522	-1.674 to 1.745	No	ns	>0.9999
Dpep1	Abs Prog vs. Pro-Senescent CC	0.1062	-0.9652 to 1.178	No	ns	>0.9999
Dpep1	Pre-DCC vs. Pre-PCC	-18.91	-19.78 to -18.03	Yes	****	<0.0001
Dpep1	Pre-DCC vs. Pro-DCC	-0.4068	-1.021 to 0.2077	No	ns	0.6468
Dpep1	Pre-DCC vs. Mature DCC	-0.01200	-0.5755 to 0.5515	No	ns	>0.9999
Dpep1	Pre-DCC vs. Mature PCC	-4.591	-5.237 to -3.944	Yes	****	<0.0001
Dpep1	Pre-DCC vs. P-I CC	0.4219	-1.063 to 1.907	No	ns	0.9999
Dpep1	Pre-DCC vs. Pro-Senescent CC	0.4929	-0.1644 to 1.150	No	ns	0.4197
Dpep1	Pre-PCC vs. Pro-DCC	18.50	17.72 to 19.28	Yes	****	<0.0001
Dpep1	Pre-PCC vs. Mature DCC	18.90	18.15 to 19.64	Yes	****	<0.0001
Dpep1	Pre-PCC vs. Mature PCC	14.32	13.51 to 15.12	Yes	****	<0.0001
Dpep1	Pre-PCC vs. P-I CC	19.33	17.77 to 20.89	Yes	****	<0.0001
Dpep1	Pre-PCC vs. Pro-Senescent CC	19.40	18.59 to 20.22	Yes	****	<0.0001

Dpep1	Pro-DCC vs. Mature DCC	0.3948	-0.002949 to 0.7926	No	ns	0.0542
Dpep1	Pro-DCC vs. Mature PCC	-4.184	-4.692 to -3.675	Yes	****	<0.0001
Dpep1	Pro-DCC vs. P-I CC	0.8288	-0.6020 to 2.260	No	ns	0.8336
Dpep1	Pro-DCC vs. Pro-Senescent CC	0.8998	0.3775 to 1.422	Yes	****	<0.0001
Dpep1	Mature DCC vs. Mature PCC	-4.579	-5.024 to -4.133	Yes	****	<0.0001
Dpep1	Mature DCC vs. P-I CC	0.4339	-0.9757 to 1.844	No	ns	0.9996
Dpep1	Mature DCC vs. Pro-Senescent CC	0.5049	0.04375 to 0.9661	Yes	*	0.0165
Dpep1	Mature PCC vs. P-I CC	5.013	3.568 to 6.457	Yes	****	<0.0001
Dpep1	Mature PCC vs. Pro-Senescent CC	5.084	4.524 to 5.643	Yes	****	<0.0001
Dpep1	P-I CC vs. Pro-Senescent CC	0.07099	-1.379 to 1.521	No	ns	>0.9999
Dpep1	Sec Prog vs. Paneth-like	-0.02132	-0.6518 to 0.6091	No	ns	>0.9999
Dpep1	Sec Prog vs. Goblet	0.01527	-0.5385 to 0.5690	No	ns	>0.9999
Dpep1	Sec Prog vs. Endocrine	-0.5517	-1.610 to 0.5071	No	ns	0.9216
Dpep1	Sec Prog vs. Tuft	0.06641	-0.8448 to 0.9776	No	ns	>0.9999
Dpep1	Sec Prog vs. Abs Prog	-0.1679	-1.266 to 0.9305	No	ns	>0.9999
Dpep1	Sec Prog vs. Pre-DCC	-0.5547	-1.255 to 0.1458	No	ns	0.3214
Dpep1	Sec Prog vs. Pre-PCC	-19.46	-20.31 to -18.61	Yes	****	<0.0001
Dpep1	Sec Prog vs. Pro-DCC	-0.9615	-1.537 to -0.3858	Yes	****	<0.0001
Dpep1	Sec Prog vs. Mature DCC	-0.5667	-1.088 to -0.04577	Yes	*	0.0180
Dpep1	Sec Prog vs. Mature PCC	-5.145	-5.755 to -4.536	Yes	****	<0.0001
Dpep1	Sec Prog vs. P-I CC	-0.1327	-1.602 to 1.337	No	ns	>0.9999
Dpep1	Sec Prog vs. Pro-Senescent CC	-0.06174	-0.6829 to 0.5595	No	ns	>0.9999
Dpep1	Paneth-like vs. Goblet	0.03660	-0.4729 to 0.5461	No	ns	>0.9999
Dpep1	Paneth-like vs. Endocrine	-0.5303	-1.567 to 0.5060	No	ns	0.9319
Dpep1	Paneth-like vs. Tuft	0.08774	-0.7972 to 0.9727	No	ns	>0.9999
Dpep1	Paneth-like vs. Abs Prog	-0.1466	-1.223 to 0.9302	No	ns	>0.9999
Dpep1	Paneth-like vs. Pre-DCC	-0.5333	-1.199 to 0.1327	No	ns	0.3021
Dpep1	Paneth-like vs. Pre-PCC	-19.44	-20.26 to -18.62	Yes	****	<0.0001
Dpep1	Paneth-like vs. Pro-DCC	-0.9402	-1.473 to -0.4069	Yes	****	<0.0001
Dpep1	Paneth-like vs. Mature DCC	-0.5453	-1.019 to -0.07180	Yes	**	0.0079
Dpep1	Paneth-like vs. Mature PCC	-5.124	-5.694 to -4.554	Yes	****	<0.0001
Dpep1	Paneth-like vs. P-I CC	-0.1114	-1.565 to 1.342	No	ns	>0.9999
Dpep1	Paneth-like vs. Pro-Senescent CC	-0.04041	-0.6225 to 0.5417	No	ns	>0.9999
Dpep1	Goblet vs. Endocrine	-0.5669	-1.558 to 0.4246	No	ns	0.8472
Dpep1	Goblet vs. Tuft	0.05114	-0.7809 to 0.8832	No	ns	>0.9999
Dpep1	Goblet vs. Abs Prog	-0.1832	-1.217 to 0.8506	No	ns	>0.9999
Dpep1	Goblet vs. Pre-DCC	-0.5699	-1.164 to 0.02405	No	ns	0.0766
Dpep1	Goblet vs. Pre-PCC	-19.48	-20.24 to -18.71	Yes	****	<0.0001
Dpep1	Goblet vs. Pro-DCC	-0.9768	-1.417 to -0.5368	Yes	****	<0.0001
Dpep1	Goblet vs. Mature DCC	-0.5819	-0.9472 to -0.2167	Yes	****	<0.0001
Dpep1	Goblet vs. Mature PCC	-5.161	-5.644 to -4.677	Yes	****	<0.0001
Dpep1	Goblet vs. P-I CC	-0.1480	-1.570 to 1.274	No	ns	>0.9999
Dpep1	Goblet vs. Pro-Senescent CC	-0.07701	-0.5750 to 0.4210	No	ns	>0.9999
Dpep1	Endocrine vs. Tuft	0.6181	-0.6094 to 1.846	No	ns	0.9402
Dpep1	Endocrine vs. Abs Prog	0.3837	-0.9885 to 1.756	No	ns	0.9999
Dpep1	Endocrine vs. Pre-DCC	-0.003011	-1.083 to 1.077	No	ns	>0.9999
Dpep1	Endocrine vs. Pre-PCC	-18.91	-20.09 to -17.73	Yes	****	<0.0001
Dpep1	Endocrine vs. Pro-DCC	-0.4099	-1.414 to 0.5941	No	ns	0.9913
Dpep1	Endocrine vs. Mature DCC	-0.01501	-0.9886 to 0.9585	No	ns	>0.9999
Dpep1	Endocrine vs. Mature PCC	-4.594	-5.617 to -3.570	Yes	****	<0.0001
Dpep1	Endocrine vs. P-I CC	0.4189	-1.265 to 2.103	No	ns	>0.9999
Dpep1	Endocrine vs. Pro-Senescent CC	0.4899	-0.5408 to 1.521	No	ns	0.9632
Dpep1	Tuft vs. Abs Prog	-0.2344	-1.496 to 1.028	No	ns	>0.9999
Dpep1	Tuft vs. Pre-DCC	-0.6211	-1.557 to 0.3151	No	ns	0.6432
Dpep1	Tuft vs. Pre-PCC	-19.53	-20.58 to -18.48	Yes	****	<0.0001
Dpep1	Tuft vs. Pro-DCC	-1.028	-1.875 to -0.1811	Yes	**	0.0034
Dpep1	Tuft vs. Mature DCC	-0.6331	-1.444 to 0.1775	No	ns	0.3455
Dpep1	Tuft vs. Mature PCC	-5.212	-6.082 to -4.341	Yes	****	<0.0001
Dpep1	Tuft vs. P-I CC	-0.1991	-1.795 to 1.396	No	ns	>0.9999
Dpep1	Tuft vs. Pro-Senescent CC	-0.1281	-1.007 to 0.7503	No	ns	>0.9999

Gene	Tukey's multiple comparisons test	Mean Diff.	95.00% CI of diff.	Below threshold?	Summary	Adjusted P Value
Emp1	Lgr5-hi vs. Lgr5-dim	0.05521	-2.038 to 2.148	No	ns	>0.9999
Emp1	Lgr5-hi vs. Early TA	-0.1237	-2.980 to 2.733	No	ns	>0.9999
Emp1	Lgr5-hi vs. Sec Prog	0.1814	-1.432 to 1.794	No	ns	>0.9999
Emp1	Lgr5-hi vs. Paneth-like	0.3377	-1.187 to 1.863	No	ns	>0.9999
Emp1	Lgr5-hi vs. Goblet	0.3190	-1.020 to 1.658	No	ns	>0.9999
Emp1	Lgr5-hi vs. Endocrine	0.3712	-2.192 to 2.934	No	ns	>0.9999
Emp1	Lgr5-hi vs. Tuft	0.3465	-1.859 to 2.552	No	ns	>0.9999
Emp1	Lgr5-hi vs. Abs Prog	0.004762	-2.654 to 2.664	No	ns	>0.9999
Emp1	Lgr5-hi vs. Pre-DCC	0.1130	-1.582 to 1.808	No	ns	>0.9999
Emp1	Lgr5-hi vs. Pre-PCC	-47.15	-49.21 to -45.10	Yes	****	<0.0001
Emp1	Lgr5-hi vs. Pro-DCC	-0.1689	-1.561 to 1.223	No	ns	>0.9999
Emp1	Lgr5-hi vs. Mature DCC	-0.08818	-1.348 to 1.171	No	ns	>0.9999
Emp1	Lgr5-hi vs. Mature PCC	-6.800	-8.274 to -5.325	Yes	****	<0.0001
Emp1	Lgr5-hi vs. P-I CC	-0.1455	-3.704 to 3.413	No	ns	>0.9999
Emp1	Lgr5-hi vs. Pro-Senescent CC	0.2248	-1.278 to 1.727	No	ns	>0.9999
Emp1	Lgr5-dim vs. Early TA	-0.1789	-3.332 to 2.974	No	ns	>0.9999
Emp1	Lgr5-dim vs. Sec Prog	0.1262	-1.968 to 2.220	No	ns	>0.9999
Emp1	Lgr5-dim vs. Paneth-like	0.2825	-1.744 to 2.309	No	ns	>0.9999
Emp1	Lgr5-dim vs. Goblet	0.2638	-1.627 to 2.155	No	ns	>0.9999
Emp1	Lgr5-dim vs. Endocrine	0.3160	-2.574 to 3.206	No	ns	>0.9999
Emp1	Lgr5-dim vs. Tuft	0.2913	-2.287 to 2.869	No	ns	>0.9999
Emp1	Lgr5-dim vs. Abs Prog	-0.05044	-3.026 to 2.925	No	ns	>0.9999
Emp1	Lgr5-dim vs. Pre-DCC	0.05778	-2.100 to 2.215	No	ns	>0.9999
Emp1	Lgr5-dim vs. Pre-PCC	-47.21	-49.66 to -44.76	Yes	****	<0.0001
Emp1	Lgr5-dim vs. Pro-DCC	-0.2241	-2.153 to 1.705	No	ns	>0.9999
Emp1	Lgr5-dim vs. Mature DCC	-0.1434	-1.979 to 1.692	No	ns	>0.9999
Emp1	Lgr5-dim vs. Mature PCC	-6.855	-8.844 to -4.866	Yes	****	<0.0001
Emp1	Lgr5-dim vs. P-I CC	-0.2007	-4.001 to 3.600	No	ns	>0.9999
Emp1	Lgr5-dim vs. Pro-Senescent CC	0.1696	-1.841 to 2.180	No	ns	>0.9999
Emp1	Early TA vs. Sec Prog	0.3051	-2.552 to 3.162	No	ns	>0.9999
Emp1	Early TA vs. Paneth-like	0.4614	-2.347 to 3.270	No	ns	>0.9999
Emp1	Early TA vs. Goblet	0.4428	-2.269 to 3.155	No	ns	>0.9999
Emp1	Early TA vs. Endocrine	0.4949	-2.988 to 3.978	No	ns	>0.9999
Emp1	Early TA vs. Tuft	0.4702	-2.759 to 3.699	No	ns	>0.9999
Emp1	Early TA vs. Abs Prog	0.1285	-3.426 to 3.683	No	ns	>0.9999
Emp1	Early TA vs. Pre-DCC	0.2367	-2.667 to 3.141	No	ns	>0.9999
Emp1	Early TA vs. Pre-PCC	-47.03	-50.16 to -43.90	Yes	****	<0.0001
Emp1	Early TA vs. Pro-DCC	-0.04514	-2.784 to 2.694	No	ns	>0.9999
Emp1	Early TA vs. Mature DCC	0.03554	-2.638 to 2.709	No	ns	>0.9999
Emp1	Early TA vs. Mature PCC	-6.676	-9.458 to -3.895	Yes	****	<0.0001
Emp1	Early TA vs. P-I CC	-0.02182	-4.291 to 4.247	No	ns	>0.9999
Emp1	Early TA vs. Pro-Senescent CC	0.3485	-2.448 to 3.145	No	ns	>0.9999
Emp1	Abs Prog vs. Pre-DCC	0.1082	-2.602 to 2.818	No	ns	>0.9999
Emp1	Abs Prog vs. Pre-PCC	-47.16	-50.11 to -44.21	Yes	****	<0.0001
Emp1	Abs Prog vs. Pro-DCC	-0.1736	-2.706 to 2.358	No	ns	>0.9999
Emp1	Abs Prog vs. Mature DCC	-0.09294	-2.554 to 2.369	No	ns	>0.9999
Emp1	Abs Prog vs. Mature PCC	-6.805	-9.383 to -4.226	Yes	****	<0.0001
Emp1	Abs Prog vs. P-I CC	-0.1503	-4.290 to 3.989	No	ns	>0.9999
Emp1	Abs Prog vs. Pro-Senescent CC	0.2200	-2.374 to 2.814	No	ns	>0.9999
Emp1	Pre-DCC vs. Pre-PCC	-47.27	-49.39 to -45.14	Yes	****	<0.0001
Emp1	Pre-DCC vs. Pro-DCC	-0.2819	-1.770 to 1.206	No	ns	>0.9999
Emp1	Pre-DCC vs. Mature DCC	-0.2012	-1.566 to 1.163	No	ns	>0.9999
Emp1	Pre-DCC vs. Mature PCC	-6.913	-8.478 to -5.348	Yes	****	<0.0001
Emp1	Pre-DCC vs. P-I CC	-0.2585	-3.855 to 3.338	No	ns	>0.9999
Emp1	Pre-DCC vs. Pro-Senescent CC	0.1118	-1.480 to 1.703	No	ns	>0.9999
Emp1	Pre-PCC vs. Pro-DCC	46.99	45.10 to 48.88	Yes	****	<0.0001
Emp1	Pre-PCC vs. Mature DCC	47.07	45.27 to 48.86	Yes	****	<0.0001
Emp1	Pre-PCC vs. Mature PCC	40.35	38.40 to 42.31	Yes	****	<0.0001
Emp1	Pre-PCC vs. P-I CC	47.01	43.23 to 50.79	Yes	****	<0.0001
Emp1	Pre-PCC vs. Pro-Senescent CC	47.38	45.41 to 49.35	Yes	****	<0.0001
Emp1	Pro-DCC vs. Mature DCC	0.08069	-0.8825 to 1.044	No	ns	>0.9999
Emp1	Pro-DCC vs. Mature PCC	-6.631	-7.862 to -5.400	Yes	****	<0.0001

Emp1	Pro-DCC vs. P-I CC	0.02333	-3.441 to 3.488	No	ns	>0.9999
Emp1	Pro-DCC vs. Pro-Senescent CC	0.3936	-0.8711 to 1.658	No	ns	0.9996
Emp1	Mature DCC vs. Mature PCC	-6.712	-7.790 to -5.633	Yes	****	<0.0001
Emp1	Mature DCC vs. P-I CC	-0.05736	-3.471 to 3.356	No	ns	>0.9999
Emp1	Mature DCC vs. Pro-Senescent CC	0.3129	-0.8037 to 1.430	No	ns	0.9999
Emp1	Mature PCC vs. P-I CC	6.654	3.156 to 10.15	Yes	****	<0.0001
Emp1	Mature PCC vs. Pro-Senescent CC	7.025	5.670 to 8.379	Yes	****	<0.0001
Emp1	P-I CC vs. Pro-Senescent CC	0.3703	-3.140 to 3.880	No	ns	>0.9999
Emp1	Sec Prog vs. Paneth-like	0.1563	-1.370 to 1.683	No	ns	>0.9999
Emp1	Sec Prog vs. Goblet	0.1376	-1.203 to 1.478	No	ns	>0.9999
Emp1	Sec Prog vs. Endocrine	0.1898	-2.374 to 2.754	No	ns	>0.9999
Emp1	Sec Prog vs. Tuft	0.1651	-2.041 to 2.371	No	ns	>0.9999
Emp1	Sec Prog vs. Abs Prog	-0.1767	-2.836 to 2.483	No	ns	>0.9999
Emp1	Sec Prog vs. Pre-DCC	-0.06843	-1.765 to 1.628	No	ns	>0.9999
Emp1	Sec Prog vs. Pre-PCC	-47.34	-49.39 to -45.28	Yes	****	<0.0001
Emp1	Sec Prog vs. Pro-DCC	-0.3503	-1.744 to 1.044	No	ns	>0.9999
Emp1	Sec Prog vs. Mature DCC	-0.2696	-1.531 to 0.9917	No	ns	>0.9999
Emp1	Sec Prog vs. Mature PCC	-6.981	-8.457 to -5.505	Yes	****	<0.0001
Emp1	Sec Prog vs. P-I CC	-0.3269	-3.886 to 3.232	No	ns	>0.9999
Emp1	Sec Prog vs. Pro-Senescent CC	0.04335	-1.461 to 1.548	No	ns	>0.9999
Emp1	Paneth-like vs. Goblet	-0.01864	-1.252 to 1.215	No	ns	>0.9999
Emp1	Paneth-like vs. Endocrine	0.03353	-2.476 to 2.543	No	ns	>0.9999
Emp1	Paneth-like vs. Tuft	0.008807	-2.134 to 2.152	No	ns	>0.9999
Emp1	Paneth-like vs. Abs Prog	-0.3329	-2.940 to 2.274	No	ns	>0.9999
Emp1	Paneth-like vs. Pre-DCC	-0.2247	-1.837 to 1.388	No	ns	>0.9999
Emp1	Paneth-like vs. Pre-PCC	-47.49	-49.48 to -45.50	Yes	****	<0.0001
Emp1	Paneth-like vs. Pro-DCC	-0.5066	-1.798 to 0.7846	No	ns	0.9942
Emp1	Paneth-like vs. Mature DCC	-0.4259	-1.572 to 0.7207	No	ns	0.9968
Emp1	Paneth-like vs. Mature PCC	-7.138	-8.517 to -5.758	Yes	****	<0.0001
Emp1	Paneth-like vs. P-I CC	-0.4832	-4.003 to 3.037	No	ns	>0.9999
Emp1	Paneth-like vs. Pro-Senescent CC	-0.1129	-1.522 to 1.296	No	ns	>0.9999
Emp1	Goblet vs. Endocrine	0.05217	-2.349 to 2.453	No	ns	>0.9999
Emp1	Goblet vs. Tuft	0.02745	-1.987 to 2.042	No	ns	>0.9999
Emp1	Goblet vs. Abs Prog	-0.3143	-2.817 to 2.189	No	ns	>0.9999
Emp1	Goblet vs. Pre-DCC	-0.2061	-1.644 to 1.232	No	ns	>0.9999
Emp1	Goblet vs. Pre-PCC	-47.47	-49.32 to -45.62	Yes	****	<0.0001
Emp1	Goblet vs. Pro-DCC	-0.4879	-1.553 to 0.5774	No	ns	0.9736
Emp1	Goblet vs. Mature DCC	-0.4072	-1.292 to 0.4773	No	ns	0.9723
Emp1	Goblet vs. Mature PCC	-7.119	-8.290 to -5.948	Yes	****	<0.0001
Emp1	Goblet vs. P-I CC	-0.4646	-3.908 to 2.979	No	ns	>0.9999
Emp1	Goblet vs. Pro-Senescent CC	-0.09428	-1.300 to 1.112	No	ns	>0.9999
Emp1	Endocrine vs. Tuft	-0.02472	-2.997 to 2.947	No	ns	>0.9999
Emp1	Endocrine vs. Abs Prog	-0.3665	-3.689 to 2.956	No	ns	>0.9999
Emp1	Endocrine vs. Pre-DCC	-0.2582	-2.874 to 2.358	No	ns	>0.9999
Emp1	Endocrine vs. Pre-PCC	-47.53	-50.39 to -44.66	Yes	****	<0.0001
Emp1	Endocrine vs. Pro-DCC	-0.5401	-2.971 to 1.891	No	ns	>0.9999
Emp1	Endocrine vs. Mature DCC	-0.4594	-2.817 to 1.898	No	ns	>0.9999
Emp1	Endocrine vs. Mature PCC	-7.171	-9.650 to -4.692	Yes	****	<0.0001
Emp1	Endocrine vs. P-I CC	-0.5168	-4.595 to 3.561	No	ns	>0.9999
Emp1	Endocrine vs. Pro-Senescent CC	-0.1465	-2.642 to 2.349	No	ns	>0.9999
Emp1	Tuft vs. Abs Prog	-0.3417	-3.397 to 2.714	No	ns	>0.9999
Emp1	Tuft vs. Pre-DCC	-0.2335	-2.500 to 2.033	No	ns	>0.9999
Emp1	Tuft vs. Pre-PCC	-47.50	-50.05 to -44.95	Yes	****	<0.0001
Emp1	Tuft vs. Pro-DCC	-0.5154	-2.566 to 1.535	No	ns	>0.9999
Emp1	Tuft vs. Mature DCC	-0.4347	-2.397 to 1.528	No	ns	>0.9999
Emp1	Tuft vs. Mature PCC	-7.146	-9.254 to -5.039	Yes	****	<0.0001
Emp1	Tuft vs. P-I CC	-0.4920	-4.356 to 3.371	No	ns	>0.9999
Emp1	Tuft vs. Pro-Senescent CC	-0.1217	-2.249 to 2.005	No	ns	>0.9999

Supplementary Table 5. One-way ANOVA of IL-22–inducible genes from naïve BL/6 mice shown in Figure ED6b.

Gene	Tukey's multiple comparisons test	Mean Diff.	95.00% CI of diff.	Below threshold?	Summary	Adjusted P Value
S100a8	Lgr5-hi vs. Lgr5-dim	0.000	-0.01969 to 0.01969	No	ns	>0.9999
S100a8	Lgr5-hi vs. Early TA	0.000	-0.02695 to 0.02695	No	ns	>0.9999
S100a8	Lgr5-hi vs. Abs Prog	-0.01613	-0.04138 to 0.009125	No	ns	0.7050
S100a8	Lgr5-hi vs. Pre-DCC	-0.004329	-0.02032 to 0.01166	No	ns	>0.9999
S100a8	Lgr5-hi vs. Pre-PCC	-0.01613	-0.03554 to 0.003279	No	ns	0.2419
S100a8	Lgr5-hi vs. Pro-DCC	0.000	-0.01314 to 0.01314	No	ns	>0.9999
S100a8	Lgr5-hi vs. Mature DCC	-0.003167	-0.01505 to 0.008718	No	ns	>0.9999
S100a8	Lgr5-hi vs. Mature PCC	-0.004831	-0.01875 to 0.009091	No	ns	0.9985
S100a8	Lgr5-hi vs. P-I CC	0.000	-0.03357 to 0.03357	No	ns	>0.9999
S100a8	Lgr5-hi vs. Pro-Senescent CC	-0.002639	-0.01682 to 0.01154	No	ns	>0.9999
S100a8	Lgr5-hi vs. Sec Prog	0.000	-0.01522 to 0.01522	No	ns	>0.9999
S100a8	Lgr5-hi vs. Paneth-like	0.000	-0.01439 to 0.01439	No	ns	>0.9999
S100a8	Lgr5-hi vs. Goblet	0.000	-0.01264 to 0.01264	No	ns	>0.9999
S100a8	Lgr5-hi vs. EEC	-0.01449	-0.03867 to 0.009689	No	ns	0.7940
S100a8	Lgr5-hi vs. Tuft	0.000	-0.02081 to 0.02081	No	ns	>0.9999
S100a8	Lgr5-dim vs. Early TA	0.000	-0.02971 to 0.02971	No	ns	>0.9999
S100a8	Lgr5-dim vs. Abs Prog	-0.01613	-0.04431 to 0.01205	No	ns	0.8462
S100a8	Lgr5-dim vs. Pre-DCC	-0.004329	-0.02463 to 0.01597	No	ns	>0.9999
S100a8	Lgr5-dim vs. Pre-PCC	-0.01613	-0.03922 to 0.006960	No	ns	0.5518
S100a8	Lgr5-dim vs. Pro-DCC	0.000	-0.01814 to 0.01814	No	ns	>0.9999
S100a8	Lgr5-dim vs. Mature DCC	-0.003167	-0.02042 to 0.01409	No	ns	>0.9999
S100a8	Lgr5-dim vs. Mature PCC	-0.004831	-0.02355 to 0.01388	No	ns	>0.9999
S100a8	Lgr5-dim vs. P-I CC	0.000	-0.03583 to 0.03583	No	ns	>0.9999
S100a8	Lgr5-dim vs. Pro-Senescent CC	-0.002639	-0.02154 to 0.01627	No	ns	>0.9999
S100a8	Lgr5-dim vs. Sec Prog	0.000	-0.01970 to 0.01970	No	ns	>0.9999
S100a8	Lgr5-dim vs. Paneth-like	0.000	-0.01907 to 0.01907	No	ns	>0.9999
S100a8	Lgr5-dim vs. Goblet	0.000	-0.01778 to 0.01778	No	ns	>0.9999
S100a8	Lgr5-dim vs. EEC	-0.01449	-0.04172 to 0.01273	No	ns	0.9077
S100a8	Lgr5-dim vs. Tuft	0.000	-0.02428 to 0.02428	No	ns	>0.9999
S100a8	Early TA vs. Abs Prog	-0.01613	-0.04979 to 0.01753	No	ns	0.9605
S100a8	Early TA vs. Pre-DCC	-0.004329	-0.03173 to 0.02307	No	ns	>0.9999
S100a8	Early TA vs. Pre-PCC	-0.01613	-0.04566 to 0.01340	No	ns	0.8885
S100a8	Early TA vs. Pro-DCC	0.000	-0.02584 to 0.02584	No	ns	>0.9999
S100a8	Early TA vs. Mature DCC	-0.003167	-0.02839 to 0.02206	No	ns	>0.9999
S100a8	Early TA vs. Mature PCC	-0.004831	-0.03108 to 0.02142	No	ns	>0.9999
S100a8	Early TA vs. P-I CC	0.000	-0.04028 to 0.04028	No	ns	>0.9999
S100a8	Early TA vs. Pro-Senescent CC	-0.002639	-0.02902 to 0.02375	No	ns	>0.9999
S100a8	Early TA vs. Sec Prog	0.000	-0.02696 to 0.02696	No	ns	>0.9999
S100a8	Early TA vs. Paneth-like	0.000	-0.02650 to 0.02650	No	ns	>0.9999
S100a8	Early TA vs. Goblet	0.000	-0.02559 to 0.02559	No	ns	>0.9999
S100a8	Early TA vs. EEC	-0.01449	-0.04736 to 0.01837	No	ns	0.9814
S100a8	Early TA vs. Tuft	0.000	-0.03047 to 0.03047	No	ns	>0.9999
S100a8	Abs Prog vs. Pre-DCC	0.01180	-0.01393 to 0.03753	No	ns	0.9733
S100a8	Abs Prog vs. Pre-PCC	0.000	-0.02799 to 0.02799	No	ns	>0.9999
S100a8	Abs Prog vs. Pro-DCC	0.01613	-0.007936 to 0.04019	No	ns	0.6258
S100a8	Abs Prog vs. Mature DCC	0.01296	-0.01044 to 0.03637	No	ns	0.8771
S100a8	Abs Prog vs. Mature PCC	0.01130	-0.01320 to 0.03580	No	ns	0.9719
S100a8	Abs Prog vs. P-I CC	0.01613	-0.02303 to 0.05529	No	ns	0.9905
S100a8	Abs Prog vs. Pro-Senescent CC	0.01349	-0.01116 to 0.03814	No	ns	0.8870
S100a8	Abs Prog vs. Sec Prog	0.01613	-0.009133 to 0.04139	No	ns	0.7055
S100a8	Abs Prog vs. Paneth-like	0.01613	-0.008641 to 0.04090	No	ns	0.6743
S100a8	Abs Prog vs. Goblet	0.01613	-0.007665 to 0.03992	No	ns	0.6060
S100a8	Abs Prog vs. EEC	0.001636	-0.02985 to 0.03312	No	ns	>0.9999
S100a8	Abs Prog vs. Tuft	0.01613	-0.01285 to 0.04510	No	ns	0.8726
S100a8	Pre-DCC vs. Pre-PCC	-0.01180	-0.03183 to 0.008230	No	ns	0.8147
S100a8	Pre-DCC vs. Pro-DCC	0.004329	-0.009711 to 0.01837	No	ns	0.9996
S100a8	Pre-DCC vs. Mature DCC	0.001162	-0.01171 to 0.01404	No	ns	>0.9999

S100a8	Pre-DCC vs. Mature PCC	-0.0005019	-0.01528 to 0.01427	No	ns	>0.9999
S100a8	Pre-DCC vs. P-I CC	0.004329	-0.02961 to 0.03827	No	ns	>0.9999
S100a8	Pre-DCC vs. Pro-Senescent CC	0.001690	-0.01333 to 0.01671	No	ns	>0.9999
S100a8	Pre-DCC vs. Sec Prog	0.004329	-0.01168 to 0.02033	No	ns	>0.9999
S100a8	Pre-DCC vs. Paneth-like	0.004329	-0.01089 to 0.01955	No	ns	0.9999
S100a8	Pre-DCC vs. Goblet	0.004329	-0.009242 to 0.01790	No	ns	0.9994
S100a8	Pre-DCC vs. EEC	-0.01016	-0.03485 to 0.01452	No	ns	0.9905
S100a8	Pre-DCC vs. Tuft	0.004329	-0.01706 to 0.02572	No	ns	>0.9999
S100a8	Pre-PCC vs. Pro-DCC	0.01613	-0.001705 to 0.03396	No	ns	0.1305
S100a8	Pre-PCC vs. Mature DCC	0.01296	-0.003970 to 0.02989	No	ns	0.3817
S100a8	Pre-PCC vs. Mature PCC	0.01130	-0.007121 to 0.02972	No	ns	0.7637
S100a8	Pre-PCC vs. P-I CC	0.01613	-0.01955 to 0.05180	No	ns	0.9765
S100a8	Pre-PCC vs. Pro-Senescent CC	0.01349	-0.005123 to 0.03210	No	ns	0.4835
S100a8	Pre-PCC vs. Sec Prog	0.01613	-0.003290 to 0.03555	No	ns	0.2427
S100a8	Pre-PCC vs. Paneth-like	0.01613	-0.002646 to 0.03490	No	ns	0.1936
S100a8	Pre-PCC vs. Goblet	0.01613	-0.001338 to 0.03360	No	ns	0.1093
S100a8	Pre-PCC vs. EEC	0.001636	-0.02539 to 0.02866	No	ns	>0.9999
S100a8	Pre-PCC vs. Tuft	0.01613	-0.007922 to 0.04018	No	ns	0.6247
S100a8	Pro-DCC vs. Mature DCC	-0.003167	-0.01226 to 0.005923	No	ns	0.9984
S100a8	Pro-DCC vs. Mature PCC	-0.004831	-0.01646 to 0.006796	No	ns	0.9896
S100a8	Pro-DCC vs. P-I CC	0.000	-0.03269 to 0.03269	No	ns	>0.9999
S100a8	Pro-DCC vs. Pro-Senescent CC	-0.002639	-0.01457 to 0.009295	No	ns	>0.9999
S100a8	Pro-DCC vs. Sec Prog	0.000	-0.01315 to 0.01315	No	ns	>0.9999
S100a8	Pro-DCC vs. Paneth-like	0.000	-0.01218 to 0.01218	No	ns	>0.9999
S100a8	Pro-DCC vs. Goblet	0.000	-0.01005 to 0.01005	No	ns	>0.9999
S100a8	Pro-DCC vs. EEC	-0.01449	-0.03743 to 0.008445	No	ns	0.7214
S100a8	Pro-DCC vs. Tuft	0.000	-0.01935 to 0.01935	No	ns	>0.9999
S100a8	Mature DCC vs. Mature PCC	-0.001664	-0.01185 to 0.008526	No	ns	>0.9999
S100a8	Mature DCC vs. P-I CC	0.003167	-0.02904 to 0.03537	No	ns	>0.9999
S100a8	Mature DCC vs. Pro-Senescent CC	0.0005285	-0.01001 to 0.01107	No	ns	>0.9999
S100a8	Mature DCC vs. Sec Prog	0.003167	-0.008735 to 0.01507	No	ns	>0.9999
S100a8	Mature DCC vs. Paneth-like	0.003167	-0.007653 to 0.01399	No	ns	0.9998
S100a8	Mature DCC vs. Goblet	0.003167	-0.005180 to 0.01151	No	ns	0.9959
S100a8	Mature DCC vs. EEC	-0.01133	-0.03357 to 0.01092	No	ns	0.9346
S100a8	Mature DCC vs. Tuft	0.003167	-0.01535 to 0.02169	No	ns	>0.9999
S100a8	Mature PCC vs. P-I CC	0.004831	-0.02818 to 0.03784	No	ns	>0.9999
S100a8	Mature PCC vs. Pro-Senescent CC	0.002192	-0.01060 to 0.01498	No	ns	>0.9999
S100a8	Mature PCC vs. Sec Prog	0.004831	-0.009105 to 0.01877	No	ns	0.9985
S100a8	Mature PCC vs. Paneth-like	0.004831	-0.008194 to 0.01786	No	ns	0.9968
S100a8	Mature PCC vs. Goblet	0.004831	-0.006225 to 0.01589	No	ns	0.9830
S100a8	Mature PCC vs. EEC	-0.009662	-0.03306 to 0.01373	No	ns	0.9902
S100a8	Mature PCC vs. Tuft	0.004831	-0.01506 to 0.02472	No	ns	>0.9999
S100a8	P-I CC vs. Pro-Senescent CC	-0.002639	-0.03576 to 0.03048	No	ns	>0.9999
S100a8	P-I CC vs. Sec Prog	0.000	-0.03358 to 0.03358	No	ns	>0.9999
S100a8	P-I CC vs. Paneth-like	0.000	-0.03321 to 0.03321	No	ns	>0.9999
S100a8	P-I CC vs. Goblet	0.000	-0.03249 to 0.03249	No	ns	>0.9999
S100a8	P-I CC vs. EEC	-0.01449	-0.05297 to 0.02399	No	ns	0.9962
S100a8	P-I CC vs. Tuft	0.000	-0.03646 to 0.03646	No	ns	>0.9999
S100a8	Pro-Senescent CC vs. Sec Prog	0.002639	-0.01155 to 0.01683	No	ns	>0.9999
S100a8	Pro-Senescent CC vs. Paneth-like	0.002639	-0.01066 to 0.01594	No	ns	>0.9999
S100a8	Pro-Senescent CC vs. Goblet	0.002639	-0.008739 to 0.01402	No	ns	>0.9999
S100a8	Pro-Senescent CC vs. EEC	-0.01185	-0.03540 to 0.01170	No	ns	0.9404
S100a8	Pro-Senescent CC vs. Tuft	0.002639	-0.01743 to 0.02271	No	ns	>0.9999
S100a8	Sec Prog vs. Paneth-like	0.000	-0.01440 to 0.01440	No	ns	>0.9999
S100a8	Sec Prog vs. Goblet	0.000	-0.01265 to 0.01265	No	ns	>0.9999
S100a8	Sec Prog vs. EEC	-0.01449	-0.03868 to 0.009698	No	ns	0.7944
S100a8	Sec Prog vs. Tuft	0.000	-0.02082 to 0.02082	No	ns	>0.9999

S100a8	Paneth-like vs. Goblet	0.000	-0.01164 to 0.01164	No	ns	>0.9999
S100a8	Paneth-like vs. EEC	-0.01449	-0.03817 to 0.009184	No	ns	0.7666
S100a8	Paneth-like vs. Tuft	0.000	-0.02022 to 0.02022	No	ns	>0.9999
S100a8	Goblet vs. EEC	-0.01449	-0.03715 to 0.008161	No	ns	0.7024
S100a8	Goblet vs. Tuft	0.000	-0.01901 to 0.01901	No	ns	>0.9999
S100a8	EEC vs. Tuft	0.01449	-0.01355 to 0.04254	No	ns	0.9265

Gene	Tukey's multiple comparisons test	Mean Diff.	95.00% CI of diff.	Below threshold?	Summary	Adjusted P Value
Cxcl2	Lgr5-hi vs. Lgr5-dim	-0.5603	-0.9715 to -0.1490	Yes	***	0.0003
Cxcl2	Lgr5-hi vs. Early TA	-0.08241	-0.6454 to 0.4806	No	ns	>0.9999
Cxcl2	Lgr5-hi vs. Abs Prog	-0.1591	-0.6866 to 0.3684	No	ns	0.9997
Cxcl2	Lgr5-hi vs. Pre-DCC	0.004004	-0.3300 to 0.3380	No	ns	>0.9999
Cxcl2	Lgr5-hi vs. Pre-PCC	0.3812	-0.02417 to 0.7866	No	ns	0.0928
Cxcl2	Lgr5-hi vs. Pro-DCC	0.3241	0.04972 to 0.5986	Yes	**	0.0053
Cxcl2	Lgr5-hi vs. Mature DCC	0.3426	0.09433 to 0.5908	Yes	***	0.0003
Cxcl2	Lgr5-hi vs. Mature PCC	0.3845	0.09367 to 0.6752	Yes	***	0.0007
Cxcl2	Lgr5-hi vs. P-I CC	0.3580	-0.3433 to 1.059	No	ns	0.9332
Cxcl2	Lgr5-hi vs. Pro-Senescent CC	0.3708	0.07466 to 0.6670	Yes	**	0.0019
Cxcl2	Lgr5-hi vs. Sec Prog	0.3355	0.01761 to 0.6534	Yes	*	0.0266
Cxcl2	Lgr5-hi vs. Paneth-like	0.3554	0.05482 to 0.6560	Yes	**	0.0052
Cxcl2	Lgr5-hi vs. Goblet	0.3852	0.1213 to 0.6491	Yes	****	<0.0001
Cxcl2	Lgr5-hi vs. EEC	0.3893	-0.1158 to 0.8944	No	ns	0.3694
Cxcl2	Lgr5-hi vs. Tuft	0.3893	-0.04535 to 0.8239	No	ns	0.1413
Cxcl2	Lgr5-dim vs. Early TA	0.4779	-0.1427 to 1.099	No	ns	0.3711
Cxcl2	Lgr5-dim vs. Abs Prog	0.4012	-0.1874 to 0.9898	No	ns	0.5963
Cxcl2	Lgr5-dim vs. Pre-DCC	0.5643	0.1402 to 0.9884	Yes	***	0.0006
Cxcl2	Lgr5-dim vs. Pre-PCC	0.9415	0.4592 to 1.424	Yes	****	<0.0001
Cxcl2	Lgr5-dim vs. Pro-DCC	0.8844	0.5056 to 1.263	Yes	****	<0.0001
Cxcl2	Lgr5-dim vs. Mature DCC	0.9029	0.5425 to 1.263	Yes	****	<0.0001
Cxcl2	Lgr5-dim vs. Mature PCC	0.9447	0.5538 to 1.336	Yes	****	<0.0001
Cxcl2	Lgr5-dim vs. P-I CC	0.9183	0.1700 to 1.667	Yes	**	0.0028
Cxcl2	Lgr5-dim vs. Pro-Senescent CC	0.9311	0.5362 to 1.326	Yes	****	<0.0001
Cxcl2	Lgr5-dim vs. Sec Prog	0.8958	0.4843 to 1.307	Yes	****	<0.0001
Cxcl2	Lgr5-dim vs. Paneth-like	0.9157	0.5175 to 1.314	Yes	****	<0.0001
Cxcl2	Lgr5-dim vs. Goblet	0.9455	0.5741 to 1.317	Yes	****	<0.0001
Cxcl2	Lgr5-dim vs. EEC	0.9496	0.3809 to 1.518	Yes	****	<0.0001
Cxcl2	Lgr5-dim vs. Tuft	0.9496	0.4425 to 1.457	Yes	****	<0.0001
Cxcl2	Early TA vs. Abs Prog	-0.07669	-0.7797 to 0.6264	No	ns	>0.9999
Cxcl2	Early TA vs. Pre-DCC	0.08642	-0.4860 to 0.6588	No	ns	>0.9999
Cxcl2	Early TA vs. Pre-PCC	0.4636	-0.1531 to 1.080	No	ns	0.4152
Cxcl2	Early TA vs. Pro-DCC	0.4066	-0.1332 to 0.9463	No	ns	0.4116
Cxcl2	Early TA vs. Mature DCC	0.4250	-0.1020 to 0.9519	No	ns	0.2901
Cxcl2	Early TA vs. Mature PCC	0.4669	-0.08141 to 1.015	No	ns	0.2058
Cxcl2	Early TA vs. P-I CC	0.4404	-0.4009 to 1.282	No	ns	0.9187
Cxcl2	Early TA vs. Pro-Senescent CC	0.4532	-0.09791 to 1.004	No	ns	0.2583
Cxcl2	Early TA vs. Sec Prog	0.4179	-0.1452 to 0.9811	No	ns	0.4390
Cxcl2	Early TA vs. Paneth-like	0.4378	-0.1157 to 0.9913	No	ns	0.3233
Cxcl2	Early TA vs. Goblet	0.4676	-0.06690 to 1.002	No	ns	0.1695
Cxcl2	Early TA vs. EEC	0.4717	-0.2147 to 1.158	No	ns	0.5817
Cxcl2	Early TA vs. Tuft	0.4717	-0.1647 to 1.108	No	ns	0.4413
Cxcl2	Abs Prog vs. Pre-DCC	0.1631	-0.3744 to 0.7006	No	ns	0.9997
Cxcl2	Abs Prog vs. Pre-PCC	0.5403	-0.04423 to 1.125	No	ns	0.1084
Cxcl2	Abs Prog vs. Pro-DCC	0.4832	-0.01942 to 0.9859	No	ns	0.0752
Cxcl2	Abs Prog vs. Mature DCC	0.5017	0.01281 to 0.9905	Yes	*	0.0374
Cxcl2	Abs Prog vs. Mature PCC	0.5436	0.03178 to 1.055	Yes	*	0.0246
Cxcl2	Abs Prog vs. P-I CC	0.5171	-0.3009 to 1.335	No	ns	0.7206
Cxcl2	Abs Prog vs. Pro-Senescent CC	0.5299	0.01507 to 1.045	Yes	*	0.0361
Cxcl2	Abs Prog vs. Sec Prog	0.4946	-0.03304 to 1.022	No	ns	0.0955
Cxcl2	Abs Prog vs. Paneth-like	0.5145	-0.002910 to 1.032	No	ns	0.0531
Cxcl2	Abs Prog vs. Goblet	0.5443	0.04729 to 1.041	Yes	*	0.0164
Cxcl2	Abs Prog vs. EEC	0.5484	-0.1093 to 1.206	No	ns	0.2367
Cxcl2	Abs Prog vs. Tuft	0.5484	-0.05682 to 1.154	No	ns	0.1285

Cxcl2	Pre-DCC vs. Pre-PCC	0.3772	-0.04117 to 0.7956	No	ns	0.1339
Cxcl2	Pre-DCC vs. Pro-DCC	0.3201	0.02687 to 0.6134	Yes	*	0.0172
Cxcl2	Pre-DCC vs. Mature DCC	0.3386	0.06963 to 0.6075	Yes	**	0.0017
Cxcl2	Pre-DCC vs. Mature PCC	0.3805	0.07181 to 0.6891	Yes	**	0.0026
Cxcl2	Pre-DCC vs. P-I CC	0.3540	-0.3548 to 1.063	No	ns	0.9441
Cxcl2	Pre-DCC vs. Pro-Senescent CC	0.3668	0.05311 to 0.6805	Yes	**	0.0062
Cxcl2	Pre-DCC vs. Sec Prog	0.3315	-0.002795 to 0.6658	No	ns	0.0547
Cxcl2	Pre-DCC vs. Paneth-like	0.3514	0.03352 to 0.6693	Yes	*	0.0144
Cxcl2	Pre-DCC vs. Goblet	0.3812	0.09772 to 0.6647	Yes	***	0.0005
Cxcl2	Pre-DCC vs. EEC	0.3853	-0.1303 to 0.9009	No	ns	0.4263
Cxcl2	Pre-DCC vs. Tuft	0.3853	-0.06150 to 0.8321	No	ns	0.1884
Cxcl2	Pre-PCC vs. Pro-DCC	-0.05708	-0.4296 to 0.3154	No	ns	>0.9999
Cxcl2	Pre-PCC vs. Mature DCC	-0.03865	-0.3923 to 0.3150	No	ns	>0.9999
Cxcl2	Pre-PCC vs. Mature PCC	0.003234	-0.3815 to 0.3880	No	ns	>0.9999
Cxcl2	Pre-PCC vs. P-I CC	-0.02319	-0.7684 to 0.7220	No	ns	>0.9999
Cxcl2	Pre-PCC vs. Pro-Senescent CC	-0.01041	-0.3992 to 0.3784	No	ns	>0.9999
Cxcl2	Pre-PCC vs. Sec Prog	-0.04570	-0.4513 to 0.3599	No	ns	>0.9999
Cxcl2	Pre-PCC vs. Paneth-like	-0.02583	-0.4180 to 0.3663	No	ns	>0.9999
Cxcl2	Pre-PCC vs. Goblet	0.003983	-0.3609 to 0.3688	No	ns	>0.9999
Cxcl2	Pre-PCC vs. EEC	0.008065	-0.5564 to 0.5725	No	ns	>0.9999
Cxcl2	Pre-PCC vs. Tuft	0.008065	-0.4943 to 0.5104	No	ns	>0.9999
Cxcl2	Pro-DCC vs. Mature DCC	0.01843	-0.1714 to 0.2083	No	ns	>0.9999
Cxcl2	Pro-DCC vs. Mature PCC	0.06031	-0.1826 to 0.3032	No	ns	>0.9999
Cxcl2	Pro-DCC vs. P-I CC	0.03389	-0.6489 to 0.7167	No	ns	>0.9999
Cxcl2	Pro-DCC vs. Pro-Senescent CC	0.04667	-0.2026 to 0.2959	No	ns	>0.9999
Cxcl2	Pro-DCC vs. Sec Prog	0.01138	-0.2634 to 0.2861	No	ns	>0.9999
Cxcl2	Pro-DCC vs. Paneth-like	0.03124	-0.2232 to 0.2857	No	ns	>0.9999
Cxcl2	Pro-DCC vs. Goblet	0.06106	-0.1489 to 0.2710	No	ns	0.9998
Cxcl2	Pro-DCC vs. EEC	0.06514	-0.4140 to 0.5443	No	ns	>0.9999
Cxcl2	Pro-DCC vs. Tuft	0.06514	-0.3390 to 0.4693	No	ns	>0.9999
Cxcl2	Mature DCC vs. Mature PCC	0.04188	-0.1710 to 0.2547	No	ns	>0.9999
Cxcl2	Mature DCC vs. P-I CC	0.01546	-0.6573 to 0.6882	No	ns	>0.9999
Cxcl2	Mature DCC vs. Pro-Senescent CC	0.02824	-0.1919 to 0.2484	No	ns	>0.9999
Cxcl2	Mature DCC vs. Sec Prog	-0.007049	-0.2557 to 0.2416	No	ns	>0.9999
Cxcl2	Mature DCC vs. Paneth-like	0.01282	-0.2132 to 0.2388	No	ns	>0.9999
Cxcl2	Mature DCC vs. Goblet	0.04263	-0.1317 to 0.2170	No	ns	>0.9999
Cxcl2	Mature DCC vs. EEC	0.04671	-0.4179 to 0.5113	No	ns	>0.9999
Cxcl2	Mature DCC vs. Tuft	0.04671	-0.3401 to 0.4336	No	ns	>0.9999
Cxcl2	Mature PCC vs. P-I CC	-0.02642	-0.7160 to 0.6631	No	ns	>0.9999
Cxcl2	Mature PCC vs. Pro-Senescent CC	-0.01364	-0.2808 to 0.2535	No	ns	>0.9999
Cxcl2	Mature PCC vs. Sec Prog	-0.04893	-0.3400 to 0.2422	No	ns	>0.9999
Cxcl2	Mature PCC vs. Paneth-like	-0.02907	-0.3011 to 0.2430	No	ns	>0.9999
Cxcl2	Mature PCC vs. Goblet	0.0007493	-0.2302 to 0.2317	No	ns	>0.9999
Cxcl2	Mature PCC vs. EEC	0.004831	-0.4838 to 0.4935	No	ns	>0.9999
Cxcl2	Mature PCC vs. Tuft	0.004831	-0.4106 to 0.4203	No	ns	>0.9999
Cxcl2	P-I CC vs. Pro-Senescent CC	0.01278	-0.6791 to 0.7046	No	ns	>0.9999
Cxcl2	P-I CC vs. Sec Prog	-0.02251	-0.7239 to 0.6789	No	ns	>0.9999
Cxcl2	P-I CC vs. Paneth-like	-0.002648	-0.6964 to 0.6911	No	ns	>0.9999
Cxcl2	P-I CC vs. Goblet	0.02717	-0.6515 to 0.7058	No	ns	>0.9999
Cxcl2	P-I CC vs. EEC	0.03125	-0.7725 to 0.8350	No	ns	>0.9999
Cxcl2	P-I CC vs. Tuft	0.03125	-0.7302 to 0.7927	No	ns	>0.9999
Cxcl2	Pro-Senescent CC vs. Sec Prog	-0.03529	-0.3318 to 0.2612	No	ns	>0.9999
Cxcl2	Pro-Senescent CC vs. Paneth-like	-0.01543	-0.2932 to 0.2624	No	ns	>0.9999
Cxcl2	Pro-Senescent CC vs. Goblet	0.01439	-0.2233 to 0.2520	No	ns	>0.9999
Cxcl2	Pro-Senescent CC vs. EEC	0.01847	-0.4734 to 0.5104	No	ns	>0.9999
Cxcl2	Pro-Senescent CC vs. Tuft	0.01847	-0.4007 to 0.4377	No	ns	>0.9999
Cxcl2	Sec Prog vs. Paneth-like	0.01987	-0.2810 to 0.3207	No	ns	>0.9999

Cxcl2	Sec Prog vs. Goblet	0.04968	-0.2146 to 0.3140	No	ns	>0.9999
Cxcl2	Sec Prog vs. EEC	0.05376	-0.4515 to 0.5591	No	ns	>0.9999
Cxcl2	Sec Prog vs. Tuft	0.05376	-0.3811 to 0.4886	No	ns	>0.9999
Cxcl2	Paneth-like vs. Goblet	0.02982	-0.2133 to 0.2730	No	ns	>0.9999
Cxcl2	Paneth-like vs. EEC	0.03390	-0.4607 to 0.5285	No	ns	>0.9999
Cxcl2	Paneth-like vs. Tuft	0.03390	-0.3884 to 0.4562	No	ns	>0.9999
Cxcl2	Goblet vs. EEC	0.004082	-0.4691 to 0.4773	No	ns	>0.9999
Cxcl2	Goblet vs. Tuft	0.004082	-0.3930 to 0.4012	No	ns	>0.9999
Cxcl2	EEC vs. Tuft	0.000	-0.5858 to 0.5858	No	ns	>0.9999

Gene	Tukey's multiple comparisons test	Mean Diff.	95.00% CI of diff.	Below threshold?	Summary	Adjusted P Value
Cxcl5	Lgr5-hi vs. Lgr5-dim	-0.02710	-0.1958 to 0.1416	No	ns	>0.9999
Cxcl5	Lgr5-hi vs. Early TA	-0.09575	-0.3267 to 0.1352	No	ns	0.9898
Cxcl5	Lgr5-hi vs. Abs Prog	-0.1621	-0.3785 to 0.05433	No	ns	0.4220
Cxcl5	Lgr5-hi vs. Pre-DCC	0.07781	-0.05924 to 0.2149	No	ns	0.8542
Cxcl5	Lgr5-hi vs. Pre-PCC	0.1766	0.01028 to 0.3429	Yes	*	0.0247
Cxcl5	Lgr5-hi vs. Pro-DCC	0.07887	-0.03372 to 0.1915	No	ns	0.5467
Cxcl5	Lgr5-hi vs. Mature DCC	0.08723	-0.01462 to 0.1891	No	ns	0.1977
Cxcl5	Lgr5-hi vs. Mature PCC	0.1984	0.07912 to 0.3177	Yes	****	<0.0001
Cxcl5	Lgr5-hi vs. P-I CC	-0.05625	-0.3440 to 0.2315	No	ns	>0.9999
Cxcl5	Lgr5-hi vs. Pro-Senescent CC	0.1907	0.06919 to 0.3122	Yes	****	<0.0001
Cxcl5	Lgr5-hi vs. Sec Prog	0.1461	0.01571 to 0.2766	Yes	*	0.0119
Cxcl5	Lgr5-hi vs. Paneth-like	0.1826	0.05931 to 0.3059	Yes	****	<0.0001
Cxcl5	Lgr5-hi vs. Goblet	0.2209	0.1126 to 0.3292	Yes	****	<0.0001
Cxcl5	Lgr5-hi vs. EEC	0.2250	0.01776 to 0.4322	Yes	*	0.0185
Cxcl5	Lgr5-hi vs. Tuft	0.2152	0.03687 to 0.3935	Yes	**	0.0037
Cxcl5	Lgr5-dim vs. Early TA	-0.06865	-0.3233 to 0.1860	No	ns	>0.9999
Cxcl5	Lgr5-dim vs. Abs Prog	-0.1350	-0.3765 to 0.1065	No	ns	0.8689
Cxcl5	Lgr5-dim vs. Pre-DCC	0.1049	-0.06907 to 0.2789	No	ns	0.7863
Cxcl5	Lgr5-dim vs. Pre-PCC	0.2037	0.005842 to 0.4016	Yes	*	0.0360
Cxcl5	Lgr5-dim vs. Pro-DCC	0.1060	-0.04948 to 0.2614	No	ns	0.5959
Cxcl5	Lgr5-dim vs. Mature DCC	0.1143	-0.03352 to 0.2622	No	ns	0.3634
Cxcl5	Lgr5-dim vs. Mature PCC	0.2255	0.06515 to 0.3859	Yes	***	0.0002
Cxcl5	Lgr5-dim vs. P-I CC	-0.02915	-0.3362 to 0.2779	No	ns	>0.9999
Cxcl5	Lgr5-dim vs. Pro-Senescent CC	0.2178	0.05577 to 0.3798	Yes	***	0.0005
Cxcl5	Lgr5-dim vs. Sec Prog	0.1732	0.004425 to 0.3421	Yes	*	0.0374
Cxcl5	Lgr5-dim vs. Paneth-like	0.2097	0.04634 to 0.3731	Yes	**	0.0012
Cxcl5	Lgr5-dim vs. Goblet	0.2480	0.09566 to 0.4004	Yes	****	<0.0001
Cxcl5	Lgr5-dim vs. EEC	0.2521	0.01878 to 0.4854	Yes	*	0.0197
Cxcl5	Lgr5-dim vs. Tuft	0.2423	0.03424 to 0.4504	Yes	**	0.0067
Cxcl5	Early TA vs. Abs Prog	-0.06634	-0.3548 to 0.2221	No	ns	>0.9999
Cxcl5	Early TA vs. Pre-DCC	0.1736	-0.06128 to 0.4084	No	ns	0.4468
Cxcl5	Early TA vs. Pre-PCC	0.2724	0.01932 to 0.5254	Yes	*	0.0207
Cxcl5	Early TA vs. Pro-DCC	0.1746	-0.04683 to 0.3961	No	ns	0.3287
Cxcl5	Early TA vs. Mature DCC	0.1830	-0.03321 to 0.3992	No	ns	0.2145
Cxcl5	Early TA vs. Mature PCC	0.2942	0.06923 to 0.5191	Yes	***	0.0008
Cxcl5	Early TA vs. P-I CC	0.03950	-0.3057 to 0.3847	No	ns	>0.9999
Cxcl5	Early TA vs. Pro-Senescent CC	0.2865	0.06033 to 0.5126	Yes	**	0.0015
Cxcl5	Early TA vs. Sec Prog	0.2419	0.01086 to 0.4729	Yes	*	0.0294
Cxcl5	Early TA vs. Paneth-like	0.2784	0.05128 to 0.5055	Yes	**	0.0028
Cxcl5	Early TA vs. Goblet	0.3167	0.09737 to 0.5360	Yes	****	<0.0001
Cxcl5	Early TA vs. EEC	0.3208	0.03912 to 0.6024	Yes	**	0.0094
Cxcl5	Early TA vs. Tuft	0.3110	0.04986 to 0.5720	Yes	**	0.0046
Cxcl5	Abs Prog vs. Pre-DCC	0.2399	0.01937 to 0.4605	Yes	*	0.0180
Cxcl5	Abs Prog vs. Pre-PCC	0.3387	0.09887 to 0.5785	Yes	***	0.0002
Cxcl5	Abs Prog vs. Pro-DCC	0.2410	0.03473 to 0.4472	Yes	**	0.0063
Cxcl5	Abs Prog vs. Mature DCC	0.2493	0.04875 to 0.4499	Yes	**	0.0022
Cxcl5	Abs Prog vs. Mature PCC	0.3605	0.1505 to 0.5705	Yes	****	<0.0001
Cxcl5	Abs Prog vs. P-I CC	0.1058	-0.2298 to 0.4415	No	ns	0.9995
Cxcl5	Abs Prog vs. Pro-Senescent CC	0.3528	0.1416 to 0.5640	Yes	****	<0.0001
Cxcl5	Abs Prog vs. Sec Prog	0.3082	0.09175 to 0.5247	Yes	***	0.0001
Cxcl5	Abs Prog vs. Paneth-like	0.3447	0.1324 to 0.5570	Yes	****	<0.0001

Cxcl5	Abs Prog vs. Goblet	0.3830	0.1791 to 0.5869	Yes	****	<0.0001
Cxcl5	Abs Prog vs. EEC	0.3871	0.1173 to 0.6569	Yes	***	0.0001
Cxcl5	Abs Prog vs. Tuft	0.3773	0.1290 to 0.6256	Yes	****	<0.0001
Cxcl5	Pre-DCC vs. Pre-PCC	0.09880	-0.07286 to 0.2705	No	ns	0.8404
Cxcl5	Pre-DCC vs. Pro-DCC	0.001059	-0.1193 to 0.1214	No	ns	>0.9999
Cxcl5	Pre-DCC vs. Mature DCC	0.009419	-0.1009 to 0.1198	No	ns	>0.9999
Cxcl5	Pre-DCC vs. Mature PCC	0.1206	-0.006015 to 0.2472	No	ns	0.0822
Cxcl5	Pre-DCC vs. P-I CC	-0.1341	-0.4249 to 0.1568	No	ns	0.9720
Cxcl5	Pre-DCC vs. Pro-Senescent CC	0.1129	-0.01582 to 0.2416	No	ns	0.1663
Cxcl5	Pre-DCC vs. Sec Prog	0.06833	-0.06883 to 0.2055	No	ns	0.9452
Cxcl5	Pre-DCC vs. Paneth-like	0.1048	-0.02560 to 0.2352	No	ns	0.2960
Cxcl5	Pre-DCC vs. Goblet	0.1431	0.02680 to 0.2594	Yes	**	0.0026
Cxcl5	Pre-DCC vs. EEC	0.1472	-0.06436 to 0.3587	No	ns	0.5591
Cxcl5	Pre-DCC vs. Tuft	0.1374	-0.04593 to 0.3207	No	ns	0.4208
Cxcl5	Pre-PCC vs. Pro-DCC	-0.09774	-0.2506 to 0.05510	No	ns	0.7030
Cxcl5	Pre-PCC vs. Mature DCC	-0.08938	-0.2345 to 0.05573	No	ns	0.7580
Cxcl5	Pre-PCC vs. Mature PCC	0.02182	-0.1360 to 0.1797	No	ns	>0.9999
Cxcl5	Pre-PCC vs. P-I CC	-0.2329	-0.5386 to 0.07287	No	ns	0.3910
Cxcl5	Pre-PCC vs. Pro-Senescent CC	0.01409	-0.1454 to 0.1736	No	ns	>0.9999
Cxcl5	Pre-PCC vs. Sec Prog	-0.03047	-0.1969 to 0.1360	No	ns	>0.9999
Cxcl5	Pre-PCC vs. Paneth-like	0.006014	-0.1549 to 0.1669	No	ns	>0.9999
Cxcl5	Pre-PCC vs. Goblet	0.04431	-0.1054 to 0.1940	No	ns	0.9998
Cxcl5	Pre-PCC vs. EEC	0.04839	-0.1832 to 0.2800	No	ns	>0.9999
Cxcl5	Pre-PCC vs. Tuft	0.03858	-0.1675 to 0.2447	No	ns	>0.9999
Cxcl5	Pro-DCC vs. Mature DCC	0.008360	-0.06954 to 0.08626	No	ns	>0.9999
Cxcl5	Pro-DCC vs. Mature PCC	0.1196	0.01991 to 0.2192	Yes	**	0.0041
Cxcl5	Pro-DCC vs. P-I CC	-0.1351	-0.4153 to 0.1450	No	ns	0.9583
Cxcl5	Pro-DCC vs. Pro-Senescent CC	0.1118	0.009556 to 0.2141	Yes	*	0.0168
Cxcl5	Pro-DCC vs. Sec Prog	0.06727	-0.04545 to 0.1800	No	ns	0.7993
Cxcl5	Pro-DCC vs. Paneth-like	0.1038	-0.0006594 to 0.2082	No	ns	0.0535
Cxcl5	Pro-DCC vs. Goblet	0.1420	0.05590 to 0.2282	Yes	****	<0.0001
Cxcl5	Pro-DCC vs. EEC	0.1461	-0.05045 to 0.3427	No	ns	0.4360
Cxcl5	Pro-DCC vs. Tuft	0.1363	-0.02949 to 0.3021	No	ns	0.2588
Cxcl5	Mature DCC vs. Mature PCC	0.1112	0.02387 to 0.1985	Yes	**	0.0014
Cxcl5	Mature DCC vs. P-I CC	-0.1435	-0.4195 to 0.1325	No	ns	0.9230
Cxcl5	Mature DCC vs. Pro-Senescent CC	0.1035	0.01316 to 0.1938	Yes	**	0.0086
Cxcl5	Mature DCC vs. Sec Prog	0.05891	-0.04309 to 0.1609	No	ns	0.8367
Cxcl5	Mature DCC vs. Paneth-like	0.09539	0.002665 to 0.1881	Yes	*	0.0363
Cxcl5	Mature DCC vs. Goblet	0.1337	0.06215 to 0.2052	Yes	****	<0.0001
Cxcl5	Mature DCC vs. EEC	0.1378	-0.05286 to 0.3284	No	ns	0.4888
Cxcl5	Mature DCC vs. Tuft	0.1280	-0.03076 to 0.2867	No	ns	0.2906
Cxcl5	Mature PCC vs. P-I CC	-0.2547	-0.5376 to 0.02824	No	ns	0.1356
Cxcl5	Mature PCC vs. Pro-Senescent CC	-0.007731	-0.1173 to 0.1019	No	ns	>0.9999
Cxcl5	Mature PCC vs. Sec Prog	-0.05228	-0.1717 to 0.06715	No	ns	0.9827
Cxcl5	Mature PCC vs. Paneth-like	-0.01580	-0.1274 to 0.09582	No	ns	>0.9999
Cxcl5	Mature PCC vs. Goblet	0.02249	-0.07226 to 0.1172	No	ns	>0.9999
Cxcl5	Mature PCC vs. EEC	0.02657	-0.1739 to 0.2271	No	ns	>0.9999
Cxcl5	Mature PCC vs. Tuft	0.01677	-0.1537 to 0.1872	No	ns	>0.9999
Cxcl5	P-I CC vs. Pro-Senescent CC	0.2469	-0.03690 to 0.5308	No	ns	0.1767
Cxcl5	P-I CC vs. Sec Prog	0.2024	-0.08539 to 0.4902	No	ns	0.5394
Cxcl5	P-I CC vs. Paneth-like	0.2389	-0.04575 to 0.5235	No	ns	0.2270
Cxcl5	P-I CC vs. Goblet	0.2772	-0.001280 to 0.5556	No	ns	0.0526
Cxcl5	P-I CC vs. EEC	0.2813	-0.04853 to 0.6110	No	ns	0.2037
Cxcl5	P-I CC vs. Tuft	0.2714	-0.04098 to 0.5839	No	ns	0.1784
Cxcl5	Pro-Senescent CC vs. Sec Prog	-0.04455	-0.1662 to 0.07708	No	ns	0.9972
Cxcl5	Pro-Senescent CC vs. Paneth-like	-0.008072	-0.1220 to 0.1059	No	ns	>0.9999
Cxcl5	Pro-Senescent CC vs. Goblet	0.03022	-0.06729 to 0.1277	No	ns	0.9996

Cxcl5	Pro-Senescent CC vs. EEC	0.03430	-0.1675 to 0.2361	No	ns	>0.9999
Cxcl5	Pro-Senescent CC vs. Tuft	0.02450	-0.1475 to 0.1965	No	ns	>0.9999
Cxcl5	Sec Prog vs. Paneth-like	0.03648	-0.08696 to 0.1599	No	ns	0.9998
Cxcl5	Sec Prog vs. Goblet	0.07477	-0.03366 to 0.1832	No	ns	0.5753
Cxcl5	Sec Prog vs. EEC	0.07885	-0.1285 to 0.2862	No	ns	0.9958
Cxcl5	Sec Prog vs. Tuft	0.06905	-0.1094 to 0.2475	No	ns	0.9950
Cxcl5	Paneth-like vs. Goblet	0.03829	-0.06146 to 0.1380	No	ns	0.9954
Cxcl5	Paneth-like vs. EEC	0.04237	-0.1605 to 0.2453	No	ns	>0.9999
Cxcl5	Paneth-like vs. Tuft	0.03257	-0.1407 to 0.2058	No	ns	>0.9999
Cxcl5	Goblet vs. EEC	0.004082	-0.1901 to 0.1982	No	ns	>0.9999
Cxcl5	Goblet vs. Tuft	-0.005722	-0.1686 to 0.1572	No	ns	>0.9999
Cxcl5	EEC vs. Tuft	-0.009804	-0.2502 to 0.2305	No	ns	>0.9999

Gene	Tukey's multiple comparisons test	Mean Diff.	95.00% CI of diff.	Below threshold?	Summary	Adjusted P Value
Lrg1	Lgr5-hi vs. Lgr5-dim	0.000	-0.02557 to 0.02557	No	ns	>0.9999
Lrg1	Lgr5-hi vs. Early TA	0.000	-0.03501 to 0.03501	No	ns	>0.9999
Lrg1	Lgr5-hi vs. Abs Prog	0.000	-0.03280 to 0.03280	No	ns	>0.9999
Lrg1	Lgr5-hi vs. Pre-DCC	-0.01299	-0.03376 to 0.007785	No	ns	0.7369
Lrg1	Lgr5-hi vs. Pre-PCC	0.000	-0.02521 to 0.02521	No	ns	>0.9999
Lrg1	Lgr5-hi vs. Pro-DCC	0.000	-0.01707 to 0.01707	No	ns	>0.9999
Lrg1	Lgr5-hi vs. Mature DCC	0.000	-0.01544 to 0.01544	No	ns	>0.9999
Lrg1	Lgr5-hi vs. Mature PCC	0.000	-0.01808 to 0.01808	No	ns	>0.9999
Lrg1	Lgr5-hi vs. P-I CC	0.000	-0.04361 to 0.04361	No	ns	>0.9999
Lrg1	Lgr5-hi vs. Pro-Senescent CC	0.000	-0.01842 to 0.01842	No	ns	>0.9999
Lrg1	Lgr5-hi vs. Sec Prog	-0.01792	-0.03769 to 0.001848	No	ns	0.1280
Lrg1	Lgr5-hi vs. Paneth-like	-0.03390	-0.05259 to -0.01521	Yes	****	<0.0001
Lrg1	Lgr5-hi vs. Goblet	0.000	-0.01641 to 0.01641	No	ns	>0.9999
Lrg1	Lgr5-hi vs. EEC	0.000	-0.03141 to 0.03141	No	ns	>0.9999
Lrg1	Lgr5-hi vs. Tuft	0.000	-0.02703 to 0.02703	No	ns	>0.9999
Lrg1	Lgr5-dim vs. Early TA	0.000	-0.03859 to 0.03859	No	ns	>0.9999
Lrg1	Lgr5-dim vs. Abs Prog	0.000	-0.03660 to 0.03660	No	ns	>0.9999
Lrg1	Lgr5-dim vs. Pre-DCC	-0.01299	-0.03936 to 0.01338	No	ns	0.9502
Lrg1	Lgr5-dim vs. Pre-PCC	0.000	-0.02999 to 0.02999	No	ns	>0.9999
Lrg1	Lgr5-dim vs. Pro-DCC	0.000	-0.02356 to 0.02356	No	ns	>0.9999
Lrg1	Lgr5-dim vs. Mature DCC	0.000	-0.02241 to 0.02241	No	ns	>0.9999
Lrg1	Lgr5-dim vs. Mature PCC	0.000	-0.02431 to 0.02431	No	ns	>0.9999
Lrg1	Lgr5-dim vs. P-I CC	0.000	-0.04654 to 0.04654	No	ns	>0.9999
Lrg1	Lgr5-dim vs. Pro-Senescent CC	0.000	-0.02456 to 0.02456	No	ns	>0.9999
Lrg1	Lgr5-dim vs. Sec Prog	-0.01792	-0.04351 to 0.007666	No	ns	0.5470
Lrg1	Lgr5-dim vs. Paneth-like	-0.03390	-0.05866 to -0.009135	Yes	***	0.0003
Lrg1	Lgr5-dim vs. Goblet	0.000	-0.02309 to 0.02309	No	ns	>0.9999
Lrg1	Lgr5-dim vs. EEC	0.000	-0.03536 to 0.03536	No	ns	>0.9999
Lrg1	Lgr5-dim vs. Tuft	0.000	-0.03153 to 0.03153	No	ns	>0.9999
Lrg1	Early TA vs. Abs Prog	0.000	-0.04372 to 0.04372	No	ns	>0.9999
Lrg1	Early TA vs. Pre-DCC	-0.01299	-0.04858 to 0.02261	No	ns	0.9973
Lrg1	Early TA vs. Pre-PCC	0.000	-0.03835 to 0.03835	No	ns	>0.9999
Lrg1	Early TA vs. Pro-DCC	0.000	-0.03357 to 0.03357	No	ns	>0.9999
Lrg1	Early TA vs. Mature DCC	0.000	-0.03277 to 0.03277	No	ns	>0.9999
Lrg1	Early TA vs. Mature PCC	0.000	-0.03409 to 0.03409	No	ns	>0.9999
Lrg1	Early TA vs. P-I CC	0.000	-0.05232 to 0.05232	No	ns	>0.9999
Lrg1	Early TA vs. Pro-Senescent CC	0.000	-0.03427 to 0.03427	No	ns	>0.9999
Lrg1	Early TA vs. Sec Prog	-0.01792	-0.05294 to 0.01710	No	ns	0.9319
Lrg1	Early TA vs. Paneth-like	-0.03390	-0.06832 to 0.0005224	No	ns	0.0589
Lrg1	Early TA vs. Goblet	0.000	-0.03324 to 0.03324	No	ns	>0.9999
Lrg1	Early TA vs. EEC	0.000	-0.04269 to 0.04269	No	ns	>0.9999
Lrg1	Early TA vs. Tuft	0.000	-0.03957 to 0.03957	No	ns	>0.9999
Lrg1	Abs Prog vs. Pre-DCC	-0.01299	-0.04641 to 0.02044	No	ns	0.9948
Lrg1	Abs Prog vs. Pre-PCC	0.000	-0.03635 to 0.03635	No	ns	>0.9999
Lrg1	Abs Prog vs. Pro-DCC	0.000	-0.03126 to 0.03126	No	ns	>0.9999
Lrg1	Abs Prog vs. Mature DCC	0.000	-0.03040 to 0.03040	No	ns	>0.9999
Lrg1	Abs Prog vs. Mature PCC	0.000	-0.03183 to 0.03183	No	ns	>0.9999
Lrg1	Abs Prog vs. P-I CC	0.000	-0.05087 to 0.05087	No	ns	>0.9999

Lrg1	Abs Prog vs. Pro-Senescent CC	0.000	-0.03202 to 0.03202	No	ns	>0.9999
Lrg1	Abs Prog vs. Sec Prog	-0.01792	-0.05073 to 0.01489	No	ns	0.8887
Lrg1	Abs Prog vs. Paneth-like	-0.03390	-0.06607 to -0.001724	Yes	*	0.0272
Lrg1	Abs Prog vs. Goblet	0.000	-0.03091 to 0.03091	No	ns	>0.9999
Lrg1	Abs Prog vs. EEC	0.000	-0.04090 to 0.04090	No	ns	>0.9999
Lrg1	Abs Prog vs. Tuft	0.000	-0.03763 to 0.03763	No	ns	>0.9999
Lrg1	Pre-DCC vs. Pre-PCC	0.01299	-0.01303 to 0.03900	No	ns	0.9443
Lrg1	Pre-DCC vs. Pro-DCC	0.01299	-0.005250 to 0.03122	No	ns	0.5163
Lrg1	Pre-DCC vs. Mature DCC	0.01299	-0.003737 to 0.02971	No	ns	0.3557
Lrg1	Pre-DCC vs. Mature PCC	0.01299	-0.006206 to 0.03218	No	ns	0.6091
Lrg1	Pre-DCC vs. P-I CC	0.01299	-0.03109 to 0.05707	No	ns	0.9998
Lrg1	Pre-DCC vs. Pro-Senescent CC	0.01299	-0.006520 to 0.03249	No	ns	0.6373
Lrg1	Pre-DCC vs. Sec Prog	-0.004934	-0.02572 to 0.01586	No	ns	>0.9999
Lrg1	Pre-DCC vs. Paneth-like	-0.02091	-0.04068 to -0.001145	Yes	*	0.0259
Lrg1	Pre-DCC vs. Goblet	0.01299	-0.004641 to 0.03061	No	ns	0.4528
Lrg1	Pre-DCC vs. EEC	0.01299	-0.01908 to 0.04505	No	ns	0.9919
Lrg1	Pre-DCC vs. Tuft	0.01299	-0.01480 to 0.04077	No	ns	0.9682
Lrg1	Pre-PCC vs. Pro-DCC	0.000	-0.02316 to 0.02316	No	ns	>0.9999
Lrg1	Pre-PCC vs. Mature DCC	0.000	-0.02199 to 0.02199	No	ns	>0.9999
Lrg1	Pre-PCC vs. Mature PCC	0.000	-0.02392 to 0.02392	No	ns	>0.9999
Lrg1	Pre-PCC vs. P-I CC	0.000	-0.04634 to 0.04634	No	ns	>0.9999
Lrg1	Pre-PCC vs. Pro-Senescent CC	0.000	-0.02418 to 0.02418	No	ns	>0.9999
Lrg1	Pre-PCC vs. Sec Prog	-0.01792	-0.04314 to 0.007302	No	ns	0.5205
Lrg1	Pre-PCC vs. Paneth-like	-0.03390	-0.05829 to -0.009511	Yes	***	0.0002
Lrg1	Pre-PCC vs. Goblet	0.000	-0.02269 to 0.02269	No	ns	>0.9999
Lrg1	Pre-PCC vs. EEC	0.000	-0.03510 to 0.03510	No	ns	>0.9999
Lrg1	Pre-PCC vs. Tuft	0.000	-0.03124 to 0.03124	No	ns	>0.9999
Lrg1	Pro-DCC vs. Mature DCC	0.000	-0.01181 to 0.01181	No	ns	>0.9999
Lrg1	Pro-DCC vs. Mature PCC	0.000	-0.01510 to 0.01510	No	ns	>0.9999
Lrg1	Pro-DCC vs. P-I CC	0.000	-0.04246 to 0.04246	No	ns	>0.9999
Lrg1	Pro-DCC vs. Pro-Senescent CC	0.000	-0.01550 to 0.01550	No	ns	>0.9999
Lrg1	Pro-DCC vs. Sec Prog	-0.01792	-0.03501 to -0.0008357	Yes	*	0.0287
Lrg1	Pro-DCC vs. Paneth-like	-0.03390	-0.04972 to -0.01807	Yes	****	<0.0001
Lrg1	Pro-DCC vs. Goblet	0.000	-0.01306 to 0.01306	No	ns	>0.9999
Lrg1	Pro-DCC vs. EEC	0.000	-0.02979 to 0.02979	No	ns	>0.9999
Lrg1	Pro-DCC vs. Tuft	0.000	-0.02513 to 0.02513	No	ns	>0.9999
Lrg1	Mature DCC vs. Mature PCC	0.000	-0.01324 to 0.01324	No	ns	>0.9999
Lrg1	Mature DCC vs. P-I CC	0.000	-0.04183 to 0.04183	No	ns	>0.9999
Lrg1	Mature DCC vs. Pro-Senescent CC	0.000	-0.01369 to 0.01369	No	ns	>0.9999
Lrg1	Mature DCC vs. Sec Prog	-0.01792	-0.03338 to -0.002461	Yes	**	0.0072
Lrg1	Mature DCC vs. Paneth-like	-0.03390	-0.04795 to -0.01984	Yes	****	<0.0001
Lrg1	Mature DCC vs. Goblet	0.000	-0.01084 to 0.01084	No	ns	>0.9999
Lrg1	Mature DCC vs. EEC	0.000	-0.02889 to 0.02889	No	ns	>0.9999
Lrg1	Mature DCC vs. Tuft	0.000	-0.02406 to 0.02406	No	ns	>0.9999
Lrg1	Mature PCC vs. P-I CC	0.000	-0.04288 to 0.04288	No	ns	>0.9999
Lrg1	Mature PCC vs. Pro-Senescent CC	0.000	-0.01661 to 0.01661	No	ns	>0.9999
Lrg1	Mature PCC vs. Sec Prog	-0.01792	-0.03602 to 0.0001808	No	ns	0.0557
Lrg1	Mature PCC vs. Paneth-like	-0.03390	-0.05082 to -0.01698	Yes	****	<0.0001
Lrg1	Mature PCC vs. Goblet	0.000	-0.01436 to 0.01436	No	ns	>0.9999
Lrg1	Mature PCC vs. EEC	0.000	-0.03039 to 0.03039	No	ns	>0.9999
Lrg1	Mature PCC vs. Tuft	0.000	-0.02583 to 0.02583	No	ns	>0.9999
Lrg1	P-I CC vs. Pro-Senescent CC	0.000	-0.04302 to 0.04302	No	ns	>0.9999
Lrg1	P-I CC vs. Sec Prog	-0.01792	-0.06154 to 0.02570	No	ns	0.9907
Lrg1	P-I CC vs. Paneth-like	-0.03390	-0.07704 to 0.009242	No	ns	0.3348
Lrg1	P-I CC vs. Goblet	0.000	-0.04220 to 0.04220	No	ns	>0.9999
Lrg1	P-I CC vs. EEC	0.000	-0.04998 to 0.04998	No	ns	>0.9999
Lrg1	P-I CC vs. Tuft	0.000	-0.04735 to 0.04735	No	ns	>0.9999
Lrg1	Pro-Senescent CC vs. Sec Prog	-0.01792	-0.03636 to 0.0005143	No	ns	0.0673

Lrg1	Pro-Senescent CC vs. Paneth-like	-0.03390	-0.05117 to -0.01662	Yes	****	<0.0001
Lrg1	Pro-Senescent CC vs. Goblet	0.000	-0.01478 to 0.01478	No	ns	>0.9999
Lrg1	Pro-Senescent CC vs. EEC	0.000	-0.03059 to 0.03059	No	ns	>0.9999
Lrg1	Pro-Senescent CC vs. Tuft	0.000	-0.02607 to 0.02607	No	ns	>0.9999
Lrg1	Sec Prog vs. Paneth-like	-0.01598	-0.03469 to 0.002732	No	ns	0.2018
Lrg1	Sec Prog vs. Goblet	0.01792	0.001487 to 0.03435	Yes	*	0.0174
Lrg1	Sec Prog vs. EEC	0.01792	-0.01350 to 0.04934	No	ns	0.8497
Lrg1	Sec Prog vs. Tuft	0.01792	-0.009120 to 0.04496	No	ns	0.6450
Lrg1	Paneth-like vs. Goblet	0.03390	0.01878 to 0.04902	Yes	****	<0.0001
Lrg1	Paneth-like vs. EEC	0.03390	0.003144 to 0.06465	Yes	*	0.0150
Lrg1	Paneth-like vs. Tuft	0.03390	0.007635 to 0.06016	Yes	**	0.0011
Lrg1	Goblet vs. EEC	0.000	-0.02943 to 0.02943	No	ns	>0.9999
Lrg1	Goblet vs. Tuft	0.000	-0.02469 to 0.02469	No	ns	>0.9999
Lrg1	EEC vs. Tuft	0.000	-0.03643 to 0.03643	No	ns	>0.9999

Gene	Tukey's multiple comparisons test	Mean Diff.	95.00% CI of diff.	Below threshold?	Summary	Adjusted P Value
Fut2	Lgr5-hi vs. Lgr5-dim	-0.1735	-0.4863 to 0.1392	No	ns	0.8754
Fut2	Lgr5-hi vs. Early TA	-0.04272	-0.4708 to 0.3854	No	ns	>0.9999
Fut2	Lgr5-hi vs. Abs Prog	-0.3592	-0.7603 to 0.04190	No	ns	0.1414
Fut2	Lgr5-hi vs. Pre-DCC	-0.5232	-0.7772 to -0.2691	Yes	****	<0.0001
Fut2	Lgr5-hi vs. Pre-PCC	-1.198	-1.506 to -0.8897	Yes	****	<0.0001
Fut2	Lgr5-hi vs. Pro-DCC	-0.003924	-0.2126 to 0.2048	No	ns	>0.9999
Fut2	Lgr5-hi vs. Mature DCC	0.09316	-0.09561 to 0.2819	No	ns	0.9493
Fut2	Lgr5-hi vs. Mature PCC	-0.1602	-0.3813 to 0.06091	No	ns	0.4840
Fut2	Lgr5-hi vs. P-I CC	0.1589	-0.3743 to 0.6922	No	ns	0.9997
Fut2	Lgr5-hi vs. Pro-Senescent CC	0.1898	-0.03544 to 0.4150	No	ns	0.2210
Fut2	Lgr5-hi vs. Sec Prog	-1.467	-1.708 to -1.225	Yes	****	<0.0001
Fut2	Lgr5-hi vs. Paneth-like	-1.756	-1.985 to -1.527	Yes	****	<0.0001
Fut2	Lgr5-hi vs. Goblet	0.1085	-0.09219 to 0.3092	No	ns	0.8966
Fut2	Lgr5-hi vs. EEC	-0.2858	-0.6699 to 0.09828	No	ns	0.4341
Fut2	Lgr5-hi vs. Tuft	-0.01387	-0.3444 to 0.3166	No	ns	>0.9999
Fut2	Lgr5-dim vs. Early TA	0.1308	-0.3411 to 0.6027	No	ns	0.9999
Fut2	Lgr5-dim vs. Abs Prog	-0.1857	-0.6333 to 0.2619	No	ns	0.9897
Fut2	Lgr5-dim vs. Pre-DCC	-0.3496	-0.6721 to -0.02717	Yes	*	0.0188
Fut2	Lgr5-dim vs. Pre-PCC	-1.024	-1.391 to -0.6577	Yes	****	<0.0001
Fut2	Lgr5-dim vs. Pro-DCC	0.1696	-0.1185 to 0.4577	No	ns	0.8155
Fut2	Lgr5-dim vs. Mature DCC	0.2667	-0.007344 to 0.5407	No	ns	0.0665
Fut2	Lgr5-dim vs. Mature PCC	0.01332	-0.2839 to 0.3106	No	ns	>0.9999
Fut2	Lgr5-dim vs. P-I CC	0.3325	-0.2366 to 0.9015	No	ns	0.8242
Fut2	Lgr5-dim vs. Pro-Senescent CC	0.3633	0.06300 to 0.6636	Yes	**	0.0036
Fut2	Lgr5-dim vs. Sec Prog	-1.293	-1.606 to -0.9803	Yes	****	<0.0001
Fut2	Lgr5-dim vs. Paneth-like	-1.582	-1.885 to -1.280	Yes	****	<0.0001
Fut2	Lgr5-dim vs. Goblet	0.2820	-0.0003517 to 0.5644	No	ns	0.0507
Fut2	Lgr5-dim vs. EEC	-0.1123	-0.5447 to 0.3201	No	ns	>0.9999
Fut2	Lgr5-dim vs. Tuft	0.1597	-0.2259 to 0.5453	No	ns	0.9899
Fut2	Early TA vs. Abs Prog	-0.3165	-0.8511 to 0.2181	No	ns	0.8089
Fut2	Early TA vs. Pre-DCC	-0.4804	-0.9157 to -0.04518	Yes	*	0.0147
Fut2	Early TA vs. Pre-PCC	-1.155	-1.624 to -0.6862	Yes	****	<0.0001
Fut2	Early TA vs. Pro-DCC	0.03880	-0.3717 to 0.4493	No	ns	>0.9999
Fut2	Early TA vs. Mature DCC	0.1359	-0.2648 to 0.5366	No	ns	0.9988
Fut2	Early TA vs. Mature PCC	-0.1175	-0.5344 to 0.2994	No	ns	0.9999
Fut2	Early TA vs. P-I CC	0.2017	-0.4381 to 0.8414	No	ns	0.9995
Fut2	Early TA vs. Pro-Senescent CC	0.2325	-0.1866 to 0.6516	No	ns	0.8756
Fut2	Early TA vs. Sec Prog	-1.424	-1.852 to -0.9958	Yes	****	<0.0001
Fut2	Early TA vs. Paneth-like	-1.713	-2.134 to -1.292	Yes	****	<0.0001
Fut2	Early TA vs. Goblet	0.1512	-0.2552 to 0.5577	No	ns	0.9967
Fut2	Early TA vs. EEC	-0.2431	-0.7651 to 0.2789	No	ns	0.9693
Fut2	Early TA vs. Tuft	0.02886	-0.4550 to 0.5128	No	ns	>0.9999
Fut2	Abs Prog vs. Pre-DCC	-0.1639	-0.5727 to 0.2448	No	ns	0.9927
Fut2	Abs Prog vs. Pre-PCC	-0.8387	-1.283 to -0.3942	Yes	****	<0.0001

Fut2	Abs Prog vs. Pro-DCC	0.3553	-0.02694 to 0.7375	No	ns	0.1031
Fut2	Abs Prog vs. Mature DCC	0.4524	0.08064 to 0.8241	Yes	**	0.0032
Fut2	Abs Prog vs. Mature PCC	0.1990	-0.1902 to 0.5882	No	ns	0.9324
Fut2	Abs Prog vs. P-I CC	0.5181	-0.1039 to 1.140	No	ns	0.2383
Fut2	Abs Prog vs. Pro-Senescent CC	0.5490	0.1575 to 0.9405	Yes	***	0.0002
Fut2	Abs Prog vs. Sec Prog	-1.108	-1.509 to -0.7063	Yes	****	<0.0001
Fut2	Abs Prog vs. Paneth-like	-1.397	-1.790 to -1.003	Yes	****	<0.0001
Fut2	Abs Prog vs. Goblet	0.4677	0.08978 to 0.8457	Yes	**	0.0024
Fut2	Abs Prog vs. EEC	0.07340	-0.4267 to 0.5735	No	ns	>0.9999
Fut2	Abs Prog vs. Tuft	0.3454	-0.1149 to 0.8056	No	ns	0.4184
Fut2	Pre-DCC vs. Pre-PCC	-0.6748	-0.9929 to -0.3566	Yes	****	<0.0001
Fut2	Pre-DCC vs. Pro-DCC	0.5192	0.2962 to 0.7422	Yes	****	<0.0001
Fut2	Pre-DCC vs. Mature DCC	0.6163	0.4118 to 0.8208	Yes	****	<0.0001
Fut2	Pre-DCC vs. Mature PCC	0.3629	0.1283 to 0.5976	Yes	****	<0.0001
Fut2	Pre-DCC vs. P-I CC	0.6821	0.1430 to 1.221	Yes	**	0.0016
Fut2	Pre-DCC vs. Pro-Senescent CC	0.7129	0.4744 to 0.9515	Yes	****	<0.0001
Fut2	Pre-DCC vs. Sec Prog	-0.9436	-1.198 to -0.6894	Yes	****	<0.0001
Fut2	Pre-DCC vs. Paneth-like	-1.233	-1.475 to -0.9911	Yes	****	<0.0001
Fut2	Pre-DCC vs. Goblet	0.6317	0.4161 to 0.8472	Yes	****	<0.0001
Fut2	Pre-DCC vs. EEC	0.2373	-0.1547 to 0.6294	No	ns	0.7812
Fut2	Pre-DCC vs. Tuft	0.5093	0.1696 to 0.8490	Yes	****	<0.0001
Fut2	Pre-PCC vs. Pro-DCC	1.194	0.9107 to 1.477	Yes	****	<0.0001
Fut2	Pre-PCC vs. Mature DCC	1.291	1.022 to 1.560	Yes	****	<0.0001
Fut2	Pre-PCC vs. Mature PCC	1.038	0.7452 to 1.330	Yes	****	<0.0001
Fut2	Pre-PCC vs. P-I CC	1.357	0.7902 to 1.923	Yes	****	<0.0001
Fut2	Pre-PCC vs. Pro-Senescent CC	1.388	1.092 to 1.683	Yes	****	<0.0001
Fut2	Pre-PCC vs. Sec Prog	-0.2688	-0.5773 to 0.03962	No	ns	0.1744
Fut2	Pre-PCC vs. Paneth-like	-0.5580	-0.8563 to -0.2598	Yes	****	<0.0001
Fut2	Pre-PCC vs. Goblet	1.306	1.029 to 1.584	Yes	****	<0.0001
Fut2	Pre-PCC vs. EEC	0.9121	0.4829 to 1.341	Yes	****	<0.0001
Fut2	Pre-PCC vs. Tuft	1.184	0.8021 to 1.566	Yes	****	<0.0001
Fut2	Pro-DCC vs. Mature DCC	0.09709	-0.04729 to 0.2415	No	ns	0.6200
Fut2	Pro-DCC vs. Mature PCC	-0.1563	-0.3410 to 0.02839	No	ns	0.2147
Fut2	Pro-DCC vs. P-I CC	0.1629	-0.3564 to 0.6821	No	ns	0.9995
Fut2	Pro-DCC vs. Pro-Senescent CC	0.1937	0.004146 to 0.3832	Yes	*	0.0392
Fut2	Pro-DCC vs. Sec Prog	-1.463	-1.672 to -1.254	Yes	****	<0.0001
Fut2	Pro-DCC vs. Paneth-like	-1.752	-1.946 to -1.559	Yes	****	<0.0001
Fut2	Pro-DCC vs. Goblet	0.1124	-0.04723 to 0.2721	No	ns	0.5371
Fut2	Pro-DCC vs. EEC	-0.2819	-0.6462 to 0.08244	No	ns	0.3623
Fut2	Pro-DCC vs. Tuft	-0.009942	-0.3173 to 0.2974	No	ns	>0.9999
Fut2	Mature DCC vs. Mature PCC	-0.2534	-0.4152 to -0.09153	Yes	****	<0.0001
Fut2	Mature DCC vs. P-I CC	0.06577	-0.4458 to 0.5773	No	ns	>0.9999
Fut2	Mature DCC vs. Pro-Senescent CC	0.09660	-0.07077 to 0.2640	No	ns	0.8374
Fut2	Mature DCC vs. Sec Prog	-1.560	-1.749 to -1.371	Yes	****	<0.0001
Fut2	Mature DCC vs. Paneth-like	-1.849	-2.021 to -1.677	Yes	****	<0.0001
Fut2	Mature DCC vs. Goblet	0.01534	-0.1172 to 0.1479	No	ns	>0.9999
Fut2	Mature DCC vs. EEC	-0.3790	-0.7323 to -0.02567	Yes	*	0.0217
Fut2	Mature DCC vs. Tuft	-0.1070	-0.4012 to 0.1871	No	ns	0.9974
Fut2	Mature PCC vs. P-I CC	0.3191	-0.2052 to 0.8435	No	ns	0.7742
Fut2	Mature PCC vs. Pro-Senescent CC	0.3500	0.1468 to 0.5531	Yes	****	<0.0001
Fut2	Mature PCC vs. Sec Prog	-1.307	-1.528 to -1.085	Yes	****	<0.0001
Fut2	Mature PCC vs. Paneth-like	-1.596	-1.803 to -1.389	Yes	****	<0.0001
Fut2	Mature PCC vs. Goblet	0.2687	0.09311 to 0.4443	Yes	****	<0.0001
Fut2	Mature PCC vs. EEC	-0.1256	-0.4972 to 0.2460	No	ns	0.9989
Fut2	Mature PCC vs. Tuft	0.1463	-0.1696 to 0.4623	No	ns	0.9707
Fut2	P-I CC vs. Pro-Senescent CC	0.03084	-0.4952 to 0.5569	No	ns	>0.9999
Fut2	P-I CC vs. Sec Prog	-1.626	-2.159 to -1.092	Yes	****	<0.0001
Fut2	P-I CC vs. Paneth-like	-1.915	-2.442 to -1.387	Yes	****	<0.0001
Fut2	P-I CC vs. Goblet	-0.05043	-0.5665 to 0.4656	No	ns	>0.9999

Fut2	P-I CC vs. EEC	-0.4447	-1.056 to 0.1665	No	ns	0.4760
Fut2	P-I CC vs. Tuft	-0.1728	-0.7518 to 0.4062	No	ns	0.9997
Fut2	Pro-Senescent CC vs. Sec Prog	-1.657	-1.882 to -1.431	Yes	****	<0.0001
Fut2	Pro-Senescent CC vs. Paneth-like	-1.946	-2.157 to -1.735	Yes	****	<0.0001
Fut2	Pro-Senescent CC vs. Goblet	-0.08126	-0.2620 to 0.09946	No	ns	0.9777
Fut2	Pro-Senescent CC vs. EEC	-0.4756	-0.8496 to -0.1015	Yes	**	0.0014
Fut2	Pro-Senescent CC vs. Tuft	-0.2036	-0.5224 to 0.1151	No	ns	0.7047
Fut2	Sec Prog vs. Paneth-like	-0.2892	-0.5180 to -0.06044	Yes	**	0.0016
Fut2	Sec Prog vs. Goblet	1.575	1.374 to 1.776	Yes	****	<0.0001
Fut2	Sec Prog vs. EEC	1.181	0.7967 to 1.565	Yes	****	<0.0001
Fut2	Sec Prog vs. Tuft	1.453	1.122 to 1.784	Yes	****	<0.0001
Fut2	Paneth-like vs. Goblet	1.864	1.680 to 2.049	Yes	****	<0.0001
Fut2	Paneth-like vs. EEC	1.470	1.094 to 1.846	Yes	****	<0.0001
Fut2	Paneth-like vs. Tuft	1.742	1.421 to 2.063	Yes	****	<0.0001
Fut2	Goblet vs. EEC	-0.3943	-0.7541 to -0.03450	Yes	*	0.0163
Fut2	Goblet vs. Tuft	-0.1224	-0.4243 to 0.1796	No	ns	0.9919
Fut2	EEC vs. Tuft	0.2720	-0.1735 to 0.7174	No	ns	0.7701

Supplementary Table 6. One-way ANOVA of IL-22–inducible genes from d9 *C.r*-infected control *Il22*^{hCD4} mice shown in Figure ED6d.

Gene	Tukey's multiple comparisons test	Mean Diff.	95.00% CI of diff.	Below threshold?	Summary	Adjusted P Value
S100a8	Lgr5-hi vs. Lgr5-dim	-0.2698	-3.272 to 2.733	No	ns	>0.9999
S100a8	Lgr5-hi vs. Early TA	-0.6201	-4.385 to 3.145	No	ns	>0.9999
S100a8	Lgr5-hi vs. Abs Prog	-1.198	-4.339 to 1.943	No	ns	0.9957
S100a8	Lgr5-hi vs. Pre-DCC	-1.377	-4.314 to 1.559	No	ns	0.9673
S100a8	Lgr5-hi vs. Pre-PCC	-2.575	-6.588 to 1.437	No	ns	0.6975
S100a8	Lgr5-hi vs. Pro-DCC	-18.02	-21.65 to -14.39	Yes	****	<0.0001
S100a8	Lgr5-hi vs. Mature DCC	-3.080	-6.040 to -0.1188	Yes	*	0.0318
S100a8	Lgr5-hi vs. Mature PCC	-1.084	-4.147 to 1.979	No	ns	0.9981
S100a8	Lgr5-hi vs. P-I CC	-10.98	-14.42 to -7.544	Yes	****	<0.0001
S100a8	Lgr5-hi vs. Pro-Senescent CC	-0.7220	-3.479 to 2.035	No	ns	>0.9999
S100a8	Lgr5-hi vs. Sec Prog	-1.817	-5.277 to 1.643	No	ns	0.9168
S100a8	Lgr5-hi vs. Paneth-like	-0.6355	-4.157 to 2.886	No	ns	>0.9999
S100a8	Lgr5-hi vs. Goblet	-0.9085	-5.017 to 3.200	No	ns	>0.9999
S100a8	Lgr5-hi vs. Endocrine	-6.584	-10.83 to -2.341	Yes	****	<0.0001
S100a8	Lgr5-hi vs. Tuft	-1.820	-8.998 to 5.358	No	ns	>0.9999
S100a8	Lgr5-dim vs. Early TA	-0.3504	-3.890 to 3.190	No	ns	>0.9999
S100a8	Lgr5-dim vs. Abs Prog	-0.9282	-3.796 to 1.940	No	ns	0.9993
S100a8	Lgr5-dim vs. Pre-DCC	-1.108	-3.750 to 1.534	No	ns	0.9886
S100a8	Lgr5-dim vs. Pre-PCC	-2.306	-6.108 to 1.497	No	ns	0.7792
S100a8	Lgr5-dim vs. Pro-DCC	-17.75	-21.15 to -14.36	Yes	****	<0.0001
S100a8	Lgr5-dim vs. Mature DCC	-2.810	-5.478 to -0.1411	Yes	*	0.0274
S100a8	Lgr5-dim vs. Mature PCC	-0.8138	-3.595 to 1.968	No	ns	0.9998
S100a8	Lgr5-dim vs. P-I CC	-10.71	-13.90 to -7.523	Yes	****	<0.0001
S100a8	Lgr5-dim vs. Pro-Senescent CC	-0.4522	-2.893 to 1.989	No	ns	>0.9999
S100a8	Lgr5-dim vs. Sec Prog	-1.547	-4.761 to 1.666	No	ns	0.9589
S100a8	Lgr5-dim vs. Paneth-like	-0.3658	-3.645 to 2.914	No	ns	>0.9999
S100a8	Lgr5-dim vs. Goblet	-0.6388	-4.542 to 3.264	No	ns	>0.9999
S100a8	Lgr5-dim vs. Endocrine	-6.315	-10.36 to -2.270	Yes	****	<0.0001
S100a8	Lgr5-dim vs. Tuft	-1.550	-8.612 to 5.513	No	ns	>0.9999
S100a8	Early TA vs. Abs Prog	-0.5779	-4.236 to 3.081	No	ns	>0.9999
S100a8	Early TA vs. Pre-DCC	-0.7572	-4.241 to 2.727	No	ns	>0.9999
S100a8	Early TA vs. Pre-PCC	-1.955	-6.384 to 2.474	No	ns	0.9812
S100a8	Early TA vs. Pro-DCC	-17.40	-21.49 to -13.32	Yes	****	<0.0001
S100a8	Early TA vs. Mature DCC	-2.459	-5.964 to 1.045	No	ns	0.5435
S100a8	Early TA vs. Mature PCC	-0.4635	-4.055 to 3.128	No	ns	>0.9999
S100a8	Early TA vs. P-I CC	-10.36	-14.27 to -6.446	Yes	****	<0.0001
S100a8	Early TA vs. Pro-Senescent CC	-0.1019	-3.436 to 3.232	No	ns	>0.9999
S100a8	Early TA vs. Sec Prog	-1.197	-5.132 to 2.738	No	ns	0.9997
S100a8	Early TA vs. Paneth-like	-0.01540	-4.005 to 3.974	No	ns	>0.9999
S100a8	Early TA vs. Goblet	-0.2884	-4.804 to 4.228	No	ns	>0.9999
S100a8	Early TA vs. Endocrine	-5.964	-10.60 to -1.325	Yes	**	0.0012
S100a8	Early TA vs. Tuft	-1.200	-8.618 to 6.219	No	ns	>0.9999
S100a8	Abs Prog vs. Pre-DCC	-0.1793	-2.978 to 2.619	No	ns	>0.9999
S100a8	Abs Prog vs. Pre-PCC	-1.377	-5.290 to 2.535	No	ns	0.9982
S100a8	Abs Prog vs. Pro-DCC	-16.82	-20.34 to -13.30	Yes	****	<0.0001
S100a8	Abs Prog vs. Mature DCC	-1.882	-4.705 to 0.9424	No	ns	0.6361
S100a8	Abs Prog vs. Mature PCC	0.1144	-2.817 to 3.045	No	ns	>0.9999
S100a8	Abs Prog vs. P-I CC	-9.782	-13.10 to -6.463	Yes	****	<0.0001
S100a8	Abs Prog vs. Pro-Senescent CC	0.4760	-2.134 to 3.086	No	ns	>0.9999
S100a8	Abs Prog vs. Sec Prog	-0.6190	-3.963 to 2.725	No	ns	>0.9999
S100a8	Abs Prog vs. Paneth-like	0.5625	-2.845 to 3.970	No	ns	>0.9999
S100a8	Abs Prog vs. Goblet	0.2895	-3.721 to 4.300	No	ns	>0.9999
S100a8	Abs Prog vs. Endocrine	-5.386	-9.535 to -1.237	Yes	***	0.0010
S100a8	Abs Prog vs. Tuft	-0.6216	-7.744 to 6.501	No	ns	>0.9999
S100a8	Pre-DCC vs. Pre-PCC	-1.198	-4.948 to 2.552	No	ns	0.9994
S100a8	Pre-DCC vs. Pro-DCC	-16.64	-19.98 to -13.31	Yes	****	<0.0001
S100a8	Pre-DCC vs. Mature DCC	-1.702	-4.296 to 0.8921	No	ns	0.6619

S100a8	Pre-DCC vs. Mature PCC	0.2938	-2.417 to 3.004	No	ns	>0.9999
S100a8	Pre-DCC vs. P-I CC	-9.603	-12.73 to -6.477	Yes	****	<0.0001
S100a8	Pre-DCC vs. Pro-Senescent CC	0.6553	-1.704 to 3.015	No	ns	0.9999
S100a8	Pre-DCC vs. Sec Prog	-0.4397	-3.592 to 2.712	No	ns	>0.9999
S100a8	Pre-DCC vs. Paneth-like	0.7418	-2.478 to 3.961	No	ns	>0.9999
S100a8	Pre-DCC vs. Goblet	0.4688	-3.384 to 4.321	No	ns	>0.9999
S100a8	Pre-DCC vs. Endocrine	-5.207	-9.203 to -1.211	Yes	***	0.0009
S100a8	Pre-DCC vs. Tuft	-0.4423	-7.477 to 6.592	No	ns	>0.9999
S100a8	Pre-PCC vs. Pro-DCC	-15.45	-19.76 to -11.13	Yes	****	<0.0001
S100a8	Pre-PCC vs. Mature DCC	-0.5042	-4.273 to 3.265	No	ns	>0.9999
S100a8	Pre-PCC vs. Mature PCC	1.492	-2.358 to 5.342	No	ns	0.9949
S100a8	Pre-PCC vs. P-I CC	-8.405	-12.56 to -4.252	Yes	****	<0.0001
S100a8	Pre-PCC vs. Pro-Senescent CC	1.853	-1.758 to 5.465	No	ns	0.9305
S100a8	Pre-PCC vs. Sec Prog	0.7583	-3.414 to 4.931	No	ns	>0.9999
S100a8	Pre-PCC vs. Paneth-like	1.940	-2.284 to 6.164	No	ns	0.9729
S100a8	Pre-PCC vs. Goblet	1.667	-3.057 to 6.391	No	ns	0.9982
S100a8	Pre-PCC vs. Endocrine	-4.009	-8.851 to 0.8329	No	ns	0.2477
S100a8	Pre-PCC vs. Tuft	0.7557	-6.792 to 8.303	No	ns	>0.9999
S100a8	Pro-DCC vs. Mature DCC	14.94	11.58 to 18.30	Yes	****	<0.0001
S100a8	Pro-DCC vs. Mature PCC	16.94	13.49 to 20.39	Yes	****	<0.0001
S100a8	Pro-DCC vs. P-I CC	7.041	3.257 to 10.83	Yes	****	<0.0001
S100a8	Pro-DCC vs. Pro-Senescent CC	17.30	14.12 to 20.48	Yes	****	<0.0001
S100a8	Pro-DCC vs. Sec Prog	16.20	12.40 to 20.01	Yes	****	<0.0001
S100a8	Pro-DCC vs. Paneth-like	17.39	13.52 to 21.25	Yes	****	<0.0001
S100a8	Pro-DCC vs. Goblet	17.11	12.71 to 21.52	Yes	****	<0.0001
S100a8	Pro-DCC vs. Endocrine	11.44	6.907 to 15.97	Yes	****	<0.0001
S100a8	Pro-DCC vs. Tuft	16.20	8.851 to 23.55	Yes	****	<0.0001
S100a8	Mature DCC vs. Mature PCC	1.996	-0.7405 to 4.732	No	ns	0.4716
S100a8	Mature DCC vs. P-I CC	-7.900	-11.05 to -4.752	Yes	****	<0.0001
S100a8	Mature DCC vs. Pro-Senescent CC	2.358	-0.03156 to 4.747	No	ns	0.0577
S100a8	Mature DCC vs. Sec Prog	1.262	-1.912 to 4.437	No	ns	0.9933
S100a8	Mature DCC vs. Paneth-like	2.444	-0.7973 to 5.685	No	ns	0.4097
S100a8	Mature DCC vs. Goblet	2.171	-1.700 to 6.042	No	ns	0.8659
S100a8	Mature DCC vs. Endocrine	-3.505	-7.519 to 0.5089	No	ns	0.1720
S100a8	Mature DCC vs. Tuft	1.260	-5.785 to 8.305	No	ns	>0.9999
S100a8	Mature PCC vs. P-I CC	-9.896	-13.14 to -6.652	Yes	****	<0.0001
S100a8	Mature PCC vs. Pro-Senescent CC	0.3616	-2.153 to 2.876	No	ns	>0.9999
S100a8	Mature PCC vs. Sec Prog	-0.7334	-4.003 to 2.537	No	ns	>0.9999
S100a8	Mature PCC vs. Paneth-like	0.4481	-2.887 to 3.783	No	ns	>0.9999
S100a8	Mature PCC vs. Goblet	0.1750	-3.775 to 4.125	No	ns	>0.9999
S100a8	Mature PCC vs. Endocrine	-5.501	-9.591 to -1.411	Yes	***	0.0005
S100a8	Mature PCC vs. Tuft	-0.7361	-7.824 to 6.352	No	ns	>0.9999
S100a8	P-I CC vs. Pro-Senescent CC	10.26	7.300 to 13.22	Yes	****	<0.0001
S100a8	P-I CC vs. Sec Prog	9.163	5.541 to 12.78	Yes	****	<0.0001
S100a8	P-I CC vs. Paneth-like	10.34	6.664 to 14.02	Yes	****	<0.0001
S100a8	P-I CC vs. Goblet	10.07	5.826 to 14.32	Yes	****	<0.0001
S100a8	P-I CC vs. Endocrine	4.396	0.01943 to 8.772	Yes	*	0.0476
S100a8	P-I CC vs. Tuft	9.160	1.903 to 16.42	Yes	**	0.0017
S100a8	Pro-Senescent CC vs. Sec Prog	-1.095	-4.080 to 1.890	No	ns	0.9972
S100a8	Pro-Senescent CC vs. Paneth-like	0.08649	-2.970 to 3.143	No	ns	>0.9999
S100a8	Pro-Senescent CC vs. Goblet	-0.1865	-3.904 to 3.531	No	ns	>0.9999
S100a8	Pro-Senescent CC vs. Endocrine	-5.862	-9.728 to -1.996	Yes	****	<0.0001
S100a8	Pro-Senescent CC vs. Tuft	-1.098	-8.059 to 5.864	No	ns	>0.9999
S100a8	Sec Prog vs. Paneth-like	1.182	-2.521 to 4.884	No	ns	0.9994
S100a8	Sec Prog vs. Goblet	0.9085	-3.356 to 5.173	No	ns	>0.9999
S100a8	Sec Prog vs. Endocrine	-4.767	-9.162 to -0.3724	Yes	*	0.0187

S100a8	Sec Prog vs. Tuft	-0.002627	-7.271 to 7.266	No	ns	>0.9999
S100a8	Paneth-like vs. Goblet	-0.2730	-4.588 to 4.042	No	ns	>0.9999
S100a8	Paneth-like vs. Endocrine	-5.949	-10.39 to -1.505	Yes	***	0.0005
S100a8	Paneth-like vs. Tuft	-1.184	-8.482 to 6.114	No	ns	>0.9999
S100a8	Goblet vs. Endocrine	-5.676	-10.60 to -0.7542	Yes	**	0.0078
S100a8	Goblet vs. Tuft	-0.9111	-8.510 to 6.688	No	ns	>0.9999
S100a8	Endocrine vs. Tuft	4.765	-2.908 to 12.44	No	ns	0.7467

Gene	Tukey's multiple comparisons test	Mean Diff.	95.00% CI of diff.	Below threshold?	Summary	Adjusted P Value
Cxcl2	Lgr5-dim vs. Lgr5-hi	2.157	-0.3635 to 4.678	No	ns	0.1989
Cxcl2	Lgr5-dim vs. Early TA	1.022	-1.950 to 3.993	No	ns	0.9986
Cxcl2	Lgr5-dim vs. Abs Prog	-0.5707	-2.978 to 1.837	No	ns	>0.9999
Cxcl2	Lgr5-dim vs. Pre-DCC	0.6619	-1.556 to 2.880	No	ns	0.9997
Cxcl2	Lgr5-dim vs. Pre-PCC	4.032	0.8405 to 7.224	Yes	**	0.0016
Cxcl2	Lgr5-dim vs. Pro-DCC	0.8808	-1.970 to 3.731	No	ns	0.9996
Cxcl2	Lgr5-dim vs. Mature DCC	3.290	1.049 to 5.530	Yes	****	<0.0001
Cxcl2	Lgr5-dim vs. Mature PCC	3.974	1.639 to 6.309	Yes	****	<0.0001
Cxcl2	Lgr5-dim vs. P-I CC	1.798	-0.8778 to 4.474	No	ns	0.6214
Cxcl2	Lgr5-dim vs. Pro-Senescent CC	3.813	1.764 to 5.862	Yes	****	<0.0001
Cxcl2	Lgr5-dim vs. Sec Prog	2.363	-0.3349 to 5.060	No	ns	0.1681
Cxcl2	Lgr5-dim vs. Paneth-like	1.976	-0.7769 to 4.729	No	ns	0.5016
Cxcl2	Lgr5-dim vs. Goblet	3.478	0.2011 to 6.754	Yes	*	0.0248
Cxcl2	Lgr5-dim vs. Endocrine	1.211	-2.185 to 4.607	No	ns	0.9979
Cxcl2	Lgr5-dim vs. Tuft	4.289	-1.640 to 10.22	No	ns	0.4871
Cxcl2	Lgr5-hi vs. Early TA	-1.136	-4.296 to 2.025	No	ns	0.9978
Cxcl2	Lgr5-hi vs. Abs Prog	-2.728	-5.365 to -0.09066	Yes	*	0.0340
Cxcl2	Lgr5-hi vs. Pre-DCC	-1.495	-3.961 to 0.9700	No	ns	0.7789
Cxcl2	Lgr5-hi vs. Pre-PCC	1.875	-1.493 to 5.244	No	ns	0.8727
Cxcl2	Lgr5-hi vs. Pro-DCC	-1.276	-4.323 to 1.771	No	ns	0.9887
Cxcl2	Lgr5-hi vs. Mature DCC	1.132	-1.353 to 3.618	No	ns	0.9748
Cxcl2	Lgr5-hi vs. Mature PCC	1.817	-0.7543 to 4.388	No	ns	0.5308
Cxcl2	Lgr5-hi vs. P-I CC	-0.3589	-3.243 to 2.525	No	ns	>0.9999
Cxcl2	Lgr5-hi vs. Pro-Senescent CC	1.655	-0.6591 to 3.970	No	ns	0.5083
Cxcl2	Lgr5-hi vs. Sec Prog	0.2056	-2.699 to 3.110	No	ns	>0.9999
Cxcl2	Lgr5-hi vs. Paneth-like	-0.1808	-3.137 to 2.775	No	ns	>0.9999
Cxcl2	Lgr5-hi vs. Goblet	1.321	-2.128 to 4.769	No	ns	0.9955
Cxcl2	Lgr5-hi vs. Endocrine	-0.9462	-4.508 to 2.616	No	ns	>0.9999
Cxcl2	Lgr5-hi vs. Tuft	2.132	-3.894 to 8.157	No	ns	0.9981
Cxcl2	Early TA vs. Abs Prog	-1.592	-4.663 to 1.479	No	ns	0.9247
Cxcl2	Early TA vs. Pre-DCC	-0.3598	-3.285 to 2.565	No	ns	>0.9999
Cxcl2	Early TA vs. Pre-PCC	3.011	-0.7074 to 6.729	No	ns	0.2835
Cxcl2	Early TA vs. Pro-DCC	-0.1408	-3.570 to 3.289	No	ns	>0.9999
Cxcl2	Early TA vs. Mature DCC	2.268	-0.6740 to 5.210	No	ns	0.3691
Cxcl2	Early TA vs. Mature PCC	2.953	-0.06229 to 5.967	No	ns	0.0624
Cxcl2	Early TA vs. P-I CC	0.7766	-2.509 to 4.062	No	ns	>0.9999
Cxcl2	Early TA vs. Pro-Senescent CC	2.791	-0.008023 to 5.590	No	ns	0.0516
Cxcl2	Early TA vs. Sec Prog	1.341	-1.962 to 4.645	No	ns	0.9918
Cxcl2	Early TA vs. Paneth-like	0.9547	-2.394 to 4.304	No	ns	0.9999
Cxcl2	Early TA vs. Goblet	2.456	-1.335 to 6.247	No	ns	0.6826
Cxcl2	Early TA vs. Endocrine	0.1894	-3.705 to 4.084	No	ns	>0.9999
Cxcl2	Early TA vs. Tuft	3.267	-2.961 to 9.495	No	ns	0.9175
Cxcl2	Abs Prog vs. Pre-DCC	1.233	-1.117 to 3.582	No	ns	0.9175
Cxcl2	Abs Prog vs. Pre-PCC	4.603	1.318 to 7.887	Yes	***	0.0002
Cxcl2	Abs Prog vs. Pro-DCC	1.451	-1.503 to 4.406	No	ns	0.9512
Cxcl2	Abs Prog vs. Mature DCC	3.860	1.490 to 6.231	Yes	****	<0.0001
Cxcl2	Abs Prog vs. Mature PCC	4.545	2.084 to 7.005	Yes	****	<0.0001
Cxcl2	Abs Prog vs. P-I CC	2.369	-0.4171 to 5.155	No	ns	0.2080
Cxcl2	Abs Prog vs. Pro-Senescent CC	4.383	2.193 to 6.574	Yes	****	<0.0001
Cxcl2	Abs Prog vs. Sec Prog	2.933	0.1265 to 5.740	Yes	*	0.0301
Cxcl2	Abs Prog vs. Paneth-like	2.547	-0.3133 to 5.407	No	ns	0.1480
Cxcl2	Abs Prog vs. Goblet	4.048	0.6813 to 7.415	Yes	**	0.0039
Cxcl2	Abs Prog vs. Endocrine	1.782	-1.701 to 5.265	No	ns	0.9322

Cxcl2	Abs Prog vs. Tuft	4.859	-1.120 to 10.84	No	ns	0.2774
Cxcl2	Pre-DCC vs. Pre-PCC	3.370	0.2221 to 6.519	Yes	*	0.0222
Cxcl2	Pre-DCC vs. Pro-DCC	0.2189	-2.583 to 3.021	No	ns	>0.9999
Cxcl2	Pre-DCC vs. Mature DCC	2.628	0.4498 to 4.805	Yes	**	0.0037
Cxcl2	Pre-DCC vs. Mature PCC	3.312	1.037 to 5.588	Yes	****	<0.0001
Cxcl2	Pre-DCC vs. P-I CC	1.136	-1.488 to 3.760	No	ns	0.9844
Cxcl2	Pre-DCC vs. Pro-Senescent CC	3.151	1.170 to 5.131	Yes	****	<0.0001
Cxcl2	Pre-DCC vs. Sec Prog	1.701	-0.9452 to 4.347	No	ns	0.6951
Cxcl2	Pre-DCC vs. Paneth-like	1.314	-1.388 to 4.017	No	ns	0.9552
Cxcl2	Pre-DCC vs. Goblet	2.816	-0.4184 to 6.050	No	ns	0.1758
Cxcl2	Pre-DCC vs. Endocrine	0.5491	-2.806 to 3.904	No	ns	>0.9999
Cxcl2	Pre-DCC vs. Tuft	3.627	-2.279 to 9.532	No	ns	0.7622
Cxcl2	Pre-PCC vs. Pro-DCC	-3.151	-6.773 to 0.4704	No	ns	0.1765
Cxcl2	Pre-PCC vs. Mature DCC	-0.7427	-3.907 to 2.421	No	ns	>0.9999
Cxcl2	Pre-PCC vs. Mature PCC	-0.05809	-3.290 to 3.174	No	ns	>0.9999
Cxcl2	Pre-PCC vs. P-I CC	-2.234	-5.720 to 1.252	No	ns	0.6999
Cxcl2	Pre-PCC vs. Pro-Senescent CC	-0.2196	-3.251 to 2.812	No	ns	>0.9999
Cxcl2	Pre-PCC vs. Sec Prog	-1.669	-5.172 to 1.833	No	ns	0.9624
Cxcl2	Pre-PCC vs. Paneth-like	-2.056	-5.602 to 1.490	No	ns	0.8326
Cxcl2	Pre-PCC vs. Goblet	-0.5546	-4.520 to 3.411	No	ns	>0.9999
Cxcl2	Pre-PCC vs. Endocrine	-2.821	-6.886 to 1.244	No	ns	0.5637
Cxcl2	Pre-PCC vs. Tuft	0.2565	-6.079 to 6.592	No	ns	>0.9999
Cxcl2	Pro-DCC vs. Mature DCC	2.409	-0.4107 to 5.228	No	ns	0.2013
Cxcl2	Pro-DCC vs. Mature PCC	3.093	0.1979 to 5.989	Yes	*	0.0228
Cxcl2	Pro-DCC vs. P-I CC	0.9174	-2.259 to 4.094	No	ns	0.9998
Cxcl2	Pro-DCC vs. Pro-Senescent CC	2.932	0.2618 to 5.602	Yes	*	0.0158
Cxcl2	Pro-DCC vs. Sec Prog	1.482	-1.713 to 4.677	No	ns	0.9704
Cxcl2	Pro-DCC vs. Paneth-like	1.095	-2.146 to 4.337	No	ns	0.9989
Cxcl2	Pro-DCC vs. Goblet	2.597	-1.100 to 6.294	No	ns	0.5417
Cxcl2	Pro-DCC vs. Endocrine	0.3302	-3.472 to 4.133	No	ns	>0.9999
Cxcl2	Pro-DCC vs. Tuft	3.408	-2.763 to 9.579	No	ns	0.8796
Cxcl2	Mature DCC vs. Mature PCC	0.6846	-1.612 to 2.982	No	ns	0.9997
Cxcl2	Mature DCC vs. P-I CC	-1.491	-4.134 to 1.152	No	ns	0.8603
Cxcl2	Mature DCC vs. Pro-Senescent CC	0.5231	-1.482 to 2.529	No	ns	>0.9999
Cxcl2	Mature DCC vs. Sec Prog	-0.9268	-3.592 to 1.738	No	ns	0.9985
Cxcl2	Mature DCC vs. Paneth-like	-1.313	-4.034 to 1.408	No	ns	0.9581
Cxcl2	Mature DCC vs. Goblet	0.1882	-3.061 to 3.438	No	ns	>0.9999
Cxcl2	Mature DCC vs. Endocrine	-2.079	-5.448 to 1.291	No	ns	0.7561
Cxcl2	Mature DCC vs. Tuft	0.9993	-4.915 to 6.913	No	ns	>0.9999
Cxcl2	Mature PCC vs. P-I CC	-2.176	-4.900 to 0.5479	No	ns	0.3062
Cxcl2	Mature PCC vs. Pro-Senescent CC	-0.1615	-2.273 to 1.950	No	ns	>0.9999
Cxcl2	Mature PCC vs. Sec Prog	-1.611	-4.357 to 1.134	No	ns	0.8189
Cxcl2	Mature PCC vs. Paneth-like	-1.998	-4.798 to 0.8018	No	ns	0.5126
Cxcl2	Mature PCC vs. Goblet	-0.4965	-3.812 to 2.819	No	ns	>0.9999
Cxcl2	Mature PCC vs. Endocrine	-2.763	-6.196 to 0.6702	No	ns	0.2937
Cxcl2	Mature PCC vs. Tuft	0.3146	-5.636 to 6.265	No	ns	>0.9999
Cxcl2	P-I CC vs. Pro-Senescent CC	2.014	-0.4684 to 4.497	No	ns	0.2802
Cxcl2	P-I CC vs. Sec Prog	0.5645	-2.476 to 3.605	No	ns	>0.9999
Cxcl2	P-I CC vs. Paneth-like	0.1781	-2.912 to 3.268	No	ns	>0.9999
Cxcl2	P-I CC vs. Goblet	1.679	-1.885 to 5.243	No	ns	0.9659
Cxcl2	P-I CC vs. Endocrine	-0.5872	-4.261 to 3.086	No	ns	>0.9999
Cxcl2	P-I CC vs. Tuft	2.491	-3.602 to 8.583	No	ns	0.9912
Cxcl2	Pro-Senescent CC vs. Sec Prog	-1.450	-3.956 to 1.056	No	ns	0.8351
Cxcl2	Pro-Senescent CC vs. Paneth-like	-1.836	-4.402 to 0.7295	No	ns	0.5071
Cxcl2	Pro-Senescent CC vs. Goblet	-0.3350	-3.456 to 2.786	No	ns	>0.9999
Cxcl2	Pro-Senescent CC vs. Endocrine	-2.602	-5.847 to 0.6439	No	ns	0.3004

Cxcl2	Pro-Senescent CC vs. Tuft	0.4761	-5.368 to 6.320	No	ns	>0.9999
Cxcl2	Sec Prog vs. Paneth-like	-0.3865	-3.495 to 2.722	No	ns	>0.9999
Cxcl2	Sec Prog vs. Goblet	1.115	-2.465 to 4.695	No	ns	0.9996
Cxcl2	Sec Prog vs. Endocrine	-1.152	-4.841 to 2.538	No	ns	0.9996
Cxcl2	Sec Prog vs. Tuft	1.926	-4.176 to 8.028	No	ns	0.9995
Cxcl2	Paneth-like vs. Goblet	1.501	-2.121 to 5.124	No	ns	0.9898
Cxcl2	Paneth-like vs. Endocrine	-0.7653	-4.496 to 2.965	No	ns	>0.9999
Cxcl2	Paneth-like vs. Tuft	2.312	-3.814 to 8.439	No	ns	0.9962
Cxcl2	Goblet vs. Endocrine	-2.267	-6.398 to 1.865	No	ns	0.8851
Cxcl2	Goblet vs. Tuft	0.8111	-5.568 to 7.190	No	ns	>0.9999
Cxcl2	Endocrine vs. Tuft	3.078	-3.363 to 9.519	No	ns	0.9615

Gene	Tukey's multiple comparisons test	Mean Diff.	95.00% CI of diff.	Below threshold?	Summary	Adjusted P Value
Cxcl5	Lgr5-dim vs. Lgr5-hi	2.029	0.6741 to 3.385	Yes	****	<0.0001
Cxcl5	Lgr5-dim vs. Early TA	0.8674	-0.7306 to 2.465	No	ns	0.8936
Cxcl5	Lgr5-dim vs. Abs Prog	-0.2852	-1.580 to 1.009	No	ns	>0.9999
Cxcl5	Lgr5-dim vs. Pre-DCC	1.024	-0.1692 to 2.217	No	ns	0.1950
Cxcl5	Lgr5-dim vs. Pre-PCC	2.323	0.6069 to 4.039	Yes	***	0.0004
Cxcl5	Lgr5-dim vs. Pro-DCC	-0.2150	-1.748 to 1.318	No	ns	>0.9999
Cxcl5	Lgr5-dim vs. Mature DCC	1.667	0.4624 to 2.872	Yes	***	0.0002
Cxcl5	Lgr5-dim vs. Mature PCC	2.563	1.308 to 3.819	Yes	****	<0.0001
Cxcl5	Lgr5-dim vs. P-I CC	-1.045	-2.484 to 0.3942	No	ns	0.4803
Cxcl5	Lgr5-dim vs. Pro-Senescent CC	2.398	1.296 to 3.499	Yes	****	<0.0001
Cxcl5	Lgr5-dim vs. Sec Prog	2.425	0.9747 to 3.876	Yes	****	<0.0001
Cxcl5	Lgr5-dim vs. Paneth-like	2.715	1.235 to 4.196	Yes	****	<0.0001
Cxcl5	Lgr5-dim vs. Goblet	1.934	0.1723 to 3.696	Yes	*	0.0159
Cxcl5	Lgr5-dim vs. Endocrine	2.860	1.035 to 4.686	Yes	****	<0.0001
Cxcl5	Lgr5-dim vs. Tuft	2.895	-0.2927 to 6.083	No	ns	0.1261
Cxcl5	Lgr5-hi vs. Early TA	-1.162	-2.862 to 0.5375	No	ns	0.5907
Cxcl5	Lgr5-hi vs. Abs Prog	-2.315	-3.733 to -0.8966	Yes	****	<0.0001
Cxcl5	Lgr5-hi vs. Pre-DCC	-1.006	-2.332 to 0.3204	No	ns	0.3990
Cxcl5	Lgr5-hi vs. Pre-PCC	0.2937	-1.518 to 2.105	No	ns	>0.9999
Cxcl5	Lgr5-hi vs. Pro-DCC	-2.244	-3.883 to -0.6061	Yes	***	0.0003
Cxcl5	Lgr5-hi vs. Mature DCC	-0.3625	-1.699 to 0.9740	No	ns	>0.9999
Cxcl5	Lgr5-hi vs. Mature PCC	0.5340	-0.8486 to 1.917	No	ns	0.9951
Cxcl5	Lgr5-hi vs. P-I CC	-3.074	-4.625 to -1.523	Yes	****	<0.0001
Cxcl5	Lgr5-hi vs. Pro-Senescent CC	0.3682	-0.8764 to 1.613	No	ns	0.9998
Cxcl5	Lgr5-hi vs. Sec Prog	0.3959	-1.166 to 1.958	No	ns	>0.9999
Cxcl5	Lgr5-hi vs. Paneth-like	0.6858	-0.9038 to 2.275	No	ns	0.9849
Cxcl5	Lgr5-hi vs. Goblet	-0.09530	-1.950 to 1.759	No	ns	>0.9999
Cxcl5	Lgr5-hi vs. Endocrine	0.8310	-1.084 to 2.746	No	ns	0.9841
Cxcl5	Lgr5-hi vs. Tuft	0.8658	-2.374 to 4.106	No	ns	>0.9999
Cxcl5	Early TA vs. Abs Prog	-1.153	-2.804 to 0.4989	No	ns	0.5537
Cxcl5	Early TA vs. Pre-DCC	0.1565	-1.417 to 1.730	No	ns	>0.9999
Cxcl5	Early TA vs. Pre-PCC	1.456	-0.5435 to 3.455	No	ns	0.4748
Cxcl5	Early TA vs. Pro-DCC	-1.082	-2.926 to 0.7617	No	ns	0.8191
Cxcl5	Early TA vs. Mature DCC	0.7996	-0.7823 to 2.382	No	ns	0.9384
Cxcl5	Early TA vs. Mature PCC	1.696	0.07500 to 3.317	Yes	*	0.0296
Cxcl5	Early TA vs. P-I CC	-1.912	-3.679 to -0.1452	Yes	*	0.0193
Cxcl5	Early TA vs. Pro-Senescent CC	1.530	0.02518 to 3.035	Yes	*	0.0416
Cxcl5	Early TA vs. Sec Prog	1.558	-0.2184 to 3.334	No	ns	0.1664
Cxcl5	Early TA vs. Paneth-like	1.848	0.04700 to 3.649	Yes	*	0.0374
Cxcl5	Early TA vs. Goblet	1.067	-0.9717 to 3.105	No	ns	0.9190
Cxcl5	Early TA vs. Endocrine	1.993	-0.1010 to 4.087	No	ns	0.0829
Cxcl5	Early TA vs. Tuft	2.028	-1.321 to 5.377	No	ns	0.7810
Cxcl5	Abs Prog vs. Pre-DCC	1.309	0.04525 to 2.573	Yes	*	0.0335
Cxcl5	Abs Prog vs. Pre-PCC	2.608	0.8421 to 4.374	Yes	****	<0.0001
Cxcl5	Abs Prog vs. Pro-DCC	0.07017	-1.518 to 1.659	No	ns	>0.9999
Cxcl5	Abs Prog vs. Mature DCC	1.952	0.6774 to 3.227	Yes	****	<0.0001
Cxcl5	Abs Prog vs. Mature PCC	2.849	1.526 to 4.172	Yes	****	<0.0001
Cxcl5	Abs Prog vs. P-I CC	-0.7595	-2.258 to 0.7386	No	ns	0.9369
Cxcl5	Abs Prog vs. Pro-Senescent CC	2.683	1.505 to 3.861	Yes	****	<0.0001

Cxcl5	Abs Prog vs. Sec Prog	2.711	1.201 to 4.220	Yes	****	<0.0001
Cxcl5	Abs Prog vs. Paneth-like	3.000	1.462 to 4.538	Yes	****	<0.0001
Cxcl5	Abs Prog vs. Goblet	2.219	0.4088 to 4.030	Yes	**	0.0028
Cxcl5	Abs Prog vs. Endocrine	3.146	1.273 to 5.018	Yes	****	<0.0001
Cxcl5	Abs Prog vs. Tuft	3.180	-0.03471 to 6.396	No	ns	0.0562
Cxcl5	Pre-DCC vs. Pre-PCC	1.299	-0.3939 to 2.992	No	ns	0.3774
Cxcl5	Pre-DCC vs. Pro-DCC	-1.239	-2.746 to 0.2680	No	ns	0.2588
Cxcl5	Pre-DCC vs. Mature DCC	0.6431	-0.5284 to 1.815	No	ns	0.8846
Cxcl5	Pre-DCC vs. Mature PCC	1.540	0.3157 to 2.764	Yes	**	0.0018
Cxcl5	Pre-DCC vs. P-I CC	-2.069	-3.480 to -0.6572	Yes	****	<0.0001
Cxcl5	Pre-DCC vs. Pro-Senescent CC	1.374	0.3083 to 2.439	Yes	**	0.0011
Cxcl5	Pre-DCC vs. Sec Prog	1.401	-0.02172 to 2.825	No	ns	0.0589
Cxcl5	Pre-DCC vs. Paneth-like	1.691	0.2378 to 3.145	Yes	**	0.0067
Cxcl5	Pre-DCC vs. Goblet	0.9103	-0.8291 to 2.650	No	ns	0.9190
Cxcl5	Pre-DCC vs. Endocrine	1.837	0.03244 to 3.641	Yes	*	0.0410
Cxcl5	Pre-DCC vs. Tuft	1.871	-1.304 to 5.047	No	ns	0.8144
Cxcl5	Pre-PCC vs. Pro-DCC	-2.538	-4.486 to -0.5906	Yes	***	0.0009
Cxcl5	Pre-PCC vs. Mature DCC	-0.6561	-2.358 to 1.045	No	ns	0.9952
Cxcl5	Pre-PCC vs. Mature PCC	0.2404	-1.498 to 1.978	No	ns	>0.9999
Cxcl5	Pre-PCC vs. P-I CC	-3.368	-5.242 to -1.493	Yes	****	<0.0001
Cxcl5	Pre-PCC vs. Pro-Senescent CC	0.07450	-1.556 to 1.705	No	ns	>0.9999
Cxcl5	Pre-PCC vs. Sec Prog	0.1022	-1.781 to 1.986	No	ns	>0.9999
Cxcl5	Pre-PCC vs. Paneth-like	0.3921	-1.515 to 2.299	No	ns	>0.9999
Cxcl5	Pre-PCC vs. Goblet	-0.3890	-2.522 to 1.744	No	ns	>0.9999
Cxcl5	Pre-PCC vs. Endocrine	0.5373	-1.648 to 2.723	No	ns	>0.9999
Cxcl5	Pre-PCC vs. Tuft	0.5721	-2.835 to 3.979	No	ns	>0.9999
Cxcl5	Pro-DCC vs. Mature DCC	1.882	0.3659 to 3.398	Yes	**	0.0023
Cxcl5	Pro-DCC vs. Mature PCC	2.778	1.222 to 4.335	Yes	****	<0.0001
Cxcl5	Pro-DCC vs. P-I CC	-0.8297	-2.538 to 0.8785	No	ns	0.9557
Cxcl5	Pro-DCC vs. Pro-Senescent CC	2.613	1.177 to 4.048	Yes	****	<0.0001
Cxcl5	Pro-DCC vs. Sec Prog	2.640	0.9223 to 4.358	Yes	****	<0.0001
Cxcl5	Pro-DCC vs. Paneth-like	2.930	1.187 to 4.674	Yes	****	<0.0001
Cxcl5	Pro-DCC vs. Goblet	2.149	0.1613 to 4.137	Yes	*	0.0195
Cxcl5	Pro-DCC vs. Endocrine	3.075	1.031 to 5.120	Yes	****	<0.0001
Cxcl5	Pro-DCC vs. Tuft	3.110	-0.2080 to 6.429	No	ns	0.0956
Cxcl5	Mature DCC vs. Mature PCC	0.8965	-0.3387 to 2.132	No	ns	0.4809
Cxcl5	Mature DCC vs. P-I CC	-2.712	-4.133 to -1.291	Yes	****	<0.0001
Cxcl5	Mature DCC vs. Pro-Senescent CC	0.7307	-0.3478 to 1.809	No	ns	0.6070
Cxcl5	Mature DCC vs. Sec Prog	0.7584	-0.6746 to 2.191	No	ns	0.9118
Cxcl5	Mature DCC vs. Paneth-like	1.048	-0.4149 to 2.511	No	ns	0.5052
Cxcl5	Mature DCC vs. Goblet	0.2672	-1.480 to 2.015	No	ns	>0.9999
Cxcl5	Mature DCC vs. Endocrine	1.193	-0.6184 to 3.005	No	ns	0.6554
Cxcl5	Mature DCC vs. Tuft	1.228	-1.952 to 4.408	No	ns	0.9951
Cxcl5	Mature PCC vs. P-I CC	-3.608	-5.073 to -2.144	Yes	****	<0.0001
Cxcl5	Mature PCC vs. Pro-Senescent CC	-0.1659	-1.301 to 0.9693	No	ns	>0.9999
Cxcl5	Mature PCC vs. Sec Prog	-0.1382	-1.614 to 1.338	No	ns	>0.9999
Cxcl5	Mature PCC vs. Paneth-like	0.1517	-1.354 to 1.657	No	ns	>0.9999
Cxcl5	Mature PCC vs. Goblet	-0.6293	-2.412 to 1.154	No	ns	0.9982
Cxcl5	Mature PCC vs. Endocrine	0.2970	-1.549 to 2.143	No	ns	>0.9999
Cxcl5	Mature PCC vs. Tuft	0.3318	-2.868 to 3.531	No	ns	>0.9999
Cxcl5	P-I CC vs. Pro-Senescent CC	3.442	2.107 to 4.777	Yes	****	<0.0001
Cxcl5	P-I CC vs. Sec Prog	3.470	1.835 to 5.105	Yes	****	<0.0001
Cxcl5	P-I CC vs. Paneth-like	3.760	2.099 to 5.421	Yes	****	<0.0001
Cxcl5	P-I CC vs. Goblet	2.979	1.062 to 4.895	Yes	****	<0.0001
Cxcl5	P-I CC vs. Endocrine	3.905	1.930 to 5.881	Yes	****	<0.0001
Cxcl5	P-I CC vs. Tuft	3.940	0.6640 to 7.216	Yes	**	0.0039
Cxcl5	Pro-Senescent CC vs. Sec Prog	0.02770	-1.320 to 1.375	No	ns	>0.9999
Cxcl5	Pro-Senescent CC vs. Paneth-like	0.3176	-1.062 to 1.697	No	ns	>0.9999

Cxcl5	Pro-Senescent CC vs. Goblet	-0.4635	-2.142 to 1.215	No	ns	>0.9999
Cxcl5	Pro-Senescent CC vs. Endocrine	0.4628	-1.282 to 2.208	No	ns	>0.9999
Cxcl5	Pro-Senescent CC vs. Tuft	0.4976	-2.645 to 3.640	No	ns	>0.9999
Cxcl5	Sec Prog vs. Paneth-like	0.2899	-1.382 to 1.961	No	ns	>0.9999
Cxcl5	Sec Prog vs. Goblet	-0.4912	-2.416 to 1.434	No	ns	>0.9999
Cxcl5	Sec Prog vs. Endocrine	0.4351	-1.549 to 2.419	No	ns	>0.9999
Cxcl5	Sec Prog vs. Tuft	0.4699	-2.811 to 3.751	No	ns	>0.9999
Cxcl5	Paneth-like vs. Goblet	-0.7811	-2.729 to 1.167	No	ns	0.9927
Cxcl5	Paneth-like vs. Endocrine	0.1452	-1.861 to 2.151	No	ns	>0.9999
Cxcl5	Paneth-like vs. Tuft	0.1800	-3.114 to 3.474	No	ns	>0.9999
Cxcl5	Goblet vs. Endocrine	0.9263	-1.295 to 3.148	No	ns	0.9892
Cxcl5	Goblet vs. Tuft	0.9611	-2.469 to 4.391	No	ns	0.9999
Cxcl5	Endocrine vs. Tuft	0.03482	-3.429 to 3.498	No	ns	>0.9999

Gene	Tukey's multiple comparisons test	Mean Diff.	95.00% CI of diff.	Below threshold?	Summary	Adjusted P Value
Lrg1	Lgr5-dim vs. Lgr5-hi	5.353	3.359 to 7.348	Yes	****	<0.0001
Lrg1	Lgr5-dim vs. Early TA	3.839	1.488 to 6.190	Yes	****	<0.0001
Lrg1	Lgr5-dim vs. Abs Prog	-4.988	-6.893 to -3.083	Yes	****	<0.0001
Lrg1	Lgr5-dim vs. Pre-DCC	-10.65	-12.41 to -8.898	Yes	****	<0.0001
Lrg1	Lgr5-dim vs. Pre-PCC	2.656	0.1305 to 5.182	Yes	*	0.0278
Lrg1	Lgr5-dim vs. Pro-DCC	-9.638	-11.89 to -7.383	Yes	****	<0.0001
Lrg1	Lgr5-dim vs. Mature DCC	5.934	4.161 to 7.706	Yes	****	<0.0001
Lrg1	Lgr5-dim vs. Mature PCC	6.820	4.972 to 8.667	Yes	****	<0.0001
Lrg1	Lgr5-dim vs. P-I CC	6.011	3.894 to 8.129	Yes	****	<0.0001
Lrg1	Lgr5-dim vs. Pro-Senescent CC	7.224	5.603 to 8.846	Yes	****	<0.0001
Lrg1	Lgr5-dim vs. Sec Prog	1.998	-0.1367 to 4.132	No	ns	0.0969
Lrg1	Lgr5-dim vs. Paneth-like	-2.062	-4.240 to 0.1168	No	ns	0.0874
Lrg1	Lgr5-dim vs. Goblet	0.4671	-2.125 to 3.060	No	ns	>0.9999
Lrg1	Lgr5-dim vs. Endocrine	6.790	4.103 to 9.477	Yes	****	<0.0001
Lrg1	Lgr5-dim vs. Tuft	7.300	2.609 to 11.99	Yes	****	<0.0001
Lrg1	Lgr5-hi vs. Early TA	-1.514	-4.015 to 0.9865	No	ns	0.7810
Lrg1	Lgr5-hi vs. Abs Prog	-10.34	-12.43 to -8.255	Yes	****	<0.0001
Lrg1	Lgr5-hi vs. Pre-DCC	-16.01	-17.96 to -14.06	Yes	****	<0.0001
Lrg1	Lgr5-hi vs. Pre-PCC	-2.697	-5.363 to -0.03213	Yes	*	0.0438
Lrg1	Lgr5-hi vs. Pro-DCC	-14.99	-17.40 to -12.58	Yes	****	<0.0001
Lrg1	Lgr5-hi vs. Mature DCC	0.5803	-1.386 to 2.547	No	ns	0.9998
Lrg1	Lgr5-hi vs. Mature PCC	1.466	-0.5683 to 3.501	No	ns	0.4941
Lrg1	Lgr5-hi vs. P-I CC	0.6577	-1.625 to 2.940	No	ns	0.9998
Lrg1	Lgr5-hi vs. Pro-Senescent CC	1.871	0.03946 to 3.702	Yes	*	0.0394
Lrg1	Lgr5-hi vs. Sec Prog	-3.356	-5.654 to -1.057	Yes	****	<0.0001
Lrg1	Lgr5-hi vs. Paneth-like	-7.415	-9.754 to -5.076	Yes	****	<0.0001
Lrg1	Lgr5-hi vs. Goblet	-4.886	-7.615 to -2.157	Yes	****	<0.0001
Lrg1	Lgr5-hi vs. Endocrine	1.437	-1.382 to 4.255	No	ns	0.9341
Lrg1	Lgr5-hi vs. Tuft	1.947	-2.821 to 6.715	No	ns	0.9913
Lrg1	Early TA vs. Abs Prog	-8.827	-11.26 to -6.397	Yes	****	<0.0001
Lrg1	Early TA vs. Pre-DCC	-14.49	-16.81 to -12.18	Yes	****	<0.0001
Lrg1	Early TA vs. Pre-PCC	-1.183	-4.125 to 1.759	No	ns	0.9925
Lrg1	Early TA vs. Pro-DCC	-13.48	-16.19 to -10.76	Yes	****	<0.0001
Lrg1	Early TA vs. Mature DCC	2.095	-0.2331 to 4.423	No	ns	0.1360
Lrg1	Early TA vs. Mature PCC	2.981	0.5952 to 5.366	Yes	**	0.0020
Lrg1	Early TA vs. P-I CC	2.172	-0.4278 to 4.772	No	ns	0.2339
Lrg1	Early TA vs. Pro-Senescent CC	3.385	1.171 to 5.600	Yes	****	<0.0001
Lrg1	Early TA vs. Sec Prog	-1.841	-4.455 to 0.7729	No	ns	0.5368
Lrg1	Early TA vs. Paneth-like	-5.901	-8.551 to -3.251	Yes	****	<0.0001
Lrg1	Early TA vs. Goblet	-3.372	-6.372 to -0.3722	Yes	*	0.0114
Lrg1	Early TA vs. Endocrine	2.951	-0.1303 to 6.033	No	ns	0.0780
Lrg1	Early TA vs. Tuft	3.461	-1.466 to 8.389	No	ns	0.5418
Lrg1	Abs Prog vs. Pre-DCC	-5.665	-7.524 to -3.806	Yes	****	<0.0001
Lrg1	Abs Prog vs. Pre-PCC	7.644	5.045 to 10.24	Yes	****	<0.0001
Lrg1	Abs Prog vs. Pro-DCC	-4.650	-6.988 to -2.313	Yes	****	<0.0001

Lrg1	Abs Prog vs. Mature DCC	10.92	9.046 to 12.80	Yes	****	<0.0001
Lrg1	Abs Prog vs. Mature PCC	11.81	9.861 to 13.75	Yes	****	<0.0001
Lrg1	Abs Prog vs. P-I CC	11.00	8.795 to 13.20	Yes	****	<0.0001
Lrg1	Abs Prog vs. Pro-Senescent CC	12.21	10.48 to 13.95	Yes	****	<0.0001
Lrg1	Abs Prog vs. Sec Prog	6.986	4.765 to 9.207	Yes	****	<0.0001
Lrg1	Abs Prog vs. Paneth-like	2.926	0.6632 to 5.190	Yes	**	0.0010
Lrg1	Abs Prog vs. Goblet	5.455	2.791 to 8.119	Yes	****	<0.0001
Lrg1	Abs Prog vs. Endocrine	11.78	9.022 to 14.53	Yes	****	<0.0001
Lrg1	Abs Prog vs. Tuft	12.29	7.557 to 17.02	Yes	****	<0.0001
Lrg1	Pre-DCC vs. Pre-PCC	13.31	10.82 to 15.80	Yes	****	<0.0001
Lrg1	Pre-DCC vs. Pro-DCC	1.015	-1.202 to 3.232	No	ns	0.9738
Lrg1	Pre-DCC vs. Mature DCC	16.59	14.86 to 18.31	Yes	****	<0.0001
Lrg1	Pre-DCC vs. Mature PCC	17.47	15.67 to 19.27	Yes	****	<0.0001
Lrg1	Pre-DCC vs. P-I CC	16.66	14.59 to 18.74	Yes	****	<0.0001
Lrg1	Pre-DCC vs. Pro-Senescent CC	17.88	16.31 to 19.44	Yes	****	<0.0001
Lrg1	Pre-DCC vs. Sec Prog	12.65	10.56 to 14.74	Yes	****	<0.0001
Lrg1	Pre-DCC vs. Paneth-like	8.591	6.453 to 10.73	Yes	****	<0.0001
Lrg1	Pre-DCC vs. Goblet	11.12	8.561 to 13.68	Yes	****	<0.0001
Lrg1	Pre-DCC vs. Endocrine	17.44	14.79 to 20.10	Yes	****	<0.0001
Lrg1	Pre-DCC vs. Tuft	17.95	13.28 to 22.63	Yes	****	<0.0001
Lrg1	Pre-PCC vs. Pro-DCC	-12.29	-15.16 to -9.428	Yes	****	<0.0001
Lrg1	Pre-PCC vs. Mature DCC	3.278	0.7741 to 5.781	Yes	***	0.0008
Lrg1	Pre-PCC vs. Mature PCC	4.164	1.606 to 6.721	Yes	****	<0.0001
Lrg1	Pre-PCC vs. P-I CC	3.355	0.5966 to 6.114	Yes	**	0.0032
Lrg1	Pre-PCC vs. Pro-Senescent CC	4.568	2.169 to 6.967	Yes	****	<0.0001
Lrg1	Pre-PCC vs. Sec Prog	-0.6581	-3.430 to 2.114	No	ns	>0.9999
Lrg1	Pre-PCC vs. Paneth-like	-4.718	-7.523 to -1.912	Yes	****	<0.0001
Lrg1	Pre-PCC vs. Goblet	-2.189	-5.327 to 0.9492	No	ns	0.5547
Lrg1	Pre-PCC vs. Endocrine	4.134	0.9178 to 7.350	Yes	**	0.0012
Lrg1	Pre-PCC vs. Tuft	4.644	-0.3689 to 9.658	No	ns	0.1062
Lrg1	Pro-DCC vs. Mature DCC	15.57	13.34 to 17.80	Yes	****	<0.0001
Lrg1	Pro-DCC vs. Mature PCC	16.46	14.17 to 18.75	Yes	****	<0.0001
Lrg1	Pro-DCC vs. P-I CC	15.65	13.14 to 18.16	Yes	****	<0.0001
Lrg1	Pro-DCC vs. Pro-Senescent CC	16.86	14.75 to 18.98	Yes	****	<0.0001
Lrg1	Pro-DCC vs. Sec Prog	11.64	9.108 to 14.16	Yes	****	<0.0001
Lrg1	Pro-DCC vs. Paneth-like	7.577	5.011 to 10.14	Yes	****	<0.0001
Lrg1	Pro-DCC vs. Goblet	10.11	7.180 to 13.03	Yes	****	<0.0001
Lrg1	Pro-DCC vs. Endocrine	16.43	13.42 to 19.44	Yes	****	<0.0001
Lrg1	Pro-DCC vs. Tuft	16.94	12.06 to 21.82	Yes	****	<0.0001
Lrg1	Mature DCC vs. Mature PCC	0.8861	-0.9316 to 2.704	No	ns	0.9544
Lrg1	Mature DCC vs. P-I CC	0.07745	-2.014 to 2.169	No	ns	>0.9999
Lrg1	Mature DCC vs. Pro-Senescent CC	1.291	-0.2963 to 2.878	No	ns	0.2763
Lrg1	Mature DCC vs. Sec Prog	-3.936	-6.044 to -1.827	Yes	****	<0.0001
Lrg1	Mature DCC vs. Paneth-like	-7.995	-10.15 to -5.842	Yes	****	<0.0001
Lrg1	Mature DCC vs. Goblet	-5.467	-8.038 to -2.895	Yes	****	<0.0001
Lrg1	Mature DCC vs. Endocrine	0.8565	-1.810 to 3.523	No	ns	0.9994
Lrg1	Mature DCC vs. Tuft	1.367	-3.313 to 6.046	No	ns	0.9998
Lrg1	Mature PCC vs. P-I CC	-0.8086	-2.964 to 1.347	No	ns	0.9964
Lrg1	Mature PCC vs. Pro-Senescent CC	0.4046	-1.266 to 2.075	No	ns	>0.9999
Lrg1	Mature PCC vs. Sec Prog	-4.822	-6.994 to -2.650	Yes	****	<0.0001
Lrg1	Mature PCC vs. Paneth-like	-8.881	-11.10 to -6.666	Yes	****	<0.0001
Lrg1	Mature PCC vs. Goblet	-6.353	-8.976 to -3.729	Yes	****	<0.0001
Lrg1	Mature PCC vs. Endocrine	-0.02960	-2.746 to 2.687	No	ns	>0.9999
Lrg1	Mature PCC vs. Tuft	0.4807	-4.228 to 5.189	No	ns	>0.9999
Lrg1	P-I CC vs. Pro-Senescent CC	1.213	-0.7514 to 3.178	No	ns	0.7547
Lrg1	P-I CC vs. Sec Prog	-4.013	-6.419 to -1.608	Yes	****	<0.0001
Lrg1	P-I CC vs. Paneth-like	-8.073	-10.52 to -5.628	Yes	****	<0.0001
Lrg1	P-I CC vs. Goblet	-5.544	-8.364 to -2.724	Yes	****	<0.0001
Lrg1	P-I CC vs. Endocrine	0.7790	-2.128 to 3.686	No	ns	>0.9999

Lrg1	P-I CC vs. Tuft	1.289	-3.531 to 6.110	No	ns	>0.9999
Lrg1	Pro-Senescent CC vs. Sec Prog	-5.226	-7.210 to -3.243	Yes	****	<0.0001
Lrg1	Pro-Senescent CC vs. Paneth-like	-9.286	-11.32 to -7.256	Yes	****	<0.0001
Lrg1	Pro-Senescent CC vs. Goblet	-6.757	-9.227 to -4.288	Yes	****	<0.0001
Lrg1	Pro-Senescent CC vs. Endocrine	-0.4342	-3.002 to 2.134	No	ns	>0.9999
Lrg1	Pro-Senescent CC vs. Tuft	0.07614	-4.548 to 4.700	No	ns	>0.9999
Lrg1	Sec Prog vs. Paneth-like	-4.060	-6.519 to -1.600	Yes	****	<0.0001
Lrg1	Sec Prog vs. Goblet	-1.531	-4.364 to 1.302	No	ns	0.8971
Lrg1	Sec Prog vs. Endocrine	4.792	1.873 to 7.712	Yes	****	<0.0001
Lrg1	Sec Prog vs. Tuft	5.303	0.4744 to 10.13	Yes	*	0.0158
Lrg1	Paneth-like vs. Goblet	2.529	-0.3374 to 5.395	No	ns	0.1590
Lrg1	Paneth-like vs. Endocrine	8.852	5.900 to 11.80	Yes	****	<0.0001
Lrg1	Paneth-like vs. Tuft	9.362	4.514 to 14.21	Yes	****	<0.0001
Lrg1	Goblet vs. Endocrine	6.323	3.054 to 9.592	Yes	****	<0.0001
Lrg1	Goblet vs. Tuft	6.833	1.786 to 11.88	Yes	***	0.0004
Lrg1	Endocrine vs. Tuft	0.5103	-4.586 to 5.607	No	ns	>0.9999

Gene	Tukey's multiple comparisons test	Mean Diff.	95.00% CI of diff.	Below threshold?	Summary	Adjusted P Value
Fut2	Lgr5-dim vs. Lgr5-hi	0.7497	0.2878 to 1.212	Yes	****	<0.0001
Fut2	Lgr5-dim vs. Early TA	0.6145	0.07003 to 1.159	Yes	*	0.0107
Fut2	Lgr5-dim vs. Abs Prog	-0.6146	-1.056 to -0.1735	Yes	***	0.0002
Fut2	Lgr5-dim vs. Pre-DCC	-1.550	-1.956 to -1.144	Yes	****	<0.0001
Fut2	Lgr5-dim vs. Pre-PCC	-1.252	-1.837 to -0.6675	Yes	****	<0.0001
Fut2	Lgr5-dim vs. Pro-DCC	-1.105	-1.627 to -0.5825	Yes	****	<0.0001
Fut2	Lgr5-dim vs. Mature DCC	0.2825	-0.1280 to 0.6930	No	ns	0.5790
Fut2	Lgr5-dim vs. Mature PCC	0.4369	0.009053 to 0.8648	Yes	*	0.0395
Fut2	Lgr5-dim vs. P-I CC	0.4300	-0.06030 to 0.9203	No	ns	0.1664
Fut2	Lgr5-dim vs. Pro-Senescent CC	0.9233	0.5479 to 1.299	Yes	****	<0.0001
Fut2	Lgr5-dim vs. Sec Prog	-2.925	-3.419 to -2.431	Yes	****	<0.0001
Fut2	Lgr5-dim vs. Paneth-like	-5.820	-6.325 to -5.316	Yes	****	<0.0001
Fut2	Lgr5-dim vs. Goblet	-1.985	-2.585 to -1.384	Yes	****	<0.0001
Fut2	Lgr5-dim vs. Endocrine	0.4372	-0.1850 to 1.059	No	ns	0.5412
Fut2	Lgr5-dim vs. Tuft	0.9933	-0.09309 to 2.080	No	ns	0.1191
Fut2	Lgr5-hi vs. Early TA	-0.1351	-0.7143 to 0.4440	No	ns	>0.9999
Fut2	Lgr5-hi vs. Abs Prog	-1.364	-1.847 to -0.8811	Yes	****	<0.0001
Fut2	Lgr5-hi vs. Pre-DCC	-2.300	-2.751 to -1.848	Yes	****	<0.0001
Fut2	Lgr5-hi vs. Pre-PCC	-2.002	-2.619 to -1.385	Yes	****	<0.0001
Fut2	Lgr5-hi vs. Pro-DCC	-1.855	-2.413 to -1.296	Yes	****	<0.0001
Fut2	Lgr5-hi vs. Mature DCC	-0.4672	-0.9226 to -0.01174	Yes	*	0.0375
Fut2	Lgr5-hi vs. Mature PCC	-0.3127	-0.7839 to 0.1584	No	ns	0.6424
Fut2	Lgr5-hi vs. P-I CC	-0.3197	-0.8482 to 0.2089	No	ns	0.7826
Fut2	Lgr5-hi vs. Pro-Senescent CC	0.1736	-0.2505 to 0.5977	No	ns	0.9910
Fut2	Lgr5-hi vs. Sec Prog	-3.675	-4.207 to -3.142	Yes	****	<0.0001
Fut2	Lgr5-hi vs. Paneth-like	-6.570	-7.112 to -6.028	Yes	****	<0.0001
Fut2	Lgr5-hi vs. Goblet	-2.734	-3.366 to -2.102	Yes	****	<0.0001
Fut2	Lgr5-hi vs. Endocrine	-0.3125	-0.9652 to 0.3402	No	ns	0.9609
Fut2	Lgr5-hi vs. Tuft	0.2436	-0.8605 to 1.348	No	ns	>0.9999
Fut2	Early TA vs. Abs Prog	-1.229	-1.792 to -0.6664	Yes	****	<0.0001
Fut2	Early TA vs. Pre-DCC	-2.165	-2.700 to -1.629	Yes	****	<0.0001
Fut2	Early TA vs. Pre-PCC	-1.867	-2.548 to -1.186	Yes	****	<0.0001
Fut2	Early TA vs. Pro-DCC	-1.719	-2.348 to -1.091	Yes	****	<0.0001
Fut2	Early TA vs. Mature DCC	-0.3320	-0.8711 to 0.2070	No	ns	0.7582
Fut2	Early TA vs. Mature PCC	-0.1776	-0.7300 to 0.3748	No	ns	0.9994
Fut2	Early TA vs. P-I CC	-0.1845	-0.7866 to 0.4176	No	ns	0.9996
Fut2	Early TA vs. Pro-Senescent CC	0.3088	-0.2041 to 0.8217	No	ns	0.7884
Fut2	Early TA vs. Sec Prog	-3.539	-4.145 to -2.934	Yes	****	<0.0001
Fut2	Early TA vs. Paneth-like	-6.435	-7.049 to -5.821	Yes	****	<0.0001
Fut2	Early TA vs. Goblet	-2.599	-3.294 to -1.904	Yes	****	<0.0001

Fut2	Early TA vs. Endocrine	-0.1774	-0.8909 to 0.5362	No	ns	>0.9999
Fut2	Early TA vs. Tuft	0.3787	-0.7624 to 1.520	No	ns	0.9991
Fut2	Abs Prog vs. Pre-DCC	-0.9354	-1.366 to -0.5049	Yes	****	<0.0001
Fut2	Abs Prog vs. Pre-PCC	-0.6377	-1.240 to -0.03589	Yes	*	0.0253
Fut2	Abs Prog vs. Pro-DCC	-0.4903	-1.032 to 0.05102	No	ns	0.1289
Fut2	Abs Prog vs. Mature DCC	0.8971	0.4627 to 1.331	Yes	****	<0.0001
Fut2	Abs Prog vs. Mature PCC	1.052	0.6007 to 1.502	Yes	****	<0.0001
Fut2	Abs Prog vs. P-I CC	1.045	0.5341 to 1.555	Yes	****	<0.0001
Fut2	Abs Prog vs. Pro-Senescent CC	1.538	1.136 to 1.939	Yes	****	<0.0001
Fut2	Abs Prog vs. Sec Prog	-2.310	-2.825 to -1.796	Yes	****	<0.0001
Fut2	Abs Prog vs. Paneth-like	-5.206	-5.730 to -4.682	Yes	****	<0.0001
Fut2	Abs Prog vs. Goblet	-1.370	-1.987 to -0.7530	Yes	****	<0.0001
Fut2	Abs Prog vs. Endocrine	1.052	0.4136 to 1.690	Yes	****	<0.0001
Fut2	Abs Prog vs. Tuft	1.608	0.5123 to 2.703	Yes	****	<0.0001
Fut2	Pre-DCC vs. Pre-PCC	0.2977	-0.2792 to 0.8745	No	ns	0.9274
Fut2	Pre-DCC vs. Pro-DCC	0.4452	-0.06821 to 0.9585	No	ns	0.1810
Fut2	Pre-DCC vs. Mature DCC	1.833	1.433 to 2.232	Yes	****	<0.0001
Fut2	Pre-DCC vs. Mature PCC	1.987	1.570 to 2.404	Yes	****	<0.0001
Fut2	Pre-DCC vs. P-I CC	1.980	1.499 to 2.461	Yes	****	<0.0001
Fut2	Pre-DCC vs. Pro-Senescent CC	2.473	2.110 to 2.836	Yes	****	<0.0001
Fut2	Pre-DCC vs. Sec Prog	-1.375	-1.860 to -0.8901	Yes	****	<0.0001
Fut2	Pre-DCC vs. Paneth-like	-4.270	-4.766 to -3.775	Yes	****	<0.0001
Fut2	Pre-DCC vs. Goblet	-0.4345	-1.027 to 0.1581	No	ns	0.4618
Fut2	Pre-DCC vs. Endocrine	1.987	1.372 to 2.602	Yes	****	<0.0001
Fut2	Pre-DCC vs. Tuft	2.543	1.461 to 3.625	Yes	****	<0.0001
Fut2	Pre-PCC vs. Pro-DCC	0.1475	-0.5162 to 0.8111	No	ns	>0.9999
Fut2	Pre-PCC vs. Mature DCC	1.535	0.9551 to 2.115	Yes	****	<0.0001
Fut2	Pre-PCC vs. Mature PCC	1.689	1.097 to 2.281	Yes	****	<0.0001
Fut2	Pre-PCC vs. P-I CC	1.682	1.044 to 2.321	Yes	****	<0.0001
Fut2	Pre-PCC vs. Pro-Senescent CC	2.176	1.620 to 2.731	Yes	****	<0.0001
Fut2	Pre-PCC vs. Sec Prog	-1.673	-2.314 to -1.031	Yes	****	<0.0001
Fut2	Pre-PCC vs. Paneth-like	-4.568	-5.218 to -3.918	Yes	****	<0.0001
Fut2	Pre-PCC vs. Goblet	-0.7322	-1.459 to -0.005498	Yes	*	0.0460
Fut2	Pre-PCC vs. Endocrine	1.689	0.9447 to 2.434	Yes	****	<0.0001
Fut2	Pre-PCC vs. Tuft	2.246	1.085 to 3.407	Yes	****	<0.0001
Fut2	Pro-DCC vs. Mature DCC	1.387	0.8708 to 1.904	Yes	****	<0.0001
Fut2	Pro-DCC vs. Mature PCC	1.542	1.011 to 2.072	Yes	****	<0.0001
Fut2	Pro-DCC vs. P-I CC	1.535	0.9528 to 2.117	Yes	****	<0.0001
Fut2	Pro-DCC vs. Pro-Senescent CC	2.028	1.539 to 2.517	Yes	****	<0.0001
Fut2	Pro-DCC vs. Sec Prog	-1.820	-2.406 to -1.235	Yes	****	<0.0001
Fut2	Pro-DCC vs. Paneth-like	-4.715	-5.310 to -4.121	Yes	****	<0.0001
Fut2	Pro-DCC vs. Goblet	-0.8797	-1.557 to -0.2023	Yes	***	0.0010
Fut2	Pro-DCC vs. Endocrine	1.542	0.8452 to 2.239	Yes	****	<0.0001
Fut2	Pro-DCC vs. Tuft	2.098	0.9674 to 3.229	Yes	****	<0.0001
Fut2	Mature DCC vs. Mature PCC	0.1544	-0.2665 to 0.5753	No	ns	0.9972
Fut2	Mature DCC vs. P-I CC	0.1475	-0.3368 to 0.6318	No	ns	0.9997
Fut2	Mature DCC vs. Pro-Senescent CC	0.6408	0.2733 to 1.008	Yes	****	<0.0001
Fut2	Mature DCC vs. Sec Prog	-3.207	-3.696 to -2.719	Yes	****	<0.0001
Fut2	Mature DCC vs. Paneth-like	-6.103	-6.601 to -5.604	Yes	****	<0.0001
Fut2	Mature DCC vs. Goblet	-2.267	-2.862 to -1.672	Yes	****	<0.0001
Fut2	Mature DCC vs. Endocrine	0.1546	-0.4628 to 0.7721	No	ns	>0.9999
Fut2	Mature DCC vs. Tuft	0.7107	-0.3729 to 1.794	No	ns	0.6625
Fut2	Mature PCC vs. P-I CC	-0.006905	-0.5060 to 0.4922	No	ns	>0.9999
Fut2	Mature PCC vs. Pro-Senescent CC	0.4864	0.09958 to 0.8732	Yes	**	0.0018
Fut2	Mature PCC vs. Sec Prog	-3.362	-3.865 to -2.859	Yes	****	<0.0001
Fut2	Mature PCC vs. Paneth-like	-6.257	-6.770 to -5.744	Yes	****	<0.0001
Fut2	Mature PCC vs. Goblet	-2.421	-3.029 to -1.814	Yes	****	<0.0001
Fut2	Mature PCC vs. Endocrine	0.0002377	-0.6289 to 0.6293	No	ns	>0.9999
Fut2	Mature PCC vs. Tuft	0.5563	-0.5340 to 1.647	No	ns	0.9336

Fut2	P-I CC vs. Pro-Senescent CC	0.4933	0.03836 to 0.9482	Yes	*	0.0188
Fut2	P-I CC vs. Sec Prog	-3.355	-3.912 to -2.798	Yes	****	<0.0001
Fut2	P-I CC vs. Paneth-like	-6.250	-6.816 to -5.684	Yes	****	<0.0001
Fut2	P-I CC vs. Goblet	-2.415	-3.068 to -1.762	Yes	****	<0.0001
Fut2	P-I CC vs. Endocrine	0.007142	-0.6660 to 0.6803	No	ns	>0.9999
Fut2	P-I CC vs. Tuft	0.5632	-0.5531 to 1.680	No	ns	0.9393
Fut2	Pro-Senescent CC vs. Sec Prog	-3.848	-4.307 to -3.389	Yes	****	<0.0001
Fut2	Pro-Senescent CC vs. Paneth-like	-6.744	-7.214 to -6.274	Yes	****	<0.0001
Fut2	Pro-Senescent CC vs. Goblet	-2.908	-3.480 to -2.336	Yes	****	<0.0001
Fut2	Pro-Senescent CC vs. Endocrine	-0.4862	-1.081 to 0.1085	No	ns	0.2679
Fut2	Pro-Senescent CC vs. Tuft	0.06995	-1.001 to 1.141	No	ns	>0.9999
Fut2	Sec Prog vs. Paneth-like	-2.895	-3.465 to -2.326	Yes	****	<0.0001
Fut2	Sec Prog vs. Goblet	0.9404	0.2844 to 1.596	Yes	***	0.0001
Fut2	Sec Prog vs. Endocrine	3.362	2.686 to 4.038	Yes	****	<0.0001
Fut2	Sec Prog vs. Tuft	3.918	2.800 to 5.036	Yes	****	<0.0001
Fut2	Paneth-like vs. Goblet	3.836	3.172 to 4.500	Yes	****	<0.0001
Fut2	Paneth-like vs. Endocrine	6.258	5.574 to 6.941	Yes	****	<0.0001
Fut2	Paneth-like vs. Tuft	6.814	5.691 to 7.936	Yes	****	<0.0001
Fut2	Goblet vs. Endocrine	2.422	1.665 to 3.179	Yes	****	<0.0001
Fut2	Goblet vs. Tuft	2.978	1.809 to 4.147	Yes	****	<0.0001
Fut2	Endocrine vs. Tuft	0.5561	-0.6241 to 1.736	No	ns	0.9660

Supplementary Table 7. One-way ANOVA of IL-22–inducible genes from d9 *C.r*-infected T cell-specific IL-22 (*Il22*$^{\Delta Tcell}$) mice shown in Figure ED6f.

Gene	Tukey's multiple comparisons test	Mean Diff.	95.00% CI of diff.	Below threshold?	Summary	Adjusted P Value
S100a8	Lgr5-hi vs. Lgr5-dim	-0.1353	-0.7644 to 0.4938	No	ns	>0.9999
S100a8	Lgr5-hi vs. Early TA	-0.01625	-0.8198 to 0.7873	No	ns	>0.9999
S100a8	Lgr5-hi vs. Abs Prog	-0.1530	-0.7916 to 0.4856	No	ns	>0.9999
S100a8	Lgr5-hi vs. Pre-DCC	-0.04022	-0.6247 to 0.5443	No	ns	>0.9999
S100a8	Lgr5-hi vs. Pre-PCC	-0.2825	-1.177 to 0.6118	No	ns	0.9995
S100a8	Lgr5-hi vs. Pro-DCC	-1.083	-1.902 to -0.2645	Yes	***	0.0007
S100a8	Lgr5-hi vs. Mature DCC	-0.4832	-0.9954 to 0.02904	No	ns	0.0901
S100a8	Lgr5-hi vs. Mature PCC	-0.2674	-0.9308 to 0.3960	No	ns	0.9923
S100a8	Lgr5-hi vs. P-I CC	-1.018	-1.726 to -0.3098	Yes	****	<0.0001
S100a8	Lgr5-hi vs. Pro-Senescent CC	-0.09921	-0.8162 to 0.6178	No	ns	>0.9999
S100a8	Lgr5-hi vs. Sec Prog	0.002267	-0.7245 to 0.7290	No	ns	>0.9999
S100a8	Lgr5-hi vs. Paneth-like	-0.006242	-0.6916 to 0.6791	No	ns	>0.9999
S100a8	Lgr5-hi vs. Goblet	-0.07599	-0.8409 to 0.6889	No	ns	>0.9999
S100a8	Lgr5-hi vs. Endocrine	-1.005	-2.072 to 0.06284	No	ns	0.0921
S100a8	Lgr5-hi vs. Tuft	-0.001437	-1.368 to 1.365	No	ns	>0.9999
S100a8	Lgr5-dim vs. Early TA	0.1190	-0.7115 to 0.9496	No	ns	>0.9999
S100a8	Lgr5-dim vs. Abs Prog	-0.01771	-0.6901 to 0.6546	No	ns	>0.9999
S100a8	Lgr5-dim vs. Pre-DCC	0.09507	-0.5261 to 0.7162	No	ns	>0.9999
S100a8	Lgr5-dim vs. Pre-PCC	-0.1472	-1.066 to 0.7714	No	ns	>0.9999
S100a8	Lgr5-dim vs. Pro-DCC	-0.9478	-1.793 to -0.1027	Yes	*	0.0118
S100a8	Lgr5-dim vs. Mature DCC	-0.3479	-0.9016 to 0.2058	No	ns	0.7295
S100a8	Lgr5-dim vs. Mature PCC	-0.1321	-0.8280 to 0.5638	No	ns	>0.9999
S100a8	Lgr5-dim vs. P-I CC	-0.8824	-1.621 to -0.1440	Yes	**	0.0044
S100a8	Lgr5-dim vs. Pro-Senescent CC	0.03607	-0.7111 to 0.7833	No	ns	>0.9999
S100a8	Lgr5-dim vs. Sec Prog	0.1376	-0.6190 to 0.8941	No	ns	>0.9999
S100a8	Lgr5-dim vs. Paneth-like	0.1290	-0.5878 to 0.8459	No	ns	>0.9999
S100a8	Lgr5-dim vs. Goblet	0.05930	-0.7339 to 0.8525	No	ns	>0.9999
S100a8	Lgr5-dim vs. Endocrine	-0.8692	-1.957 to 0.2186	No	ns	0.3057
S100a8	Lgr5-dim vs. Tuft	0.1338	-1.249 to 1.516	No	ns	>0.9999
S100a8	Early TA vs. Abs Prog	-0.1367	-0.9745 to 0.7011	No	ns	>0.9999
S100a8	Early TA vs. Pre-DCC	-0.02397	-0.8213 to 0.7733	No	ns	>0.9999
S100a8	Early TA vs. Pre-PCC	-0.2662	-1.312 to 0.7796	No	ns	>0.9999
S100a8	Early TA vs. Pro-DCC	-1.067	-2.049 to -0.08497	Yes	*	0.0183
S100a8	Early TA vs. Mature DCC	-0.4669	-1.213 to 0.2790	No	ns	0.7350
S100a8	Early TA vs. Mature PCC	-0.2512	-1.108 to 0.6057	No	ns	0.9998
S100a8	Early TA vs. P-I CC	-1.001	-1.893 to -0.1097	Yes	*	0.0115
S100a8	Early TA vs. Pro-Senescent CC	-0.08296	-0.9819 to 0.8160	No	ns	>0.9999
S100a8	Early TA vs. Sec Prog	0.01852	-0.8883 to 0.9253	No	ns	>0.9999
S100a8	Early TA vs. Paneth-like	0.01001	-0.8639 to 0.8839	No	ns	>0.9999
S100a8	Early TA vs. Goblet	-0.05974	-0.9973 to 0.8779	No	ns	>0.9999
S100a8	Early TA vs. Endocrine	-0.9883	-2.186 to 0.2089	No	ns	0.2523
S100a8	Early TA vs. Tuft	0.01481	-1.455 to 1.485	No	ns	>0.9999
S100a8	Abs Prog vs. Pre-DCC	0.1128	-0.5180 to 0.7436	No	ns	>0.9999
S100a8	Abs Prog vs. Pre-PCC	-0.1295	-1.055 to 0.7957	No	ns	>0.9999
S100a8	Abs Prog vs. Pro-DCC	-0.9301	-1.782 to -0.07787	Yes	*	0.0172
S100a8	Abs Prog vs. Mature DCC	-0.3302	-0.8947 to 0.2343	No	ns	0.8227
S100a8	Abs Prog vs. Mature PCC	-0.1144	-0.8189 to 0.5901	No	ns	>0.9999
S100a8	Abs Prog vs. P-I CC	-0.8647	-1.611 to -0.1181	Yes	**	0.0073
S100a8	Abs Prog vs. Pro-Senescent CC	0.05378	-0.7014 to 0.8090	No	ns	>0.9999
S100a8	Abs Prog vs. Sec Prog	0.1553	-0.6092 to 0.9198	No	ns	>0.9999
S100a8	Abs Prog vs. Paneth-like	0.1468	-0.5785 to 0.8720	No	ns	>0.9999
S100a8	Abs Prog vs. Goblet	0.07701	-0.7238 to 0.8778	No	ns	>0.9999
S100a8	Abs Prog vs. Endocrine	-0.8515	-1.945 to 0.2419	No	ns	0.3506
S100a8	Abs Prog vs. Tuft	0.1516	-1.235 to 1.538	No	ns	>0.9999
S100a8	Pre-DCC vs. Pre-PCC	-0.2423	-1.131 to 0.6464	No	ns	>0.9999
S100a8	Pre-DCC vs. Pro-DCC	-1.043	-1.855 to -0.2304	Yes	**	0.0012
S100a8	Pre-DCC vs. Mature DCC	-0.4430	-0.9454 to 0.05944	No	ns	0.1598

S100a8	Pre-DCC vs. Mature PCC	-0.2272	-0.8830 to 0.4286	No	ns	0.9985
S100a8	Pre-DCC vs. P-I CC	-0.9775	-1.678 to -0.2767	Yes	***	0.0002
S100a8	Pre-DCC vs. Pro-Senescent CC	-0.05900	-0.7690 to 0.6510	No	ns	>0.9999
S100a8	Pre-DCC vs. Sec Prog	0.04248	-0.6774 to 0.7624	No	ns	>0.9999
S100a8	Pre-DCC vs. Paneth-like	0.03398	-0.6440 to 0.7120	No	ns	>0.9999
S100a8	Pre-DCC vs. Goblet	-0.03577	-0.7941 to 0.7226	No	ns	>0.9999
S100a8	Pre-DCC vs. Endocrine	-0.9643	-2.027 to 0.09838	No	ns	0.1269
S100a8	Pre-DCC vs. Tuft	0.03878	-1.324 to 1.402	No	ns	>0.9999
S100a8	Pre-PCC vs. Pro-DCC	-0.8006	-1.858 to 0.2568	No	ns	0.4018
S100a8	Pre-PCC vs. Mature DCC	-0.2007	-1.044 to 0.6422	No	ns	>0.9999
S100a8	Pre-PCC vs. Mature PCC	0.01508	-0.9274 to 0.9575	No	ns	>0.9999
S100a8	Pre-PCC vs. P-I CC	-0.7352	-1.709 to 0.2391	No	ns	0.4080
S100a8	Pre-PCC vs. Pro-Senescent CC	0.1833	-0.7976 to 1.164	No	ns	>0.9999
S100a8	Pre-PCC vs. Sec Prog	0.2848	-0.7033 to 1.273	No	ns	0.9998
S100a8	Pre-PCC vs. Paneth-like	0.2763	-0.6818 to 1.234	No	ns	0.9998
S100a8	Pre-PCC vs. Goblet	0.2065	-0.8099 to 1.223	No	ns	>0.9999
S100a8	Pre-PCC vs. Endocrine	-0.7220	-1.982 to 0.5379	No	ns	0.8447
S100a8	Pre-PCC vs. Tuft	0.2811	-1.241 to 1.803	No	ns	>0.9999
S100a8	Pro-DCC vs. Mature DCC	0.5999	-0.1622 to 1.362	No	ns	0.3316
S100a8	Pro-DCC vs. Mature PCC	0.8157	-0.05525 to 1.687	No	ns	0.0963
S100a8	Pro-DCC vs. P-I CC	0.06543	-0.8399 to 0.9707	No	ns	>0.9999
S100a8	Pro-DCC vs. Pro-Senescent CC	0.9839	0.07145 to 1.896	Yes	*	0.0202
S100a8	Pro-DCC vs. Sec Prog	1.085	0.1652 to 2.006	Yes	**	0.0054
S100a8	Pro-DCC vs. Paneth-like	1.077	0.1891 to 1.965	Yes	**	0.0034
S100a8	Pro-DCC vs. Goblet	1.007	0.05660 to 1.958	Yes	*	0.0254
S100a8	Pro-DCC vs. Endocrine	0.07858	-1.129 to 1.286	No	ns	>0.9999
S100a8	Pro-DCC vs. Tuft	1.082	-0.3967 to 2.560	No	ns	0.4656
S100a8	Mature DCC vs. Mature PCC	0.2158	-0.3766 to 0.8081	No	ns	0.9974
S100a8	Mature DCC vs. P-I CC	-0.5345	-1.176 to 0.1073	No	ns	0.2386
S100a8	Mature DCC vs. Pro-Senescent CC	0.3840	-0.2679 to 1.036	No	ns	0.8146
S100a8	Mature DCC vs. Sec Prog	0.4855	-0.1771 to 1.148	No	ns	0.4629
S100a8	Mature DCC vs. Paneth-like	0.4770	-0.1399 to 1.094	No	ns	0.3634
S100a8	Mature DCC vs. Goblet	0.4072	-0.2969 to 1.111	No	ns	0.8352
S100a8	Mature DCC vs. Endocrine	-0.5213	-1.546 to 0.5034	No	ns	0.9350
S100a8	Mature DCC vs. Tuft	0.4818	-0.8517 to 1.815	No	ns	0.9976
S100a8	Mature PCC vs. P-I CC	-0.7503	-1.518 to 0.01757	No	ns	0.0639
S100a8	Mature PCC vs. Pro-Senescent CC	0.1682	-0.6081 to 0.9445	No	ns	>0.9999
S100a8	Mature PCC vs. Sec Prog	0.2697	-0.5156 to 1.055	No	ns	0.9987
S100a8	Mature PCC vs. Paneth-like	0.2612	-0.4859 to 1.008	No	ns	0.9984
S100a8	Mature PCC vs. Goblet	0.1914	-0.6293 to 1.012	No	ns	>0.9999
S100a8	Mature PCC vs. Endocrine	-0.7371	-1.845 to 0.3709	No	ns	0.6385
S100a8	Mature PCC vs. Tuft	0.2660	-1.133 to 1.664	No	ns	>0.9999
S100a8	P-I CC vs. Pro-Senescent CC	0.9185	0.1039 to 1.733	Yes	*	0.0109
S100a8	P-I CC vs. Sec Prog	1.020	0.1967 to 1.843	Yes	**	0.0023
S100a8	P-I CC vs. Paneth-like	1.011	0.2246 to 1.798	Yes	**	0.0012
S100a8	P-I CC vs. Goblet	0.9417	0.08465 to 1.799	Yes	*	0.0157
S100a8	P-I CC vs. Endocrine	0.01315	-1.122 to 1.148	No	ns	>0.9999
S100a8	P-I CC vs. Tuft	1.016	-0.4039 to 2.436	No	ns	0.5071
S100a8	Pro-Senescent CC vs. Sec Prog	0.1015	-0.7296 to 0.9326	No	ns	>0.9999
S100a8	Pro-Senescent CC vs. Paneth-like	0.09297	-0.7021 to 0.8881	No	ns	>0.9999
S100a8	Pro-Senescent CC vs. Goblet	0.02323	-0.8414 to 0.8878	No	ns	>0.9999
S100a8	Pro-Senescent CC vs. Endocrine	-0.9053	-2.046 to 0.2356	No	ns	0.3177
S100a8	Pro-Senescent CC vs. Tuft	0.09778	-1.327 to 1.522	No	ns	>0.9999
S100a8	Sec Prog vs. Paneth-like	-0.008509	-0.8124 to 0.7954	No	ns	>0.9999
S100a8	Sec Prog vs. Goblet	-0.07826	-0.9510 to 0.7944	No	ns	>0.9999
S100a8	Sec Prog vs. Endocrine	-1.007	-2.154 to 0.1403	No	ns	0.1654

S100a8	Sec Prog vs. Tuft	-0.003704	-1.433 to 1.426	No	ns	>0.9999
S100a8	Paneth-like vs. Goblet	-0.06975	-0.9083 to 0.7688	No	ns	>0.9999
S100a8	Paneth-like vs. Endocrine	-0.9983	-2.120 to 0.1230	No	ns	0.1482
S100a8	Paneth-like vs. Tuft	0.004805	-1.404 to 1.414	No	ns	>0.9999
S100a8	Goblet vs. Endocrine	-0.9285	-2.100 to 0.2431	No	ns	0.3198
S100a8	Goblet vs. Tuft	0.07455	-1.375 to 1.524	No	ns	>0.9999
S100a8	Endocrine vs. Tuft	1.003	-0.6263 to 2.632	No	ns	0.7587

Gene	Tukey's multiple comparisons test	Mean Diff.	95.00% CI of diff.	Below threshold?	Summary	Adjusted P Value
Cxcl2	Lgr5-hi vs. Lgr5-dim	-0.6617	-1.996 to 0.6723	No	ns	0.9471
Cxcl2	Lgr5-hi vs. Early TA	-1.394	-3.098 to 0.3102	No	ns	0.2669
Cxcl2	Lgr5-hi vs. Abs Prog	-0.5821	-1.939 to 0.7745	No	ns	0.9857
Cxcl2	Lgr5-hi vs. Pre-DCC	-0.2568	-1.496 to 0.9827	No	ns	>0.9999
Cxcl2	Lgr5-hi vs. Pre-PCC	0.3411	-1.555 to 2.237	No	ns	>0.9999
Cxcl2	Lgr5-hi vs. Pro-DCC	-0.2956	-2.023 to 1.432	No	ns	>0.9999
Cxcl2	Lgr5-hi vs. Mature DCC	0.6906	-0.3956 to 1.777	No	ns	0.7117
Cxcl2	Lgr5-hi vs. Mature PCC	1.012	-0.3949 to 2.419	No	ns	0.4975
Cxcl2	Lgr5-hi vs. P-I CC	0.2108	-1.290 to 1.712	No	ns	>0.9999
Cxcl2	Lgr5-hi vs. Pro-Senescent CC	1.128	-0.3923 to 2.648	No	ns	0.4394
Cxcl2	Lgr5-hi vs. Sec Prog	-0.3134	-1.855 to 1.228	No	ns	>0.9999
Cxcl2	Lgr5-hi vs. Paneth-like	1.197	-0.2560 to 2.651	No	ns	0.2554
Cxcl2	Lgr5-hi vs. Goblet	0.9925	-0.6295 to 2.614	No	ns	0.7669
Cxcl2	Lgr5-hi vs. Endocrine	0.7574	-1.506 to 3.021	No	ns	0.9990
Cxcl2	Lgr5-hi vs. Tuft	1.390	-1.507 to 4.288	No	ns	0.9601
Cxcl2	Lgr5-dim vs. Early TA	-0.7319	-2.493 to 1.029	No	ns	0.9895
Cxcl2	Lgr5-dim vs. Abs Prog	0.07963	-1.348 to 1.508	No	ns	>0.9999
Cxcl2	Lgr5-dim vs. Pre-DCC	0.4050	-0.9122 to 1.722	No	ns	0.9996
Cxcl2	Lgr5-dim vs. Pre-PCC	1.003	-0.9452 to 2.951	No	ns	0.9286
Cxcl2	Lgr5-dim vs. Pro-DCC	0.3661	-1.418 to 2.150	No	ns	>0.9999
Cxcl2	Lgr5-dim vs. Mature DCC	1.352	0.1782 to 2.527	Yes	**	0.0079
Cxcl2	Lgr5-dim vs. Mature PCC	1.674	0.1979 to 3.149	Yes	**	0.0100
Cxcl2	Lgr5-dim vs. P-I CC	0.8725	-0.6933 to 2.438	No	ns	0.8716
Cxcl2	Lgr5-dim vs. Pro-Senescent CC	1.790	0.2054 to 3.374	Yes	*	0.0106
Cxcl2	Lgr5-dim vs. Sec Prog	0.3483	-1.256 to 1.953	No	ns	>0.9999
Cxcl2	Lgr5-dim vs. Paneth-like	1.859	0.3389 to 3.379	Yes	**	0.0030
Cxcl2	Lgr5-dim vs. Goblet	1.654	-0.02790 to 3.336	No	ns	0.0598
Cxcl2	Lgr5-dim vs. Endocrine	1.419	-0.8878 to 3.726	No	ns	0.7597
Cxcl2	Lgr5-dim vs. Tuft	2.052	-0.8797 to 4.984	No	ns	0.5481
Cxcl2	Early TA vs. Abs Prog	0.8116	-0.9668 to 2.590	No	ns	0.9744
Cxcl2	Early TA vs. Pre-DCC	1.137	-0.5538 to 2.828	No	ns	0.6199
Cxcl2	Early TA vs. Pre-PCC	1.735	-0.4829 to 3.952	No	ns	0.3427
Cxcl2	Early TA vs. Pro-DCC	1.098	-0.9772 to 3.173	No	ns	0.9118
Cxcl2	Early TA vs. Mature DCC	2.084	0.5025 to 3.666	Yes	***	0.0007
Cxcl2	Early TA vs. Mature PCC	2.405	0.5886 to 4.222	Yes	***	0.0006
Cxcl2	Early TA vs. P-I CC	1.604	-0.2864 to 3.495	No	ns	0.2108
Cxcl2	Early TA vs. Pro-Senescent CC	2.522	0.6154 to 4.428	Yes	***	0.0007
Cxcl2	Early TA vs. Sec Prog	1.080	-0.8426 to 3.003	No	ns	0.8642
Cxcl2	Early TA vs. Paneth-like	2.591	0.7377 to 4.444	Yes	***	0.0002
Cxcl2	Early TA vs. Goblet	2.386	0.3979 to 4.374	Yes	**	0.0041
Cxcl2	Early TA vs. Endocrine	2.151	-0.3877 to 4.690	No	ns	0.2129
Cxcl2	Early TA vs. Tuft	2.784	-0.3335 to 5.901	No	ns	0.1446
Cxcl2	Abs Prog vs. Pre-DCC	0.3253	-1.015 to 1.665	No	ns	>0.9999
Cxcl2	Abs Prog vs. Pre-PCC	0.9232	-1.040 to 2.887	No	ns	0.9665
Cxcl2	Abs Prog vs. Pro-DCC	0.2865	-1.515 to 2.088	No	ns	>0.9999
Cxcl2	Abs Prog vs. Mature DCC	1.273	0.07303 to 2.472	Yes	*	0.0250
Cxcl2	Abs Prog vs. Mature PCC	1.594	0.09781 to 3.090	Yes	*	0.0237
Cxcl2	Abs Prog vs. P-I CC	0.7929	-0.7922 to 2.378	No	ns	0.9433
Cxcl2	Abs Prog vs. Pro-Senescent CC	1.710	0.1067 to 3.314	Yes	*	0.0233
Cxcl2	Abs Prog vs. Sec Prog	0.2687	-1.354 to 1.892	No	ns	>0.9999
Cxcl2	Abs Prog vs. Paneth-like	1.779	0.2394 to 3.319	Yes	**	0.0075
Cxcl2	Abs Prog vs. Goblet	1.575	-0.1255 to 3.275	No	ns	0.1064
Cxcl2	Abs Prog vs. Endocrine	1.339	-0.9805 to 3.659	No	ns	0.8370

Cxcl2	Abs Prog vs. Tuft	1.972	-0.9697 to 4.914	No	ns	0.6252
Cxcl2	Pre-DCC vs. Pre-PCC	0.5979	-1.287 to 2.482	No	ns	0.9995
Cxcl2	Pre-DCC vs. Pro-DCC	-0.03884	-1.753 to 1.676	No	ns	>0.9999
Cxcl2	Pre-DCC vs. Mature DCC	0.9474	-0.1180 to 2.013	No	ns	0.1495
Cxcl2	Pre-DCC vs. Mature PCC	1.269	-0.1221 to 2.659	No	ns	0.1215
Cxcl2	Pre-DCC vs. P-I CC	0.4676	-1.018 to 1.954	No	ns	0.9995
Cxcl2	Pre-DCC vs. Pro-Senescent CC	1.385	-0.1208 to 2.890	No	ns	0.1131
Cxcl2	Pre-DCC vs. Sec Prog	-0.05664	-1.583 to 1.470	No	ns	>0.9999
Cxcl2	Pre-DCC vs. Paneth-like	1.454	0.01624 to 2.892	Yes	*	0.0442
Cxcl2	Pre-DCC vs. Goblet	1.249	-0.3588 to 2.857	No	ns	0.3550
Cxcl2	Pre-DCC vs. Endocrine	1.014	-1.239 to 3.268	No	ns	0.9775
Cxcl2	Pre-DCC vs. Tuft	1.647	-1.243 to 4.537	No	ns	0.8503
Cxcl2	Pre-PCC vs. Pro-DCC	-0.6367	-2.873 to 1.599	No	ns	0.9999
Cxcl2	Pre-PCC vs. Mature DCC	0.3495	-1.438 to 2.137	No	ns	>0.9999
Cxcl2	Pre-PCC vs. Mature PCC	0.6707	-1.328 to 2.669	No	ns	0.9990
Cxcl2	Pre-PCC vs. P-I CC	-0.1303	-2.196 to 1.936	No	ns	>0.9999
Cxcl2	Pre-PCC vs. Pro-Senescent CC	0.7870	-1.293 to 2.867	No	ns	0.9961
Cxcl2	Pre-PCC vs. Sec Prog	-0.6545	-2.750 to 1.441	No	ns	0.9996
Cxcl2	Pre-PCC vs. Paneth-like	0.8561	-1.175 to 2.888	No	ns	0.9880
Cxcl2	Pre-PCC vs. Goblet	0.6513	-1.504 to 2.807	No	ns	0.9997
Cxcl2	Pre-PCC vs. Endocrine	0.4163	-2.255 to 3.088	No	ns	>0.9999
Cxcl2	Pre-PCC vs. Tuft	1.049	-2.178 to 4.276	No	ns	0.9993
Cxcl2	Pro-DCC vs. Mature DCC	0.9862	-0.6211 to 2.594	No	ns	0.7632
Cxcl2	Pro-DCC vs. Mature PCC	1.307	-0.5317 to 3.147	No	ns	0.5194
Cxcl2	Pro-DCC vs. P-I CC	0.5064	-1.406 to 2.419	No	ns	>0.9999
Cxcl2	Pro-DCC vs. Pro-Senescent CC	1.424	-0.5038 to 3.351	No	ns	0.4479
Cxcl2	Pro-DCC vs. Sec Prog	-0.01781	-1.962 to 1.926	No	ns	>0.9999
Cxcl2	Pro-DCC vs. Paneth-like	1.493	-0.3821 to 3.368	No	ns	0.3118
Cxcl2	Pro-DCC vs. Goblet	1.288	-0.7205 to 3.297	No	ns	0.6985
Cxcl2	Pro-DCC vs. Endocrine	1.053	-1.502 to 3.608	No	ns	0.9904
Cxcl2	Pro-DCC vs. Tuft	1.686	-1.445 to 4.816	No	ns	0.8994
Cxcl2	Mature DCC vs. Mature PCC	0.3212	-0.9349 to 1.577	No	ns	>0.9999
Cxcl2	Mature DCC vs. P-I CC	-0.4798	-1.841 to 0.8811	No	ns	0.9982
Cxcl2	Mature DCC vs. Pro-Senescent CC	0.4374	-0.9448 to 1.820	No	ns	0.9995
Cxcl2	Mature DCC vs. Sec Prog	-1.004	-2.409 to 0.4009	No	ns	0.5096
Cxcl2	Mature DCC vs. Paneth-like	0.5066	-0.8014 to 1.815	No	ns	0.9949
Cxcl2	Mature DCC vs. Goblet	0.3018	-1.191 to 1.795	No	ns	>0.9999
Cxcl2	Mature DCC vs. Endocrine	0.06675	-2.106 to 2.240	No	ns	>0.9999
Cxcl2	Mature DCC vs. Tuft	0.6997	-2.128 to 3.527	No	ns	>0.9999
Cxcl2	Mature PCC vs. P-I CC	-0.8010	-2.429 to 0.8272	No	ns	0.9506
Cxcl2	Mature PCC vs. Pro-Senescent CC	0.1163	-1.530 to 1.762	No	ns	>0.9999
Cxcl2	Mature PCC vs. Sec Prog	-1.325	-2.990 to 0.3400	No	ns	0.3126
Cxcl2	Mature PCC vs. Paneth-like	0.1854	-1.399 to 1.770	No	ns	>0.9999
Cxcl2	Mature PCC vs. Goblet	-0.01938	-1.760 to 1.721	No	ns	>0.9999
Cxcl2	Mature PCC vs. Endocrine	-0.2544	-2.604 to 2.095	No	ns	>0.9999
Cxcl2	Mature PCC vs. Tuft	0.3785	-2.587 to 3.344	No	ns	>0.9999
Cxcl2	P-I CC vs. Pro-Senescent CC	0.9173	-0.8101 to 2.645	No	ns	0.9094
Cxcl2	P-I CC vs. Sec Prog	-0.5242	-2.270 to 1.221	No	ns	0.9997
Cxcl2	P-I CC vs. Paneth-like	0.9864	-0.6822 to 2.655	No	ns	0.8105
Cxcl2	P-I CC vs. Goblet	0.7816	-1.036 to 2.599	No	ns	0.9853
Cxcl2	P-I CC vs. Endocrine	0.5466	-1.861 to 2.954	No	ns	>0.9999
Cxcl2	P-I CC vs. Tuft	1.179	-1.832 to 4.191	No	ns	0.9943
Cxcl2	Pro-Senescent CC vs. Sec Prog	-1.441	-3.204 to 0.3208	No	ns	0.2669
Cxcl2	Pro-Senescent CC vs. Paneth-like	0.06917	-1.617 to 1.755	No	ns	>0.9999
Cxcl2	Pro-Senescent CC vs. Goblet	-0.1356	-1.969 to 1.698	No	ns	>0.9999
Cxcl2	Pro-Senescent CC vs. Endocrine	-0.3707	-2.790 to 2.049	No	ns	>0.9999

Cxcl2	Pro-Senescent CC vs. Tuft	0.2622	-2.759 to 3.283	No	ns	>0.9999
Cxcl2	Sec Prog vs. Paneth-like	1.511	-0.1941 to 3.215	No	ns	0.1537
Cxcl2	Sec Prog vs. Goblet	1.306	-0.5448 to 3.156	No	ns	0.5331
Cxcl2	Sec Prog vs. Endocrine	1.071	-1.362 to 3.503	No	ns	0.9817
Cxcl2	Sec Prog vs. Tuft	1.704	-1.328 to 4.735	No	ns	0.8639
Cxcl2	Paneth-like vs. Goblet	-0.2048	-1.983 to 1.573	No	ns	>0.9999
Cxcl2	Paneth-like vs. Endocrine	-0.4399	-2.818 to 1.938	No	ns	>0.9999
Cxcl2	Paneth-like vs. Tuft	0.1931	-2.795 to 3.181	No	ns	>0.9999
Cxcl2	Goblet vs. Endocrine	-0.2351	-2.720 to 2.249	No	ns	>0.9999
Cxcl2	Goblet vs. Tuft	0.3978	-2.676 to 3.471	No	ns	>0.9999
Cxcl2	Endocrine vs. Tuft	0.6329	-2.822 to 4.088	No	ns	>0.9999

Gene	Tukey's multiple comparisons test	Mean Diff.	95.00% CI of diff.	Below threshold?	Summary	Adjusted P Value
Cxcl5	Lgr5-hi vs. Lgr5-dim	-1.367	-2.130 to -0.6050	Yes	****	<0.0001
Cxcl5	Lgr5-hi vs. Early TA	-0.9252	-1.899 to 0.04869	No	ns	0.0843
Cxcl5	Lgr5-hi vs. Abs Prog	-0.9158	-1.690 to -0.1417	Yes	**	0.0052
Cxcl5	Lgr5-hi vs. Pre-DCC	-0.3202	-1.029 to 0.3882	No	ns	0.9765
Cxcl5	Lgr5-hi vs. Pre-PCC	0.2399	-0.8440 to 1.324	No	ns	>0.9999
Cxcl5	Lgr5-hi vs. Pro-DCC	-1.241	-2.230 to -0.2509	Yes	**	0.0019
Cxcl5	Lgr5-hi vs. Mature DCC	0.08817	-0.5327 to 0.7090	No	ns	>0.9999
Cxcl5	Lgr5-hi vs. Mature PCC	0.2492	-0.5548 to 1.053	No	ns	0.9996
Cxcl5	Lgr5-hi vs. P-I CC	-0.4271	-1.285 to 0.4309	No	ns	0.9455
Cxcl5	Lgr5-hi vs. Pro-Senescent CC	0.2353	-0.6337 to 1.104	No	ns	>0.9999
Cxcl5	Lgr5-hi vs. Sec Prog	-0.2184	-1.099 to 0.6625	No	ns	>0.9999
Cxcl5	Lgr5-hi vs. Paneth-like	0.2576	-0.5730 to 1.088	No	ns	0.9996
Cxcl5	Lgr5-hi vs. Goblet	0.2013	-0.7258 to 1.128	No	ns	>0.9999
Cxcl5	Lgr5-hi vs. Endocrine	0.3211	-0.9726 to 1.615	No	ns	>0.9999
Cxcl5	Lgr5-hi vs. Tuft	0.3464	-1.310 to 2.003	No	ns	>0.9999
Cxcl5	Lgr5-dim vs. Early TA	0.4423	-0.5644 to 1.449	No	ns	0.9820
Cxcl5	Lgr5-dim vs. Abs Prog	0.4517	-0.3632 to 1.267	No	ns	0.8763
Cxcl5	Lgr5-dim vs. Pre-DCC	1.047	0.2944 to 1.800	Yes	***	0.0002
Cxcl5	Lgr5-dim vs. Pre-PCC	1.607	0.4939 to 2.721	Yes	****	<0.0001
Cxcl5	Lgr5-dim vs. Pro-DCC	0.1268	-0.8953 to 1.149	No	ns	>0.9999
Cxcl5	Lgr5-dim vs. Mature DCC	1.456	0.7845 to 2.127	Yes	****	<0.0001
Cxcl5	Lgr5-dim vs. Mature PCC	1.617	0.7732 to 2.460	Yes	****	<0.0001
Cxcl5	Lgr5-dim vs. P-I CC	0.9404	0.04538 to 1.835	Yes	*	0.0282
Cxcl5	Lgr5-dim vs. Pro-Senescent CC	1.603	0.6972 to 2.508	Yes	****	<0.0001
Cxcl5	Lgr5-dim vs. Sec Prog	1.149	0.2321 to 2.066	Yes	**	0.0019
Cxcl5	Lgr5-dim vs. Paneth-like	1.625	0.7562 to 2.494	Yes	****	<0.0001
Cxcl5	Lgr5-dim vs. Goblet	1.569	0.6073 to 2.530	Yes	****	<0.0001
Cxcl5	Lgr5-dim vs. Endocrine	1.689	0.3700 to 3.007	Yes	**	0.0013
Cxcl5	Lgr5-dim vs. Tuft	1.714	0.03819 to 3.390	Yes	*	0.0388
Cxcl5	Early TA vs. Abs Prog	0.009410	-1.006 to 1.025	No	ns	>0.9999
Cxcl5	Early TA vs. Pre-DCC	0.6049	-0.3614 to 1.571	No	ns	0.7349
Cxcl5	Early TA vs. Pre-PCC	1.165	-0.1025 to 2.433	No	ns	0.1138
Cxcl5	Early TA vs. Pro-DCC	-0.3155	-1.504 to 0.8726	No	ns	>0.9999
Cxcl5	Early TA vs. Mature DCC	1.013	0.1092 to 1.917	Yes	*	0.0119
Cxcl5	Early TA vs. Mature PCC	1.174	0.1359 to 2.213	Yes	*	0.0104
Cxcl5	Early TA vs. P-I CC	0.4981	-0.5827 to 1.579	No	ns	0.9720
Cxcl5	Early TA vs. Pro-Senescent CC	1.160	0.07092 to 2.250	Yes	*	0.0237
Cxcl5	Early TA vs. Sec Prog	0.7068	-0.3922 to 1.806	No	ns	0.6941
Cxcl5	Early TA vs. Paneth-like	1.183	0.1236 to 2.242	Yes	*	0.0125
Cxcl5	Early TA vs. Goblet	1.126	-0.009937 to 2.263	No	ns	0.0550
Cxcl5	Early TA vs. Endocrine	1.246	-0.2048 to 2.697	No	ns	0.1938
Cxcl5	Early TA vs. Tuft	1.272	-0.5102 to 3.053	No	ns	0.5122
Cxcl5	Abs Prog vs. Pre-DCC	0.5955	-0.1690 to 1.360	No	ns	0.3502
Cxcl5	Abs Prog vs. Pre-PCC	1.156	0.03430 to 2.277	Yes	*	0.0355
Cxcl5	Abs Prog vs. Pro-DCC	-0.3249	-1.356 to 0.7058	No	ns	0.9995
Cxcl5	Abs Prog vs. Mature DCC	1.004	0.3198 to 1.688	Yes	****	<0.0001
Cxcl5	Abs Prog vs. Mature PCC	1.165	0.3111 to 2.019	Yes	***	0.0003
Cxcl5	Abs Prog vs. P-I CC	0.4887	-0.4161 to 1.394	No	ns	0.8973
Cxcl5	Abs Prog vs. Pro-Senescent CC	1.151	0.2357 to 2.066	Yes	**	0.0018

Cxcl5	Abs Prog vs. Sec Prog	0.6974	-0.2292 to 1.624	No	ns	0.4128
Cxcl5	Abs Prog vs. Paneth-like	1.173	0.2944 to 2.052	Yes	***	0.0005
Cxcl5	Abs Prog vs. Goblet	1.117	0.1464 to 2.088	Yes	**	0.0080
Cxcl5	Abs Prog vs. Endocrine	1.237	-0.08836 to 2.562	No	ns	0.0994
Cxcl5	Abs Prog vs. Tuft	1.262	-0.4188 to 2.943	No	ns	0.4172
Cxcl5	Pre-DCC vs. Pre-PCC	0.5601	-0.5170 to 1.637	No	ns	0.9227
Cxcl5	Pre-DCC vs. Pro-DCC	-0.9204	-1.903 to 0.06191	No	ns	0.0959
Cxcl5	Pre-DCC vs. Mature DCC	0.4084	-0.2005 to 1.017	No	ns	0.6244
Cxcl5	Pre-DCC vs. Mature PCC	0.5694	-0.2254 to 1.364	No	ns	0.5051
Cxcl5	Pre-DCC vs. P-I CC	-0.1068	-0.9562 to 0.7425	No	ns	>0.9999
Cxcl5	Pre-DCC vs. Pro-Senescent CC	0.5556	-0.3050 to 1.416	No	ns	0.6880
Cxcl5	Pre-DCC vs. Sec Prog	0.1019	-0.7707 to 0.9744	No	ns	>0.9999
Cxcl5	Pre-DCC vs. Paneth-like	0.5779	-0.2439 to 1.400	No	ns	0.5396
Cxcl5	Pre-DCC vs. Goblet	0.5215	-0.3976 to 1.441	No	ns	0.8547
Cxcl5	Pre-DCC vs. Endocrine	0.6414	-0.6467 to 1.929	No	ns	0.9454
Cxcl5	Pre-DCC vs. Tuft	0.6667	-0.9851 to 2.318	No	ns	0.9922
Cxcl5	Pre-PCC vs. Pro-DCC	-1.481	-2.760 to -0.2007	Yes	**	0.0074
Cxcl5	Pre-PCC vs. Mature DCC	-0.1517	-1.173 to 0.8699	No	ns	>0.9999
Cxcl5	Pre-PCC vs. Mature PCC	0.009335	-1.133 to 1.152	No	ns	>0.9999
Cxcl5	Pre-PCC vs. P-I CC	-0.6669	-1.848 to 0.5139	No	ns	0.8591
Cxcl5	Pre-PCC vs. Pro-Senescent CC	-0.004554	-1.193 to 1.184	No	ns	>0.9999
Cxcl5	Pre-PCC vs. Sec Prog	-0.4583	-1.656 to 0.7393	No	ns	0.9955
Cxcl5	Pre-PCC vs. Paneth-like	0.01775	-1.143 to 1.179	No	ns	>0.9999
Cxcl5	Pre-PCC vs. Goblet	-0.03860	-1.271 to 1.193	No	ns	>0.9999
Cxcl5	Pre-PCC vs. Endocrine	0.08124	-1.446 to 1.608	No	ns	>0.9999
Cxcl5	Pre-PCC vs. Tuft	0.1066	-1.738 to 1.951	No	ns	>0.9999
Cxcl5	Pro-DCC vs. Mature DCC	1.329	0.4077 to 2.250	Yes	****	<0.0001
Cxcl5	Pro-DCC vs. Mature PCC	1.490	0.4365 to 2.543	Yes	***	0.0001
Cxcl5	Pro-DCC vs. P-I CC	0.8136	-0.2815 to 1.909	No	ns	0.4369
Cxcl5	Pro-DCC vs. Pro-Senescent CC	1.476	0.3722 to 2.580	Yes	***	0.0005
Cxcl5	Pro-DCC vs. Sec Prog	1.022	-0.09084 to 2.135	No	ns	0.1146
Cxcl5	Pro-DCC vs. Paneth-like	1.498	0.4245 to 2.572	Yes	***	0.0002
Cxcl5	Pro-DCC vs. Goblet	1.442	0.2919 to 2.592	Yes	**	0.0019
Cxcl5	Pro-DCC vs. Endocrine	1.562	0.1000 to 3.024	Yes	*	0.0228
Cxcl5	Pro-DCC vs. Tuft	1.587	-0.2035 to 3.378	No	ns	0.1534
Cxcl5	Mature DCC vs. Mature PCC	0.1610	-0.5569 to 0.8790	No	ns	>0.9999
Cxcl5	Mature DCC vs. P-I CC	-0.5153	-1.293 to 0.2626	No	ns	0.6457
Cxcl5	Mature DCC vs. Pro-Senescent CC	0.1471	-0.6429 to 0.9372	No	ns	>0.9999
Cxcl5	Mature DCC vs. Sec Prog	-0.3066	-1.110 to 0.4965	No	ns	0.9957
Cxcl5	Mature DCC vs. Paneth-like	0.1694	-0.5782 to 0.9171	No	ns	>0.9999
Cxcl5	Mature DCC vs. Goblet	0.1131	-0.7404 to 0.9665	No	ns	>0.9999
Cxcl5	Mature DCC vs. Endocrine	0.2329	-1.009 to 1.475	No	ns	>0.9999
Cxcl5	Mature DCC vs. Tuft	0.2582	-1.358 to 1.874	No	ns	>0.9999
Cxcl5	Mature PCC vs. P-I CC	-0.6763	-1.607 to 0.2544	No	ns	0.4784
Cxcl5	Mature PCC vs. Pro-Senescent CC	-0.01389	-0.9547 to 0.9270	No	ns	>0.9999
Cxcl5	Mature PCC vs. Sec Prog	-0.4676	-1.419 to 0.4842	No	ns	0.9512
Cxcl5	Mature PCC vs. Paneth-like	0.008419	-0.8971 to 0.9139	No	ns	>0.9999
Cxcl5	Mature PCC vs. Goblet	-0.04794	-1.043 to 0.9467	No	ns	>0.9999
Cxcl5	Mature PCC vs. Endocrine	0.07191	-1.271 to 1.415	No	ns	>0.9999
Cxcl5	Mature PCC vs. Tuft	0.09722	-1.598 to 1.792	No	ns	>0.9999
Cxcl5	P-I CC vs. Pro-Senescent CC	0.6624	-0.3249 to 1.650	No	ns	0.6239
Cxcl5	P-I CC vs. Sec Prog	0.2087	-0.7891 to 1.206	No	ns	>0.9999
Cxcl5	P-I CC vs. Paneth-like	0.6847	-0.2690 to 1.638	No	ns	0.5010
Cxcl5	P-I CC vs. Goblet	0.6283	-0.4104 to 1.667	No	ns	0.7821
Cxcl5	P-I CC vs. Endocrine	0.7482	-0.6277 to 2.124	No	ns	0.8921
Cxcl5	P-I CC vs. Tuft	0.7735	-0.9477 to 2.495	No	ns	0.9778
Cxcl5	Pro-Senescent CC vs. Sec Prog	-0.4537	-1.461 to 0.5536	No	ns	0.9773
Cxcl5	Pro-Senescent CC vs. Paneth-like	0.02231	-0.9414 to 0.9860	No	ns	>0.9999

Cxcl5	Pro-Senescent CC vs. Goblet	-0.03405	-1.082 to 1.014	No	ns	>0.9999
Cxcl5	Pro-Senescent CC vs. Endocrine	0.08579	-1.297 to 1.469	No	ns	>0.9999
Cxcl5	Pro-Senescent CC vs. Tuft	0.1111	-1.616 to 1.838	No	ns	>0.9999
Cxcl5	Sec Prog vs. Paneth-like	0.4760	-0.4983 to 1.450	No	ns	0.9534
Cxcl5	Sec Prog vs. Goblet	0.4197	-0.6381 to 1.477	No	ns	0.9935
Cxcl5	Sec Prog vs. Endocrine	0.5395	-0.8508 to 1.930	No	ns	0.9948
Cxcl5	Sec Prog vs. Tuft	0.5648	-1.168 to 2.298	No	ns	0.9993
Cxcl5	Paneth-like vs. Goblet	-0.05636	-1.073 to 0.9599	No	ns	>0.9999
Cxcl5	Paneth-like vs. Endocrine	0.06349	-1.296 to 1.423	No	ns	>0.9999
Cxcl5	Paneth-like vs. Tuft	0.08880	-1.619 to 1.797	No	ns	>0.9999
Cxcl5	Goblet vs. Endocrine	0.1198	-1.300 to 1.540	No	ns	>0.9999
Cxcl5	Goblet vs. Tuft	0.1452	-1.612 to 1.902	No	ns	>0.9999
Cxcl5	Endocrine vs. Tuft	0.02532	-1.950 to 2.000	No	ns	>0.9999

Gene	Tukey's multiple comparisons test	Mean Diff.	95.00% CI of diff.	Below threshold?	Summary	Adjusted P Value
Lrg1	Lgr5-hi vs. Lgr5-dim	-1.570	-2.719 to -0.4201	Yes	***	0.0003
Lrg1	Lgr5-hi vs. Early TA	-0.6504	-2.119 to 0.8179	No	ns	0.9806
Lrg1	Lgr5-hi vs. Abs Prog	-4.688	-5.855 to -3.521	Yes	****	<0.0001
Lrg1	Lgr5-hi vs. Pre-DCC	-3.942	-5.010 to -2.874	Yes	****	<0.0001
Lrg1	Lgr5-hi vs. Pre-PCC	0.2838	-1.350 to 1.918	No	ns	>0.9999
Lrg1	Lgr5-hi vs. Pro-DCC	-0.3280	-1.817 to 1.161	No	ns	>0.9999
Lrg1	Lgr5-hi vs. Mature DCC	1.275	0.3392 to 2.211	Yes	***	0.0003
Lrg1	Lgr5-hi vs. Mature PCC	1.269	0.05673 to 2.481	Yes	*	0.0295
Lrg1	Lgr5-hi vs. P-I CC	1.163	-0.1300 to 2.457	No	ns	0.1365
Lrg1	Lgr5-hi vs. Pro-Senescent CC	1.278	-0.03221 to 2.588	No	ns	0.0650
Lrg1	Lgr5-hi vs. Sec Prog	-1.052	-2.380 to 0.2764	No	ns	0.3212
Lrg1	Lgr5-hi vs. Paneth-like	-2.812	-4.064 to -1.559	Yes	****	<0.0001
Lrg1	Lgr5-hi vs. Goblet	-0.4686	-1.866 to 0.9291	No	ns	0.9990
Lrg1	Lgr5-hi vs. Endocrine	0.6547	-1.296 to 2.605	No	ns	0.9990
Lrg1	Lgr5-hi vs. Tuft	1.300	-1.197 to 3.797	No	ns	0.9220
Lrg1	Lgr5-dim vs. Early TA	0.9193	-0.5984 to 2.437	No	ns	0.7804
Lrg1	Lgr5-dim vs. Abs Prog	-3.118	-4.346 to -1.889	Yes	****	<0.0001
Lrg1	Lgr5-dim vs. Pre-DCC	-2.372	-3.507 to -1.237	Yes	****	<0.0001
Lrg1	Lgr5-dim vs. Pre-PCC	1.854	0.1749 to 3.532	Yes	*	0.0147
Lrg1	Lgr5-dim vs. Pro-DCC	1.242	-0.2958 to 2.779	No	ns	0.2877
Lrg1	Lgr5-dim vs. Mature DCC	2.845	1.833 to 3.857	Yes	****	<0.0001
Lrg1	Lgr5-dim vs. Mature PCC	2.839	1.567 to 4.110	Yes	****	<0.0001
Lrg1	Lgr5-dim vs. P-I CC	2.733	1.384 to 4.083	Yes	****	<0.0001
Lrg1	Lgr5-dim vs. Pro-Senescent CC	2.848	1.482 to 4.213	Yes	****	<0.0001
Lrg1	Lgr5-dim vs. Sec Prog	0.5181	-0.8644 to 1.901	No	ns	0.9964
Lrg1	Lgr5-dim vs. Paneth-like	-1.242	-2.552 to 0.06792	No	ns	0.0859
Lrg1	Lgr5-dim vs. Goblet	1.101	-0.3484 to 2.551	No	ns	0.3957
Lrg1	Lgr5-dim vs. Endocrine	2.224	0.2364 to 4.212	Yes	*	0.0122
Lrg1	Lgr5-dim vs. Tuft	2.870	0.3435 to 5.396	Yes	**	0.0098
Lrg1	Early TA vs. Abs Prog	-4.037	-5.568 to -2.506	Yes	****	<0.0001
Lrg1	Early TA vs. Pre-DCC	-3.291	-4.748 to -1.834	Yes	****	<0.0001
Lrg1	Early TA vs. Pre-PCC	0.9342	-0.9769 to 2.845	No	ns	0.9532
Lrg1	Early TA vs. Pro-DCC	0.3224	-1.466 to 2.111	No	ns	>0.9999
Lrg1	Early TA vs. Mature DCC	1.926	0.5625 to 3.289	Yes	***	0.0002
Lrg1	Early TA vs. Mature PCC	1.919	0.3536 to 3.485	Yes	**	0.0028
Lrg1	Early TA vs. P-I CC	1.814	0.1844 to 3.443	Yes	*	0.0131
Lrg1	Early TA vs. Pro-Senescent CC	1.928	0.2856 to 3.571	Yes	**	0.0059
Lrg1	Early TA vs. Sec Prog	-0.4012	-2.058 to 1.256	No	ns	>0.9999
Lrg1	Early TA vs. Paneth-like	-2.161	-3.758 to -0.5644	Yes	***	0.0004
Lrg1	Early TA vs. Goblet	0.1818	-1.532 to 1.895	No	ns	>0.9999
Lrg1	Early TA vs. Endocrine	1.305	-0.8827 to 3.493	No	ns	0.7997
Lrg1	Early TA vs. Tuft	1.951	-0.7359 to 4.637	No	ns	0.4799
Lrg1	Abs Prog vs. Pre-DCC	0.7460	-0.4067 to 1.899	No	ns	0.6841
Lrg1	Abs Prog vs. Pre-PCC	4.971	3.281 to 6.662	Yes	****	<0.0001
Lrg1	Abs Prog vs. Pro-DCC	4.360	2.809 to 5.910	Yes	****	<0.0001

Lrg1	Abs Prog vs. Mature DCC	5.963	4.931 to 6.994	Yes	****	<0.0001
Lrg1	Abs Prog vs. Mature PCC	5.957	4.669 to 7.244	Yes	****	<0.0001
Lrg1	Abs Prog vs. P-I CC	5.851	4.487 to 7.215	Yes	****	<0.0001
Lrg1	Abs Prog vs. Pro-Senescent CC	5.966	4.586 to 7.346	Yes	****	<0.0001
Lrg1	Abs Prog vs. Sec Prog	3.636	2.239 to 5.033	Yes	****	<0.0001
Lrg1	Abs Prog vs. Paneth-like	1.876	0.5506 to 3.201	Yes	***	0.0001
Lrg1	Abs Prog vs. Goblet	4.219	2.756 to 5.682	Yes	****	<0.0001
Lrg1	Abs Prog vs. Endocrine	5.342	3.344 to 7.340	Yes	****	<0.0001
Lrg1	Abs Prog vs. Tuft	5.988	3.453 to 8.522	Yes	****	<0.0001
Lrg1	Pre-DCC vs. Pre-PCC	4.225	2.602 to 5.849	Yes	****	<0.0001
Lrg1	Pre-DCC vs. Pro-DCC	3.614	2.136 to 5.091	Yes	****	<0.0001
Lrg1	Pre-DCC vs. Mature DCC	5.217	4.299 to 6.135	Yes	****	<0.0001
Lrg1	Pre-DCC vs. Mature PCC	5.211	4.012 to 6.409	Yes	****	<0.0001
Lrg1	Pre-DCC vs. P-I CC	5.105	3.824 to 6.386	Yes	****	<0.0001
Lrg1	Pre-DCC vs. Pro-Senescent CC	5.220	3.922 to 6.517	Yes	****	<0.0001
Lrg1	Pre-DCC vs. Sec Prog	2.890	1.575 to 4.205	Yes	****	<0.0001
Lrg1	Pre-DCC vs. Paneth-like	1.130	-0.1091 to 2.369	No	ns	0.1218
Lrg1	Pre-DCC vs. Goblet	3.473	2.087 to 4.859	Yes	****	<0.0001
Lrg1	Pre-DCC vs. Endocrine	4.596	2.654 to 6.538	Yes	****	<0.0001
Lrg1	Pre-DCC vs. Tuft	5.242	2.751 to 7.732	Yes	****	<0.0001
Lrg1	Pre-PCC vs. Pro-DCC	-0.6118	-2.539 to 1.315	No	ns	0.9995
Lrg1	Pre-PCC vs. Mature DCC	0.9914	-0.5489 to 2.532	No	ns	0.6928
Lrg1	Pre-PCC vs. Mature PCC	0.9851	-0.7370 to 2.707	No	ns	0.8466
Lrg1	Pre-PCC vs. P-I CC	0.8796	-0.9007 to 2.660	No	ns	0.9488
Lrg1	Pre-PCC vs. Pro-Senescent CC	0.9942	-0.7983 to 2.787	No	ns	0.8757
Lrg1	Pre-PCC vs. Sec Prog	-1.335	-3.141 to 0.4701	No	ns	0.4453
Lrg1	Pre-PCC vs. Paneth-like	-3.096	-4.846 to -1.345	Yes	****	<0.0001
Lrg1	Pre-PCC vs. Goblet	-0.7524	-2.610 to 1.105	No	ns	0.9919
Lrg1	Pre-PCC vs. Endocrine	0.3708	-1.932 to 2.673	No	ns	>0.9999
Lrg1	Pre-PCC vs. Tuft	1.016	-1.764 to 3.797	No	ns	0.9973
Lrg1	Pro-DCC vs. Mature DCC	1.603	0.2181 to 2.988	Yes	**	0.0073
Lrg1	Pro-DCC vs. Mature PCC	1.597	0.01207 to 3.182	Yes	*	0.0460
Lrg1	Pro-DCC vs. P-I CC	1.491	-0.1564 to 3.139	No	ns	0.1297
Lrg1	Pro-DCC vs. Pro-Senescent CC	1.606	-0.05503 to 3.267	No	ns	0.0710
Lrg1	Pro-DCC vs. Sec Prog	-0.7236	-2.399 to 0.9515	No	ns	0.9847
Lrg1	Pro-DCC vs. Paneth-like	-2.484	-4.100 to -0.8680	Yes	****	<0.0001
Lrg1	Pro-DCC vs. Goblet	-0.1406	-1.871 to 1.590	No	ns	>0.9999
Lrg1	Pro-DCC vs. Endocrine	0.9826	-1.219 to 3.184	No	ns	0.9791
Lrg1	Pro-DCC vs. Tuft	1.628	-1.069 to 4.326	No	ns	0.7850
Lrg1	Mature DCC vs. Mature PCC	-0.006222	-1.089 to 1.076	No	ns	>0.9999
Lrg1	Mature DCC vs. P-I CC	-0.1117	-1.284 to 1.061	No	ns	>0.9999
Lrg1	Mature DCC vs. Pro-Senescent CC	0.002806	-1.188 to 1.194	No	ns	>0.9999
Lrg1	Mature DCC vs. Sec Prog	-2.327	-3.538 to -1.116	Yes	****	<0.0001
Lrg1	Mature DCC vs. Paneth-like	-4.087	-5.214 to -2.960	Yes	****	<0.0001
Lrg1	Mature DCC vs. Goblet	-1.744	-3.031 to -0.4571	Yes	***	0.0004
Lrg1	Mature DCC vs. Endocrine	-0.6205	-2.493 to 1.252	No	ns	0.9991
Lrg1	Mature DCC vs. Tuft	0.02503	-2.412 to 2.462	No	ns	>0.9999
Lrg1	Mature PCC vs. P-I CC	-0.1055	-1.509 to 1.298	No	ns	>0.9999
Lrg1	Mature PCC vs. Pro-Senescent CC	0.009028	-1.409 to 1.428	No	ns	>0.9999
Lrg1	Mature PCC vs. Sec Prog	-2.321	-3.756 to -0.8856	Yes	****	<0.0001
Lrg1	Mature PCC vs. Paneth-like	-4.081	-5.446 to -2.715	Yes	****	<0.0001
Lrg1	Mature PCC vs. Goblet	-1.738	-3.237 to -0.2379	Yes	**	0.0072
Lrg1	Mature PCC vs. Endocrine	-0.6143	-2.639 to 1.411	No	ns	0.9997
Lrg1	Mature PCC vs. Tuft	0.03125	-2.524 to 2.587	No	ns	>0.9999
Lrg1	P-I CC vs. Pro-Senescent CC	0.1145	-1.374 to 1.603	No	ns	>0.9999
Lrg1	P-I CC vs. Sec Prog	-2.215	-3.719 to -0.7108	Yes	****	<0.0001
Lrg1	P-I CC vs. Paneth-like	-3.975	-5.413 to -2.537	Yes	****	<0.0001
Lrg1	P-I CC vs. Goblet	-1.632	-3.198 to -0.06594	Yes	*	0.0311
Lrg1	P-I CC vs. Endocrine	-0.5088	-2.583 to 1.566	No	ns	>0.9999

Lrg1	P-I CC vs. Tuft	0.1368	-2.458 to 2.732	No	ns	>0.9999
Lrg1	Pro-Senescent CC vs. Sec Prog	-2.330	-3.848 to -0.8110	Yes	****	<0.0001
Lrg1	Pro-Senescent CC vs. Paneth-like	-4.090	-5.543 to -2.637	Yes	****	<0.0001
Lrg1	Pro-Senescent CC vs. Goblet	-1.747	-3.327 to -0.1667	Yes	*	0.0144
Lrg1	Pro-Senescent CC vs. Endocrine	-0.6233	-2.708 to 1.462	No	ns	0.9997
Lrg1	Pro-Senescent CC vs. Tuft	0.02222	-2.581 to 2.626	No	ns	>0.9999
Lrg1	Sec Prog vs. Paneth-like	-1.760	-3.229 to -0.2911	Yes	**	0.0042
Lrg1	Sec Prog vs. Goblet	0.5830	-1.012 to 2.178	No	ns	0.9973
Lrg1	Sec Prog vs. Endocrine	1.706	-0.3899 to 3.802	No	ns	0.2747
Lrg1	Sec Prog vs. Tuft	2.352	-0.2606 to 4.964	No	ns	0.1355
Lrg1	Paneth-like vs. Goblet	2.343	0.8109 to 3.875	Yes	****	<0.0001
Lrg1	Paneth-like vs. Endocrine	3.466	1.417 to 5.515	Yes	****	<0.0001
Lrg1	Paneth-like vs. Tuft	4.112	1.537 to 6.687	Yes	****	<0.0001
Lrg1	Goblet vs. Endocrine	1.123	-1.018 to 3.264	No	ns	0.9173
Lrg1	Goblet vs. Tuft	1.769	-0.8797 to 4.417	No	ns	0.6318
Lrg1	Endocrine vs. Tuft	0.6456	-2.332 to 3.623	No	ns	>0.9999

Gene	Tukey's multiple comparisons test	Mean Diff.	95.00% CI of diff.	Below threshold?	Summary	Adjusted P Value
Fut2	Lgr5-hi vs. Lgr5-dim	-0.1589	-0.5292 to 0.2114	No	ns	0.9856
Fut2	Lgr5-hi vs. Early TA	-0.07905	-0.5520 to 0.3939	No	ns	>0.9999
Fut2	Lgr5-hi vs. Abs Prog	-0.9137	-1.290 to -0.5377	Yes	****	<0.0001
Fut2	Lgr5-hi vs. Pre-DCC	-1.297	-1.641 to -0.9532	Yes	****	<0.0001
Fut2	Lgr5-hi vs. Pre-PCC	-0.1321	-0.6585 to 0.3943	No	ns	>0.9999
Fut2	Lgr5-hi vs. Pro-DCC	-0.5237	-1.003 to -0.04419	Yes	*	0.0171
Fut2	Lgr5-hi vs. Mature DCC	0.1903	-0.1112 to 0.4918	No	ns	0.7229
Fut2	Lgr5-hi vs. Mature PCC	0.09032	-0.3002 to 0.4808	No	ns	>0.9999
Fut2	Lgr5-hi vs. P-I CC	0.2134	-0.2032 to 0.6301	No	ns	0.9313
Fut2	Lgr5-hi vs. Pro-Senescent CC	0.3760	-0.04603 to 0.7981	No	ns	0.1473
Fut2	Lgr5-hi vs. Sec Prog	-2.740	-3.167 to -2.312	Yes	****	<0.0001
Fut2	Lgr5-hi vs. Paneth-like	-4.340	-4.743 to -3.936	Yes	****	<0.0001
Fut2	Lgr5-hi vs. Goblet	-0.4098	-0.8600 to 0.04042	No	ns	0.1237
Fut2	Lgr5-hi vs. Endocrine	-0.02365	-0.6519 to 0.6046	No	ns	>0.9999
Fut2	Lgr5-hi vs. Tuft	0.2827	-0.5217 to 1.087	No	ns	0.9982
Fut2	Lgr5-dim vs. Early TA	0.07989	-0.4090 to 0.5688	No	ns	>0.9999
Fut2	Lgr5-dim vs. Abs Prog	-0.7547	-1.150 to -0.3590	Yes	****	<0.0001
Fut2	Lgr5-dim vs. Pre-DCC	-1.138	-1.504 to -0.7727	Yes	****	<0.0001
Fut2	Lgr5-dim vs. Pre-PCC	0.02686	-0.5139 to 0.5676	No	ns	>0.9999
Fut2	Lgr5-dim vs. Pro-DCC	-0.3648	-0.8600 to 0.1305	No	ns	0.4530
Fut2	Lgr5-dim vs. Mature DCC	0.3492	0.02332 to 0.6752	Yes	*	0.0220
Fut2	Lgr5-dim vs. Mature PCC	0.2493	-0.1604 to 0.6589	No	ns	0.7744
Fut2	Lgr5-dim vs. P-I CC	0.3724	-0.06227 to 0.8070	No	ns	0.1972
Fut2	Lgr5-dim vs. Pro-Senescent CC	0.5350	0.09514 to 0.9748	Yes	**	0.0032
Fut2	Lgr5-dim vs. Sec Prog	-2.581	-3.026 to -2.135	Yes	****	<0.0001
Fut2	Lgr5-dim vs. Paneth-like	-4.181	-4.603 to -3.759	Yes	****	<0.0001
Fut2	Lgr5-dim vs. Goblet	-0.2509	-0.7178 to 0.2161	No	ns	0.9011
Fut2	Lgr5-dim vs. Endocrine	0.1353	-0.5051 to 0.7756	No	ns	>0.9999
Fut2	Lgr5-dim vs. Tuft	0.4416	-0.3722 to 1.255	No	ns	0.8936
Fut2	Early TA vs. Abs Prog	-0.8346	-1.328 to -0.3415	Yes	****	<0.0001
Fut2	Early TA vs. Pre-DCC	-1.218	-1.688 to -0.7489	Yes	****	<0.0001
Fut2	Early TA vs. Pre-PCC	-0.05303	-0.6686 to 0.5626	No	ns	>0.9999
Fut2	Early TA vs. Pro-DCC	-0.4447	-1.021 to 0.1314	No	ns	0.3664
Fut2	Early TA vs. Mature DCC	0.2694	-0.1697 to 0.7084	No	ns	0.7635
Fut2	Early TA vs. Mature PCC	0.1694	-0.3350 to 0.6737	No	ns	0.9990
Fut2	Early TA vs. P-I CC	0.2925	-0.2324 to 0.8174	No	ns	0.8715
Fut2	Early TA vs. Pro-Senescent CC	0.4551	-0.07409 to 0.9842	No	ns	0.1921
Fut2	Early TA vs. Sec Prog	-2.660	-3.194 to -2.127	Yes	****	<0.0001
Fut2	Early TA vs. Paneth-like	-4.261	-4.775 to -3.746	Yes	****	<0.0001
Fut2	Early TA vs. Goblet	-0.3307	-0.8826 to 0.2211	No	ns	0.7939

Fut2	Early TA vs. Endocrine	0.05540	-0.6493 to 0.7601	No	ns	>0.9999
Fut2	Early TA vs. Tuft	0.3617	-0.5036 to 1.227	No	ns	0.9889
Fut2	Abs Prog vs. Pre-DCC	-0.3836	-0.7549 to -0.01232	Yes	*	0.0345
Fut2	Abs Prog vs. Pre-PCC	0.7816	0.2370 to 1.326	Yes	***	0.0001
Fut2	Abs Prog vs. Pro-DCC	0.3899	-0.1095 to 0.8894	No	ns	0.3462
Fut2	Abs Prog vs. Mature DCC	1.104	0.7717 to 1.436	Yes	****	<0.0001
Fut2	Abs Prog vs. Mature PCC	1.004	0.5893 to 1.419	Yes	****	<0.0001
Fut2	Abs Prog vs. P-I CC	1.127	0.6877 to 1.567	Yes	****	<0.0001
Fut2	Abs Prog vs. Pro-Senescent CC	1.290	0.8451 to 1.734	Yes	****	<0.0001
Fut2	Abs Prog vs. Sec Prog	-1.826	-2.276 to -1.376	Yes	****	<0.0001
Fut2	Abs Prog vs. Paneth-like	-3.426	-3.853 to -2.999	Yes	****	<0.0001
Fut2	Abs Prog vs. Goblet	0.5039	0.03250 to 0.9752	Yes	*	0.0227
Fut2	Abs Prog vs. Endocrine	0.8900	0.2464 to 1.534	Yes	***	0.0002
Fut2	Abs Prog vs. Tuft	1.196	0.3800 to 2.013	Yes	****	<0.0001
Fut2	Pre-DCC vs. Pre-PCC	1.165	0.6421 to 1.688	Yes	****	<0.0001
Fut2	Pre-DCC vs. Pro-DCC	0.7735	0.2976 to 1.249	Yes	****	<0.0001
Fut2	Pre-DCC vs. Mature DCC	1.488	1.192 to 1.783	Yes	****	<0.0001
Fut2	Pre-DCC vs. Mature PCC	1.388	1.002 to 1.774	Yes	****	<0.0001
Fut2	Pre-DCC vs. P-I CC	1.511	1.098 to 1.923	Yes	****	<0.0001
Fut2	Pre-DCC vs. Pro-Senescent CC	1.673	1.255 to 2.091	Yes	****	<0.0001
Fut2	Pre-DCC vs. Sec Prog	-1.442	-1.866 to -1.019	Yes	****	<0.0001
Fut2	Pre-DCC vs. Paneth-like	-3.042	-3.442 to -2.643	Yes	****	<0.0001
Fut2	Pre-DCC vs. Goblet	0.8875	0.4411 to 1.334	Yes	****	<0.0001
Fut2	Pre-DCC vs. Endocrine	1.274	0.6481 to 1.899	Yes	****	<0.0001
Fut2	Pre-DCC vs. Tuft	1.580	0.7778 to 2.382	Yes	****	<0.0001
Fut2	Pre-PCC vs. Pro-DCC	-0.3917	-1.012 to 0.2290	No	ns	0.7232
Fut2	Pre-PCC vs. Mature DCC	0.3224	-0.1738 to 0.8185	No	ns	0.6776
Fut2	Pre-PCC vs. Mature PCC	0.2224	-0.3324 to 0.7771	No	ns	0.9927
Fut2	Pre-PCC vs. P-I CC	0.3455	-0.2280 to 0.9190	No	ns	0.7872
Fut2	Pre-PCC vs. Pro-Senescent CC	0.5081	-0.06930 to 1.085	No	ns	0.1622
Fut2	Pre-PCC vs. Sec Prog	-2.607	-3.189 to -2.026	Yes	****	<0.0001
Fut2	Pre-PCC vs. Paneth-like	-4.208	-4.772 to -3.644	Yes	****	<0.0001
Fut2	Pre-PCC vs. Goblet	-0.2777	-0.8760 to 0.3206	No	ns	0.9701
Fut2	Pre-PCC vs. Endocrine	0.1084	-0.6332 to 0.8500	No	ns	>0.9999
Fut2	Pre-PCC vs. Tuft	0.4148	-0.4809 to 1.310	No	ns	0.9708
Fut2	Pro-DCC vs. Mature DCC	0.7140	0.2679 to 1.160	Yes	****	<0.0001
Fut2	Pro-DCC vs. Mature PCC	0.6140	0.1035 to 1.125	Yes	**	0.0039
Fut2	Pro-DCC vs. P-I CC	0.7372	0.2064 to 1.268	Yes	***	0.0002
Fut2	Pro-DCC vs. Pro-Senescent CC	0.8997	0.3647 to 1.435	Yes	****	<0.0001
Fut2	Pro-DCC vs. Sec Prog	-2.216	-2.755 to -1.676	Yes	****	<0.0001
Fut2	Pro-DCC vs. Paneth-like	-3.816	-4.336 to -3.296	Yes	****	<0.0001
Fut2	Pro-DCC vs. Goblet	0.1139	-0.4436 to 0.6715	No	ns	>0.9999
Fut2	Pro-DCC vs. Endocrine	0.5001	-0.2091 to 1.209	No	ns	0.5343
Fut2	Pro-DCC vs. Tuft	0.8064	-0.06256 to 1.675	No	ns	0.1046
Fut2	Mature DCC vs. Mature PCC	-0.09998	-0.4487 to 0.2487	No	ns	0.9998
Fut2	Mature DCC vs. P-I CC	0.02315	-0.3546 to 0.4009	No	ns	>0.9999
Fut2	Mature DCC vs. Pro-Senescent CC	0.1857	-0.1980 to 0.5694	No	ns	0.9569
Fut2	Mature DCC vs. Sec Prog	-2.930	-3.320 to -2.540	Yes	****	<0.0001
Fut2	Mature DCC vs. Paneth-like	-4.530	-4.893 to -4.167	Yes	****	<0.0001
Fut2	Mature DCC vs. Goblet	-0.6001	-1.015 to -0.1856	Yes	****	<0.0001
Fut2	Mature DCC vs. Endocrine	-0.2140	-0.8171 to 0.3892	No	ns	0.9981
Fut2	Mature DCC vs. Tuft	0.09238	-0.6925 to 0.8773	No	ns	>0.9999
Fut2	Mature PCC vs. P-I CC	0.1231	-0.3288 to 0.5751	No	ns	>0.9999
Fut2	Mature PCC vs. Pro-Senescent CC	0.2857	-0.1712 to 0.7426	No	ns	0.7367
Fut2	Mature PCC vs. Sec Prog	-2.830	-3.292 to -2.368	Yes	****	<0.0001
Fut2	Mature PCC vs. Paneth-like	-4.430	-4.870 to -3.990	Yes	****	<0.0001
Fut2	Mature PCC vs. Goblet	-0.5001	-0.9832 to -0.01704	Yes	*	0.0337
Fut2	Mature PCC vs. Endocrine	-0.1140	-0.7662 to 0.5383	No	ns	>0.9999
Fut2	Mature PCC vs. Tuft	0.1924	-0.6308 to 1.016	No	ns	>0.9999

Fut2	P-I CC vs. Pro-Senescent CC	0.1626	-0.3169 to 0.6421	No	ns	0.9988
Fut2	P-I CC vs. Sec Prog	-2.953	-3.438 to -2.468	Yes	****	<0.0001
Fut2	P-I CC vs. Paneth-like	-4.553	-5.016 to -4.090	Yes	****	<0.0001
Fut2	P-I CC vs. Goblet	-0.6232	-1.128 to -0.1188	Yes	**	0.0025
Fut2	P-I CC vs. Endocrine	-0.2371	-0.9053 to 0.4311	No	ns	0.9981
Fut2	P-I CC vs. Tuft	0.06923	-0.7667 to 0.9052	No	ns	>0.9999
Fut2	Pro-Senescent CC vs. Sec Prog	-3.116	-3.605 to -2.626	Yes	****	<0.0001
Fut2	Pro-Senescent CC vs. Paneth-like	-4.716	-5.184 to -4.248	Yes	****	<0.0001
Fut2	Pro-Senescent CC vs. Goblet	-0.7858	-1.295 to -0.2769	Yes	****	<0.0001
Fut2	Pro-Senescent CC vs. Endocrine	-0.3997	-1.071 to 0.2719	No	ns	0.8026
Fut2	Pro-Senescent CC vs. Tuft	-0.09333	-0.9319 to 0.7453	No	ns	>0.9999
Fut2	Sec Prog vs. Paneth-like	-1.600	-2.073 to -1.127	Yes	****	<0.0001
Fut2	Sec Prog vs. Goblet	2.330	1.816 to 2.843	Yes	****	<0.0001
Fut2	Sec Prog vs. Endocrine	2.716	2.041 to 3.391	Yes	****	<0.0001
Fut2	Sec Prog vs. Tuft	3.022	2.181 to 3.864	Yes	****	<0.0001
Fut2	Paneth-like vs. Goblet	3.930	3.436 to 4.423	Yes	****	<0.0001
Fut2	Paneth-like vs. Endocrine	4.316	3.656 to 4.976	Yes	****	<0.0001
Fut2	Paneth-like vs. Tuft	4.622	3.793 to 5.452	Yes	****	<0.0001
Fut2	Goblet vs. Endocrine	0.3861	-0.3035 to 1.076	No	ns	0.8673
Fut2	Goblet vs. Tuft	0.6925	-0.1607 to 1.546	No	ns	0.2794
Fut2	Endocrine vs. Tuft	0.3063	-0.6528 to 1.265	No	ns	0.9994

Supplementary Table 8. Primers used for RT-PCR.

Gene name	Forward Primer	Reverse Primer
Cxcl2	GCT GTC AAT GCC TGA AGA	TTC AGG GTC AAG GCA AAC
Cxcl5	TCT TGT CCA CAA TGA GCC TCC A	AAC AGC AAC AGA AAT GCC AGC G
Dpep1	ACA AAG ATG CCG TGA AGA G	CCA AGG CTG CTG TCA ATT A
eae	CCA AAG GAA TCG GAG TGT AGT T	TAG GTG GCA AGC TGA TGT ATG
Fabp2	CGG TGT AAA CTT TCC CTA CAG	TGG CCT CAA CTC CTT CAT A
Fut2	CCC ACT TCC TCA TCT TTG TC	CGC CTG TAA TTC CTT CTC TG
Gapdh	TCC ATG ACA ACT TTG GCA TTG	CAG TCT GGG TGG CAG TGA
Guca2a	GCA CCA CAG CTA TGT AGT AG	GAG AAA GGC AAG CGA TGT
Il10rb	AGC CGT GGA CAA CTT ACT GCA T	TTC GTC ATT GCC TGT CCG TTC A
Il22ra1	CAC CGT CTA CAG TGT GGA ATA TAA	CGT GAC CTT GGC GTA GTA AA
Lbp	CAG CCG CAT TTG TGA TTT G	TGG CAG AGT CTG GAG ATA AG
Lcn2	TTT CAC CCG CTT TGC CAA GTC T	CAC ACT CAC CAC CCA TTC AGT TGT
Lrg1	CCT CAA GGA ATG CCT GAT AC	GAG AAT TCC ACC GAC AGA TG
Ly6g	GAC TTC CTG CAA CAC AAC TA	TCA CGT TGA CAG CAT TAC C
Muc1	GCA TAA GAA GGA GGC AGA TG	GGG CAA GGA AAT AGA CGA TAG
Muc2	AAG TGG CAT TGT GTG CCA ACC A	TGC AGC ACT TGT CAT CTG GGT T
Reg3b	ATG GCT CCT ACT GCT CC	GTG TCC TCC AGG CCT CTT T
S100a8	TGA GTG TCC TCA GTT TGT GCA G	TGT GAG ATG CCA CAC CCA CTT T
Saa1	ATT GCT GAC CAG GAA GCC AAC A	AGG ACG CTC AGT ATT TGT CAG GCA
Slc20a1	TTC CCA TCA GCA CAA CAC	CCA GTC AAC AGC CTT CTT T
Slc26a3	GCG TGT ACT CCC TCA AAT AC	GCT CCC TGC AAA TCC TTT
Slc37a2	GCC TTC CTA GTG GCT TAT G	CCA AAG AGG GAG GTG AAT AG
Slc46a1	GGC ATC TTC AAC TCC ATC TAC	AAC TCC GGG TGT GGA TTA
