# Supplementary material for: Distal colonocytes targeted by C. rodentium recruit T-cell help for barrier defence
Source: Nature. 2024 Apr 10;629(8012):669–78. doi: 10.1038/s41586-024-07288-1 (PMC11096101; doi:10.1038/s41586-024-07288-1)
Supplement: Supplementary file 2 — Reporting Summary [file 41586_2024_7288_MOESM2_ESM.pdf]

nature portfolio

Corresponding author(s): DAPR and Nature 2023-04-07013

Last updated by author(s): May 8, 2023

# Reporting Summary

Nature Portfolio wishes to improve the reproducibility of the work that we publish. This form provides structure for consistency and transparency in reporting. For further information on Nature Portfolio policies, see our Editorial Policies and the Editorial Policy Checklist.

Please do not complete any field with "not applicable" or n/a. Refer to the help text for what text to use if an item is not relevant to your study.
For final submission: please carefully check your responses for accuracy; you will not be able to make changes later.

## Statistics

For all statistical analyses, confirm that the following items are present in the figure legend, table legend, main text, or Methods section.

| n/a | Confirmed | |
|---|---|---|
| ☐ | ☒ | The exact sample size (*n*) for each experimental group/condition, given as a discrete number and unit of measurement |
| ☐ | ☒ | A statement on whether measurements were taken from distinct samples or whether the same sample was measured repeatedly |
| ☐ | ☒ | The statistical test(s) used AND whether they are one- or two-sided<br>*Only common tests should be described solely by name; describe more complex techniques in the Methods section.* |
| ☒ | ☐ | A description of all covariates tested |
| ☐ | ☒ | A description of any assumptions or corrections, such as tests of normality and adjustment for multiple comparisons |
| ☐ | ☒ | A full description of the statistical parameters including central tendency (e.g. means) or other basic estimates (e.g. regression coefficient) AND variation (e.g. standard deviation) or associated estimates of uncertainty (e.g. confidence intervals) |
| ☐ | ☒ | For null hypothesis testing, the test statistic (e.g. *F*, *t*, *r*) with confidence intervals, effect sizes, degrees of freedom and *P* value noted<br>*Give P values as exact values whenever suitable.* |
| ☒ | ☐ | For Bayesian analysis, information on the choice of priors and Markov chain Monte Carlo settings |
| ☒ | ☐ | For hierarchical and complex designs, identification of the appropriate level for tests and full reporting of outcomes |
| ☒ | ☐ | Estimates of effect sizes (e.g. Cohen's *d*, Pearson's *r*), indicating how they were calculated |

*Our web collection on statistics for biologists contains articles on many of the points above.*

## Software and code

Policy information about availability of computer code

| | |
|---|---|
| Data collection | Cell Ranger pipeline software (v.3.0.2; 10x Genomics)<br>Seurat132 (v.3.0.0), using the R package |
| Data analysis | ComplexHeatmap (v2.11.1) package in R<br>The fgsea R package (v1.4.0)<br>R package ggplot2 (v3.3.5)<br>scVelo package (v0.2.2) with Scanpy (v1.6.1) on Python<br>(v3.8.5) velocyto (v0.17.16)<br>qiime2 (v2023.9)<br>SILVA database (release 138.1) |

For manuscripts utilizing custom algorithms or software that are central to the research but not yet described in published literature, software must be made available to editors and reviewers. We strongly encourage code deposition in a community repository (e.g. GitHub). See the Nature Portfolio guidelines for submitting code & software for further information.

# Data

Policy information about availability of data

All manuscripts must include a data availability statement. This statement should provide the following information, where applicable:

- Accession codes, unique identifiers, or web links for publicly available datasets
- A description of any restrictions on data availability
- For clinical datasets or third party data, please ensure that the statement adheres to our policy

The scRNA-seq data used in this study has been deposited in the Gene Expression Omnibus database under the accession number GSE227331. All other data generated in this study are provided within the article and its Supplementary Information/Source Data file. The shell, R and Python scripts that enabled the main steps of the analyses performed in this project are available on request.

# Research involving human participants, their data, or biological material

Policy information about studies with human participants or human data. See also policy information about sex, gender (identity/presentation), and sexual orientation and race, ethnicity and racism.

| | |
|---|---|
| Reporting on sex and gender | N/A |
| Reporting on race, ethnicity, or other socially relevant groupings | N/A |
| Population characteristics | N/A |
| Recruitment | N/A |
| Ethics oversight | N/A |

Note that full information on the approval of the study protocol must also be provided in the manuscript.

# Field-specific reporting

Please select the one below that is the best fit for your research. If you are not sure, read the appropriate sections before making your selection.

☒ Life sciences ☐ Behavioural & social sciences ☐ Ecological, evolutionary & environmental sciences

For a reference copy of the document with all sections, see nature.com/documents/nr-reporting-summary-flat.pdf

# Life sciences study design

All studies must disclose on these points even when the disclosure is negative.

| | |
|---|---|
| Sample size | In vivo animal experiments use group sizes of 4-6 animals and are repeated. This typical group size is based on empirical power analysis, using one-way ANOVA and empirical data; analyzing at least 4 mice/group provides 90% power to detect an effect size of 1.33 (or a 30% change) where P < 0.05. scRNA-seq data were generated from cells sorted from pools of tissues representing 2-3 animals per group and n=2 biological replicates. Experiments that required sorting of epithelial cells from pools of tissues from 2-3 animals were run in duplicate with a maximum of 4 groups to limit cell death after sorting. |
| Data exclusions | Data exclusion was considered when animals appeared sick. |
| Replication | All experimental findings were repeated and successful. Where possible, data represents first and repeat experiments combined. |
| Randomization | Cohorts of adult mice of similar age group (8-12 weeks old) were randomly assigned to either control or infected groups of at least 4 mice per group. |
| Blinding | Blinding is not possible due to safety reasons and potential cross-contamination between infected and non-infected naive animals. |

# Reporting for specific materials, systems and methods

We require information from authors about some types of materials, experimental systems and methods used in many studies. Here, indicate whether each material, system or method listed is relevant to your study. If you are not sure if a list item applies to your research, read the appropriate section before selecting a response.

## Materials & experimental systems

| n/a | Involved in the study | |
|---|---|---|
| ☐ | ☒ | Antibodies |
| ☒ | ☐ | Eukaryotic cell lines |
| ☒ | ☐ | Palaeontology and archaeology |
| ☐ | ☒ | Animals and other organisms |
| ☒ | ☐ | Clinical data |
| ☒ | ☐ | Dual use research of concern |
| ☒ | ☐ | Plants |

## Methods

| n/a | Involved in the study | |
|---|---|---|
| ☒ | ☐ | ChIP-seq |
| ☐ | ☒ | Flow cytometry |
| ☒ | ☐ | MRI-based neuroimaging |

# Antibodies

Antibodies used

Rat anti-mouse Ly6G-Alexa Fluor594 (1A8); Biolegend 127636
Rat anti-mouse Ly6G-Biotin (1A8); ThermoFisher 13-9668-82
Rat anti-mouse Ly6G-APC (1A8); Biolegend 127614
Goat anti-mouse/rat FABP2; R&D/Fisher AF1486 (polyclonal)
Donkey Anti-goat Alexa Fluor488; ThermoFisher A-11008
Donkey Anti-goat Alexa Fluor647; ThermoFisher A-21447
SA-Alexa Fluor594; ThermoFisher S32356
Rabbit Anti-C.r-LPS; Accurate Chemical and Scientific of Denka Seiken Co., Ltd YCC312-012
Rabbit Anti-GFP; ThermoFisher A-11122
Goat Anti-rabbit Alexa Fluor488; ThermoFisher A-11008
Goat Anti-rabbit Alexa Fluor594; ThermoFisher A-11037
Rabbit Anti-Fluorescein Alexa Fluor488; ThermoFisher A-11090
Rat anti-mouse CD45 PE (30-F11); Biolegend 103106
Rat anti-mouse EpCAM1 PE-Cy7 (G8.8); ThermoFisher 25-5791-80
Rat anti-mouse EpCAM1 FITC (G8.8); ThermoFisher 11-5791-82
Rat anti-mouse EpCAM1 Biotin (G8.8); ThermoFisher 13-5791-82
Rat anti-mouse MHCII eFluor450 (M5/114.15.2); (I-A/I-E); Fisher 50-163-69
Rabbit anti-pSTAT3 (Tyr705; Clone D3A7); Cell Signaling 9145P
Rat anti-mouse CD4 FITC (RM4-5); BioLegend 100510
Armenian hamster anti-mouse/human TCRb (H57-957); ThermoFisher 17-5961-82
Rat anti-mouse CD44 BV510 (IM7); BioLegend 103044
Rat anti-mouse CD45.1 PE-Cy7 (A20); ThermoFisher 25-0453-82
Mouse anti-mouse CD45.2 BV711 (104); BD Biosciences
563685
Rat anti-mouse IFNg eFluor450 (XMG1.2); ThermoFisher 48-7311-82
Rat anti-mouse IL-22 PE (1H8PWSR); ThermoFisher/eBioscience 12-7221-82
Rat anti-mouse IL-17A FITC (eBio17B7); ThermoFisher/eBioscience 11-7177-81
Rat anti-mouse IL-17A PerCP-Cy5.5 (TC11-18H10); BD Biosciences 560666
Live/Dead Fixable Near-IR dead cell dye (ThermoFisher)
Rat anti-BrdU (BU1/75); Abcam ab6326
Goat anti-HA Tag (polyclonal); Fisher/Novus Biologicals NB600362
Streptavidin-Alexa Fluor 594; ThermoFisher S32356
Prolong Diamond antifade mountant with DAPI; ThermoFisher P36962

Validation

See manufacturer's websites and publication: Zindl et al., Immunity (2022) 55: 494-511.

# Animals and other research organisms

Policy information about studies involving animals; ARRIVE guidelines recommended for reporting animal research, and Sex and Gender in Research

Laboratory animals

Il22hCD4.fl reporter/floxed and Il22ΔTcell cKO mice were previously generated within our laboratory (Zindl et al., Immunity (2022) 55:494-511). C57BL/6 (WT), C3H/HeJ, H2-Ab1 floxed, mCd4-cre, SMARTA-1 CD45.1, Tcra–/–, Villin-cre and Villin-cre/ERT2 mice were purchased from Jackson Laboratory. Experimental adult animals (8-12 wk old) were co-caged in groups of 2-7 mice.

Wild animals

This study did not involve wild animals.

Reporting on sex

Both sexes were used per experimental group whenever possible.

Field-collected samples

This study did not include field-collected samples.

Ethics oversight

All mouse strains were bred and maintained at UAB in accordance with IACUC guidelines.

Note that full information on the approval of the study protocol must also be provided in the manuscript.

# Flow Cytometry

## Plots

Confirm that:

☒ The axis labels state the marker and fluorochrome used (e.g. CD4-FITC).

☒ The axis scales are clearly visible. Include numbers along axes only for bottom left plot of group (a 'group' is an analysis of identical markers).

☒ All plots are contour plots with outliers or pseudocolor plots.

☒ A numerical value for number of cells or percentage (with statistics) is provided.

## Methodology

Sample preparation

Intestinal tissue was flushed, cut into regions (5 cm ileum, 4 cm mid-distal colon or 2 cm each for proximal, middle, or distal colon), opened longitudinally and then cut into strips of 1 cm length. Tissue pieces were incubated for 20 min at37°C with 1 mM DTT (Sigma), followed by 2 mM EDTA (Invitrogen) in H5H media (1x HBSS, 5% FBS, 20 mM Hepes, and 2.5 mM 2-β-ME). Tissue pieces were vortexed briefly after each 20 min incubation, followed by washing with H5H prior to centrifugation at 1800 rpm for 10 min at 4°C. IECs were then purified on a 40%/75% Percoll gradient by centrifugation for 20 min at 25°C and 2000 rpm with no brake. For analysis of C.r-GFP attached to IECs, tissue pieces from 4 cm of mid-distal colon were incubated for 20 min at 37°C with 1 mM EDTA in H5H media, followed by gentle mixing and washing with H5H. Colonic IECs were stained with Fc Block (Clone 2.4G2) followed by staining with fluorescent-labeled antibodies in IEC buffer (1x PBS with 5% FBS and 2mM EDTA to reduce cell clumping) on ice in 1.5 ml microcentrifuge tubes. For intracellular staining, cells were fixed and permeabilized using BD Cytofix/Cytoperm kit (BD Bioscience). Colonic lamina propria CD4 T cells were isolated by flushing colons with 1x PBS, opening longitudinally and cutting into small pieces and placed in H5H media. Tissue was then minced in
1.5 ml microcentrifuge tubes for 2-3 min before being transferred into scintillation vials with 10 ml of complete R10 media (1x RPMI 1640, 10% FBS, 1x Pen/Strep, 1x NEAA, 1mM Sodium pyruvate, 2 mM L-glutamine, and 2.5 mM 2-β-ME) with collagenase IV (Sigma, 100 U/ml) and DNase I (Sigma, 20 mg/mL). Tissue was digested at 37C for 40 min with stirring followed by filtering over a 70 um filter and washing with complete R10 media. Cells were then centrifuged at 1750 rpm at 4C for 10 min and then purified on a 40%/75% Percoll as described above. Where indicated, cells were stimulated with PMA (50 ng/mL) and Ionomycin (750 ng/mL) at 37□C for 4 hrs in the presence of GolgiPlug (BD Biosciences).

Instrument

Samples were acquired on an Attune NxT flow cytometer (Life Technologies) or sorted on either a BD FACS Aria or Aria II (BD Biosciences).

Software

FlowJo 10.8.1

Cell population abundance

A portion of sorted cells were resorted to confirm >95% purity.

Gating strategy

FSC/SSC included all cells (lymphocytes and epithelial cells) except small debris

IEC gating strategy:
1. Live cells were gated as LIVE/DEAD Fixable Near-IR Dead Cell dye (ThermoFisher L34976) negative, live cells were gated
2.CD45-PE negative, EpCAM1-PE-Cy7 positive epithelial cells were analyzed for C.r-GFP or Ly6G-APC or FABP2 followed by anti-goat Alexa Fluor488.

Colonic LP T cell gating strategy:
1. Colonic LP CD4 T cells were gated as LIVE/DEAD dye negative, CD4+ live cells were gated
2. TCRb+ cells from the live CD4+ population were gated
3. TCRb+ cells were then gated on IFNg+, IL-17A+, or IL-17A+IFNg+ by quadrant gate or were gated on CD44+IL-22+

Colonic LP T cell gating strategy from SMARTA-1 CD45.1+ adoptive transfer experiments:
1. Colonic LP CD4 T cells were gated as LIVE/DEAD dye negative, CD4+ live cells were gated
2. TCRb+ cells from the live CD4+ population were gated
3. TCRb+ cells were then gated on CD45.1+; CD45.2+ cells were excluded
4. Finally, CD45.1+ cells were then gated on IFNg+, IL-17A+, or IL-17A+IFNg+ or were gated on CD44+IL-22+

☒ Tick this box to confirm that a figure exemplifying the gating strategy is provided in the Supplementary Information.

This checklist template is licensed under a Creative Commons Attribution 4.0 International License, which permits use, sharing, adaptation, distribution and reproduction in any medium or format, as long as you give appropriate credit to the original author(s) and the source, provide a link to the Creative Commons license, and indicate if changes were made. The images or other third party material in this article are included in the article's Creative Commons license, unless indicated otherwise in a credit line to the material. If material is not included in the article's Creative Commons license and your intended use is not permitted by statutory regulation or exceeds the permitted use, you will need to obtain permission directly from the copyright holder. To view a copy of this license, visit http://creativecommons.org/licenses/by/4.0/
